# The *Mecyclothorax* beetles (Coleoptera, Carabidae, Moriomorphini) of Tahiti, Society Islands

**DOI:** 10.3897/zookeys.322.5492

**Published:** 2013-08-09

**Authors:** James K. Liebherr

**Affiliations:** 1 Cornell University Insect Collection, Department of Entomology, 2144 John H. and Anna B. Comstock Hall, Cornell University, Ithaca, NY 14853-2601, USA

**Keywords:** Allopatric speciation, biodiversity, biogeography, genitalic evolution, revisionary systematics

## Abstract

The 101 species of *Mecyclothorax* Sharp known to inhabit Tahiti Island, French Polynesia are taxonomically revised, including 28 species that are newly described: *Mecyclothorax claridgeiae* **sp. n.**, *Mecyclothorax jeanyvesi* **sp. n.**, *Mecyclothorax poria* **sp. n.**, *Mecyclothorax aano* **sp. n.**, *Mecyclothorax papau* **sp. n.**, *Mecyclothorax manina* **sp. n.**, *Mecyclothorax everardi* **sp. n.**, *Mecyclothorax ramagei* **sp. n.**, *Mecyclothorax pitohitiensis* **sp. n.**, *Mecyclothorax curtisi* **sp. n.**, *Mecyclothorax hoeahiti* **sp. n.**, *Mecyclothorax ninamu* **sp. n.**, *Mecyclothorax kokone* **sp. n.**, *Mecyclothorax paahonu* **sp. n.**, *Mecyclothorax kayballae* **sp. n.**, *Mecyclothorax ehu* **sp. n.**, *Mecyclothorax papuhiti* **sp. n.**, *Mecyclothorax tuea* **sp. n.**, *Mecyclothorax taatitore* **sp. n.**, *Mecyclothorax konemata* **sp. n.**, *Mecyclothorax arboricola* **sp. n.**, *Mecyclothorax rahimata* **sp. n.**, M. *oaoa* **sp. n.**, *Mecyclothorax maninapopoti* **sp. n.**, *Mecyclothorax hunapopoti* **sp. n.**, *Mecyclothorax fefemata* **sp. n.**, *Mecyclothorax maninamata* **sp. n.**, and *Mecyclothorax niho* **sp. n.** *Mecyclothorax muriauxioides* Perrault, 1984 is **newly synonymized** with *Mecyclothorax muriauxi* Perrault, 1978. Lectotypes are designated for: *Thriscothorax altiusculus* Britton, 1938; *Thriscothorax bryobius* Britton, 1938; *Mecyclothorax globosus* Britton, 1948: and *Mecyclothorax sabulicola* Britton, 1948. Dichotomous identification keys augmented by dorsal habitus and male aedeagal photographs are provided to the various species-groups and all included species. The spermatophore of *Mecyclothorax papau* **sp. n.** is described, with the ampulla and collar found to correspond dimensionally to the length of the internal sac flagellar plate. Variation among characters of the female reproductive tract is presented for all newly described plus other representative species comprising the radiation. Taxa are assigned to species groups, modified from the classification of G.G. Perrault, based on derived character states polarized using the Australian outgroup taxon *Mecyclothorax punctipennis* (MacLeay). Much of the species-level diversity on this small Pacific island is partitioned allopatrically over very small distributional ranges. No species is shared between Tahiti Nui and Tahiti Iti, and nearly all species in Tahiti Nui are geographically restricted to one ridgelike massif of that volcano. Cladistically similar species are often distributed on different massifs suggesting that vicariance associated with erosional valley formation has facilitated speciation, however several instances in which sister species occupy sympatric distributions on the same ridge system demonstrate that speciation may also occur across extremely localized landscapes. Such localized differentiation is facilitated by the low vagility of these small-bodied, flightless predators whose fragmented populations can persist and diverge within spatially limited habitat patches. The intense philopatry of Tahitian *Mecyclothorax* spp. coupled with the highly dissected landscape has produced the geographically densest adaptive radiation on Earth. This radiation has occurred very rapidly, with species durations averaging 300,000 yr; a speciation rate similar to that observed in Hawaiian *Oliarus* planthoppers and *Laupala* crickets, and East African Rift lake cichlid fishes.

## Introduction

The area of Tahiti Island comprises 1045 km^2^, yet as is shown by this taxonomic revision, the island supports more than 100 precinctive carabid beetle species in the genus *Mecyclothorax* Sharp. How could such high levels of endemic diversity have evolved within such a restricted area? This question was asked by Georges Perrault (1992) after he taxonomically revised 67 species that occupied four separate massifs in Tahiti (Perrault 1978a, 1978b, 1984, 1986, 1987, 1988, 1989). Perrault (1992) concluded that such diversity would have resulted from speciation events brought about by fragmentation of populations in isolated montane habitats that are currently separated by ecologically inhospitable lowland valleys. He observed that closely related allopatric representatives occupied adjacent, isolated massifs (Perrault 1992, table 10.2). Thus he concluded that allopatric speciation occurring in concert with irreversible habitat fragmentation had led to the diversification of Tahitian *Mecyclothorax*.

This contribution returns to the question of Tahitian *Mecyclothorax* diversity and finds that the situation is even more complicated than Perrault was able to appreciate. All taxonomic studies are held hostage by the relative availability of field-collected specimens, and in this instance, the accrual of taxonomic material from a recent biological survey has resulted in the discovery of numerous undescribed species, in more than several instances from localities never before visited by entomologists. The addition of this material has also shown that closely related species may occur in habitats that do not appear particularly isolated, one from the other. Localities on the same ridge system but separated by several hundred meters of elevation are shown to house different, closely related species. Thus the landscape of speciation for Tahitian *Mecyclothorax* is shown here to be much more localized than previously thought. This suggests that we are still in the early stages in the development of geographically and ecologically comprehensive samples of field-based specimens that will allow a full appreciation of the biodiversity of these beetles in Tahiti.

This contribution first and foremost documents the currently known diversity of *Mecyclothorax* species occupying Tahiti. Identification keys, descriptions, and photographic illustrations support the identification function. However, given that only slightly more than half of the isolated massifs of Tahiti have been visited by entomologists, assuredly many more species remain to be discovered. Some still-to-be discovered species will be closely related to various species treated in this revision. Knowledge gained to date regarding species’ morphological attributes and ecological preferences will prove predictive regarding attributes and preferences of the future new discoveries. Therefore, this contribution celebrates what we currently know at the same time that it aims to optimize the efficient search of new locales for further undescribed *Mecyclothorax* species. It is in the spirit of encouraging successful and productive fieldwork in Tahiti that this taxonomic revision is presented.

Tahiti has recently seen the adventive introduction of various alien pest species, among these the little fire ant, *Wasmannia auropunctata* (Roger) (Hymenoptera: Formicidae) (I.P.P.C. 2005). The known range in Tahiti of this voracious predator (Loope and Krushelnycky 2007) at least borders, and may very well have overrun, the ranges of several native *Mecyclothorax* species. At this point we do not have data adequate to determine whether or not this has occurred. This work allows the identification of the most diverse native Tahitian beetle group (Nishida 2008), and provides current distributional data for the included taxa, thereby allowing assessment of a major component of native biodiversity at risk from alien invasives.

## Methods

### Taxonomic material

This study is based on 1,992 *Mecyclothorax* specimens, 1,388 that were studied by G.G. Perrault (Perrault 1978a et seq.) and 604 collected during recent biological surveys. Holotype specimens of all newly described species are deposited in the Muséum National d’Histoire Naturelle, Paris MNHN and incorporated into the Georges Perrault collection of Tahitian Carabidae. Consistent with Perrault’s type series, and in support of a more comprehensive representation of *Mecyclothorax* morphological characters within the Perrault collection, an allotypic paratype, when available, is deposited with the holotype. Other institutional depositories include: Bernice Pauahi Bishop Museum, Honolulu BPBM; Cornell University Insect Collection CUIC; Essig Museum of Entomology Collection, University of California, Berkeley EMEC; Field Museum of Natural History, Chicago FMNH; Natural History Museum, Basel MHNB; U.S. National Museum of Natural History, Smithsonian Institution, Washington, DC NMNH. Perrault’s collection holds all holotypes of his species, with the exception of *Mecyclothorax fosbergi* Perrault and *Mecyclothorax zimmermani* Perrault described from Bishop Museum material. The series of Britton’s (1938) type specimens are divided between the Natural History Museum, London and Bishop Museum, with lectotypes designated using specimens deposited at the latter.

### Laboratory techniques

This paper follows directly the laboratory protocols presented in Liebherr (2009, 2011, 2012a). Those papers may be consulted for explanations of the procedures used to elucidate taxonomic characters of the various species. Given the great utility for characters of the male aedeagus to assist in species identification, one additional step to specimen preparation is suggested for future taxonomic studies of Tahitian *Mecyclothorax*, indeed any *Mecyclothorax*; i.e., manual eversion of the male aedeagus during pointing of male specimens. Male specimens can be told by the presence of two parallel rows of squamose setae on the ventral surfaces of protarsomeres 1–3, the male protarsomeres broader than those of females. Also, the apical visible abdominal ventrite of males bears a single marginal seta each side of the midline whereas that ventrite of females bears two setae each side, plus a set of 4, occasionally 5, short setae on the midline; the setae arranged as a trapezoid. To evert the male aedeagus, the specimens must be killed in ethyl acetate and held in that atmosphere for 24 hours, at which point the muscles will be relaxed, and the aedeagus may be pulled out with fine forceps or a hooked minute nadeln while the abdomen and elytra are gently squeezed with the thumb and forefinger. Such eversion will not likely be successful using alcohol-killed specimens, though the aedeagus may evert spontaneously in rare events using that killing procedure.

### Descriptive conventions

Treatments for all newly described species include an extended diagnosis, description of male and female genitalia if known, type specimen and species epithet information, and notes regarding geographical distribution, collecting method, or habitat. The extended diagnosis format includes an initial section that provides criteria to distinguish the species from all others known, followed by brief synopses of characters of the head, pronotum, elytra, microsculpture, and coloration. Holotype label data are transcribed verbatim, with individual lines of label text indicated by a slash, “/”, and separate labels indicated by a double slash; “//.” Many species epithets are derived from the Tahitian language, with meanings taken from Wahlroos (2002). All species epithets derived from Tahitian are to be treated as nouns in apposition. For all previously described species, a short identification section noting salient characters that will confirm the identification is accompanied by notes summarizing what is known of distribution and habitat. Habitat notes for all species are a compilation of those recorded by Perrault (1978a et seq.) plus data associated with more recently collected specimens.

Various ratios of length and width are used to describe shapes of the head, pronotum and elytra, necessitating the availability of a measuring reticle in the microscope ocular. For the head these include: 1, the ocular ratio, the maximum head width across the outer surface of the compound eyes (MHW) divided by the minimum width of the frons between the eyes (mFW); and 2, the ocular lobe ratio, the diameter of the eye measured from above, divided by the distance from the front margin of the eye to the juncture of the ocular lobe and gena, measured from the same vantage point. Prothoracic dimensions presented as ratios include: MPW, maximum pronotal width; BPW, basal pronotal width, measured between the hind angles; APW, apical pronotal width measured between the two most anterior points at the pronotal front angles; and PL, pronotal length measured along the midline. Elytral dimensions include MEW, or maximum elytral width, and HuW, humeral width, measured between the most anteriorly positioned points, i.e., the humeral angles. Standardized body length comprises the sum of three values: 1, the length of the head from labral anterior margin to cervical ridge, the position of the ridge estimated from its lateral reaches when hidden medially under the pronotal margin; 2, median pronotal length; and 3, the length of the longer elytron from the basal ridge of the scutellum to its apex, measured parallel to the suture.

When more than one individual was available for the calculation of ratios, the number of individuals used for ratio calculation in that particular description is provided – as “(n = X)” – after the first mention of a ratio, with X representing the number of individuals measured. For larger series, a maximum of five individuals were so measured, with the largest individual, the smallest individual, and representatives of both sexes included in the sample of five. By this method the most disparate range of ratios was sought, although the ratios are used only for descriptive purposes and are not statistically evaluated. Measured specimens bear a numerical tag with values 1–5.

### Collecting localities

**Figure 1.**
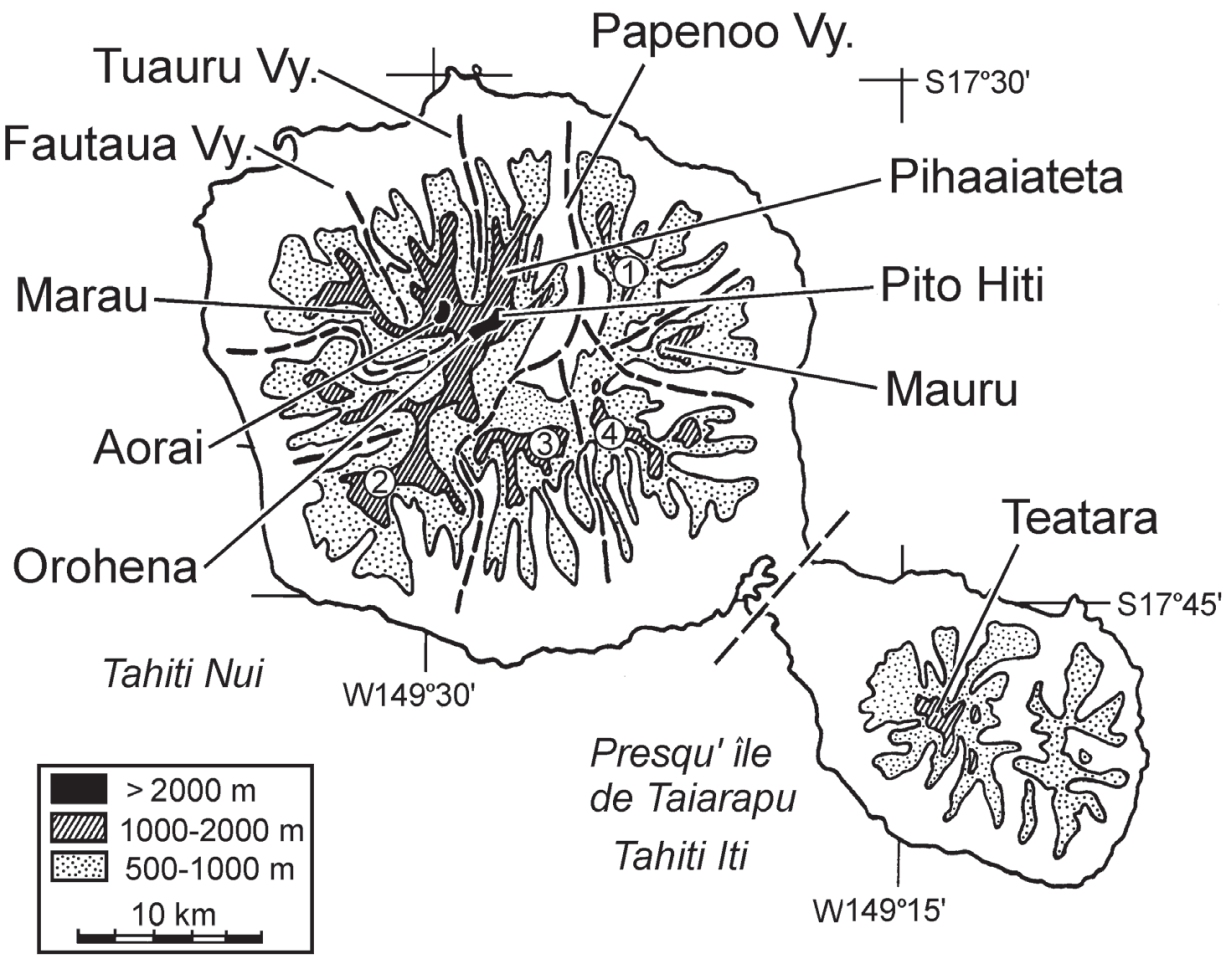
Massifs of the Tahiti Nui and Tahiti Iti volcanoes from which *Mecyclothorax* specimens have been collected (redrawn and amended from Perrault 1992). Dashed lines indicate lowland valleys and associated headlands below 1000 m elevation that separate the major massifs. Spellings and names for localities conform to I.G.N. (1994). The summit in Tahiti Iti that has been sampled for *Mecyclothorax* has been variously named Mont Atara (Perrault 1989, 1992), or Teatara (I.G.N. 1994; R.G.P.F. 2001), the latter official name used herein. Massifs not yet sampled for *Mecyclothorax* but certainly housing species of the genus include Mont Aramaoro (1), Monts Ivirairai plus Iviroa (2), Mont Tetufera (3), and Mont Urufa (4).

Perrault (1992) and colleagues gained access to four major massifs in support of his major taxonomic revision of the Tahitian *Mecyclothorax*. He was able to make extensive collections on Monts Marau, and Aorai in Tahiti Nui, and Teatara in Tahiti Iti (Fig. 1), as well as more limited collections at the lower elevations of the ridge he termed Pihaaiateta (Perrault 1992, table 10.2). More recent survey activites have expanded geographic coverage of Tahitian *Mecyclothorax* populations to include Mont Mauru (Liebherr 2012b) and Pito Hiti; a summit above Pihaaiateta on the ridge that culminates at Mont Orohena (Fig. 1). Mont Tohiea, Moorea also houses *Mecyclothorax* species (Liebherr 2012a), expanding the generic distribution to a second Society Island. Nevertheless, the geographic coverage of biological surveys for Tahitian *Mecyclothorax* as well as other insects and arthropods remains significantly incomplete, with Mont Aramaoro in the northeast quadrant of Tahiti Nui, and the distinct massif systems associated with Monts Ivirairai, Tetufera, and Urufa across the southern reaches of Tahiti Nui completely unexplored (Fig. 1). Given the local endemicity of the Tahitian *Mecyclothorax* biota already discovered, many species occupying these massifs remain to be described.

## Taxonomic treatment

### Lectotype designation

Britton (1938) described the first four *Mecyclothorax* spp. from Tahiti, placing them in *Thriscothorax* Sharp, 1903, a genus proposed by Sharp (1903) for Hawaiian species with a single lateral seta each side of the pronotum. In his subsequent treatment of the Hawaiian fauna, Britton (1948) realized that Sharp’s various 1903 genera, based only on pronotal setation, were artificial. Indeed, Sharp himself (1903) commented, “The problem as to whether *Mecyclothorax robustus* and *Thriscothorax robustus* (differing almost solely by their setae) may be really only one species is not the least interesting of the questions raised by my slight study of Hawaiian Carabidae, and as the insects are apparently not rare on Haleakala it may be possible to decide it by observation of the forms in their haunts (p. 185).” Britton (1948) therefore placed all Hawaiian species described in Sharp’s various 1903 genera under *Mecyclothorax* Sharp, similarly recombining his four Tahitian species. This change caused two of Britton’s 1938 Tahitian species names to become junior homonyms. Thus he proposed *Mecyclothorax globosus* Britton, 1948, and *Mecyclothorax sabulicola* Britton, 1948 as replacement names.

Sharp’s four species were described with the type data limited to a statement of the numbers of specimens collected for each, along with ecological data and recording that E.C. Zimmerman was the collector. Following British Museum (Natural History) convention, he placed a round, red-bordered “Type” label on one of the specimens, and round yellow-bordered “Paratype” labels on the remaining members of the type series. The series were divided, with the “Type” and various numbers of “Paratypes” returned to Bishop Museum, where they were accessioned as holotypes and paratypes. However as no single specimen was designated as holotype in the description, all specimens of the type series for each species are syntypes. Given that Britton’s type series were divided between London and Honolulu, and given the subtle differences between the various Tahitian species, lectotypes are designated below to stabilize the nomenclature of these species. In all cases, the specimen bearing the round red-bordered “Type” label is chosen as the lectotype. The remaining syntypes become paralectotypes, and those in the Bishop Museum are so labeled.

*Thriscothorax altiusculus* Britton, 1938: lectotype female hereby designated. Label data include: round red-bordered Type label // Tahiti I. / 5500–6300 ft. / IX-15-34 // Mt. Aorai / Trail // Society / Islands // Freycinetia // ECZimmerman / collector // TYPE / Thriscothorax / altiusculus sp. n. / E.B.Britton. // Holotype / No. 981 / Thriscothorax / altiusculus / Britton // Lectotype ♀ / Thriscothorax / altiusculus / Britton / des. J.K. Liebherr 2013 (black-bordered red label). Two paralectotypes are also labeled.

*Thriscothorax bryobius* Britton, 1938: lectotype male hereby designated. Label data include: round red-bordered Type label // Tahiti I. / 5500–6300 ft. / IX-15-34 // Mt. Aorai / Trail // Society / Islands // beating moss / on trees and / shrubs // ECZimmerman / collector // TYPE / Thriscothorax / bryobius sp. n. / E.B.Britton. // Male aedeagus mounted in Canada balsam between two round cover slips and mounted to pin by glued card // Holotype / No. 984 / Thriscothorax / bryobius / Britton // Lectotype ♂ / Thriscothorax / bryobius / Britton / des. J.K. Liebherr 2013 (black-bordered red label). One paralectotype is also labeled.

*Mecyclothorax globosus* Britton, 1948 (= *Thriscothorax constrictus* Britton, 1938, junior secondary homonym): lectotype female hereby designated. Label data include: round red-bordered Type label // Tahiti I. / IX-13-34 / 3500–4500 ft. // Mt. Aorai / Trail // Society / Islands // beating shrubs // ECZimmerman / collector // TYPE / Thriscothorax / constrictus sp. n. / E.B.Britton. // Holotype / No. 982 / Thriscothorax / constrictus / Britton // Lectotype ♀ / Mecyclothorax / globosus / Britton / des. J.K. Liebherr 2013 (black-bordered red label).

*Mecyclothorax sabulicola* Britton, 1948 (= *Thriscothorax minutus* Britton, 1938, junior secondary homonym): lectotype male hereby designated. Label data include: round red-bordered Type label // Tahiti I. / Taohiri, 3500 ft. / IX-13-34 // Mt. Aorai / Trail // Society / Islands // edge of pond / on sand // ECZimmerman / collector // TYPE / Thriscothorax / minutus sp. n. / E.B.Britton. // Holotype / No. 983 / Thriscothorax / minutus / Britton // Lectotype ♂ / Mecyclothorax / sabulicola / Britton / des. J.K. Liebherr 2013 (black-bordered red label). One paralectotype is also labeled.

### Character systems

This contribution aims to provide support for unambiguous identification of the *Mecyclothorax* species currently known to reside in Tahiti. Even given the great diversity of species, this activity can be undertaken with confidence given an understanding of several very important systems of anatomical characters. Based on examination of multiple specimens derived from many collecting events, the reliability of particular characters can be evaluated. Thus, even though many of the species are known from single specimens, the configuration of particular characters for as yet uncollected specimens can be predicted, with those predictions tested by anybody using this reference. External anatomical and internal genitalic characters were extensively recorded for specimens representing all newly described species, and findings for four of these classes of characters are summarized below. In addition, traits defined by shape and color are used extensively in the key and descriptions, with dorsal habitus photographs allowing the user to directly assess what the descriptive language attempts to convey. All Tahitian taxa are characterized by reduced metathoracic flight wings however the degree of reduction of the wings varies among the Tahitian species. Where viewable on a specimen from which the abdomen had been removed in the course of genitalic dissection, the configuration of these rudiments is reported, including wing vein homologies based on the system proposed by Kukalová-Peck and Lawrence (1993).

**Figure 2.**
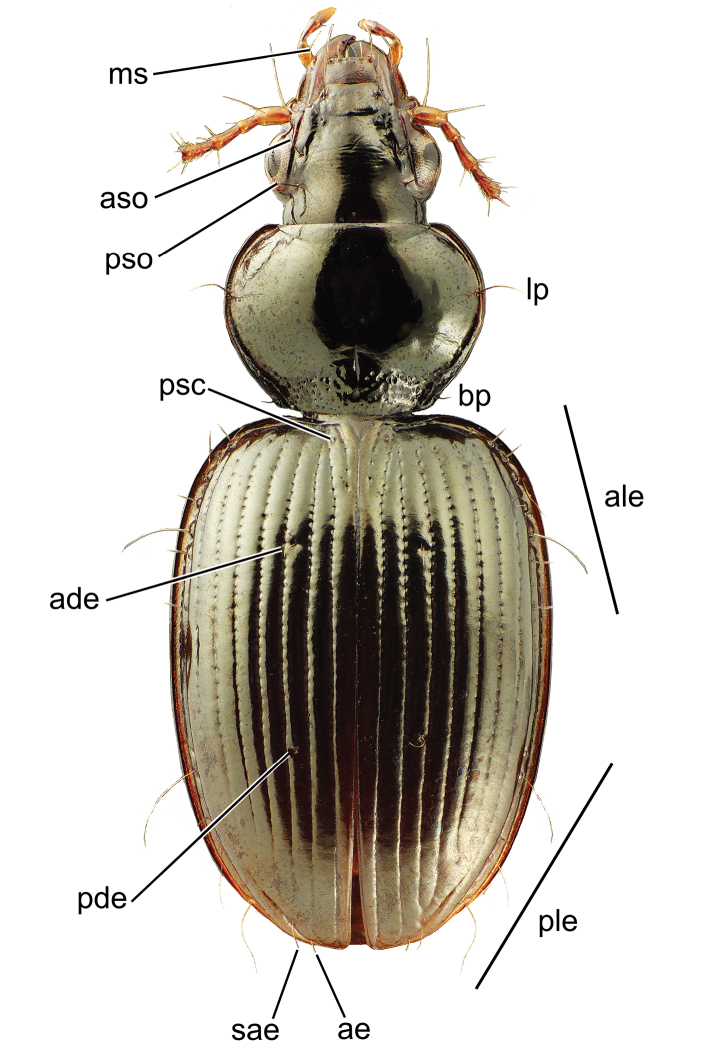
Head, pronotum, and elytra of *Mecyclothorax wallisi* with setae labeled: head, **ms** mandibular scrobe seta **aso** anterior supraorbital seta **pso** posterior supraorbital seta; pronotum **lp** lateral pronotal seta **bp** basal pronotal seta; elytra **ade** anterior dorsal elytral seta **pde** posterior dorsal elytral seta **ale** anterior series of lateral elytral setae **ple** posterior series of lateral elytral setae **psc** parascutallar seta **sae** subapical elytral seta **ae** apical elytral seta.

Setation. Carabid beetles exhibit macrosetae at specific positions on the external surface of the body, with the presence and number of seta extremely useful for diagnosing taxa. All *Mecyclothorax* spp., being member taxa of the tribe Moriomorphini – one of the tribes comprising the Jeannel’s informal grouping, Stylifera – possess a seta in the mandibular scrobe (Fig. 2). Carabid beetles exhibit setae above the eyes and laterad the terminal reaches of the frontal grooves. In Tahitian *Mecyclothorax*, the more generalized condition involves presence of two supraorbital setae, the anterior dorsad the anteriodorsal margin of the eye, and the posterior dorsad the hind margin of the eye (Fig. 2). In several species of the *Mecyclothorax muriauxi* and *Mecyclothorax globosus* species groups, the anterior supraorbital seta is absent whereas the posterior seta is present (e.g., *Mecyclothorax taiarapu* Perrault, Fig. 39A). This character varies among individuals of *Mecyclothorax profondestriatus* Perrault, in which two of the four specimens unilaterally possess the anterior seta (Perrault 1989).

In the generalized condition the *Mecyclothorax* pronotum bears two setae along each lateral margin; the lateral pronotal seta and the basal pronotal seta (Fig. 2). The basal seta is absent from individuals of many Tahitian *Mecyclothorax*; e.g., *Mecyclothorax gerardi* Perrault and *Mecyclothorax jeanyvesi* sp. n. (Figs 11B, C). The broadly based absence of the basal pronotal seta is therefore a significant discrimator in the diagnoses of several species groups.

The elytra bear setae at standardized positions across Tahitian *Mecyclothorax*. All Tahitian species exhibit the parascutellar seta positioned near the base of the elytron immediately laterad the parascutellar striole (Fig. 2). Among described Tahitian species there are maximally two dorsal elytral setae; the setae situated in the third elytral interval, usually near the anterior quarter or third of the elytral length, and from near the midpoint to apical 2/3 of the length (Fig. 2). A third dorsal elytral seta, set between the anterior and posterior seta, is present unilaterally in the lone known specimen of *Mecyclothorax pahere* Liebherr from Moorea (Liebherr 2012a). One or both of the anterior or posterior setae may be absent in various taxa, but if only one seta is present it is homologous with the anterior seta, as single setae are always found in the anterior half of the elytral length (e.g., *Mecyclothorax fosbergi*, Fig. 15A). One or two setae may be present near each elytral apex; the apical seta near the juncture of the second elytral stria and the elytral margin, and the subapical seta positioned in the apex of elytral stria 7 posterad the fusion of elytral striae 3 and 4 (Fig. 2). In many species, only one seta, clearly the apical seta, occurs near the elytral apex. In this configuration the lone seta is present posterad the terminal apex of stria 2. However in several species – *Mecyclothorax tahitiensis* Perrault and *Mecyclothorax pitohitiensis* sp. n. (Fig. 23C) – a single apical seta is positioned more laterally along stria 7 apicad the fused terminus of striae 3+4. That this more laterally positioned seta also represents the apical seta can be affirmed by setal positions in the closely related *Mecyclothorax fairmairei* Perrault. In this species the subapical setae is unilaterally present and absent. On the side bearing both apical and subapical seta, the apical seta is also located in a lateral position posterad the fused apex of striae 3+4. The subapical seta is situated even further to the side, laterad the fused apex of striae 5+6, and near the mesal margin of stria 7. Thus in all instances where Tahitian *Mecyclothorax* exhibit only one seta near the apex of each elytron, it is the apical seta.

Elytral setation is completed by two series of lateral setae situated in the eighth elytral stria. The anterior series of lateral elytral setae consists of six to seven setae, more commonly seven, with specimens of several species exhibiting bilateral variation from six to seven setae. When seven setae are present, the third and fifth from the front are longest, with the anterior seta nearly as long. The posterior series of lateral elytral setae ranges from four to six setae, most commonly six. As in the anterior series, individuals may vary bilaterally by one or occasionally two setal counts. When six setae are present the second, fourth and sixth setae in the series are longer.

This contribution follows Perrault in presenting a setal formula (e.g., Fig. 10) as shorthand for the setal configuration observed in individuals of a species. The formula WXYZ denotes the number of supraorbital setae (W; either 1 or 2), lateral pronotal setae (X; either 1 or 2), dorsal elytral setae (Y; 0, 1, or 2), and apical plus subapical elytral setae (Z; either 1 or 2). If the setal count varies for any comparison, the range of the setal counts is shown parenthetically.

Microsculpture. The nature of reticulations in the cuticular surfaces is extremely useful for discriminating otherwise extremely similar species. This study follows the general terminology established by Lindroth (1974), focusing on the shapes of individual sculpticells as well as the overall spatial relationship of adjacent reticulations defining the borders of those sculpticells. Four general configurations of microsculpture are aligned along a gradient of shape: 1, isodiametric microsculpture wherein individual sculpticells are of equal breadth and length, and are arranged as shingled hexagons; 2, transverse-mesh microsculpture in which the sculpticells are broader than long, and arranged in transverse rows as rectangular tiles defining a mesh-like pattern; 3, transverse-line microsculpture,where sculpticells are not well defined laterally resulting in a grate-like pattern defined by laterally directed, parallel grooves; and 4, extremely reduced microsculpture wherein the cuticular surface is glossy, and sculpticells are impossible to discern. Transverse microsculpture is associated with iridescent reflection from the cuticular surface, with the spacing of sculpticell borders and parallel gratings defining different reflected colors; from silver to green to blue. For comparison of specimens, the anatomical position of the microsculptural comparison must be delimited – e.g., pronotal disc, pronotal median base, laterobasal pronotal depression – as sculpticell shape changes across the cuticular surface of various somites and structures. Little variation in microsculpture was observed between specimens of different sexes, when those were available for comparison, though for recording of microsculpture for descriptive and identification purposes, male specimens were used when available.

Male genitalia. The aedeagal median lobe and internal sac, as well as the associated parameres, exhibit substantial variation across the species of Tahitian *Mecyclothorax*. Perrault (1978a et seq.) used median lobe conformation, especially the shape of the lobe apex, to great success in sorting and describing his substantial collections. Where a male specimen is available, aedeagal characters in concert with external anatomical characters provide certain species identification.

Aedeagal orientation is complicated by torsion of the male intromittent organ during the evolutionary history of adephagan Coleoptera. Plesiomorphically the aedeagal structures – median lobe and parameres – are symmetrical, and the parameres articulate with the dorsal surface of the median lobe (Sharp and Muir 1912). In this condition the aedeagus retains the same orientation in repose and when everted. In most Carabidae, the aedeagus has rotated 90° to the right, thereby lying on its right side in repose (Deuve 1993). When in use, the aedeagus turns another 90° so that its anatomically dorsal face, bearing the parameres, become ventral until the intromittent organ is brought about to face anteriorly so that it can enter the female, at which point the anatomical dorsal surface is once again dorsally oriented. Evidence of this 180° torsion is retained in the positions of the tracheae servicing the median lobe, as they are crossed when the lobe rotates to its position of use (Jeannel 1941; Deuve 1993). For this study, the aedeagal orientation is considered to represent the point of eversion, whereby the parameres are oriented ventrally. This is the position of the aedeagus when it is manually everted during specimen preparation, and thus the orientation of the illustrations herein provides the easiest form of comparison with field-collected specimens.

**Figure 3.**
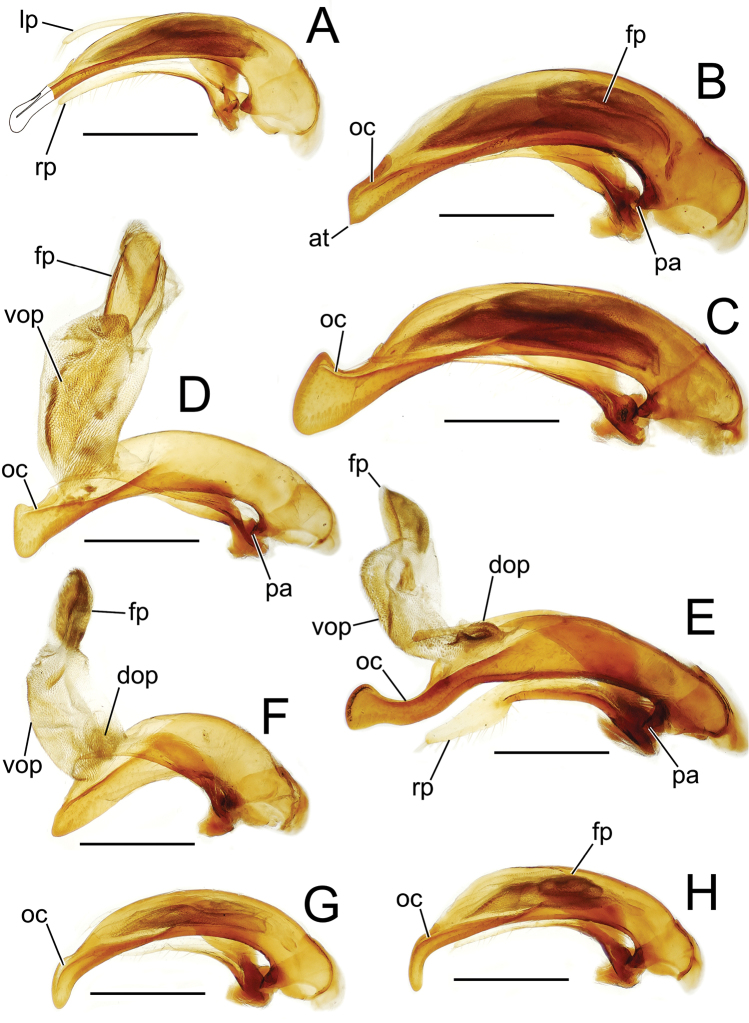
Male aedeagal median lobe and associated parameres, *Mecyclothorax* spp., right lateral view; scale bars 0.5 mm **A** *Mecyclothorax striatopunctatus* paratype (MNHN) (apex of dissection damaged and lost; apex depicted by detail drawing [Perrault 1986, fig. 23]) **B** *Mecyclothorax bougainvillei* holotype **C** *Mecyclothorax mahina* holotype **D** *Mecyclothorax poria* holotype **E** *Mecyclothorax villiersi* **F** *Mecyclothorax taatitore* paratype (CUIC) **G** *Mecyclothorax pirihao* paratype (CUIC) **H** *Mecyclothorax bryobioides* Abbreviations: **at** apical tooth **dop** dorsal ostial microtrichial patch **fp** flagellar plate **lp** left paramere **oc** ostial canal **pa** parameral articulation **rp** right paramere **vop** ventral ostial microtrichial patch.

The more generalized configuration of the median lobe entails an apex that is narrowly rounded (Fig. 3A). This form is also observed in the median lobe of the putative adelphotaxon to both the Tahitian and Hawaiian *Mecyclothorax* radiations; the Australian *Mecyclothorax punctipennis* (MacLeay) (Britton 1948; Liebherr 2012a, fig. 8A). Among Tahitian *Mecyclothorax*, the median lobe can assume a broader dorsoventral profile (Figs 3B, D), or a dorsal hooklike projection (Fig. 3C). The lobe shaft apicad the ostial opening may be prolonged and curved, with a variously expanded tip (e.g., the closely related *Mecyclothorax villiersi* Perrault and *Mecyclothorax pitohitiensis*, Figs 3E, 24F). There is a variously formed depression that extends from the apex of the ostial opening – the ostial canal (Fig. 3) – that may be straight (Figs 3B, D), curved dorsally (Fig. 3C), or curved ventrally (Fig. 3G). Among several species of the *Mecyclothorax globosus* group, the apex is foreshortened (Figs 3F, 47E, H); in one instance in combination with with a spinose dorsal projection (Fig. 47G). The apex may also be distinctly curved downward, the curved portion directed downward at an angle from the shaft axis (Figs 3G, H).

The parameres associated with the median lobe lie along the lobe’s ventral surface, and articulate at an apodeme situated at the juncture of the basal bulb and the narrower lobe shaft (Fig. 3). The right paramere is slightly broadened basally and projected narrowly to a narrow apex (Figs 13D, E), and its ventral margin is lined with setae. In repose, due to the 90° torsion exhibited by the median lobe during eversion, the right paramere lies ventrad the median lobe. The left paramere is much broader basally than the right, and extends as a whiplike projection that bears 3 setae at it apex. Both parameres are narrow apically in nearly all species of *Mecyclothorax* with the exception of several species in the *Mecyclothorax dannieae* group, in which the apex of the right paramere is expanded into a setose, spatulate lamella (Figs 3E, 24E, F, 27D, E). Nothing is known regarding the function of these lamellar expansions during mating, but given that the parameres remain outside the female vagina and bursa copulatrix, they may have a sensory function associated with the gonocoxae or associated membranes.

The median lobe internal sac in Tahitian *Mecyclothorax* is a tubular eversible structure bearing a sclerotized, apical flagellar plate (Figs 3D–F). The flagellar plate is concave, with a smooth ventral surface that may bear melanized longitudinal carinae (Figs 13F, 18C). Dorsad the flagellar plate there is an extensile membranous surface that medially bears the gonopore (Fig. 18C). The sperm duct lies between the gonopore membrane and the inner surface of the flagellar plate. In some dissection preparations, the membrane lies quite loose dorsad the flagellar plate, and in others it appears tightly appressed to the plate; the difference perhaps based on the history of reproductive activity of the male individual. For comparative purposes taking into account the dimensions of the median lobe, flagellar plate length is assessed by comparing its length to the distance from the articulation of the parameres (e.g., Figs 3C–E) to the most distant point on the apex of the median lobe. There are two primary fields of microspicules on the surface of the internal sac. Most generally, the ventral surface of the sac is covered with a broad field of uniformly sized microtrichia. In concordance with Maddison’s (1993) terminology for the male internal sacs of *Bembidion* beetles, this is termed the ventral ostial microtrichial patch (Figs 3D–F). A second field of stouter microtrichia may occur on the dorsal surface of the sac immediately adjacent to the ostial opening; the dorsal ostial microtrichial patch (Fig. 3E). The spicules comprising this patch are stout, long and acuminate in males of three species of the *Mecyclothorax globosus* group (Figs 47E, G, H).

**Figure 4.**
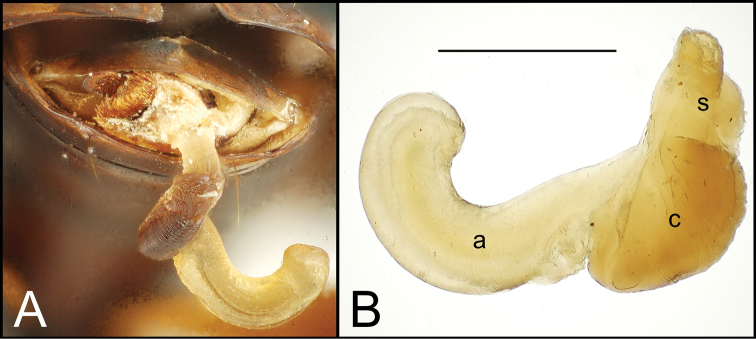
Spermatophore from male specimen of *Mecyclothorax papau* **A** spermatophore extended from ostium of uneverted male aedeagus; spicules to left of spermatophore stem form ventral ostial microtrichial patch (Fig. 24A) B spermatophore, *Mecyclothorax papau*, after extraction from male aedeagal median lobe ostial opening; scale bar 0.5 mm Abreviations: **a** ampulla **c** collar **s** stem.

Alcohol preservation of the male specimen of *Mecyclothorax papau* fortuitously resulted in eversion of a spermathophore from the partially everted sac of the aedeagal median lobe (Fig. 4A). The spermatophore consists of a curved apical ampulla, presumably holding the sperm, a broader, more melanized and laterally striated collar, and a basal stem (Fig. 4B). Jeannel (1941: 41) described a similar spermatophore for *Orotrechus stephani* Müller, calling the putative sperm-holding ampulla the head. Jeannel reported that the collar and head of the spermatophore was the same length as the copulatory piece of the *Orotrechus stephani* male internal sac. In the present instance, the hydrated spermatophore of *Mecyclothorax papau* has curved ampulla or head that extends ~0.75 mm beyond the collar (Fig. 4B). Similarly, the flagellar plate of the *Mecyclothorax papau* internal sac is also ~0.75 mm long (Fig. 24A), confirming Jeannel’s finding of a dimensional fit between a sac sclerite and the spermatophore. Given that the flagellar plate is concave ventrally, with the gonopore in the middle of the membranous, convex dorsal surface, it would appear the spermatophore apex is extruded from the gonopore, with the collar forming in association with the gonopore, and the ampullar head assuming the conformation of the internal sac lumen as held within the median lobe. During mating the flagellar plate conveys the spermatophore into the female bursa copulatrix, with the spermatophore stem presumably the last portion of the spermatophore to exit the gonopore during the mating process.

**Figure 5.**
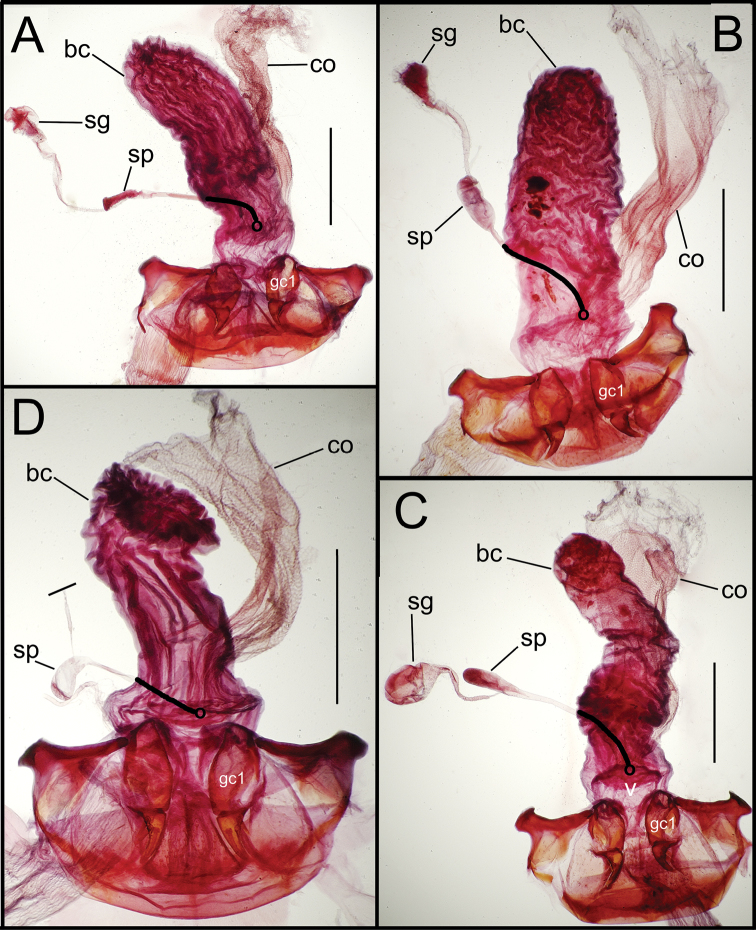
Female reproductive tract dissections, *Mecyclothorax* spp., ventral view; scale bars 0.5 mm **A** *Mecyclothorax wallisi* **B** *Mecyclothorax altiusculus* **C** *Mecyclothorax altiusculoides* **D** *Mecyclothorax marau*; spermathecal gland reservoir broken off of dissection Abbreviations: **bc** bursa copulatrix **co** common oviduct **gc1** basal gonocoxite 1 **sg** spermathecal gland **sp** spermatheca **v** vagina. Position of spermathecal duct and juncture of duct with dorsal wall of bursa indicated by black line and terminal circle, respectively.

**Figure 6.**
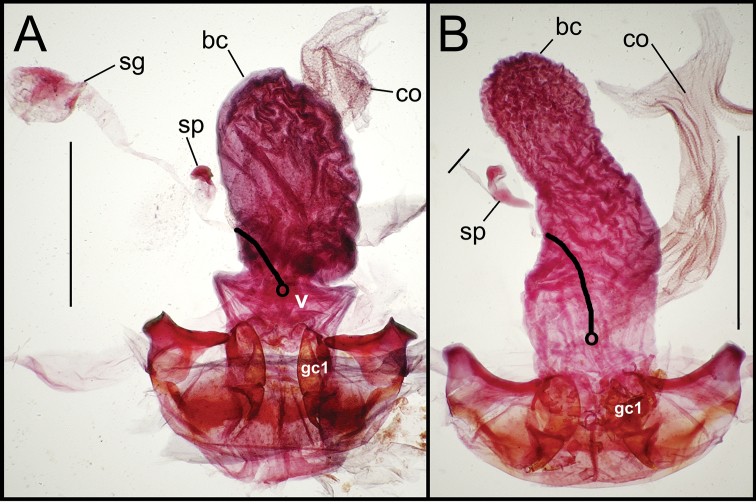
Female reproductive tract dissections, *Mecyclothorax* spp., ventral view; scale bars 0.5 mm **A** *Mecyclothorax kayballae* **B** *Mecyclothorax gourvesi*; spermathecal gland reservoir broken off of dissection Abbreviations as in Fig. 5. Position of spermathecal duct and juncture of duct with dorsal wall of bursa indicated by black line and terminal circle respectively.

**Figure 7.**
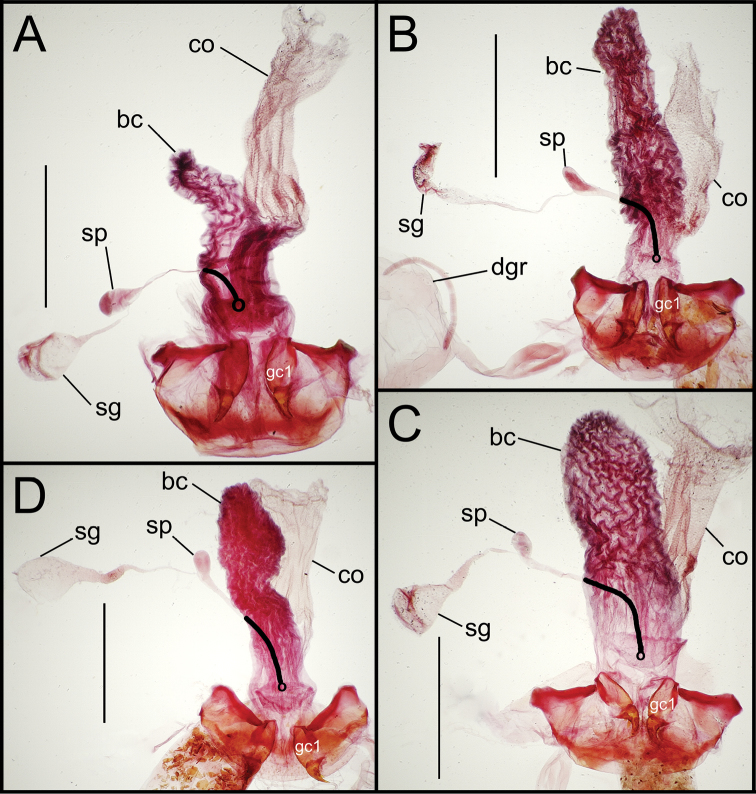
Female reproductive tract dissections, *Mecyclothorax* spp., ventral view; scale bars 0.5 mm **A** *Mecyclothorax arboricola* paratype (CUIC) **B** *Mecyclothorax globosoides* **C** *Mecyclothorax globosus* **D** *Mecyclothorax paraglobosus* Abbreviations as in Fig. 5; **dgr** defensive gland reservoir. Position of spermathecal duct and juncture of duct with dorsal wall of bursa indicated by black line and terminal circle respectively.

Female reproductive tract. Although we lack sufficient taxonomic coverage to diagnose all closely related species using female reproductive tract characters, a survey of the female reproductive tract across the Tahitian *Mecyclothorax* radiation serves to establish the levels of variation within this character system. The association of male and female reproductive structures also allows a discussion of how these structures may be evolutionarily associated during diversification. The carabid beetle female reproductive tract includes a sclerotized pair of gonocoxae derived from coxal appendages of abdominal segment IX (Deuve 1993). The female bursa copulatrix comprises a membranous invagination basad the gonocoxae, and represents abdominal segment X (Iuga and Roşca 1966). The common oviduct and various spermathecal structures and glands enter the bursa copulatrix (Liebherr and Will 1998). In female *Mecyclothorax* beetles, the common oviduct enters the bursal region near the bases of the gonocoxae, an area sometimes distinctly definable as the vagina (Figs 5C, 6A). More commonly, the membranous bursa extends inwardly from the gonocoxae with readily identified subregions (Figs 5A, B, 6B). The bursa copulatrix may be of various lengths relative to its breadth, and the convoluted surface suggests its ability to distend greatly during mating or fertilization of eggs and oviposition, or both (Figs 6, 7). Among externally similar species of the *Mecyclothorax globosus* group, the bursa may be narrowed apically (Fig. 7A), about 3× long as broad (Figs 7A, C), to columnar and elongated, with its length 4–5× breadth (Figs 7B, D). To the degree that females are available for dissection, these dimensions appear to reflect species-level differences.

Dorsal to the bursa copulatrix lies a spermatheca apically situated on a spermathecal duct, the latter entering the dorsal surface of the bursa copulatrix immediately dorsad the ventral juncture of the common oviduct and bursa. The spermatheca bears an appended spermathecal gland, the duct of which enters near the base of the fusiform spermatheca (Fig. 5C).

**Figure 8.**
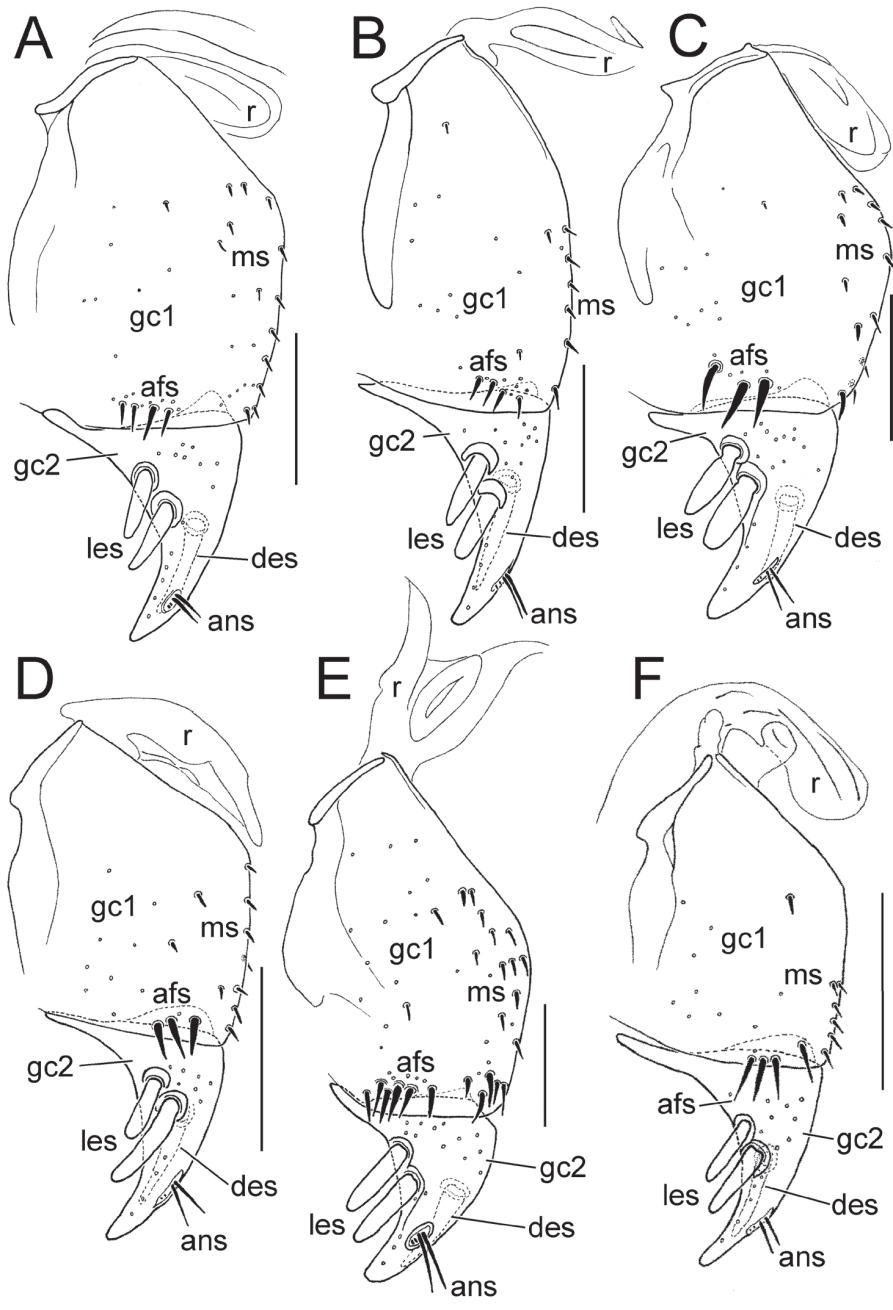
Female right gonocoxae, *Mecyclothorax* spp., ventral view, scale bars 0.1 mm **A** *Mecyclothorax wallisi* **B** *Mecyclothorax poria* **C** *Mecyclothorax altiusculus* **D** *Mecyclothorax everardi* paratype (CUIC) **E** *Mecyclothorax pitohitiensis* paratype (CUIC) **F** *Mecyclothorax hoeahiti* paratype (CUIC) Abbreviations: **afs** apical fringe setae **ans** apical nematiform setae **des** dorsal ensiform seta **gc1** basal gonocoxite 1 **gc2** apical gonocoxite 2 **les** lateral ensiform setae **ms** mesal setae of gonocoxite 1 **r** ramus

**Figure 9.**
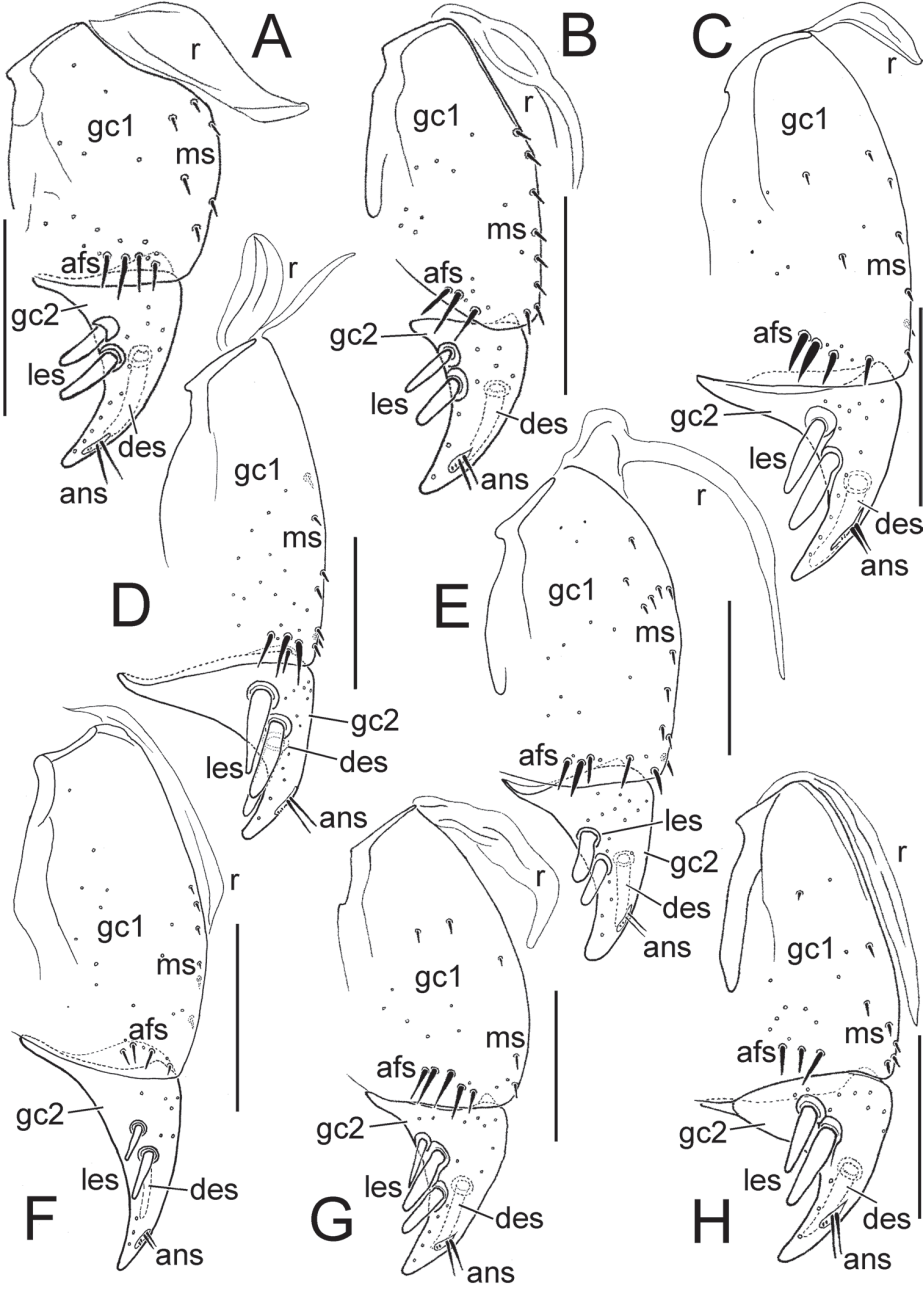
Female right gonocoxae, *Mecyclothorax* spp., ventral view, scale bars 0.1 mm **A** *Mecyclothorax ninamu* paratype (CUIC) **B** *Mecyclothorax kokone* paratype (CUIC) **C** *Mecyclothorax kayballae* paratype (CUIC) **D** *Mecyclothorax ehu* paratype (CUIC) **E** *Mecyclothorax taatitore* paratype (CUIC) **F** *Mecyclothorax arboricola* paratype (CUIC) **G** *Mecyclothorax fefemata* paratype (CUIC) **H** *Mecyclothorax maninamata* holotype. Abbreviations as in Fig. 8.

The female gonocoxae are bipartite, with a broader less sclerotized basal gonocoxite 1, and a more heavily sclerotized, triangular apical gonocoxite 2 (Figs 8, 9). Gonocoxite configuration differs across the *Mecyclothorax* radiation principally in levels of setation on gonocoxite 1, and in shape of gonocoxite 2. The externally generalized species of the *Mecyclothorax striatopunctatus* group (Liebherr 2012a) – e.g., *Mecyclothorax wallisi* (Fig. 8A) – exhibit a gonocoxite 1 with an apical fringe of setae laterad the sagittal midline of gonocoxite 2, and a broadly triangular gonocoxite 2 bearing two lateral ensiform setae and one dorsal ensiform seta. As in all *Mecyclothorax* and contribal Moriomorphini, gonocoxite 1 is bordered mediobasally by a ramus; in *Mecyclothorax* an unsclerotized remnant of a gonocoxal sclerite found mesad the base of the gonocoxae in tribes such as Carabini, Cicindelini, Scaritini, Broscini and Patrobini, among others (Liebherr and Will 1998).

An increased level of setation on gonocoxite 1 is observable in *Mecyclothorax pitohitiensis* (Fig. 8E). The additional setation includes elevated numbers of mesal setae, a patch of setae near the mesoapical margin of the coxite, and an apical fringe of 5–6 setae. Smaller body size is associated somewhat with reduced setation of gonocoxite 1, as observed in several small-bodied species of the *Mecyclothorax globosus* group; e.g., *Mecyclothorax fefemata* sp. n. and *Mecyclothorax maninamata* sp. n. (Figs 9G, H). Nevertheless the smaller bodied *Mecyclothorax kokone* sp. n. of the *Mecyclothorax viridis* group displays a broadly distributed mesal field of 6–8 setae.

The shape of gonocoxite 2 varies among species, with the base broadly extended laterally in many species (Fig. 8) versus narrow and little extended in the small-bodied *Mecyclothorax kokone* (Fig. 9B). As the large lateral apodeme at the base of gonocoxite 2 serves as the basis for gonocoxal levator muscles M29 and M30 (Bils 1976) that serve to pull the coxites upward and outward during oviposition activities, the gonocoxal basal width may be associable with a particular breeding substrate and egg-laying behavior.

In nearly all *Mecyclothorax*, the apical gonocoxite bears two large lateral ensiform setae (terminology from Ball and Shpeley 1983). Only in one individual of *Mecyclothorax fefemata* was a third, smaller ensiform seta unilaterally present on the right gonocoxa (Fig. 9G). Yet the position of the lateral ensiform setae on the ventral surface of the apical gonocoxite varies among the species. The lateral ensiform setae are most commonly situated near the ventrolateral margin of the gonocoxite, with their articulatory sockets reaching the lateral cutting margin (Figs 8, 9A–C, E, G). In the *Mecyclothorax globosus* group species *Mecyclothorax ehu* sp. n. and *Mecyclothorax maninamata*, the ensiform setae are more distant from the lateral margin and closer to the basal margin of the coxite (Figs 9D, H). In female *Mecyclothorax arboricola* sp. n., the ensiform setae are positioned more ventrally and extend little toward the lateral margin (Fig. 9F). The apical gonocoxite also bears a single ensiform seta on its dorsal surface, and an apical sensory furrow that bears a pair of apical nematiform setae, plus two furrow pegs near the apical end of the furrow.

### Identification key to the species groups of *Mecyclothorax* Sharp from the Society Islands

This key to groups is based on Perrault’s (1986) species group classification, with several changes to group recognition and group membership. First, given the anatomically isolated nature of the two largest Tahitian *Mecyclothorax*, *Mecyclothorax fosbergi* and *Mecyclothorax fosbergioides* Perrault, these species are removed from the *Mecyclothorax altiusculus* group to a distinct *Mecyclothorax fosbergi* group. Secondly, given that: 1, setational characters for *Mecyclothorax zimmermani* very poorly fit Perrault’s diagnosis of the *Mecyclothorax muriauxi* group (Perrault 1984: 22); and 2, *Mecyclothorax zimmermani* is characterized by expanded pronotal marginal depressions, observed in members of the *Mecyclothorax gourvesi* group, *Mecyclothorax zimmermani* is removed to that species group where it is shown to share setational and male aedeagal characters. Similarly, *Mecyclothorax acutangulus* Perrault is removed from the *Mecyclothorax altiusculus* group and transferred to the *Mecyclothorax gourvesi* group, as it also exhibits the broad, sinuate pronotal margins and an aedeagus characteristic of other members of the group. Finally, one newly described species, *Mecyclothorax tuea* sp. n., collected on Pito Hiti by Dr. Elin Claridge, is judged to be so distinct from all other species known from Tahiti that it is provided with its own group status.

**Table:** 

1	Pronotum distinctly narrowed basally, the lateral margins sinuate, straight, or convex anterad the hind angles, the basal width not apparently larger than the apical width	2
–	Pronotum subquadrate or trapezoidal, not or little narrowed basally, the basal width larger than the apical width, the hind angles distinctly angulate (Figs 10, 11, 12)	1. *Mecyclothorax muriauxi* species group
2	Standardized body length less than 7.5 mm	3
–	Body size larger, standardized body length 8.0–8.6 mm (Fig. 15)	2. *Mecyclothorax fosbergi* species group
3	Pronotal lateral margins convex to slightly sinuate for short distance anterad hind angles which are obtuse, blunt, or completely rounded	4
–	Pronotal lateral margins sinuate to distinctly sinuate anterad the hind angles which are well developed, obtuse, right, or in some instances acute	7
4	Pronotal basal setae present, the pronotum quadrisetose	5
–	Pronotal basal setae absent, only lateral setae present near midlength (Figs 16, 17, 20A, B)	3. *Mecyclothorax altiusculus* species group (in part)
5	Pronotal lateral margin straight to convex anterad rounded hind angle; a small toothlike projection may be associated with the articulatory socket of the basal seta	6
–	Pronotal lateral margin straight to slightly sinuate anterad distinct, obtuse hind angle (Figs 22, 23, 25, 26)	4. *Mecyclothorax dannieae* species group
6	Elytral striae very shallow, fine, discal elytral intervals nearly flat (Fig. 16A); pronotal hind angle obtuse-rounded, at most a small jag at insertion of basal seta (species polymorphic for presence of pronotal basal seta with only 2 of 49 specimens reported to possess seta; Perrault 1988: 238)	species 3–14 *Mecyclothorax jarrigei* Perrault of *Mecyclothorax altiusculus* species group
–	Elytral striae deep, well developed, punctate, the discal elytral intervals convex; pronotal hind angle obtuse, basal seta may be associated with a small jag or toothlike projection along base of lateral margin (Figs 28, 29A)	5. *Mecyclothorax striatopunctatus* species group
7	Pronotal basal seta present, pronotum quadrisetose	8
–	Pronotal basal seta absent, pronotum bisetose	9
8	Elytra narrowly ovoid, little convex, humeri very narrowly rounded; elytral disc brunneous, lateral margins and suture flavous (Figs 29B, C); dorsal elytral setae absent	6. *Mecyclothorax marginatus* species group
–	Elytra broader, humeri more broadly rounded; elytral disc rufobrunneous to rufopiceous, lateral margins and suture rufoflavous; dorsal elytral setae present or absent (Figs 31, 32)	7. *Mecyclothorax viridis* species group
9	Pronotal lateral marginal depression narrower, broadest basally outside laterobasal depression, narrower anterad	10
–	Pronotal lateral marginal depression subequally broad and explanate throughout length, surface broadly translucent in contrast to opaque disc (Figs 34, 35)	8. *Mecyclothorax gourvesi* species group
10	Pronotal lateral marginal depression narrow, especially in apical half of length where margin is beaded or narrowly upturned, pronotal margin distinctly sinuate basally anterad hind angle	11
–	Pronotal lateral marginal depression evident throughout length, pronotal margin extended from disc, margin not beadlike (Figs 20C, D, 21)	3. *Mecyclothorax altiusculus* species group (in part)
11	Pronotal and elytral bases broad, lateral margin joined to basal groove at nearly right angle at humerus, elytra subquadrate (Fig. 37A)	9. *Mecyclothorax tuea* species group
–	Pronotal and elytral bases narrow, humeral angle obtuse, subangulate, to rounded at humerus, margin narrowly and evenly rounded posteriorly, elytra ovate to obovate (Figs 37B–D, 38, 39, 41, 42, 45, 46, 48, 49)	10. *Mecyclothorax globosus* species group

### 1. *Mecyclothorax muriauxi* species group

**Figure 10.**
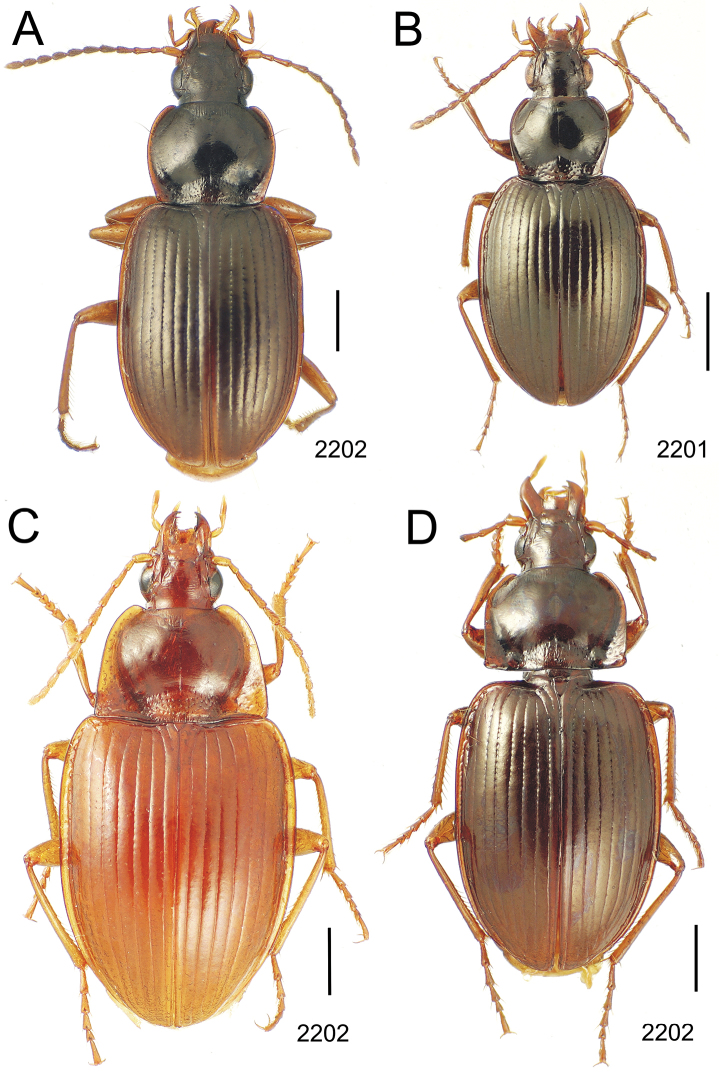
*Mecyclothorax* spp., dorsal view; scale bars 1.0 mm; setal formula (WXYZ) at lower right of each figure denotes number of supraorbital setae each side (W), number of pronotal setae each side (X), number of dorsal elytral setae each elytron (Y), and number of apical setae each elytron (Z) **A** *Mecyclothorax claridgeiae* holotype female **B** *Mecyclothorax subquadratus* holotype female **C** *Mecyclothorax muriauxi* holotype male **D** *Mecyclothorax mahina* holotype male

**Figure 11.**
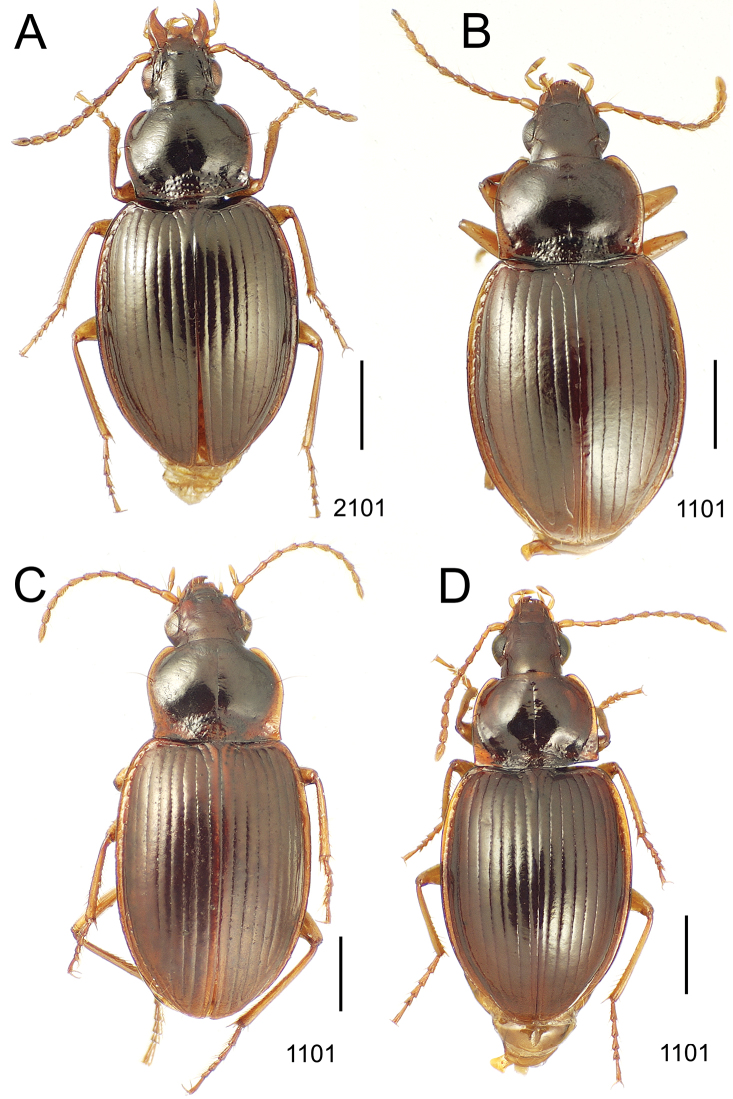
*Mecyclothorax* spp., dorsal view; scale bars 1.0 mm; setal formula (see Fig. 10) at lower right of each figure **A** *Mecyclothorax obtusus* holotype female **B** *Mecyclothorax gerardi* **C** *Mecyclothorax jeanyvesi* holotype female **D** *Mecyclothorax poria* holotype male.

**Figure 12.**
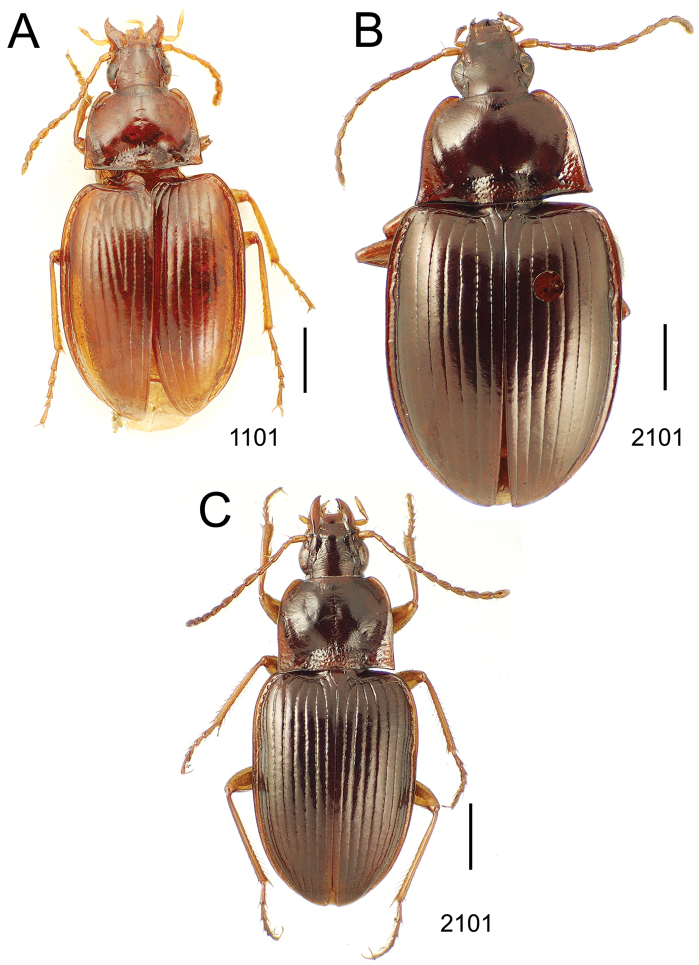
*Mecyclothorax* spp., dorsal view; scale bars 1.0 mm; setal formula (see Fig. 10) at lower right of each figure **A** *Mecyclothorax mapura* holotype male **B** *Mecyclothorax brevipennis* **C** *Mecyclothorax quadraticollis* holotype female.

**Diagnosis.** These species are characterized by broad bodies, with the elytra subparallel to broad basally and inflated behind (Figs 10–12). The pronotum is transverse and broad basally, with the basal width subequal to greater than the apical width. In several species the pronotum is clearly trapezoidal, i.e., broadest across the hind angles (Figs 10C, 12A–C). The elytra lack dorsal setae in beetles of all species. Standardized body lengths 5.1–8.4 mm.

#### Identification Key to Tahitian Species of the *Mecyclothorax muriauxi* species group

**Table:** 

1	Pronotum with two setae each side, one laterally near midlength and the second near or at hind angle	2
–	Pronotum with one seta each side near midlength	5
2	Pronotum narrowed posteriorly, maximum width at the level of the lateral setae	3
–	Pronotum not constricted basally, maximum pronotal width at the base	4
3	Both apical and subapical elytral setae present, setal formula 2202; pronotal lateral margin convex anterad hind angle, the angle marked by a minute jag just outside setal articulatory socket; elytral humeri broad, lateral margins subparallel outside anterior lateral setal series (Fig. 10A); standardized body length 7.2 mm (Pito Hiti)	1. *Mecyclothorax claridgeiae* sp. n.
–	Apical elytral seta present, subapical seta absent, setal formula 2201; pronotal lateral margin briefly sinuate before hind angle, the angle projected; humeri rounded, the elytral lateral margins divergent outside anterior lateral setal series (Fig. 10B); standardized body length 5.1 mm (Marau)	2. *Mecyclothorax subquadratus* Perrault
4	Elytral microsculpture consisting of a broad mesh, not or little transverse; pronotal margins broadly rounded anteriorly, lateral marginal depression broadly explanate throughout length (Fig. 10C); MPW/PL = 1.52–1.57 (Marau)	3. *Mecyclothorax muriauxi* Perrault
–	Elytral microsculpture consisting of a very narrow, transverse mesh; pronotal margins parallel for some distance basally, lateral marginal depression narrowed anteriorly (Fig. 10D); MPW/PL = 1.30 (Pito Hiti)	4. *Mecyclothorax mahina* Perrault
5	Pronotum narrowed basally, broadest at the level of lateral setae	6
–	Pronotum not narrowed basally, broader or of subequal breadth across the base versus across positions of pronotal lateral setae	8
6	One supraorbital seta, setal formula 1101	7
–	Two supraorbital setae (Fig. 11A), setal formula 2101 (Aorai)	5. *Mecyclothorax obtusus* Perrault
7	Pronotal lateral margins straight or only slightly sinuate anterad the hind angles which are obtuse to right, rounded at the apex (Fig. 11B); pronotal front angles distinctly projected anterad, margin straight from anteriormost point to juncture with discal convexity (Aorai)	6. *Mecyclothorax gerardi* Perrault
–	Pronotal lateral margins distinctly convergent anterad the hind angles, the sinuation angulate at point where margins anteriorly diverge, angles acute, laterally projected (Fig. 11C); pronotal front angles only slightly projected anterad, broadly rounded posterad (Pito Hiti)	7. *Mecyclothorax jeanyvesi* sp. n.
8	One supraorbital seta each side, setal formula 1101	9
–	Two supraorbital setae each side, setal formula 2101	10
9	Pronotal median base sloped anterad to meet disc, anterior portion of base smooth or with sparse shallow punctures (Fig. 11D), surface covered with shallow transverse-mesh microsculpture (Marau)	8. *Mecyclothorax poria* sp. n.
–	Pronotal median base distinctly depressed relative to disc, juncture with disc lined with distinct longitudinal strigae and elongate punctures (Fig. 12A), surface covered with distinct microsculpture, the sculpticells a mixture of isodiametric and transverse (Pito Hiti)	9. M. mapura Perrault
10	Pronotum distinctly transverse, MPW/PL = 1.49, lateral margin depression broad anteriorly (Fig. 12B); elytra very short; pronotal basal groove transverse, evenly curved from sutural stria to tightly rounded humerus (Marau)	10. *Mecyclothorax brevipennis* Perrault
–	Pronotum little transverse, MPW/PL = 1.26, lateral margin depression narrowed anteriorly (Fig. 12C); elytra very long; pronotal basal groove anteriorly recurved from base of sutural stria to angulate humerus (Marau)	11. *Mecyclothorax quadraticollis* Perrault

#### 1. Mecyclothorax claridgeiae sp. n.

http://zoobank.org/43786E69-7CEB-4FD1-8BB4-1939E950D8FC

http://species-id.net/wiki/Mecyclothorax_claridgeiae

##### Diagnosis.

Of the *Mecyclothorax muriauxi* group species with setal formula 2202, this is the only one with a pronotum that is evenly narrowed basally to the obtuse-rounded hind angles (Fig. 10A). Standardized body length 7.2 mm. *Head* broad with small eyes; frontal grooves broad, shallow, ill defined mesad eyes, deep and broad only near frontoclypeal suture; antennae elongate, filiform, antennomere 8 length 2.0× breadth; eyes little convex, sharing curvature with broad ocular lobe, ocular ratio 1.36, ocular lobe ratio 0.70. *Pronotum* subquadrate, moderately transverse, MPW/PL = 1.22; lateral margin moderately constricted before hind angle, MPW/BPW = 1.17; a minute jag at hind angle caused by flat articulatory socket of basal pronotal seta; anterior transverse impression broad, shallow, its outline obscured medially by about eight deep longitudinal strigae each side of midline; lateral margin moderately broad in apical half, broadly explanate and raised before hind angle; laterobasal depression deep, broad and smooth. *Elytra* broadly quadrate, MEW/HuW = 1.79; striae 1–6 well developed, densely punctate, the punctures expanding the strial breadth, separated along stria by length subequal to their diameter, stria 7 shallower, all striae smooth and complete apically; interval 8 broadly subcarinate, convexly protruded dorsad subapical sinuation; lateral elytral setae 7 + (5–6). *Microsculpture* on vertex a transverse mesh, sculpticell breadth 2× length, sculpticells more isodiametric on neck; pronotal disc with regular transverse mesh, breadth 2× length, median base with isodiametric sculpticells between punctures; elytral disc with transverse sculpticells, breadth 2–3× length, the sculpticells more deeply margined at elytral apex. *Coloration* of forebody rufopiceous, pronotal lateral margins and median base rufobrunneous; antennomeres 1–3 rufoflavous, 4–11 rufobrunneous; elytral disc rufobrunneous, the sutural interval rufous near scutellum, rufoflavous apically; femora rufoflavous, tibiae rufoflavous with brunneous cast.

Female reproductive tract. The lone female specimen was not dissected.

Holotype female (MNHN) labeled: French Polynesia: Tahiti Nui / Pito Hiti el. 2000 m 2-VI- / 2006 lot 02 pyrethrin fog / 17°36.790'S, 149°27.842'W / E.M. Claridge // HOLOTYPE / Mecyclothorax / claridgeiae / J.K. Liebherr 2013 (black-bordered red label).

##### Etymology.

The species epithet honors the collector, Dr. Elin Claridge, who collected the specimen during the first and only entomological expedition to Pito Hiti.

##### Distribution and habitat.

The type specimen of this species was collected via application of pyrethrin fog to moss-covered vegetation. Thus within the microhabitat of epiphytic mosses, the beetles may be considered subarboreal in habits.

#### 2. Mecyclothorax subquadratus

Perrault, 1984: 30

http://species-id.net/wiki/Mecyclothorax_subquadratus

##### Identification.

This is the smallest-bodied species in the *Mecyclothorax muriauxi* group to possess both lateral and basal pronotal setae; standardized body length 5.1 mm and setal formula 2201. The subquadrate pronotum has sinuate lateral margins and a broad base (Fig. 10B); MPW/PL = 1.20, APW/BPW = 0.78. The vertex of the head has shallow transverse-mesh microsculpture, the sculpticells difficult to discern in reflected light. The neck is covered with a more developed isodiametric mesh. The pronotal disc exhibits an evident transverse mesh, sculpticells 2–3× broad as long, and the discal elytral intervals are covered with a more distinct transverse mesh of the same dimensions.

##### Distribution and habitat.

The single holotype female specimen was collected at 1000 m elevation on Mont Marau.

#### 3. Mecyclothorax muriauxi

Perrault, 1978b: 143, 1984: 24

http://species-id.net/wiki/Mecyclothorax_muriauxi

Mecyclothorax muriauxioides Perrault, 1984: 24 (new synonymy).

##### Identification.

**Figure 13.**
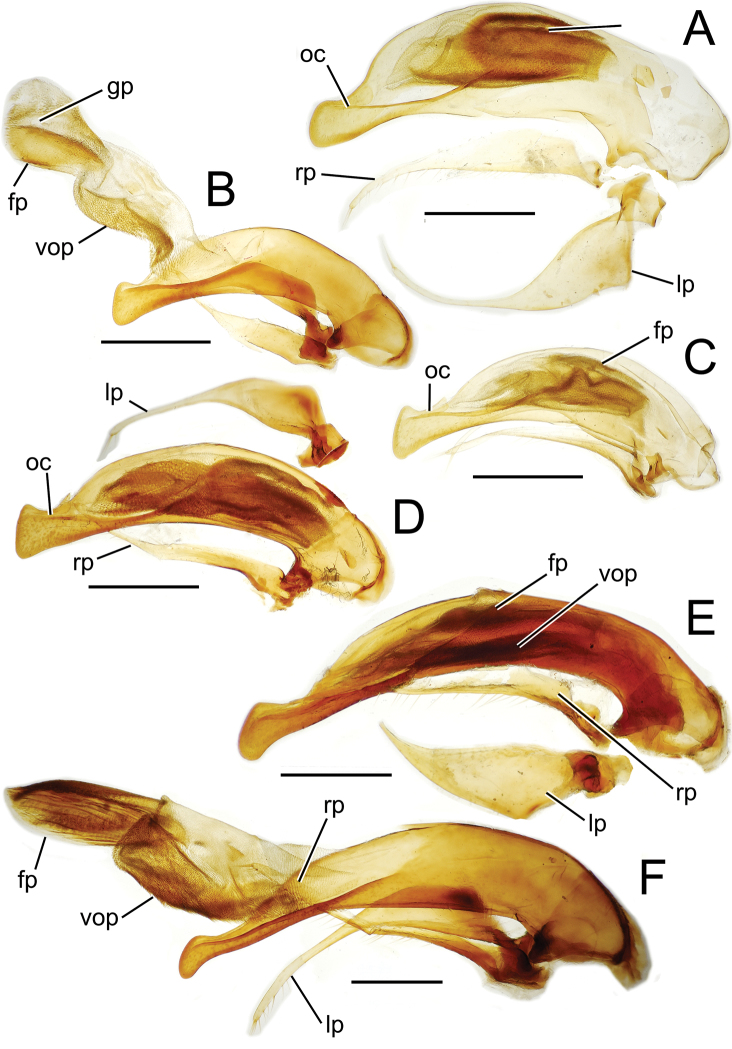
Male aedeagal median lobe and associated parameres, *Mecyclothorax* spp., right lateral view; scale bars 0.5 mm **A** *Mecyclothorax muriauxi* holotype **B** *Mecyclothorax gerardi*, internal sac everted **C** *Mecyclothorax poria* paratype (NMNH) **D** *Mecyclothorax mapura* holotype **E** *Mecyclothorax fosbergi* holotype **F** *Mecyclothorax fosbergioides* (EMEC), internal sac everted Abbreviations: **fp** flagellar plate **gp** gonopore **lp** left paramere **oc** ostial canal **rp** right paramere **vop** ventral ostial microtrichial patch.

This is a species characterized by dramatically robust proportions, with the very broadly explanate and upraised pronotal lateral margins expanded to a very broad pronotal base (Fig. 10C). The pronotal hind angles are setose, resulting in a setal formula of 2202. The head has very reduced microsculpture, though distinct transverse grooves emanate onto the frons from the frontal grooves. The pronotal disc is covered by an obsolete transverse mesh that is difficult to trace. The microsculpture of the discal elytral microsculpture is similarly difficult to discern, though subtle iridescence suggests the sculpticells are broader, therefore defining transverse lines. The male aedeagus has a broad, bluntly rounded apex and an elongate flagellar plate, plate length 0.48× the distance from the parameral articulation to the lobe apex (Fig. 13A). Standardized body length 7.6–8.0 mm.

The pronotal configurations of the male type of *Mecyclothorax muriauxi* and the two female types of *Mecyclothorax muriauxioides* do not seem diagnosably different to this author, and so the two names are herein considered synonymous. All three specimens possess both apical and subapical elytral setae, though the latter are broken off of the female paratype of *Mecyclothorax muriauxioides*.

##### Distribution and habitat.

The three specimens of this species have been collected at 1200 and 1300 m elevation on Mont Marau. This species is terricolous, with the 1200 m elevation specimen collected from under rocky debris, and those from 1300 m captured in a vinegar pitfall trap.

#### 4. Mecyclothorax mahina

Perrault, 1984: 27

http://species-id.net/wiki/Mecyclothorax_mahina

##### Identification.

This species shares a transverse pronotal configuration (Fig. 10D) with three other species in the group – *Mecyclothorax obtusus*, *Mecyclothorax gerardi*, and *Mecyclothorax poria* (Figs 11A, B, D) – but it is the only one to exhibit a quadrisetose pronotum; setal formula 2202. Also, the elytra are more parallel sided, and the elytral striae more distinctly punctate. The frons and vertex are glossy, with a reduced transverse mesh visible in the frontal grooves. The pronotum has patches of reduced isodiametric to transverse mesh over portions of the disc, and the elytra are covered with dense transverse lines causing iridescence. The male aedeagal median lobe is elongate, with a broadly expanded apex bearing an obtuse dorsal projection (Fig. 3C). Standardized body length 7.1 mm.

##### Distribution and habitat.

The only known specimen was collected in litter at 1000 m elevation, on Pihaaiateta along the ridge to Pito Hiti.

#### 5. Mecyclothorax obtusus

Perrault, 1984: 30

http://species-id.net/wiki/Mecyclothorax_obtusus

##### Identification.

Of the *Mecyclothorax muriauxi* group species with setal formula 2101 – the others include *Mecyclothorax brevipennis* and *Mecyclothorax quadraticollis* (Figs 12B, C) – this species exhibits sinuate pronotal lateral margins and a basal width less than the maximal width; MPW/BPW = 1.12 (Fig. 11A). The pronotum is moderately transverse; MPW/PL = 1.26, and the eyes do not protrude much from the ocular lobe; ocular ratio 1.38. This species is also among the smallest bodied species in the group; standardized body length 5.0 mm. The frons and vertex are covered with a distinct mesh of isodiametric and transverse sculpticells. The pronotal disc bears transverse mesh microsculpture, most visible outside the area of reflected light, and the discal elytral intervals are covered with an elongate transverse mesh, sculpticell breadth 2–3× length.

##### Distribution and habitat.

This species has been collected at l100–1400 m elevation along the Mont Aorai ridge. It is a terricolous species, with one specimen collected in moss, and a second captured in a vinegar pitfall trap.

#### 6. Mecyclothorax gerardi

Perrault, 1978b: 144, 1984: 29

http://species-id.net/wiki/Mecyclothorax_gerardi

##### Identification.

This species comprises beetles possessing only the posterior supraorbital seta and a transverse, bisetose pronotum; MPW/PL = 1.38 and setal formula 1101. The elytral humeri are distinctly angulate and the elytra are broadly ovoid, the lateral margins convex throughout their length (Fig. 11B). The vertex has transverse mesh microsculpture mesad the frontal grooves, the rows of sculpticells dissected by distinct transverse wrinkles emanating from the grooves. The pronotal disc is covered with a shallow but traceable transverse mesh, and the discal elytral intervals are lined with shallow transverse microsculpture consisting of elongate sculpticells intermixed with transverse lines. The male aedeagal median lobe has a broad apex that is rounded both dorsally and ventrally, and apically flattened (Fig. 13B). The aedeagal internal sac bears an elongate flagellar plate that is slightly more than half as long as the distance from the parameral articulations to the lobe apex, and a broadly distributed ventral ostial microtrichial patch. Standardized body length 5.3–5.4 mm.

##### Distribution and habitat.

The type series was collected from mosses at 1200 m elevation on the Mont Aorai ridge. Subsequently, two individuals were extracted through the use of pyrethrin fog from moss growing on *Metrosideros*. This second collecting event occurred at 1210 m elevation on Aorai.

#### 7. Mecyclothorax jeanyvesi sp. n.

http://zoobank.org/C054A778-328B-4DEF-A841-BC187623D75F

http://species-id.net/wiki/Mecyclothorax_jeanyvesi

##### Diagnosis.

The pronotum of this species is uniquely shaped among known members of this species group, with the lateral margins concave anterad slightly projected, acute hind angles (Fig. 13C). The eyes are convex and moderately protruded; ocular ratio 1.48, ocular lobe ratio 0.78. Only the posterior supraorbital and lateral pronotal setae are present, leading to a setal formula of 1101. Microsculpture of the dorsal body surface is well developed, the head with isodiametric and transverse sculpticells mixed in a regular mesh, the pronotal disc with a mixture of shallow isodiametric and transverse sculpticells, and the discal elytral intervals with a shallow transverse mesh, sculpticell breadth 2–3× length supporting indistinct iridescence. Standardized body length 6.0 mm. *Head* with broad, shallow, ill-defined frontal grooves, transverse wrinkles emanating from the grooves mesally onto frons; antennae short, moniliform, antennomere 8 length 1.55× maximal breadth. *Pronotum* moderately transverse, MPW/PL =1.33; pronotal base nearly as broad as maximal breadth, MPW/BPW = 1.05; front angles close together, APW/BPW = 0.65, the pronotal margin anteriorly rounded laterad the pronotal juncture with the neck; median base indistinctly depressed relative to disc near midline, more depressed laterally where juncture is marked by indistinct longitudinal wrinkles; laterobasal depression broad, impunctate, explanate lateral margin transversely wrinkled to canaliculate anterad hind angle; anterior transverse impression obsolete, mostly indicated by minute longitudinal wrinkles. *Elytra* moderately convex, maximally elevated from the depressed scutellum posteriorly to the apex of the parascutellar striole; striae 1–6 moderately incised, finely and regularly punctate throughout much of length, smooth in apical quarter, elytral intervals moderately convex; stria 7 shallow but continuous basally, finely incised, smooth at elytral apex where it is associated with the subcarinate eighth interval, that interval nearly vertically convex dorsad subapical sinuation; lateral elytral setae 7 + 6. *Coloration* of head capsule evenly rufobrunneous; antennomere 1 flavous, 2 rufoflavous, 3–11 rufobrunneous; pronotal disc dark rufous, anterior portion of lateral depression and laterobasal depression rufoflavous; elytral disc rufobrunneous, the sutural interval pale rufous near scutellum, rufoflavous apically; elytral apex graded to rufoflavous; femora flavous, tibiae flavous with a smoky cast.

Female reproductive tract. The single female specimen as not dissected.

Holotype female (MNHN) labeled: French Polynesia: Tahiti Nui / Pito Hiti el. 2070 m 2-VI- / 2006 lot 01 pyrethrin fog / 17°36.813'S, 149°27.842'W / E.M. Claridge // HOLOTYPE / Mecyclothorax / jeanyvesi / J.K. Liebherr 2013 (black-bordered red label).

##### Etymology.

The species epithet honors Dr. Jean-Yves Meyer, botanist extradordinaire, who accompanied Dr. Claridge on the expedition to Pito Hiti. The epithet is derived in a manner parallel to that of *Mecyclothorax gerardi* – to which this species bears close similarity – as that species was named to honor Gerard H. Perrault, a specialist on Formicidae, Tahitian collecting colleague, and the describer’s brother.

##### Distribution and habitat.

The single specimen was collected in a pyrethrin fog sample of moss-covered vegetation at 2070 m elevation on Pito Hiti, only 40 m elevation below the summit.

#### 8. Mecyclothorax poria sp. n.

http://zoobank.org/983750EA-0927-4ED2-952F-C55B17A52BFC

http://species-id.net/wiki/Mecyclothorax_poria

##### Diagnosis.

Of the four *Mecyclothorax muriauxi* group species with setal formula 1101 (Figs 11B–D, 12A), this species exhibits the smoothest pronotal median base, which bears 5–8 rounded punctures along the gently sloped juncture with the pronotal disc, and only indistinct transverse wrinkles basally near the pronotal hind margin. The pronotum is broadest basally, the basal width subequal to the width at midlength (Fig. 11D). The eyes are small in diameter, ocular lobe ratio 0.74–0.81 (n = 5), but convex, ocular ratio 1.44–1.50. Standardized body length 5.1–6.2 mm. *Head* with broad, shallow frontal grooves, fine transverse wrinkles emanating from grooves onto frons; posterior supraorbital seta situated dorsad fine shallow groove lining juncture of ocular lobe and gena; antennae submoniliform, short, antennomere 8 length 1.69× maximal breadth. *Pronotum* transverse, trapezoidal, the hind angles bluntly obtuse, the margin rounded behind, MPW/PL = 1.31–1.38; lateral margin straight anterad hind angle, but upraised and so appearing slightly sinuate; anterior transverse impression obsolete medially, narrow laterally where it is defined by a finely incised groove; front angles slightly protruded, rounded; lateral marginal depression narrow near midlength, edge beaded to just anterad hind angle. *Elytra* broad basally, humeri subangulate, lateral elytral margins convex (Fig. 11D); striae 1–5 evident, complete to basal groove, finely punctate in basal half and smooth apically, stria 6 shallower, stria 7 traceable as a series of isolated punctures basally, very shallow, obsolete apically, where it defines mesal border of slightly convex interval 8, that interval only indistinctly subcarinate apicad position of subapical sinuation; discal elytra intervals slightly convex; lateral elytral setae (6–7) + (5–6). *Microsculpture* obsolete on frons, indistinct isodiametric mesh on neck; pronotal disc with shallow transverse microsculpture, sculpticell breadth 2–4× length; pronotal median base glossy, transverse mesh indistinct; discal elytral intervals covered with mixed elongate transverse mesh and transverse lines. *Coloration* of vertex rufous; antennomere 1 flavous, 2–3 brunneous with apex flavous, 4–11 darker, brunneous; pronotal disc rufous, the margins broadly rufoflavous; elytral disc rufous, marginal depression and humeral angle rufoflavous; femora flavous with brunneous cast, tibiae slightly more brunneous.

Male genitalia. Aedeagal median lobe blunt apically, with a broadly rounded, slightly convex ventral face and more pointed dorsal projection (Figs 3D, 13C); ostial canal straight, terminated just ventrad the dorsal projection. The five *Mecyclothorax muriauxi* group species for which males are known all possess similar median lobes, with *Mecyclothorax poria*, *Mecyclothorax gerardi*, and *Mecyclothorax mapura* exhibiting almost identical median lobe apices. The lobe apex of *Mecyclothorax poria* (Fig. 13C) is narrower dorsoventrally than that of *Mecyclothorax mapura* (Fig. 13D), and broader and more flattened apically than that of *Mecyclothorax gerardi* (Fig. 13B). Nevertheless, in these taxa, external characters can better distinguish the taxa.

**Figure 14.**
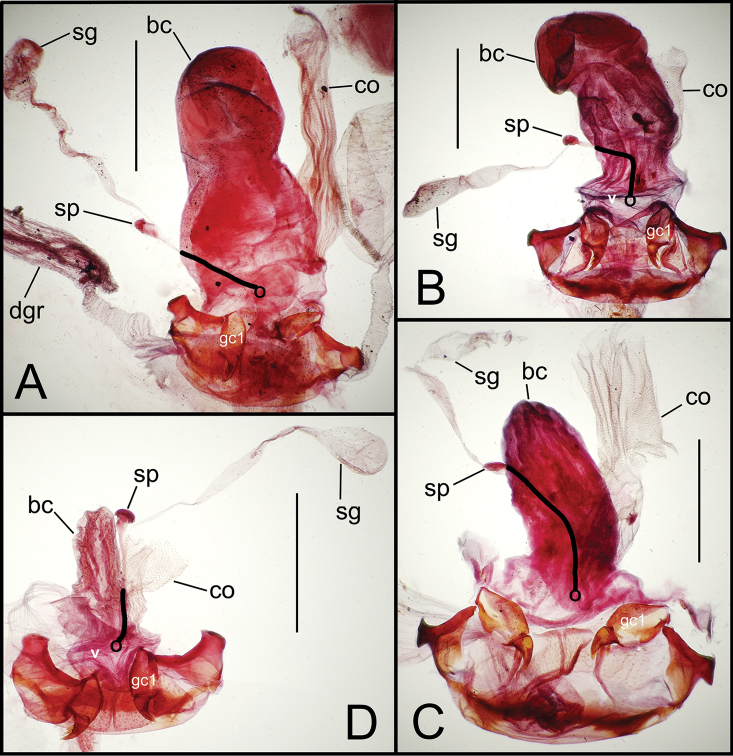
Female reproductive tract dissections, *Mecyclothorax* spp., ventral view; scale bars 0.5 mm **A** *Mecyclothorax poria* paratype (CUIC) **B** *Mecyclothorax everardi* paratype (CUIC) **C** *Mecyclothorax pitohitiensis* paratype (CUIC) **D** *Mecyclothorax hoeahiti* paratype (CUIC) Abbreviations: **bc** bursa copulatrix **co** common oviduct **dgr** defensive gland reservoir **gc1** basal gonocoxite 1 **sg** spermathecal gland **sp** spermatheca **v** vagina. Position of spermathecal duct and juncture of duct with dorsal wall of bursa indicated by black line and terminal circle, respectively.

Female reproductive tract. The bursa copulatrix of *Mecyclothorax poria* females is broadened basally, with no evidence of a discrete vagina (Fig. 14A). The bursa in the single known female has a smooth surface and a constriction just beyond midlength, and is approximately twice as long as its maximal breadth when compressed under a cover slip. The female basal gonocoxite exhibits an apical fringe of 3–4 setae (Fig. 8B), and approximately eight setae along the medial margin. The apical gonocoxite is broadened basally resulting in a broadly arcuate lateral margin. It bears two parallel-sided lateral ensiform setae, a dorsal ensiform seta, and an apical sensory furrow with two nematiform setae plus two furrow pegs.

Holotype female (MNHN) labeled: French Polynesia: Tahiti Nui / Mt. Marau road el. 1275 m / 10-IX-2006 lot 02 / 17°36.433'S, 149°32.339'W / pyr. fog horiz. *Weinmannia* / trunks + veg. J.K. Liebherr // HOLOTYPE / Mecyclothorax / poria / J.K. Liebherr 2013 (black-bordered red label).

Allotype female (MNHN) labeled: SOCIETY IS: Tahiti / Tahiti Nui Mont Marau / 1280 m el. 6-XI-1999 / D.A. Polhemus pyr. fog / sta. 1 *Weinmannia* for. // ALLOTYPE / Mecyclothorax / poria / J.K. Liebherr 2013 (black-bordered red label).

Paratypes: same data as allotype (CUIC, 2; NMNH, 1); Tahiti Nui, Mont Marau, Mt. Marau road, 1315 m el., 17°36.433'S, 149°32.333'W, 10-IX-2006 lot 05, Polhemus, pyr. fog mossy *Weinmannia* w/ *Astelia* (NMNH, 1)

##### Etymology.

The species epithet, poria, means fat or corpulent in Tahitian, the epithet signifying the broad outline of the body caused by the basally broadened pronotum and broadly based, laterally convex elytra.

##### Distribution and habitat.

All samples containing this species have come from the Mont Marau ridge between 1275 and 1315 m elevation. All specimens of the type series have been obtained by fogging moss-covered *Weinmannia* trees, in one instance when an *Astelia* plant was growing on the *Weinmannia* tree.

#### 9. Mecyclothorax mapura

Perrault, 1984: 28

http://species-id.net/wiki/Mecyclothorax_mapura

##### Identification.

Among *Mecyclothorax muriauxi* group species with setal formula 1101, this species exhibits the smallest, least convex eyes (Fig. 12A); ocular ratio 1.43, ocular lobe ratio 0.76. As in *Mecyclothorax poria*, the bisetose pronotum is broadest basally, however in this species the median base is margined by about seven longitudinal strigae along its juncture with the convex disc. The dorsal surface of the head capsule is glossy, with only indistinct sculpticells associated with wrinkles emanating from the frontal grooves. The pronotal disc is covered with a shallow, indistinct transverse mesh, the sculpticell breadth 2–3× length, and the discal elytral intervals exhibit dense transverse microsculpture; a mixture of transverse mesh and transverse lines. The male aedeagal median lobe apex is broadly expanded dorsoventrally, with the ventral surface not expanded from the more basal curvature of the shaft’s ventral surface (Fig. 13D). As in other species in the group, the male flagellar plate is elongate, its length 0.5× the distance from parameral articulations to the flattened apex. Standardized body length 6.1 mm.

##### Distribution and habitat.

The holotype specimen was collected between 900 and 1200 m elevation on Mapura, the ridge NNW of Pihaaiateta of the Pito Hiti-Orohena massif.

#### 10. Mecyclothorax brevipennis

Perrault, 1984: 26

http://species-id.net/wiki/Mecyclothorax_brevipennis

##### Identification.

This species is characterized by the broadest, most robust body, and the acutely protruded, glabrous, pronotal hind angles (Fig. 12B). The elytra are remarkably broad and foreshortened, with the humerus broadly extended and rounded at the base of the lateral marginal depression. The head is covered with transversely stretched isodiametric sculpticells arranged in transverse rows. The pronotal disc bears a shallow transverse mesh, the sculpticell breadth 2–4× length, and the discal elytral intervals are covered with a well-developed transverse mesh with sculpticell breadth 2–3× length. Setal formula 2101; standardized body length 7.2–8.1 mm.

##### Distribution and habitat.

Perrault collected the female holotype, 20-xii-1977, at 1000 m elevation on Mont Marau. Earlier that same year – 29–30-vi-1977 – another female was collected “at night” from the summit of Marau, 1490 m elevation (W.C. Gagné and S.L. Montgomery; BPBM).

#### 11. Mecyclothorax quadraticollis

Perrault, 1984: 29

http://species-id.net/wiki/Mecyclothorax_quadraticollis

##### Identification.

Unique among the *Mecyclothorax muriauxi* group in the parallel-sided elytra (Fig. 12C), this species can also be diagnosed by the broad head and little convex eyes; ocular ratio 1.40. The pronotum is quadrate with the lateral margins gently concave and upraised anterad the projected, acute hind angles. The discal elytral striae are deep with elongate punctures, the intervals convex. The frons and vertex are distinctly microsculptured, the sculpticells a mixture of isodiametric and transverse. The pronotum bears transverse mesh microsculpture that produces a subiridescent sheen, and the discal elytral intervals are lined with transverse sculpticells, their breadth 2–3× their length. Setal formula 2101; standardized body length 6.1 mm.

##### Distribution and habitat.

The holotype female was beaten from dead leaves at 1400 m elevation on Mont Marau.

### 2. *Mecyclothorax fosbergi* species group

**Figure 15.**
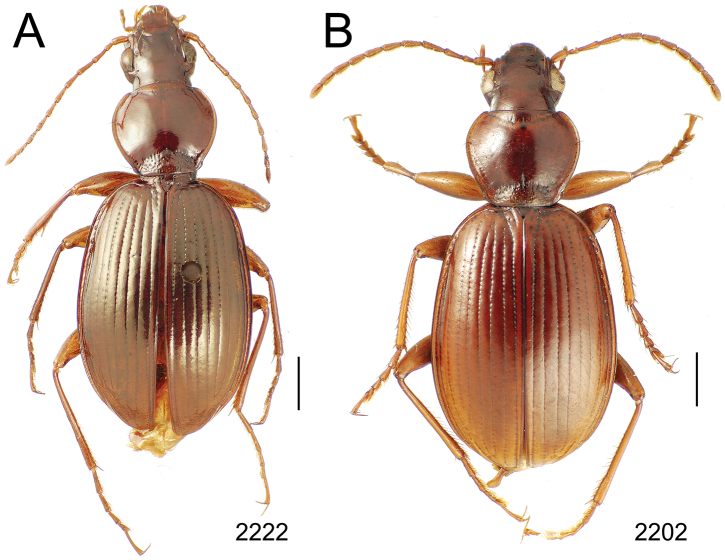
*Mecyclothorax* spp., dorsal view; scale bars 1.0 mm; setal formula (see Fig. 10) at lower right of each figure **A** *Mecyclothorax fosbergi* holotype male (BPBM) **B** *Mecyclothorax fosbergioides* male (EMEC).

**Diagnosis.** The two species of this group comprise large bodied beetles of lanky proportions – standardized body length 8.2–8.9 mm – that exhibit a narrow, quadrisetose pronotum, and basally narrowed elytra bearing well-developed, punctate elytral striae (Fig. 15).

#### Identification key to Tahitian species of the *Mecyclothorax fosbergi* species group

**Table:** 

1	Vertex and neck impression glossy, surface smooth, without evident sculpticells; pronotal lateral margins explanate, translucent throughout length, median base with distinct rounded punctures in lateral reaches; two dorsal elytral setae in third interval (Fig. 15A) (Papenoo Vy.–Mont Orohena)	12. *Mecyclothorax fosbergi* Perrault
–	Vertex and neck impression with evident, regular transverse-mesh microsculpture; pronotal lateral margins narrowed anteriorly, surface largely opaque with isolated spots of translucent cuticle (best viewed from ventrolateral aspect); third elytral interval lacking dorsal elytral setae (Fig. 15B) (Aorai + Pito Hiti)	13. *Mecyclothorax fosbergioides* Perrault

#### 12. Mecyclothorax fosbergi

Perrault, 1979: 9; 1988: 232

http://species-id.net/wiki/Mecyclothorax_fosbergi

##### Identification.

Relative to *Mecyclothorax fosbergioides*, this species can be diagnosed immediately by the reduced microsculpture that results in a glossy dorsum. The pronotum is narrow, MPW/PL = 1.07, and the humeral angles are proximate due to the narrow elytral base, MEW/HuW = 2.36. The male aedeagal median lobe is elongate and narrow, the apex expanded both dorsally and moreso ventrally, with a flattened apical face (Fig. 13E). The male flagellar plate is moderately long, its length estimated at 0.45× the distance from parameral articulations to the apex in the uneverted aedeagus. Setal formula 2222; standardized body length 8.25 mm.

##### Distribution and habitat.

This species is known only from the holotype specimen collected by the botanist F.R. Fosberg, from dead *Cyathea* fern fronds at 915 m elevation above the floor of Papenoo Valley on the lower flank of Mont Orohena.

#### 13. Mecyclothorax fosbergioides

Perrault, 1988: 232

http://species-id.net/wiki/Mecyclothorax_fosbergioides

##### Identification.

The largest bodied Tahitian *Mecyclothorax* at standardized body length 8.6–8.9 mm, and sharing the lanky proportions of its group mate, *Mecyclothorax fosbergi* (Fig. 15). However this species is characterized by a broader pronotum, MPW/PL = 1.18, and relatively narrower humeri, MEW/HuW = 2.52, with the elytra more broadly obovate in shape. The head bears shallow isodiametric sculpticells, and the pronotum is covered by a shallow elongate transverse mesh that produces an indistinct iridescence. The discal elytral intervals are covered with a dense, regular mesh, a mixture of isodiametric and transverse sculpticells, the latter twice as broad as long. The male aedeagal median lobe – first reported here – is very similar to that of *Mecyclothorax fosbergi* (Fig. 13E), although the shaft is somewhat broader (Fig. 13F). Also, the apex is much more elongate and narrower dorsoventrally, though a downturned tip and flattened apical face are shared. The internal sac bears a moderately long flagellar plate, length 0.48× distance from parameral articulations to apical face. Setal formula 2202.

##### Distribution and habitat.

The holotype female was collected by J. Gourvès at 1900 m elevation on Mont Aorai, whereas the male first reported above was collected by E.M. Claridge at 2070 m on Pito Hiti (EMEC). These two localities are an estimated 5.5 km apart taking the ridge distance between them. The microsculpture of the male is somewhat less developed than that of the female holotype. For the present the two specimens are considered conspecific, with the discovery of a male specimen from Mont Aorai the best arbiter for establishing the conspecificity or distinctiveness of the Aorai and Pito Hiti populations.

### 3. *Mecyclothorax altiusculus* species group

**Figure 16.**
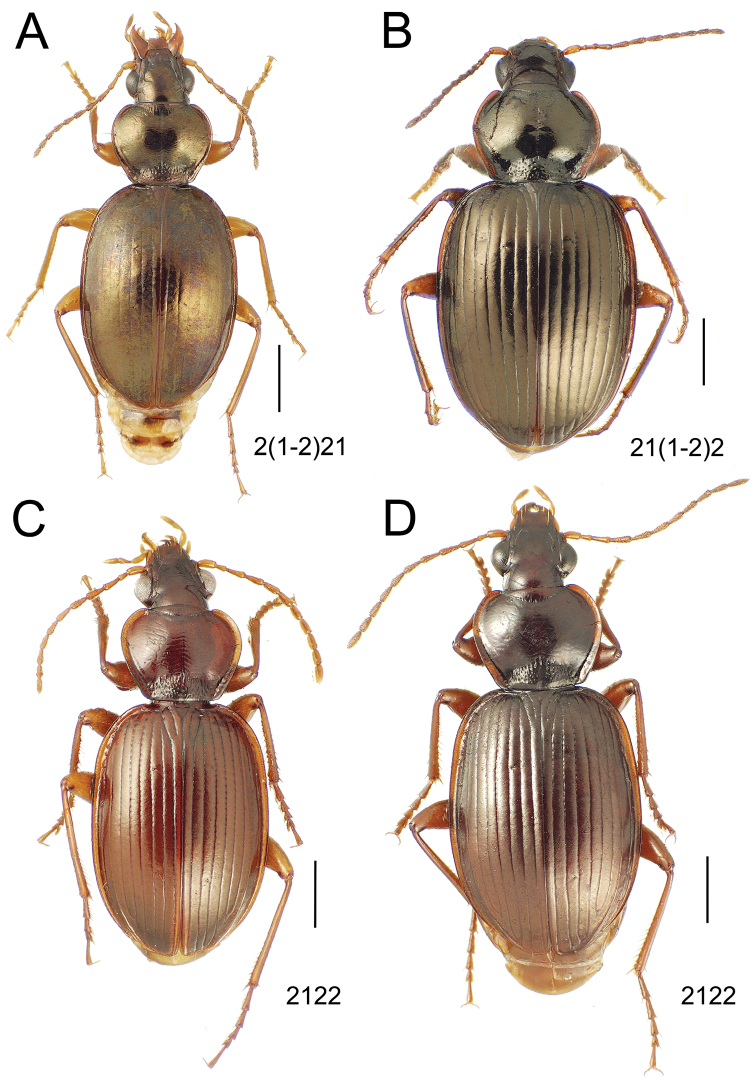
*Mecyclothorax* spp., dorsal view; scale bars 1.0 mm; setal formula (see Fig. 10) at lower right of each figure **A** *Mecyclothorax jarrigei* paratype female (NHMB) **B** *Mecyclothorax aano* holotype male **C** *Mecyclothorax hamatus* **D** *Mecyclothorax altiusculoides*

**Figure 17.**
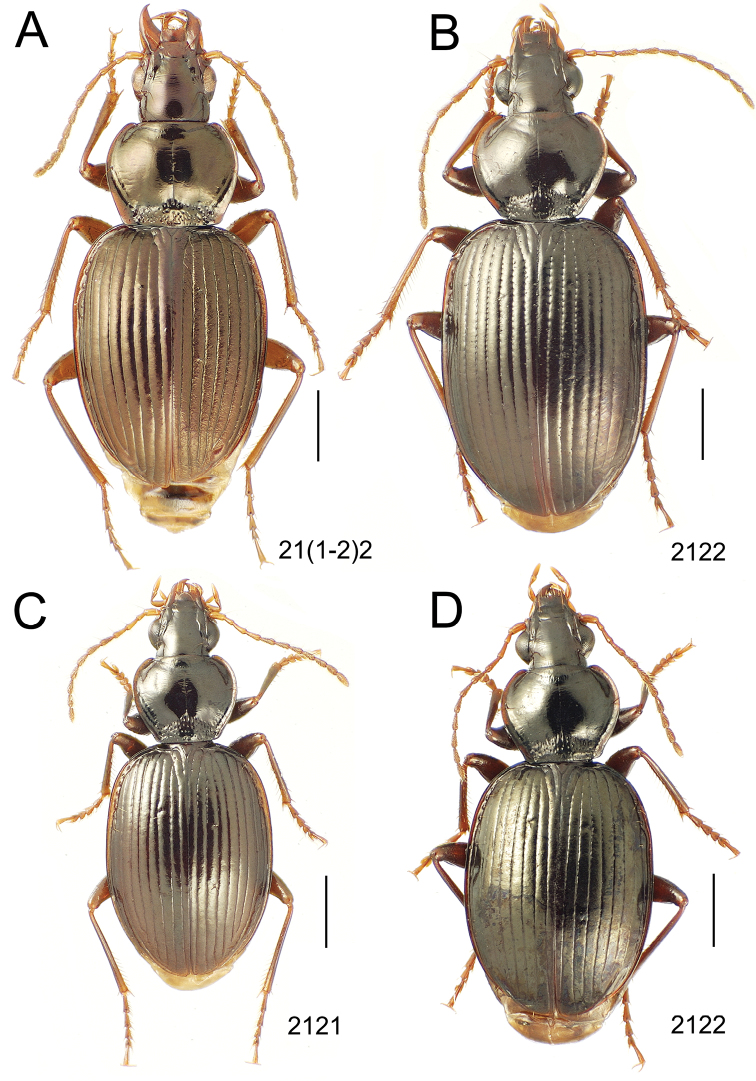
*Mecyclothorax* spp., dorsal view; scale bars 1.0 mm; setal formula (see Fig. 10) at lower right of each figure **A** *Mecyclothorax tuberculatus* holotype female **B** *Mecyclothorax paraltiusculus* **C** *Mecyclothorax pseudaltiusculus* **D** *Mecyclothorax altiusculus*

**Figure 18.**
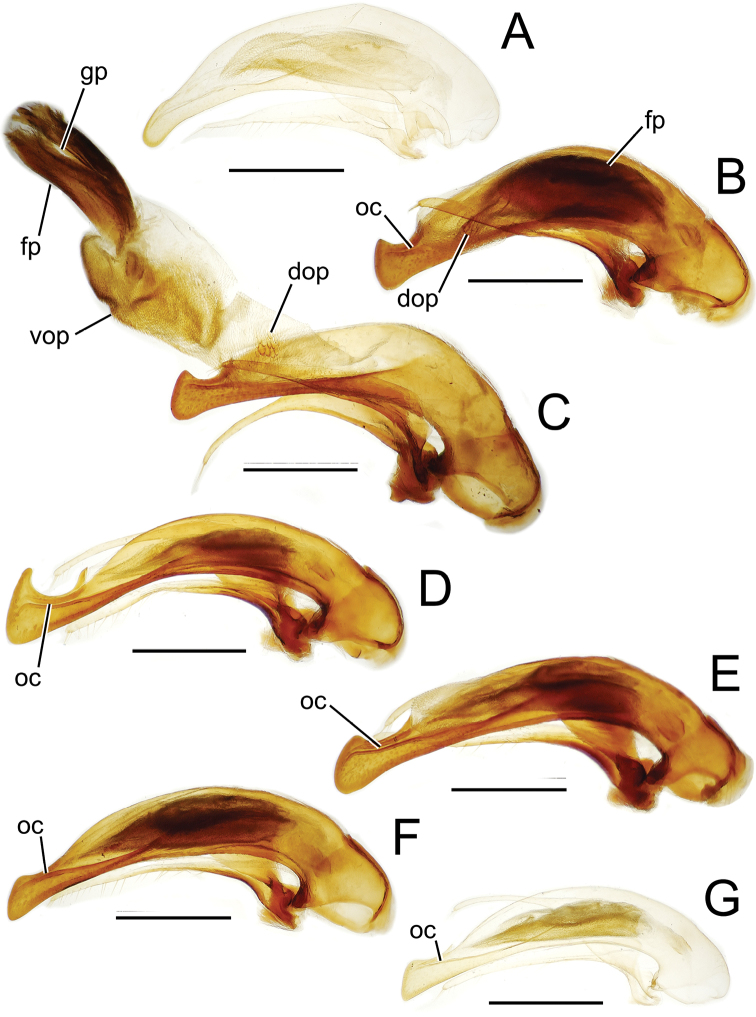
Male aedeagal median lobe and associated parameres, *Mecyclothorax* spp., right lateral view; scale bars 0.5 mm **A** *Mecyclothorax jarrigei*, teneral, Pito Hiti, 2000 m el. (EMEC) **B** *Mecyclothorax aano* holotype **C** *Mecyclothorax aano holotype*, internal sac everted **D** *Mecyclothorax hamatus* **E** *Mecyclothorax altiusculoides* **F** *Mecyclothorax paraltiusculus* **G** *Mecyclothorax pseudaltiusculus*, teneralAbbreviations: **dop** dorsal ostial microtrichial patch **fp** flagellar plate **gp** gonopore **oc** ostial canal **vop** ventral ostial microtrichial patch.

**Figure 19.**
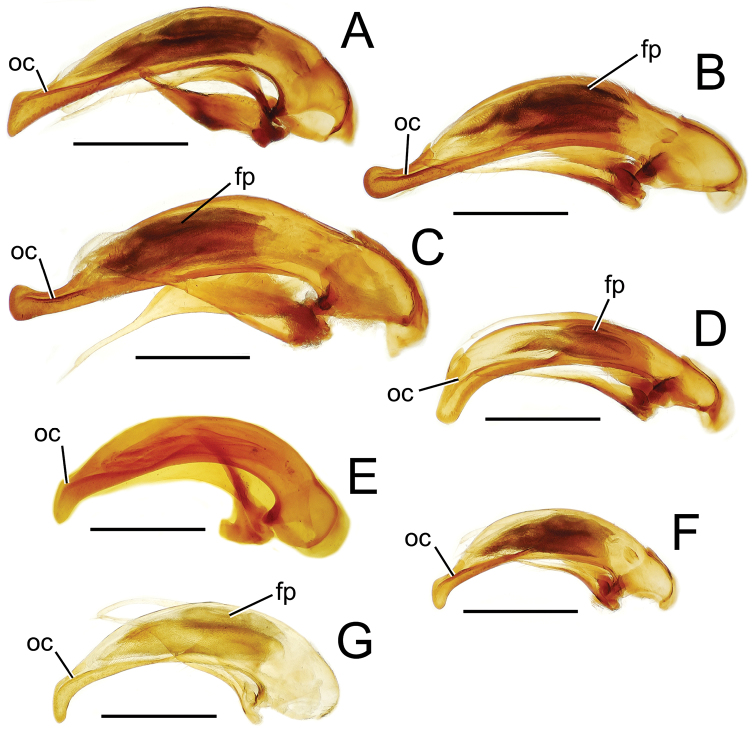
Male aedeagal median lobe and associated parameres, *Mecyclothorax* spp., right lateral view; scale bars 0.5 mm **A** *Mecyclothorax altiusculus* **B** *Mecyclothorax ovalipennis* **C** *Mecyclothorax parovalipennis* **D** *Mecyclothorax tihotii* holotype **E** *Mecyclothorax bryobius* lectotype (BPBM), aedeagus mounted in Canada balsam **F** *Mecyclothorax ballioides* **G** *Mecyclothorax ferruginosus* holotype, teneral **oc** ostial canal **fp** flagellar plate.

**Figure 20.**
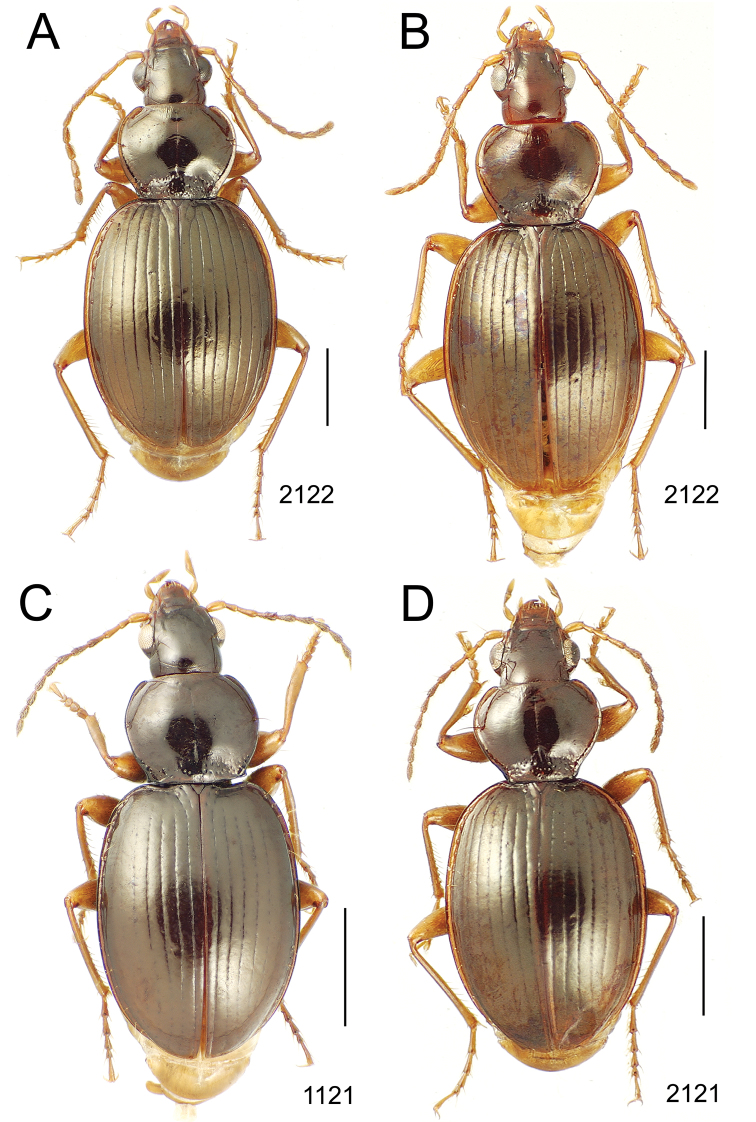
*Mecyclothorax* spp., dorsal view; scale bars 1.0 mm; setal formula (see Fig. 10) at lower right of each figure **A** *Mecyclothorax ovalipennis* **B** *Mecyclothorax parovalipennis* holotype male **C** *Mecyclothorax tihotii* holotype male **D** *Mecyclothorax bryobioides*.

**Figure 21.**
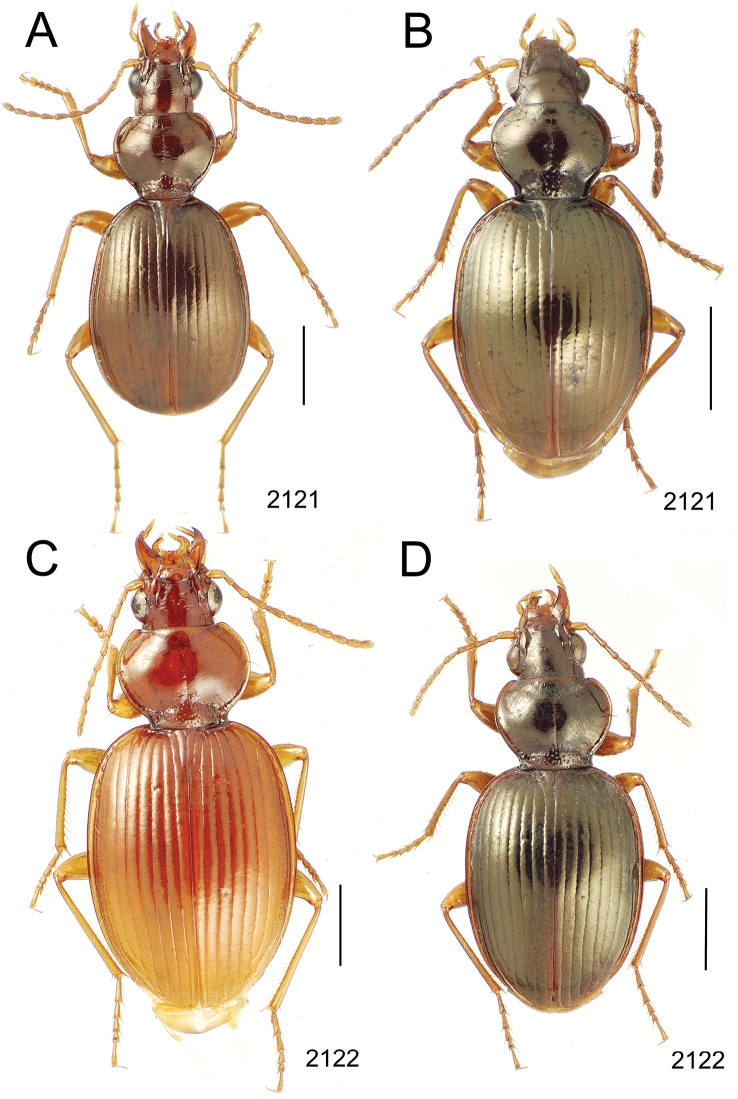
*Mecyclothorax* spp., dorsal view; scale bars 1.0 mm; setal formula (see Fig. 10) at lower right of each figure **A** *Mecyclothorax bryobius* paratype female (MNHN) **B** *Mecyclothorax ballioides* **C** *Mecyclothorax ferruginosus* holotype male **D** *Mecyclothorax sinuatus* holotype female.

**Diagnosis.** Species in this group are characterized by glabrous pronotal hind angles; the lone exception being two specimens of *Mecyclothorax jarrigei* Perrault (out of 49 known) which exhibit setose hind angles. The pronotal lateral margin may be straight, slightly sinuate, or even distinctly sinuate anterad the hind angle, but the pronotal lateral margin is never explanate, broad nor translucent (Figs 16, 17, 20, 21). With the exception of *Mecyclothorax jarrigei*, the elytral striae are deep and well developed. The elytra are ovate with narrowly rounded humeri, or obovate with the greatest breadth behind midlength; e.g., *Mecyclothorax bryobius* (Fig. 21A). As noted by Perrault (1988), there are three basic conformations of the aedeagal median lobe: 1, apex dorsoventrally expanded, sometimes bearing a dorsal projection (Figs 18, 19A); 2, apex elongate, the tip rounded (Figs 19B, C); and 3, apex downturned (Figs 19D–G). The latter configuration is also observed in *Mecyclothorax pirihao* Liebherr (Figs 3G, 47F), currently placed in the *Mecyclothorax globosus* group based on pronotal conformation. There are two Moorean species also assigned to this group (Liebherr 2012a). Standardized body lengths 4.1–7.0 mm for the Tahitian species.

#### Identification key to Tahitian species of the *Mecyclothorax altiusculus* species group

**Table:** 

1	Basal pronotal seta absent	2
–	Basal pronotal seta present (rare setal configuration observed in 2 of 49 specimens) (Aorai)	14. *Mecyclothorax jarrigei* Perrault (in part)
2	Pronotal hind angles rounded, either broadly rounded, or more narrowly rounded with the lateral margin straight or only slightly sinuate anterad the angle	3
–	Pronotal hind angles distinctly indicated, basal margin sinuate anterad hind angle, either very briefly sinuate with hind angle denticulate, or more broadly sinuate with the hind angle obtuse	12
3	Striae deep, distinct; elytral coloration flavous to piceous, but without metallic reflection	4
–	Discal elytral striae shallow (Fig. 16A); elytral coloration rufobrunneous with greenish reflection (Aorai + Pito Hiti)	14. *Mecyclothorax jarrigei* Perrault (in part)
4	Pronotal lateral depression and elytral marginal depression posterad humerus broad and deep, anterior lateral elytral seta more than twice distance to elytral margin as breadth of elytral interval 9	5
–	Pronotal lateral depression and elytral marginal depression posterad humerus narrower, distance of anterior lateral elytral seta to elytral margin no more than breadth of elytral interval 9	7
5	Pronotal lateral margin broadly and evenly reflexed along entire pronotal length; elytral humeri angulate, elytral base narrow, MEW/HuW = 2.25–2.50	6
–	Pronotal lateral margin broadly elevated basally, narrow anteriorly, pronotum appearing cychroid; elytral humeri rounded, elytral base broad (Fig. 16B), MEW/HuW = 2.12 (Pito Hiti)	15. *Mecyclothorax aano* sp. n.
6	Pronotal anterior transverse impression complete medially (Fig. 16C); aedeagal median lobe apex with distinct tooth dorsally (Fig. 18D) (Marau)	16. *Mecyclothorax hamatus* Perrault
–	Pronotal anterior transverse impression effaced medially (Fig. 16D); aedeagal median lobe apex expanded dorsoventrally, but not toothed (Fig. 18E) (Aorai)	17. *Mecyclothorax altiusculoides* Perrault
7	Apex of elytral interval 7 without projected tubercle; pronotal hind angles completely rounded	8
–	Apex of elytral interval 7 with a projected tubercle (Fig. 17A); pronotal hind angles obtuse, blunt (Aorai)	18. *Mecyclothorax tuberculatu* s Perrault
8	Elytral striae not or indistinctly punctate basally	9
–	Elytral striae distinctly punctate basally, intervals convex (Fig. 17B) (Teatara)	19. *Mecyclothorax paraltiusculus* Perrault
9	Elytral microsculpture irregularly isodiametric	10
–	Elytral microsculpture transverse	11
10	Elytral striae finely punctate basally; elytral lateral marginal depression moderately explanate outside anterior series of elytral lateral setae (Fig. 17C); standardized body length 5.5–5.6 mm (Marau)	20. *Mecyclothorax pseudaltiusculus* Perrault
–	Elytral striae smooth, impunctate; elytral lateral marginal depression very narrowly reflexed outside anterior series of elytral lateral setae (Fig. 17D); standardized body length 5.5–6.5 mm (Aorai)	21. *Mecyclothorax altiusculus* (Britton)
11	Elytral microsculpture consisting of fine transverse lines with little tendency to form a mesh (Marau)	22. *Mecyclothorax ovalipennis* Perrault
–	Elytral microsculpture a transverse mesh (Aorai)	23. *Mecyclothorax parovalipennis* Perrault
12	Pronotal hind angles obtuse to obtuse-rounded, pronotal base narrow, MPW/BPW = 1.55–1.95	13
–	Pronotal hind angles toothlike, little projected (Fig. 20C), pronotal base broader, MPW/BPW = 1.33–1.36 (Mauru)	24. *Mecyclothorax tihotii* Liebherr
13	Pronotal lateral margins divergent immediately anterad obtuse-rounded hind angles, margin only slightly concave resulting in little-developed sinuation	14
–	Pronotal basal margins subparallel laterad laterobasal depression, the hind angles prominent, distinctly obtuse, lateral pronotal margin distinctly sinuate	15
14	Elytra ovate, maximum width near midlength (Fig. 20D); pronotal hind angles obtuse but distinct posterad the shallow lateral sinuation (Teatara)	25. *Mecyclothorax bryobioides* Perrault
–	Elytra obovate, humeri extremely narrow, maximum elytral width well behind midlength (Fig. 21A); pronotal hind angles rounded posterad shallow sinuation (Aorai)	26. *Mecyclothorax bryobius* (Britton)
15	Discal elytral striae well developed, associated intervals broadly and evenly convex; both apical and subapical elytral seta present, setal formula 2122; standardized body length 4.5–5.3 mm	16
–	Discal elytral striae shallow, associated intervals only slightly convex (Fig. 21B); apical elytral seta present, subapical seta absent, setal formula 2121; standardized body length 4.0–4.4 mm (Marau)	27. *Mecyclothorax ballioides* Perrault
16	Pronotum broadly transverse, MPW/PL = 1.33, basally constricted, MPW/BPW = 1.72 (Fig. 21C); discal elytral striae minutely punctate, parascutellar striole deep, extended half the distance from basal groove to anterior dorsal elytral seta; standardized body length 6.0 mm (Teatara)	28. *Mecyclothorax ferruginosus* Perrault
–	Pronotum less transverse, MPW/PL = 1.25, broader basally, MPW/BPW = 1.59 (Fig. 21D); discal elytral striae distinctly punctate, parascutellar striole shallow, extended less than 1/3 the distance from basal groove to anterior dorsal elytral seta; standardized body length 5.0 mm (Teatara)	29. *Mecyclothorax sinuatus* Perrault

#### 14. Mecyclothorax jarrigei

Perrault, 1978b: 134; 1988: 238

http://species-id.net/wiki/Mecyclothorax_jarrigei

##### Identification.

Among species of the *Mecyclothorax altiusculus* group, this species stands out based on the narrowly ovate elytra with narrow lateral marginal depressions, and the very shallow elytral striae, the intervening intervals nearly flat (Fig. 16A). The vertex is covered with evident transverse-mesh microsculpture. The surface of the pronotal disc is glossier, though shallowly margined transverse sculpticells are visible outside areas of reflected light, and the discal elytral intervals bear transverse microsculpture, the sculpticells either isodiametric and arranged in transverse rows, or slightly transverse in dimension. Setal formula 2121 in most individuals; of 49 specimens examined, only 1 had paired basal pronotal setae, and a second unilaterally exhibited a seta at the left hind angle (Fig. 16A). The male aedeagal median lobe is evenly narrowed to the apical margin of the ostium, with the apex gently downturned with a rounded tip (Fig. 18A). This simple configuration is closest to the median lobe with more downturned apex observed in *Mecyclothorax bryobius* (Fig. 19E). Standardized body length 5.8–6.3 mm.

##### Distribution and habitat.

This species was previously known (Perrault 1988) from 1750m and 2000 m elevations on Mont Aorai. More recently – 2-vi-2006 (EMEC) – a single teneral specimen was collected in a pyrethrin fog sample from moss-covered vegetation at 2000 m elevation on Pito Hiti.

#### 15. Mecyclothorax aano sp. n.

http://zoobank.org/5A9CBF2A-9942-448E-BD38-14B83940F2A8

http://species-id.net/wiki/Mecyclothorax_aano

##### Diagnosis.

This is the broadest bodied species of the *Mecyclothorax altiusculus* group (Fig. 16B), with a broad pronotum – MPW/PL = 1.29 – and relatively broad elytra standardized by maximum head width; MEW/MHW = 2.45. The elytra are polymorphic for the number of dorsal setae; the left elytron bearing two setae and the right only the anterior seta; thus the setal formula 21(1–2)2. Standardized body length 6.9 mm. *Head* with broadly convex frons, the frontal grooves narrow posterad and separated from the anterior supraorbital seta by a thin but distinct carina; eyes somewhat bulging dorsally, ocular ratio 1.49, ocular lobe ratio 0.80, the ocular lobe joining the gena at a narrow groove; antennae elongate, filiform, antennomere 8 length 2.33× maximal breadth. *Pronotum* basally with broadly explanate and upraised lateral margins, the hind angles indicated by a flattened expansion of the margin that looks to all intents and purposes the site for placement of a basal seta (but no seta is present either side); median base distinctly depressed relative to the very convex disc, about 24 small, isolated punctures each side; anterior transverse impression finely, distinctly incised for much of breadth, shallower at midline, with indistinct longitudinal wrinkles crossing anterior callosity; lateral marginal depression narrowest just anterad lateral seta, slightly broader inside broadly rounded front angles, evenly broadened posterad lateral seta to hind angle. *Elytra* with striae 1–8 deep, complete from basal groove to apex, the discal elytral intervals broadly, moderately convex; the eighth interval broadly carinate apically, extended dorsally above narrowed apex of interval 7, and convexly bulging laterally dorsad the subapical sinuation; lateral elytral setae 7 + 6; apical elytral seta present just laterad stria 2 as it nears stria 7, and subapical elytral seta present in stria 7 just laterad terminal fusion of striae 3 + 4. *Microsculpture* of vertex reduced, surface glossy, indistinct transverse mesh on neck; pronotal disc glossy, median base mostly glossy but shallow transverse mesh in parts between punctures; discal elytral intervals with a transverse mesh, the sculpticell breadth 2–4× length, mixed with transverse lines, the microsculpture resulting in a coppery reflection. *Coloration* of head dark rufous; antennomere 1 flavous, 2–3 rufoflavous, 4–11 rufobrunneous; pronotal disc rufopiceous, lateral marginal depression anterad lateral seta rufobrunneous, median base rufous; elytral disc rufopiceous, the sutural interval basally rufous and apically rufoflavous, lateral marginal depression rufobrunneous; femora rufoflavous with brunneous cast, tibiae rufobrunneous with smoky cast most developed in apical half.

Male genitalia. Aedeagal median lobe dorsoventrally expanded apically, with a slightly convex ventral expansion and blunt dorsal tooth (Fig. 18B); ostial canal straight, terminated just ventrad the blunt dorsal tooth; internal sac bearing a large, melanized flagellar plate (Fig. 18C), plate length 0.67× distance from parameral articulations to apical face, with dorsal plate surface bearing elongate gonopore opening; internal sac with broad ventral ostial microtrichial patch and small dorsal ostial microtrichial patch.

Holotype male (MNHN) labeled: French Polynesia: Tahiti Nui / Pito Hito el. 2000 m 2-VI- / 2006 lot 02 pyrethrin fog / 17°36.790'S, 149°27.842'W / E.M. Claridge // HOLOTYPE / Mecyclothorax / aano / J.K. Liebherr 2013 (black-bordered red label).

##### Etymology.

The species epithet aano means wide or extensive in Tahitian, the name signifying the very broad body characteristic of this species.

##### Distribution and habitat.

This species is known only from the holotype obtained in a pyrethrin fog sample of moss-covered vegetation at 2000 m on Pito Hiti.

#### 16. Mecyclothorax hamatus

Perrault, 1987: 425; 1988: 237

http://species-id.net/wiki/Mecyclothorax_hamatus

##### Identification.

This species is diagnosable within the species group by the broadly reflexed lateral margins of the pronotum and elytra, the explanate and laterally reflexed margins extending the lengths of both structures (Fig. 16C), and the well-defined and complete transverse pronotal impression. The elytral striae are finely incised, and minutely punctate in their basal third, the punctures at most causing a slight expansion of the striae. The eighth elytral interval is upraised and finely carinate dorsad the apical half of the posterior series of the lateral elytral setae, and is broader and more convexly upraised dorsad the subapical sinuation. The vertex bears shallow isodiametric microsculpture, the sculpticells more upraised near the pronotal margin. The pronotal disc bears a shallow, elongate transverse mesh mixed with transverse lines, and the discal elytral intervals are covered with a distinct mesh composed of isodiametric sculpticells in transverse rows and transverse sculpticells. The male aedeagal median lobe is gracile basally, and bears a large dorsal projection at its apex (Fig. 18D). The ostial canal is curved ventrally near its apical terminus. Setal formula 2122; standardized body length 5.9–7.1 mm.

##### Distribution and habitat.

Perrault’s (1988) summarized locality data restricted this species to the elevational range of 1000–1400 m on Mont Marau. This elevational range was also observed in 2006, when this species was repeatedly captured in Malaise traps set at 1170 and 1340 m, but not at lower elevations nor at the summit; 1493 m elevation. Collections of this species in Malaise traps indicate the beetles are good climbers, most likely active on vegetation during nighttime foraging.

#### 17. Mecyclothorax altiusculoides

Perrault, 1987: 425; 1988: 237

http://species-id.net/wiki/Mecyclothorax_altiusculoides

##### Identification.

Like *Mecyclothorax hamatus*, but with the anterior transverse impression of the pronotum shallow, broad, and indistinct medially. The pronotal and lateral elytral depressions are broadly reflexed throughout their length (Fig. 16D). The discal elytral striae 1–4 are distinctly punctate in their basal half, the punctures expanding the striae. The eighth elytral interval is upraised and finely carinate dorsad the apical half of the posterior series of the lateral elytral setae, becoming broader and more convexly upraised apically. The frons is glossy with indistinct isodiametric to transverse sculpticells, and the vertex including the neck impression is covered with a regular mesh of isodiametric sculpticells in transverse rows. The pronotal disc is covered with a shallow but regular transverse mesh resulting in a subiridescent sheen, and the discal elytral intervals bear an isodiametric mesh, the sculpticells partially arranged in transverse rows. The male aedeagal median lobe is gracile, and the apex is gently expanded ventrally, moreso dorsally in the shape of a blunt tooth (Fig. 18E). The ostial canal is curved ventrally at its apical terminus. Setal formula 2122; standardized body length 5.8–7.0 mm.

##### Distribution and habitat.

The type series (Perrault 1988) was composed of specimens variously recorded from localities ranging 1100–1400 m and 1000–1800 m elevation on Mont Aorai. Subsequent records include samples from decayed fronds of *Freycinetia* piled on the ground after trail clearing, and from beating live and dead fern fronds. These latter samples spanned 1255–1320 m elevation.

#### 18. Mecyclothorax tuberculatus

Perrault, 1988: 238

http://species-id.net/wiki/Mecyclothorax_tuberculatus

##### Identification.

This species is uniquely characterized by a large tuberculate projection that distorts and upraises the eighth elytral interval and seventh stria, dorsad and immediately anterad the subapical sinuation (Fig. 17A). The seventh stria is present fore and aft the tubercle, but absent on its highest portion. The eyes are less convex and smaller in diameter than those of other *Mecyclothorax altiusculus* group species; ocular ratio 1.45, ocular lobe ratio 0.77. The head is glossy, and the vertex is covered with shallow but regular isodiametric-mesh microsculpture. The pronotal disc is similarly glossy, but shallow transverse-mesh microsculpture is evident. The discal elytra intervals are covered with a distinct transverse mesh, mixed with transverse lines. In the single known specimen, partially compromised eclosion resulted in a right elytron with a shagreened surface. The withered right elytron bears two dorsal setae, the normally eclosed left elytron only the anterior dorsal seta. Setal formula 21(1–2)2; standardized body length 6.4 mm.

##### Distribution and habitat.

The single female specimen was collected near the summit of Mont Aorai at 1900 m elevation.

#### 19. Mecyclothorax paraltiusculus

Perrault, 1988: 235

http://species-id.net/wiki/Mecyclothorax_paraltiusculus

##### Identification.

This species is best diagnosed by the pronotal lateral depressions that are narrowly reflexed anterad the lateral seta, and the narrowly incised, distinctly punctate discal elytral striae (Fig. 17B). The lateral elytral depressions are only moderately broad, such that the distance between the first three lateral elytral setae of the anterior series and the elytral margin is less than the breadth of elytral interval 9 at the posterior end of that series. The eighth interval is finely carinate dorsad the middle of the posterior series of lateral elytral setae and more upraised and broadly rounded dorsad the subapical sinuation. The vertex of the head is covered with isodiametric-mesh microsculpture arranged in transverse rows. The pronotal disc is glossy medially, with an indistinct transverse mesh laterally and basally, the sculpticells 2–3× broad as long. The discal elytral intervals are lined with a distinct transverse mesh, sculpticell breadth 2–4× length, the microsculpture producing an iridescent silvery sheen. The male aedeagal median lobe is gracile, with a slightly expanded, angular apex (Fig. 18F). The ostial canal curves dorsally only slightly near its apical terminus. Setal formula 2122; standardized body length 7.0–7.2 mm.

##### Distribution and habitat.

This species is recorded from Mont Teatara, Tahiti Iti, between 900 and 1100 m elevation. Two male specimens collected in 2006 have associated ecological data; one was found in association with dead, fermenting *Freycinetia*, and the second was collected from the wet decayed cambial layer of a dead *Reynoldsia* plant. The dead *Reynoldsia* also housed numerous cillaeine Nitidulidae adults and larvae (C.P. Ewing pers. comm.).

#### 20. Mecyclothorax pseudaltiusculus

Perrault, 1988: 233

http://species-id.net/wiki/Mecyclothorax_pseudaltiusculus

##### Identification.

This is a gracile species with a narrow orbicular prothorax, MPW/PL 1.25 (Fig. 17C). The lateral marginal depression is of subequal breadth from the rounded hind angle to the lateral seta. The eighth interval is broadly bulging and subcarinate dorsad the subapical sinuation, but not at all elevated anteriorly. The subapical elytral seta is absent, resulting in a setal formula of 2121. The head is glossy with only indistinct sculpticells traceable near the frontal grooves. The pronotal disc is glossy, with micropunctures visible but no sculpticells. The discal elytral intervals are covered with a regular isodiametric mesh. The male aedeagal median lobe appears very similar to that of *Mecyclothorax paraltiusculus* (Figs 18F, G), but the apex extends less beyond the ostium, and the flattened apical face is less oblique (Fig. 18G). Standardized body length 5.5–5.6 mm.

##### Distribution and habitat.

This species’ known distribution ranges 1100–1400 m elevation on Mont Marau. One specimen was collected in a Malaise trap, indicating the well-developed climbing abilities, and a second was beaten from a mix of *Dicranopteris* and other ferns.

#### 21. Mecyclothorax altiusculus

(Britton)

http://species-id.net/wiki/Mecyclothorax_altiusculus

Thriscothorax altiusculus Britton, 1938: 104.
Mecyclothorax altiusculus , Britton 1948: 110; Perrault 1978b: 136; 1988: 232.

##### Identification.

Beetles of this species appear superficially similar to, and are distributed sympatrically with those of *Mecyclothorax altiusculoides* (Fig. 16D), but they exhibit pronotal lateral marginal depressions that are apically narrowed, and elytral lateral marginal depressions that are only narrowly reflexed (Fig. 17D). The discal elytral striae 1–6 are deeply and narrowly incised, and are lined with elongate punctures along their basal half, the punctures causing crenulations along the strial length. The eighth interval is finely carinate dorsad the posterior series of the lateral elytral setae, and gradually toward the elytral apex where the interval is broadly convex. The frons and vertex are covered with dense transverse lines that are progressively replaced by an isodiametric mesh near the pronotal juncture. The pronotal disc is glossy medially, with an elongate, partially confused transverse mesh laterally; and the discal elytral intervals are covered with isodiametric sculpticells arranged in transverse rows. The male aedeagal median lobe is very similar to those of *Mecyclothorax paraltiusculus* and *Mecyclothorax pseudaltiusculus* (Figs 18F, G), but has a slightly more slender shaft, and an apex with a more acute ventral tip, a flatter apical face, and a more ventrally curved ostial canal (Fig. 19A). Setal formula 2122; standardized body length 5.5–6.5 mm.

##### Distribution and habitat.

This species is known to occupy habitats on Mont Aorai from 1255 to 1940 m elevation. All associated ecological data place it in the axils of dead, decaying *Freycinetia* fronds, where is occurs microsympatrically with *Mecyclothorax altiusculoides*.

#### 22. Mecyclothorax ovalipennis

Perrault, 1988: 236

http://species-id.net/wiki/Mecyclothorax_ovalipennis

##### Identification.

This and the following species, *Mecyclothorax parovalipennis*, are intermediate in pronotal configuration for the *Mecyclothorax altiusculus* group in that the pronotal hind angle apex is rounded, but the pronotal margin anterad the angle is slightly sinuate thereby tightly defining the angle (Figs 20A, B). The elytra are ellipsoid, as in *Mecyclothorax jarrigei* (Fig. 16A), but the striae are deep and broad (Fig. 20A), with minute punctures basally that only slightly expand the strial breadth. The discal elytral intervals are slightly convex. The microsculpture of the frons is reduced, the surface glossy, though shallow transverse-mesh microsculpture is visible on the neck impression to the pronotum. The pronotal disc is also glossy, though an distinct transverse mesh is visible in the larger wrinkles of the surface. The discal elytral intervals bear an elongate transverse mesh mixed with transverse lines. The male aedeagal median lobe apex is a rounded knob that is slightly extended dorsally (Fig. 19B). The ostial canal is broad and sinuously curved downward at its apical terminus. Setal formula 2122; standardized body length 5.6–5.7 mm.

##### Distribution and habitat.

This species is known from between 1125 and 1400 m elevations on Mont Marau. It has been collected by beating dead tree fern fronds, rotten *Freycinetia* stalks, and by pyrethrin fogging moss-covered *Weinmannia* trees.

#### 23. Mecyclothorax parovalipennis

Perrault, 1988: 236

http://species-id.net/wiki/Mecyclothorax_parovalipennis

##### Identification.

Like *Mecyclothorax ovalipennis*, this species is characterized by rounded hind angles that are bordered anteriorly by a slightly sinuate lateral margin (Figs 20A, B). The discal elytral striae are basally punctate, the punctures elongate and laterally expanding the striae, and the discal elytral intervals are nearly flat, even depressed in association with the microsculpture over portions of the intervals (Fig. 20B). The microsculpture is more well developed than in *Mecyclothorax ovalipennis*, with: 1, the frons bearing patches of transverse-mesh microsculpture and the neck impression with well-developed isodiametric sculpticells; 2, the pronotal disc covered with a shallow but evident transverse mesh; and 3, the discal elytral intervals lined with distinct transverse microsculpture comprising areas ranging from slightly transverse sculpticells to areas of transverse lines. The male aedeagal median lobe apex is broader than that of *Mecyclothorax ovalipennis* (Fig. 19B, C), and the apical face is more flattened. Setal formula 2122; standardized body length 5.9 mm.

##### Distribution and habitat.

This species is known only from the two type specimens collected at 1900 m elevation near the summit of Mont Aorai.

#### 24. Mecyclothorax tihotii

Liebherr, 2012b: 74

http://species-id.net/wiki/Mecyclothorax_tihotii

##### Identification.

This species is placeable in the *Mecyclothorax altiusculus* group based on the convex pronotal lateral margin anterad the narrowly denticulate, glabrous hind angle (Fig. 20C). However it is unique among Tahitian species in the broadly downturned male aedeagal median lobe apex (Fig. 19D). Among this group’s species, a downturned lobe apex, though narrower, is observable in *Mecyclothorax bryobius*, *Mecyclothorax ballioides*, and *Mecyclothorax ferruginosus* (Figs 19E–G). The absence of the anterior supraorbital seta, and the resultant setal formula of 1121, is uncommonly observed in Tahitian *Mecyclothorax*, with the exceptions of the *Mecyclothorax globosus* group species, *Mecyclothorax vaifaufa* Perrault and *Mecyclothorax profondestriatus*. However their very different aedeagal configurations (Figs 40C, D) give cold comfort to any hypothesis proposing a close phylogenetic relationship. The vertex is glossy, with the neck bearing indistinct isodiametric sculpticells. The pronotal disc is also glossy, with an indistinct transverse mesh visible outside the area of reflected light. The discal elytral intervals are covered with well-developed transverse lines, with only occasional cross-connections resulting in a subiridescent surface. Standardized body length 4.1 mm.

##### Distribution and habitat.

This species was collected at 1100 m elevation on Mont Mauru in association with moss-covered *Myrsine* and *Metrosideros* plants.

#### 25. Mecyclothorax bryobioides

Perrault, 1987: 426; 1988: 239

http://species-id.net/wiki/Mecyclothorax_bryobioides

##### Identification.

This species is among the five *Mecyclothorax altiusculus* group species characterized by a basally narrowed pronotum with briefly sinuate lateral margins (Figs 20D, 21), though the narrowed pronotal base of this species – MPW/BPW = 1.72–1.81 (n = 5) – is similar only to that seen in *Mecyclothorax bryobius*. The elytra are ovate, with maximum breadth near midlength. The discal elytral striae are finely incised and punctate basally, and the discal intervals are slightly but broadly convex. The frons is glossy, and the neck bears a shallow mesh of transversely stretched microsculpture. The pronotal disc is covered with a shallow transverse mesh, visible outside areas of reflected light. The discal elytral intervals have regular transverse-mesh microsculpture, sculpticell breadth 2–3× length. The male aedeagal median lobe apex is dramatically downturned, with the ventral extension more elongate (Fig. 3H) than observed in the other group species exhibiting a ventral extension (Figs 19D–G). Setal formula 2121; standardized body length 4.5–5.3 mm.

##### Distribution and habitat.

This species has been found from 900–1165 m elevation on Mont Teatara, Tahiti Iti. Specimens have been obtained by beating dead and living fern fronds, within the axils of *Astelia* plants, and via pyrethrin fogging of horizontal, moss-covered *Weinmannia* logs.

#### 26. Mecyclothorax bryobius

(Britton)

http://species-id.net/wiki/Mecyclothorax_bryobius

Thriscothorax bryobius Britton, 1938: 106.
Mecyclothorax bryobius , Britton 1948: 110; Perrault 1978b: 135; 1988: 239.

##### Identification.

This species is similar to *Mecyclothorax bryobioides*, but is easily distinguished by the obovate elytra, the maximal breadth well behind midlength (Fig. 21A), and the more rounded pronotal hind angles. The pronotal base is similarly narrow; MPW/BPW = 1.78–1.89 (n = 3). The microsculpture is more developed than in *Mecyclothorax bryobioides*, with the frons and vertex covered with a shallow isodiametric mesh, the pronotal disc bearing a regular though shallow transverse mesh, and the discal elytral intervals lined with an elongate transverse mesh mixed with areas of transverse lines. The male aedeagal median lobe is downturned apically (Fig. 19E), but more broadly and much less extensively than in *Mecyclothorax bryobioides* (Fig. 3H). Setal formula 2121; standardized body length 4.8–5.2 mm.

##### Distribution and habitat.

This species’ known distribution is restricted to the highest elevations on Mont Aorai; 1650–2000 m. It has been collected by beating moss on trees and shrubs, indicating that the species possesses at least partially arboreal habits.

#### 27. Mecyclothorax ballioides

Perrault, 1978b: 151; 1988: 240

http://species-id.net/wiki/Mecyclothorax_ballioides

##### Identification.

Beetles of this species are similar to those of *Mecyclothorax bryobioides* (Fig. 20D), however the pronotum is less constricted basally – MPW/BPW = 1.60–1.73 (n = 5) – and the elytra are more broadly ovoid (Fig. 21B). The pronotal hind angles are sharply obtuse, with the pronotal lateral margins subparallel to convergent immediately anterad the angles. The pronotal margin is beaded both laterad and basad the hind angle, the bead broadest immediately inside the hind angle. The discal elytral striae are shallow and distinctly punctate basally, the punctures appearing isolated due to the shallow depth of the striae. The frons and vertex are glossy with indistinct transverse wrinkles. The pronotal disc bears indistinct transverse-mesh microsculpture, the sculpticell margins not visible in areas of reflected light, and the discal elytral intervals bear a distinct mesh composed of both isodiametric and transverse sculpticells, the sculpticells arranged in irregular transverse rows. The male aedeagal median lobe has an evenly arcuate shaft and a short apex with an abrupt ventral extension that has a tightly rounded tip (Fig. 19F). Setal formula 2121; standardized body size 4.0–4.4 mm.

##### Distribution and habitat.

This species has been collected from 1125–1490 m elevation on Mont Marau, always in association with ferns. These situations include beating dead *Cyathea* tree fern fronds, or collection onto sheets via application of pyrethrin fog to banks of dead or living low-stature ferns.

#### 28. Mecyclothorax ferruginosus

Perrault, 1987: 426; 1988: 241

http://species-id.net/wiki/Mecyclothorax_ferruginosus

##### Identification.

Befitting its name, this species is characterized by the palest cuticle observed in all Tahitian *Mecyclothorax* (Fig. 21C). The male aedeagus of one of the two known specimens does not appear particularly teneral (Fig. 19G), though the flavous cast suggests the individual had not become completely melanized when collected. Beyond the coloration, this species can be told by the transverse, basally constricted pronotum; MPW/PL = 1.33, MPW/BPW = 1.75. The pronotal hind angles are distinct, nearly right, and projected, with the pronotal lateral margins convergent immediately anterad the angles. The discal elytral striae are nearly smooth, and are lined with minute punctures that cause slight irregularities in the striae in their basal half. The head is glossy with patches of transverse mesh microsculpture in depressed wrinkles associated with the frontal grooves. The pronotal disc is covered with an indistinct elongate transverse mesh that results in slight iridescence. The discal elytral intervals are covered with a dense transverse-mesh microsculpture, sculpticell breadth 2–4× length. The male aedeagal median lobe apex has an elongate ventral extension (Fig. 19G), most similar, to though not as exaggerated as, that observed in *Mecyclothorax bryobioides* (Fig. 3H). Setal formula 2122; standardized body length 5.8–6.0 mm.

##### Distribution and habitat.

Georges Perrault collected the two known specimens together between 800 and 1000 m elevation on Mont Teatara, Tahiti Iti.

#### 29. Mecyclothorax sinuatus

Perrault, 1988: 240

http://species-id.net/wiki/Mecyclothorax_sinuatus

##### Identification.

Beetles of this species appear quite similar to those of the sympatrically distributed *Mecyclothorax ferruginosus*, however consistent differences in proportions and punctation demonstrate that they are distinct. In this species, the pronotum is less transverse, MPW/PL = 1.25, and less constricted basally, MPW/BPW = 1.59. The elytra are more narrowly ovoid (Fig. 21D), with the lateral margins more narrowly rounded laterad the angulate humeri. The discal elytral striae are more distinctly punctate, with the punctures in striae 1-3 expanding the strial breadths adjacent to the anterior dorsal elytral seta. The frons and vertex are covered with an indistinct transverse mesh, the pronotal disc surface is partially glossy, with areas of transverse-mesh microsculpture, and the discal elytral intervals are covered with transverse lines irregularly joined into an elongate mesh. Setal formula 2122; standardized body length 5.0–5.2 mm.

##### Distribution and habitat.

This species is known from two female specimens collected between 800 and 1000 m elevation on Mont Teatara, Tahiti Iti.

### 4. *Mecyclothorax dannieae* species group

**Figure 22.**
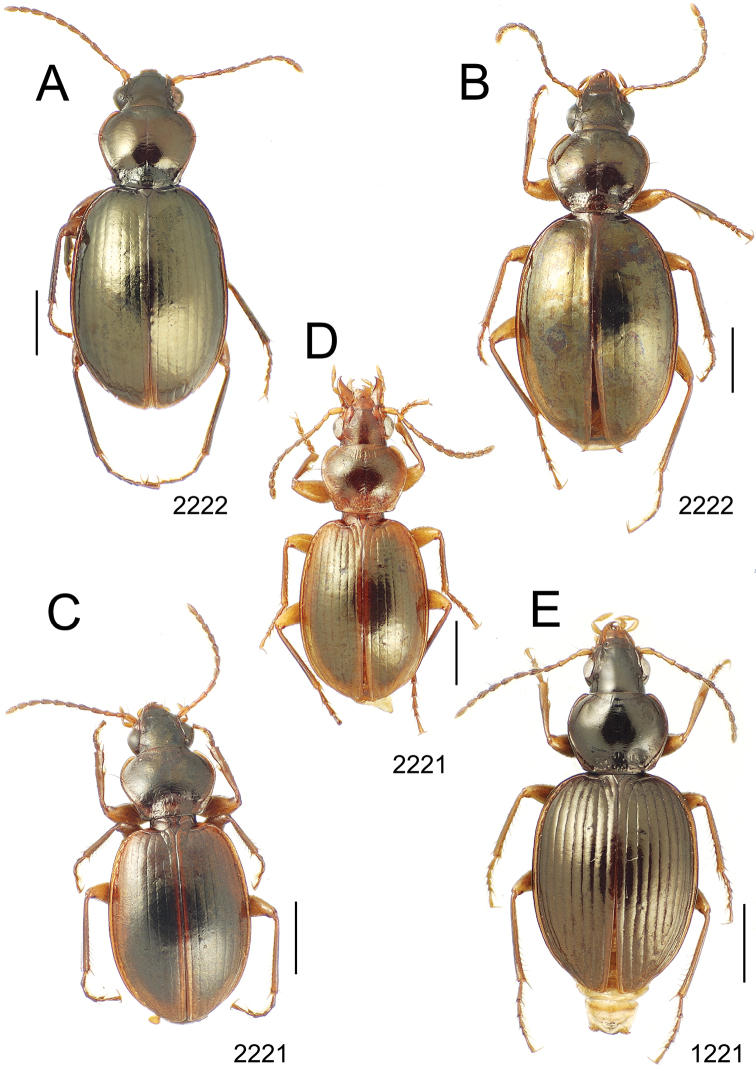
*Mecyclothorax* spp., dorsal view; scale bars 1.0 mm; setal formula (see Fig. 10) at lower right of each figure **A** *Mecyclothorax papau* holotype male **B** *Mecyclothorax manina* holotype female **C** *Mecyclothorax everardi* paratype male (CUIC) **D** *Mecyclothorax brittoni* paratype male (NHMB) **E** *Mecyclothorax ramagei* allotype female.

**Figure 23.**
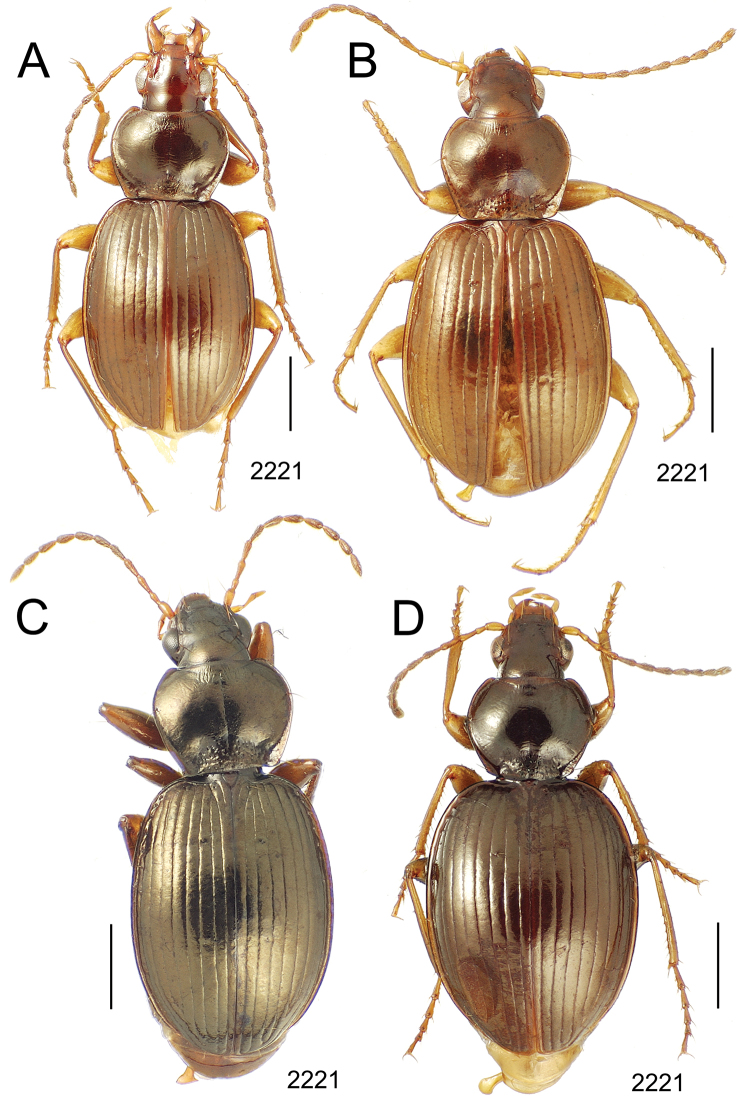
*Mecyclothorax* spp., dorsal view; scale bars 1.0 mm; setal formula (see Fig. 10) at lower right of each figure **A** *Mecyclothorax tahitiensis* holotype male, 1800 m el. **B** *Mecyclothorax tahitiensis* male (FMNH), 2100 m el. **C** *Mecyclothorax pitohitiensis* paratype male (CUIC) **D** *Mecyclothorax teatara*.

**Figure 24.**
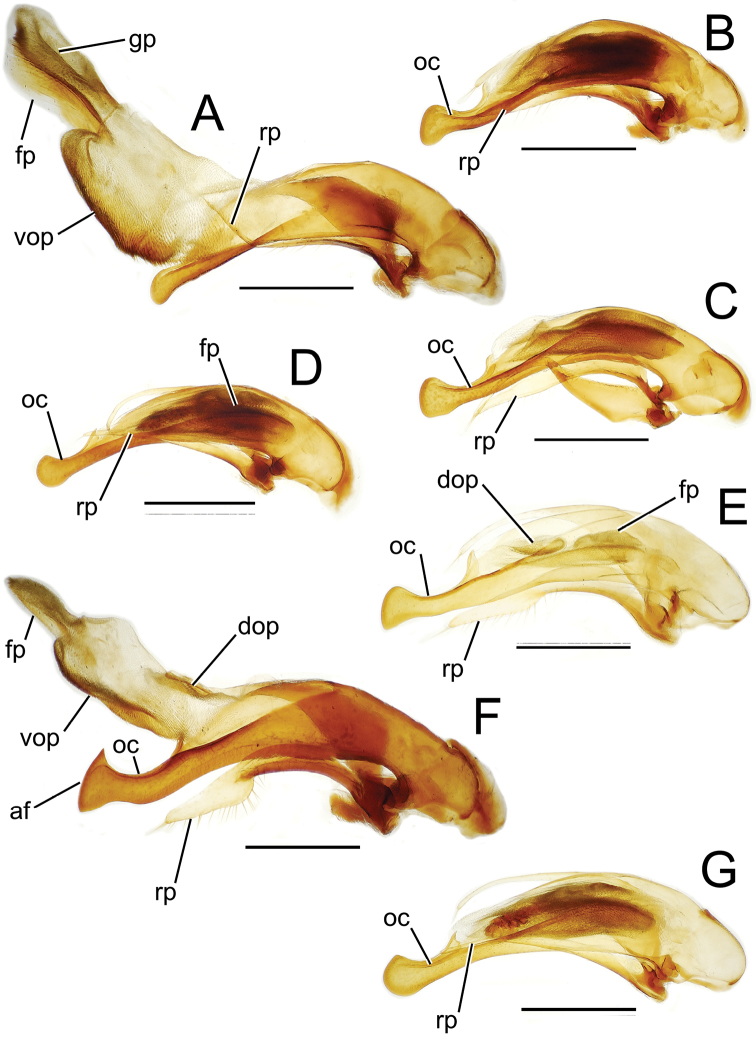
Male aedeagal median lobe and associated parameres, *Mecyclothorax* spp., right lateral view; scale bars 0.5 mm **A** *Mecyclothorax papau* holotype, internal sac everted **B** *Mecyclothorax everardi* paratype (CUIC) **C** *Mecyclothorax brittoni* paratype (MHNB) **D** *Mecyclothorax ramagei* holotype **E** *Mecyclothorax tahitiensis* **F** *Mecyclothorax pitohitiensis* paratype (CUIC), internal sac everted **G** *Mecyclothorax teatara* Abbreviations: **af** apical face **dop** dorsal ostial microtrichial patch **fp** flagellar plate **gp** gonopore **oc** ostial canal **rp** right paramere **vop** ventral ostial microtrichial patch.

**Figure 25.**
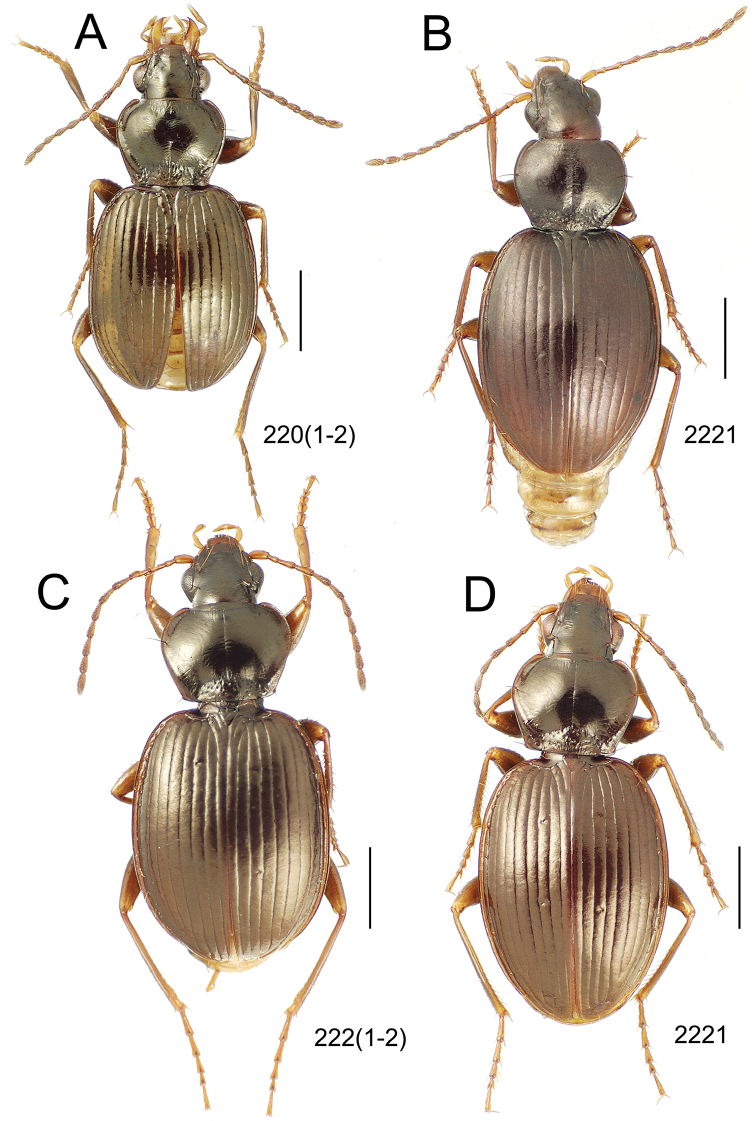
*Mecyclothorax* spp., dorsal view; scale bars 1.0 mm; setal formula (see Fig. 10) at lower right of each figure **A** *Mecyclothorax fairmairei* holotype male **B** *Mecyclothorax tutei* holotype female **C** *Mecyclothorax aorai* **D** *Mecyclothorax cooki*

**Figure 26.**
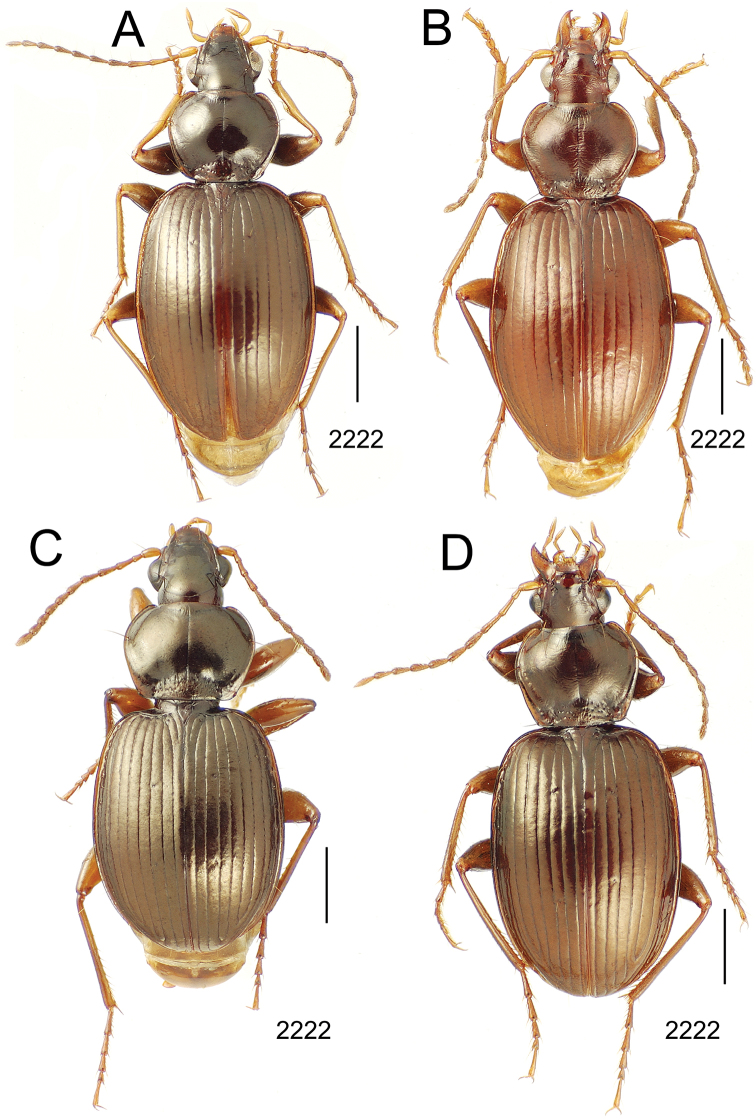
*Mecyclothorax* spp., dorsal view; scale bars 1.0 mm; setal formula (see Fig. 10) at lower right of each figure **A** *Mecyclothorax marau* **B** *Mecyclothorax dannieae* paratype male (MHNB) **C** *Mecyclothorax villiersi* **D** *Mecyclothorax negrei* paratype male (MNHN).

**Figure 27.**
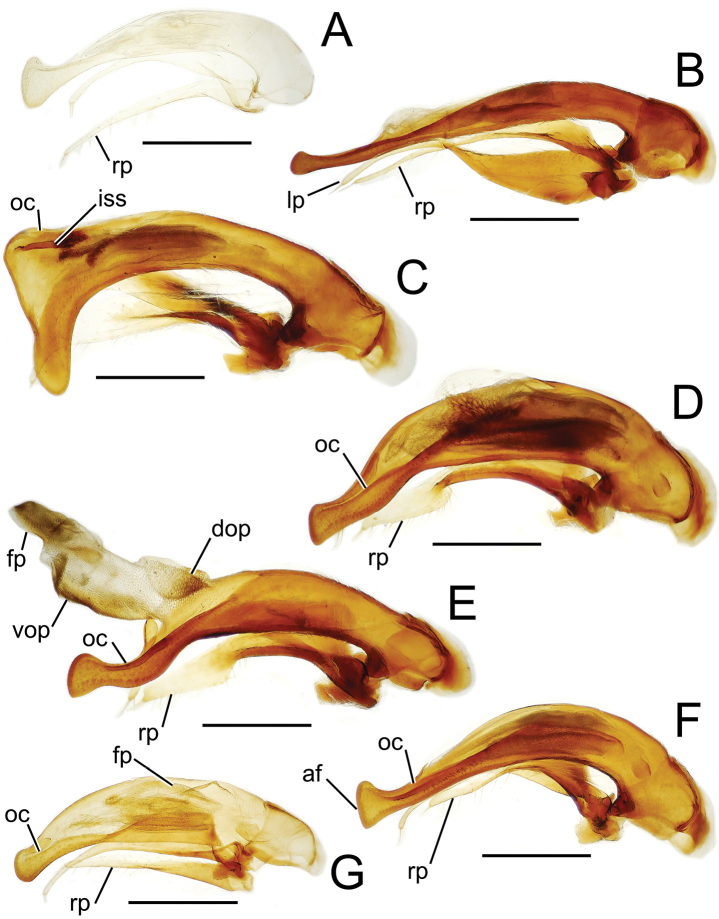
Male aedeagal median lobe and associated parameres, *Mecyclothorax* spp., right lateral view; scale bars 0.5 mm **A** *Mecyclothorax fairmairei* holotype, teneral **B** *Mecyclothorax aorai* **C** *Mecyclothorax marau* **D** *Mecyclothorax dannieae* paratype (MNHN) **E** *Mecyclothorax villiersi*, internal sac everted **F** *Mecyclothorax negrei* holotype **G** *Mecyclothorax curtisi* holotype Abbreviations: **af** apical face **dop** dorsal ostial microtrichial patch **fp** flagellar plate **iss** internal sac spicule **lp** left paramere **oc** ostial canal **rp** right paramere **vop** ventral ostial microtrichial patch.

**Diagnosis.** These species are characterized by a quadrisetose pronotum, with the lateral margins convex, straight, or only slightly sinuate before the hind angles (Figs 22, 23, 25, 26). The elytral striae are smooth (Figs 23A, B) to distinctly punctate (Fig. 25A), and the elytral intervals are nearly flat to moderately convex. Within this group are four species that exhibit a flattened lamellar apex on the right paramere (Figs 24E, F, 27D, E). Standardized body lengths 4.7–6.7 mm.

#### Identification key to Tahitian species of the *Mecyclothorax dannieae* species group

**Table:** 

1	Elytral striae superficial, shallow to obsolete, smooth to indistinctly punctate, elytral intervals flat; if intervals appear slightly convex, elytral margins contrastedly paler than elytral center	2
–	Elytral striae deeper, elytral intervals moderately convex to convex, elytral center and lateral margin concolorous though marginal depression may be narrowly paler	5
2	Center of each elytron brunneous to piceous, the sutural interval and lateral margin broadly and contrastedly paler, rufoflavous; elytral striae 1–7 evident though stria 7 may be shallower	4
–	Entire elytral surface concolorous, dark rufobrunneous, only the lateral marginal depression and sutural interval paler, the margin rufoflavous and sutural interval dark rufous; elytral striae extremely shallow, finely incised	3
3	Pronotum narrow, MPW/PL = 1.16 (Fig. 22A), lateral marginal depression and disc concolorous, dark rufous; anterior transverse impression lined with marked longitudinal strigae; elytral striae distinct, minutely punctate (Pito Hiti)	30. *Mecyclothorax papau* sp. n.
–	Pronotum transverse, MPW/PL = 1.31 (Fig. 22B), lateral marginal depression translucent, rufoflavous, contrasted to rufobrunneous disc; anterior transverse impression shallow, finely incised, smooth across breadth; elytral striae shallow, obsolete (Pito Hiti)	31. *Mecyclothorax manina* sp. n.
4	Elytral striae smooth basally, impunctate though with slight irregularities along length (Fig. 22C); elytral disc with shallow transverse-mesh microsculpture that is not visible in areas of reflected light; apex of male aedeagal median lobe expanded both ventrally and dorsally (Pito Hiti)	32. *Mecyclothorax everardi* sp. n.
–	Elytral striae indistinctly punctate in basal portion of disc (Fig. 22D); elytral disc with distinct, regular transverse-mesh microsculpture visible across field of view; apex of male aedeagal median lobe expanded dorsally, the ventral surface straight (Aorai)	33. *Mecyclothorax brittoni* Perrault
5	Elytra with only the apical seta present	6
–	Elytra with two setae apically, the apical seta near the apex of stria 2, and the subapical seta associated more basally with stria 7	13
6	Pronotum distinctly transverse, MPW/PL = 1.25–1.33	7
–	Pronotum little transverse, MPW/PL = 1.17–1.25	9
7	Two supraorbital setae over each eye; standardized body length 5.3–5.8 mm	8
–	A single, posterior supraorbital seta over each eye; standardized body length 4.6–4.8 mm	34. *Mecyclothorax ramagei* sp. n.
8	Elytra subquadrate, broad basally, MEW/HuW = 2.04 (Figs 23A, B); pronotal lateral margin explanate outside laterobasal depression, lateral marginal depression of pronotum indistinctly broadened as it joins deepest portion of depression; male aedeagal median lobe apex with rounded dorsal and ventral expansions (Fig. 24D) (Aorai)	35. *Mecyclothorax tahitiensis* Perrault
–	Elytra narrowed basally, lateral margins narrowly rounded posterad angulate humeri, MEW/HuW = 2.10–2.20 (Fig. 23C); pronotal lateral margin explanate and distinctly elevated outside laterobasal depression, lateral marginal depression of pronotum sinuously traceable to deepest portion of depression; male aedeagal median lobe apex with angulate dorsal and ventral projections (Fig. 24E) (Pito Hiti)	36. *Mecyclothorax pitohitiensis* sp. n.
9	Pronotal margins linearly narrowed anterad hind angles (Fig. 25), the angles well developed and blunt; MPW/BPW = 1.39–1.42	10
–	Pronotal lateral margins more broadly explanate anterad the obtuse-rounded hind angles (Fig. 23D), pronotal base (defined by basal setal positions) very narrow, MPW/BPW = 1.46–1.53 (Teatara)	37. *Mecyclothorax teatara* Perrault
10	Elytral microsculpture consisting of transverse mesh with a tendency to form transverse rows; two dorsal elytral setae in the third interval	11
–	Elytral microsculpture consisting of transverse lines without tendency to form a distinct mesh; dorsal elytral setae absent (Marau)	38. *Mecyclothorax fairmairei* Perrault
11	Pronotal disc with distinct transverse microsculpture, the sculpticell margins distinct, resulting in a gratelike surface reflection; pronotal lateral marginal depression moderately broad (Figs 25C, D), concavely bordering pronotal disc, pronotal margin upturned	12
–	Pronotal disc glossy, with indistinct elongate transverse microsculpture, the sculpticells not visible in areas of reflected light; pronotal lateral marginal depression very narrow laterally (Fig. 25B), the margin narrowly beaded along margin of convex disc (Mauru)	39. *Mecyclothorax tutei* Liebherr
12	Elytra subquadrate, lateral margins extended laterally outside humeral angle, lateral marginal depression moderately broad, edge upraised and translucent near anterior series of lateral elytral setae (Fig. 25C); discal elytral intervals little convex medially; transverse elytral microsculpture fine, sculpticells arranged in narrow rows; (Aorai)	40. *Mecyclothorax aorai* Perrault
–	Elytra ovoid, lateral margins curved posterad outside humeral angle, lateral marginal depression narrower, edge little upraised and not translucent near anterior series of lateral elytral setae (Fig. 25D); elytral intervals more convex, surface rounded medially; transverse elytral microsculpture distinct, the mesh broader, some isodiametric sculpticells present (Aorai)	41. *Mecyclothorax cooki* Perrault
13	Pronotal lateral margins straight or concave before well-developed obtuse hind angles; elytral microsculpture distinct; parascutellar striole completely indicated	14
–	Pronotum lateral margins convex before scarcely indicated, blunt hind angles, the laterobasal seta producing a small projection, the pronotal base narrow, MPW/BPW = 1.39 (Fig. 26A); elytral microsculpture feeble, the mesh broad; parascutellar striole and elytral basal groove superficial (Marau)	42. *Mecyclothorax marau* Perrault
14	Elytral humerus rounded (Figs 26C, D); elytral microsculpture consisting of dense, distinct transverse lines with the tendency to form a mesh	15
–	Elytral humerus angulate, the elytral margin raised as a distinct ridge (Fig. 26B); elytral microsculpture consisting of a transverse mesh (Aorai)	43. *Mecyclothorax dannieae* Perrault
15	Pronotal lateral margins straight anterad blunt, obtuse hind angles (Figs 25A, 26D)	16
–	Pronotal lateral margins sinuate anterad the distinct hind angles (Fig. 26C); elytral lateral margin raised as a distinct ridge at humerus (Marau)	4. *Mecyclothorax villiersi* Perrault
16	Body size larger, standardized body length 5.6–6.4 mm; two dorsal elytral setae in third interval	17
–	Body size smaller, standardized body length 4.3 mm; dorsal elytral setae absent (Marau)	38. *Mecyclothorax fairmairei* Perrault
17	Pronotum narrower basally, MPW/BPW = 1.37–1.41, lateral margin convex anterad obtuse-rounded hind angle (Fig. 26D) (Aorai)	45. *Mecyclothorax negrei* Perrault
–	Pronotum broader basally, MPW/BPW = 1.32, lateral margin slightly sinuate anterad obtuse hind angle, concavity of margin defined by jag at basal setal articulation (Fig. 25C) (Aorai)	40. *Mecyclothorax aorai* Perrault

#### 30. Mecyclothorax papau sp. n.

http://zoobank.org/6C1E4CAB-A7FF-4C7C-855B-305F16E0A6C9

http://species-id.net/wiki/Mecyclothorax_papau

##### Diagnosis.

Of the four species in this group with shallow elytral striae (Figs 22A–D), this species is characterized by the most quadrate pronotum; MPW/PL = 1.16–1.20 (N = 2). The pronotal base is relative broad; MPW/BPW = 1.42. This species also exhibits the darkest, most metallic dorsal body coloration. This comprises a forebody that is a rich rufobrunneous with melanized regions near internal apodemes – clypeus, frontoclypeal suture, and pronotal anterior transverse impression and median base – and rufopiceous elytra with narrowly paler sutural intervals and narrowly rufobrunneous lateral marginal depressions. The upraised elytral microsculpture, a mixture of isodiametric and slightly transverse sculpticells, results in a silvery metallic reflection. Setal formula 2222; standardized body length 5.5–5.9 mm. *Head* with frontal grooves sinuously canaliculated, slightly convergent toward frontoclypeal suture, with distinct transverse wrinkles radiating onto frons from fine carina mesad anterior supraorbital seta; eyes convex, ocular ratio 1.58–1.62, ocular lobe protruded from gena, a fine shallow groove at juncture; antennae filiform, antennomere 8 length 2.0× maximal breadth. *Pronotum* with obtuse, setose hind angles, the flattened area surrounding the setal articulatory socket protruded as a jag that comprises angle; median base smooth near midline, convexly raised above basal margin and depressed relative to convex disc, juncture with disc lined with 10–12 fine, short longitudinal wrinkles each side; anterior transverse impression bordered anteriorly by a raised margin, crossed by about eight fine longitudinal strigae each side. *Elytra* moderately convex, the elytral striae very shallow and indistinctly punctate, the striae so shallow that punctures are intermittently isolated even on disc, intervals only slightly convex; humeri obtuse angulate, the lateral marginal depression narrow throughout length; eighth interval convexly raised, subcarinate only near elytral apex, coplanar with other intervals basad subapical sinuation; lateral elytral setae 7 + (5–6). *Microsculpture* of frons and vertex a well-developed transverse mesh; pronotal disc covered with a shallow transverse mesh, the sculpticells visible only outside areas of reflected light. *Coloration* of body dorsum somber; antennomeres 1–2 rufoflavous, 3–11 rufobrunneous; femora flavous except for apices with rufoflavous cast, contrasted with partially melanized, rufobrunneous ventral sclerites; tibiae and tarsi rufopiceous.

Male genitalia. Aedeagal median lobe robust, shaft broad; apex moderately extended beyond ostium, tip gently downturned (Fig. 24A); right paramere narrow in apical half of length, extended as a whip-like structure; internal sac with broad ventral expansion that is covered with a well-developed ventral ostial microtichial patch; flagellar plate very elongate, length 0.70× distance from parameral articulations to apex.

Holotype male (MNHN) labeled: French Polynesia: Tahiti Nui / Pito Hiti el. 2080 m 2-VI- / 2006 lot 03 pyrethrin fog / 17°36.806'S, 149°27.842'W / E. M. Claridge // HOLOTYPE / Mecyclothorax / papau / J.K. Liebherr 2013 (black-bordered red label).

Paratype: Tahiti Nui, Pito Hiti, 2090 m el., 17°36.790'S, 149°27.842'W, 2-vi-2006 lot 02, Claridge, pyrethrin fog (EMEC, 1 teneral female).

##### Etymology.

The species epithet papau is the Tahitian adjective for shallow, as well as the noun for shallows. The name signifies the very shallow elytral striae observed in individuals of this species.

##### Distribution and habitat.

The two specimens were collected in pyrethrin fog samples of moss-covered vegetation 20–30 m elevation below the summit of Pito Hiti.

#### 31. Mecyclothorax manina sp. n.

http://zoobank.org/078F2FF5-C228-485C-98EA-C182B6E81B31

http://species-id.net/wiki/Mecyclothorax_manina

##### Diagnosis.

Like *Mecyclothorax papau* in the very shallow elytral striae and rufopiceous elytra with a metallic reflection, but with more narrowly ellipsoid elytra, and a more transverse and more basally constricted pronotum, MPW/PL = 1.31, MPW/BPW = 1.47 (Fig. 22B). The head and pronotum are concolorous, dark rufobrunneous, without fields of melanized cuticle as in *Mecyclothorax papau*. Setal formula 2222; standardized body length 6.0 mm. *Head* with sinuously canaliculated frontal grooves, the frons with a few indistinct wrinkles on the mesal slopes of the grooves; eyes moderately convex, ocular ratio 1.57, the ocular lobe protruded and margined behind by a distinct groove, ocular lobe ratio 0.88; antennae filiform, antennomere 8 length 2.25× maximal breadth. *Pronotum* with rounded hind angles, the basal seta producing a jag in the margin that is 0.10× the pronotal length anterad the basal margin; median base depressed relative to convex disc, 8–11 fine punctures each side, punctures smaller and more isolated medially, 5–8 elongate punctures along margin with disc; anterior transverse impression broad and shallow but complete medially, finely incised anteriorly only in lateral half of each side; lateral marginal depression of even breadth in anterior half of pronotum, not broader at front angle. *Elytra* narrowly rounded basally, the proximate humeri angled; striae 1–6 shallow and partially discontinuous on disc, punctures best developed on striae 1 and 2, stria 7 visible only as a discontinuous series of elongate depressions; eighth interval only slightly upraised laterad stria 7 from above subapical sinuation to apex; lateral elytral setae 7 + (5–6). *Microsculpture* of frons and vertex a shallow transverse mesh. Pronotal disc with an elongate transverse mesh visible outside areas of reflected light, the sculpticell breadth 4× length, and discal elytral intervals with a distinct, regular transverse mesh, sculpticell breadth 2–3× length. *Coloration* of antennomere 1 flavous, antennomeres 2–7 or 8 rufobrunneous, and antennomeres 8 or 9–11 rufoflavous; ventral body sclerites rufobrunneous, contrasted to dorsally flavous elytral epipleuron and flavous femora; tibiae and tarsi rufobrunneous.

Female reproductive tract. The female holotype was not dissected, but the gonocoxae are exerted and therefore visible. Basal gonocoxite 1 with apical fringe of 2–3 setae laterally, and 4–5 smaller setae along medial margin including apex; apical gonocoxite 2 with two lateral ensiform setae, the apical lateral seta stouter but of the same length as the basal seta, and a single dorsal ensiform seta as broad as the apical seta; apical sensory furrow bearing two nematiform setae.

Holotype female (MNHN) labeled: French Polynesia: Tahiti Nui / Pito Hiti el. 2110 m 2-VI- / 2006 lot 04 pyrethrin fog / 17°36.860'S, 149°27.837'W / E. M. Claridge // HOLOTYPE / Mecyclothorax / manina / J.K. Liebherr 2013 (black-bordered red label).

##### Etymology.

The species epithet is the Tahitian word mānina, meaning smooth, polished, or unwrinkled, signifying the smooth polished elytra observed in the holotype.

##### Distribution and habitat.

This was the sole specimen collected on the actual summit of Pito Hiti, 2110 m elevation. It was found via pyrethrin fogging of moss-covered vegetation. Only 30 m lower in elevation, the same collecting technique resulted in specimens of *Mecyclothorax papau*, *Mecyclothorax everardi*, *Mecyclothorax pitohitiensis*, *Mecyclothorax hoeahiti* sp. n., and *Mecyclothorax paraglobosus* Perrault.

#### 32. Mecyclothorax everardi sp. n.

http://zoobank.org/6FB9A92E-0819-4BE8-86C5-744E27BE54AA

http://species-id.net/wiki/Mecyclothorax_everardi

##### Diagnosis.

Among the *Mecyclothorax dannieae* group, this species shares the moderately impressed elytral striae and paler body margins observed in beetles comprising *Mecyclothorax brittoni* (Figs 22C, D), however the discal elytral striae 2–4 are at most irregularly impressed in their basal half, not punctate. This species exhibits a smaller standardized body length – 4.7–5.3 mm – than the preceding *Mecyclothorax papau* and *Mecyclothorax manina*. Setal formula 2222. *Head* with very fine, sinuous frontal grooves, their mesal slopes very shallowly wrinkled; eyes convex, ocular ratio 1.50–1.59 (n = 5), ocular lobe protruded with broad shallow groove at genal juncture; antennae shorter, submoniliform, antennomere 8 length 1.8× maximal breadth. *Pronotum* transverse, MPW/PL = 1.22–1.31, moderately constricted basally, MPW/BPW = 1.39–1.46; basal setal articulation in expanded margin resulting in small jag, the jag anterad slightly convex basal margin and so seta appears to be located at hind angle; median base moderately depressed relative to disc, irregularly punctured but with distinct longitudinal strigae lining margin with disc; anterior marginal impression shallow, broad, but crossed by 6–7 indistinct longitudinal wrinkles each side of midline; lateral marginal depression narrow, margin upraised near midlength, slightly broader and margin less upraised at front angle. *Elytra* broadly rounded basally, slightly obovate with greatest width behind midlength; humeri tightly rounded with basal groove distinctly recurved between the bases of striae 1–4; striae 1–6 broad, shallow on disc, irregularly impressed along length, stria 7 shallower basally; interval 8 only slightly elevated laterad apex of stria 7, its surface of same convexity as interval mesad stria 7; lateral elytral setae 7 + 6. *Metathoracic wings* reduced to vestigial flaps that do not extend beyond posterior margin of metanotum. *Microsculpture* of frons and vertex a distinct transverse mesh, sculpticell breadth 2× length, neck with isodiametric mesh; pronotal disc with well-developed microsculpture, a mixture of isodiametric and transverse sculpticells in transverse rows; discal elytral intervals with a regular, shallowly margined transverse mesh, sculpticell breath 2× length, the sculpticells shiny in reflected light. *Coloration* of frons and vertex rufobrunneous with metallic reflection; antennomeres 1–3 rufoflavous, 4–11 with brunneous cast; pronotal disc rufobrunneous with silvery metallic reflection, lateral margins, base and anterior callosity rufoflavous; elytral disc with intervals 2–6 dark brunneous with a metallic reflection, intervals 7–9 rufoflavous, sutural stria rufobrunneous basally, rufoflavous near apex; femora flavous, tibiae rufoflavous.

Male genitalia. Aedeagal median lobe shaft moderately slender, the apex beyond the ostium recurved first ventrally then dorsally, the apex expanded dorsoventrally, moreso ventrally, and flattened as an apical face (Fig. 24B); right paramere moderately broad, parallel sided nearly all the way to the tip; ostial canal on right face of median lobe and evenly curved in parallel with dorsal surface.

Female reproductive tract. Bursa copulatrix curved and folded to the right apically, length about 2× maximal breadth compressed on microslide (Fig. 14B); basal gonocoxite 1 broad, with apical fringe of four setae each side, approximately 10 small setae on mesal half of coxite; apical gonocoxite 2 broad basally, lateral margin broadly arcuate (Fig. 8D), two lateral ensiform setae with maximal breadth medially along length, one dorsal ensiform seta, and apical sensory furrow with two nematiform setae and two furrow pegs.

Holotype male (MNHN) labeled: French Polynesia: Tahiti Nui / Pito Hiti el. 2070 m 2-VI- / 2006 lot 01 pyrethrin fog / 17°36.813'S, 149°27.842'W / E. M. Claridge // HOLOTYPE / Mecyclothorax / everardi / J.K. Liebherr 2013 (black-bordered red label).

Allotype female (MNHN) labeled as holotype.

Paratypes: same data as holotype (CUIC, 2; EMEC, 3); Tahiti Nui, Pito Hiti, 2000 m el., 17°36.790'S, 149°27.842'W, 2-vi-2006 lot 02, Claridge, pyrethrin fog (CUIC, 3; EMEC, 8; MNHN, 2; NMNH, 2), 2080 m el., 17°36.806'S, 149°27.842'W, 2-vi-2006, lot 03, Claridge, pyrethrin fog (CUIC, 2; EMEC, 2).

##### Etymology.

Based on synapomorphies of the body coloration and male genitalia, this species is hypothesized to represent the adelphotaxon of *Mecyclothorax brittoni*. As such, the species epithet everardi places E.B. Britton’s first name adjacent to his last name in any phylogenetic classification of *Mecyclothorax*. The species epithet honors Dr. Britton’s many efforts in the study of Pacific *Mecyclothorax* beetles, as well as his subsequent illustrious career at C.S.I.R.O., Canberra.

##### Distribution and habitat.

All specimens have been collected from 2000 to 2080 m elevation on Pito Hiti via pyrethrin fogging of moss-covered vegetation.

#### 33. Mecyclothorax brittoni

Perrault, 1978b: 141; 1986: 447

http://species-id.net/wiki/Mecyclothorax_brittoni

##### Identification.

Individuals of this species share the pale elytral margins and sutural interval surrounding piceous elytral discs with *Mecyclothorax everardi* (Figs 22C, D), but the elytral humeri are closer together and the elytral base is more rounded laterad the humeral angles. The microsculpture is more well developed, with the head covered with upraised, transversely stretched sculpticells resulting in a shagreened appearance. The pronotum has a well-developed isodiametric mesh anteriorly amongst the longitudinal wrinkles crossing the anterior transverse impression. The pronotal disc is covered with an evident transverse mesh, and the discal elytral intervals bear a well-developed transverse mesh, sculpticell breadth 2–3× length. The male aedeagus (Fig. 24C) has an expanded median lobe apex, though the apex is less curved dorsally than observed in males of *Mecyclothorax everardi* (Fig. 24B). The right paramere is moderately broad and parallel sided nearly all the way to the pointed parameral tip. The ostial canal runs parallel to the dorsal margin of the aedeagus apicad the ostium on the right face of the median lobe, curving ventrally just before its apical terminus. Setal formula 2221; standardized body length 4.8–5.2 mm.

##### Distribution and habitat.

This species is known from two male specimens collected at 1900 m elevation near the summit of Mont Aorai.

#### 34. Mecyclothorax ramagei sp. n.

http://zoobank.org/44B7FBDD-64CC-4C02-A9CD-16A8AB2607F4

http://species-id.net/wiki/Mecyclothorax_ramagei

##### Diagnosis.

Beetles of this species (Fig. 22E) look like smaller-sized versions of those comprising the sympatric *Mecyclothorax teatara* (Fig. 23D), but the presence of only the posterior supraorbital seta diagnoses this species from all others in the *Mecyclothorax dannieae* group; setal formula 1221; standardized body length 4.6–4.8 mm. The pronotum is more transverse than that of *Mecyclothorax teatara*; MPW/PL = 1.27–1.30 (n = 2) in this species versus MPW/PL = 1.25 (n = 2) in *Mecyclothorax teatara*. *Head* with frontal grooves subparallel, shallow behind with small transverse wrinkles emanating onto frons, broad and deep anteriorly near the frontoclypeal suture; eyes moderately convex on little protruded ocular lobes, ocular ratio 1.44–1.51, ocular lobe ratio 0.83–0.86, a broad shallow groove at juncture of gena and hind portion of lobe; antennae moderately elongate, antennomere 8 length 1.80× maximal breadth. *Pronotum* transverse, lateral margins broadly convex from tightly rounded, little protruded front angles to obtuse-rounded hind angles; basal pronotal setal articulation causing jag in margin and a very brief sinuation anterad the seta; median base moderately depressed relative to disc, very smooth, with 6–9 sparsely distributed, shallow punctures each side, margin of base and disc lined with elongate punctures and fine longitudinal wrinkles; basal pronotal margin broadly convex between the hind angles; anterior transverse impression complete medially, anterior margin upraised at anterior callosity, impression crossed by 3–7 longitudinal wrinkles each side in median 2/4s, the wrinkles variously continued across the anterior callosity; apical width noticeably less than width across hind angles, APW/BPW = 0.81–0.85; lateral marginal depression moderately narrow, the margin upturned, depression not wider inside front angles; laterobasal depression very smooth, broad, broadly margined laterally and basally, evenly upraised anteriorly to meet disc. *Elytra* broadly ovate, basal groove evenly curved, humerus tightly rounded, and lateral margin broadly extended posterad humerus; disc flat between the third intervals each side, sides moderately sloped to lateral marginal depression; discal elytral striae 1–5 moderately deep, smooth, the associated intervals broadly, slightly convex, striae 6–7 slightly shallower, minutely punctate; apex with striae 1–6 moderately deep, stria 7 deeper and broader apicad subapical sinuation, interval 8 no more convex than inner intervals; lateral elytral setae 7(8) + 6. *Microsculpture* of vertex a shallow, transverse mesh, sculpticell breadth 2–4× length; pronotal disc with elongate transverse mesh, sculpticell breadth 3–5× length, intermixed with transverse lines; pronotal median base with evident transverse mesh, breadth 2× length, on the flat cuticle among the punctures; elytral disc with distinct transverse mesh, sculpticells 2× broad as long, plus transverse lines. *Coloration* of head rufous with a piceous cast, clypeus rufous, labrum rufoflavous; antennomere 1 flavous, 2–4 rufoflavous; 5–11 rufobrunneous; pronotal disc rufobrunneous, median base and anterior callosity concolorous, lateral marginal depressions slightly paler; elytral disc rufous with a silvery metallic reflection, apex narrowly flavous, lateral marginal depression rufoflavous outside the lateral elytral setal series; femora rufoflavous with subtle smoky cast medially, tibiae rufoflavous with brunneous cast.

Male genitalia. Aedeagal median lobe broad in basal half, evenly narrowed to apex of ostium, lobe apex narrowly extended beyond ostium, expanded into a narrowly spatulate tip with blunt dorsal expansion (Fig. 24D); ostial canal extended from ostium along dorsal margin of lobe’s right side, terminated ventrad blunt dorsal expansion; flagellar plate relatively short, length 0.4× distance from parameral articulations to apical face; right paramere narrowed apically.

Female gonocoxae. The female allotype was not dissected, but examining the distended abdomen (Fig. 22E) allows the following observations: basal gonocoxite 1 broad (dimensions similar to those in Fig. 8E), with 2–3 apical fringe setae laterally and one apical seta near medioapical angle; 7–11 setae arrayed from apex to base of mesal half of gonocoxite ventral surface; apical gonocoxite 2 narrow basally, subtriangular, with two lateral ensiform seta, the basal seta longer and narrower at base, and one dorsal ensiform seta; apical sensory furrow with two apical nematiform setae.

Holotype male (MNHN) labeled: FRENCH POLYNESIA: Tahiti Iti / Mont Atara, 815 m el. / 17°47.4425'S, 149°14.8103'W / 20-ix-2012, T. Ramage, Winkler / extraction of mossmat siftate // PF 1135 // HOLOTYPE / Mecyclothorax / ramagei / J.K. Liebherr 2013 (black-bordered red label).

Allotype female (MNHN) labeled as holotype // ALLOTYPE / Mecyclothorax / ramagei / J.K. Liebherr 2013 (black-bordered red label).

##### Etymology.

The species epithet is a patronym honoring the collector, Thibault Ramage, entomological consultant, Concarneau, France.

##### Distribution and habitat.

This species is distributed on the Tahiti Iti volcano. The type series was obtained by sifting mossmats on tree trunks, and extracting the insects from the siftate using a Winkler extractor (T. Ramage pers. comm.).

#### 35. Mecyclothorax tahitiensis

Perrault, 1978b: 140; 1986: 448

http://species-id.net/wiki/Mecyclothorax_tahitiensis

##### Identification.

Among the larger-bodied species of the *Mecyclothorax dannieae* group – standardized body length 5.4–5.6 mm – characterized by a transverse pronotum (Figs 23A, B), this species is characterized by the presence of only one apical elytral seta; setal formula 2221. The seta is present apicad the juncture of striae 3 + 4, and as detailed in the Setation section above, represents the apical seta of the plesiomorphic apical plus subapical elytral setal pair, its position evolutionarily transformed to a more basal position. The head is covered with evident microsculpture, the sculpticells transversely stretched and in transverse rows on the frons, more isodiametric on the neck. The pronotal disc and discal elytral intervals bear well-developed transverse mesh microsculpture, with sculpticell breadth 2–3× length. The male aedeagal median lobe (Fig. 24E) is apically expanded, much like that observed in *Mecyclothorax brittoni* (Figs 24C), but the apical portion is longer, and the tip flares out ventrally to complement the more broadly rounded dorsal expansion. The male right paramere is apically lamellate, the ventral surface lined with setae (Fig. 24E). The internal sac bears a dorsal ostial microtrichial patch.

##### Distribution and habitat.

This species was previously known from two male specimens Perrault (1986) collected in 1976 at 1750 m elevation on Mont Aorai. A third specimen, also a male, was collected from leaf litter, 17–20 June 1949 at 2042 m elevation on Aorai (FMNH); i.e., 24 m in elevation below the summit.

#### 36. Mecyclothorax pitohitiensis sp. n.

http://zoobank.org/C9C1CF8C-2F91-45BD-9BD8-76FA38E0F74B

http://species-id.net/wiki/Mecyclothorax_pitohitiensis

##### Diagnosis.

Among species of the *Mecyclothorax dannieae* group with well-impressed elytral striae and setal formula 2221 (Figs 23, 25B–D), this species can be diagnosed by the pronotum with explanate lateral margins outside the laterobasal depressions, and the convexly curved basal pronotal margin immediately mesad the basal pronotal seta (Fig. 23C). The elytra are narrowed basally, MEW/HuW = 2.10–2.19 (n = 5), and the humeri are narrowly rounded, with the lateral marginal depression bordered along the elytral margin by an upturned edge. If a male specimen is available, the dramatically expanded aedeagal median lobe apex with acuminate dorsal process (Fig. 24F), will easily diagnose this species. Standardized body length 5.3–5.8 mm. *Head* with shallow frontal grooves, laterally margined behind by a rounded carina mesad anterior supraorbital seta, fine wrinkles radiating onto frons, groove broad and smooth anteriorly near frontoclypeal suture; eyes moderately protruded, ocular ratio 1.39–1.49, ocular lobe extended from gena at >135° angle, lobe margined behind by broad, shallow groove at gena, ocular lobe ratio 0.75–0.83; antennae elongate, filiform, antennomere 8 length 2.25× maximal breadth. *Pronotum* moderately transverse, MPW/PL = 1.23–1.33; median base moderately depressed relative to convex disc, about 16 punctures each side and 5-8 elongate punctures along margin with disc; anterior transverse impression broad, shallow but traceable across breadth, about 6 densely arrayed longitudinal strigae crossing impression each side near midline; frontal angles broadly rounded, protruded, lateral marginal depression broader inside angle, narrower behind with margin most beadlike near lateral pronotal seta, margin progressively broader basally, the sinuous laterobasal depression lined with rounded depressions. *Elytra* obovate, the humeri narrowed basally, greatest width just posterad midlength; elytral striae 1–6 deep, irregular along length, the irregularities taking the form of crenulations in the strial walls, discal intervals broadly, moderately convex, stria 7 slightly shallower but still irregular along length; eighth interval more convex medially and laterally at elytral apex, bulging ventrally, not upraised dorsally along seventh stria; lateral elytral setae 7 + 6. *Metathoracic wings* thickened, stenopterous, apex extended beyond hind margin of metanotum, length 2.5× breadth, apex narrowly rounded; remnants of Sc+R and M veins present. *Microsculpture* of pronotum well developed, disc covered with transverse mesh, anterior callosity, lateral marginal depressions, and median base between punctures lined with upraised isodiametric mesh; discal elytral intervals covered with well-developed transverse mesh, sculpticell breadth 2–3× length. *Coloration* of head dark rufous; antennomeres 1–11 rufoflavous, 4–11 with smoky cast; pronotal disc dark rufous with silvery metallic reflection; elytral disc rufobrunneous with a purplish metallic reflection; femora rufoflavous with brunneous cast, tibiae rufobrunneous.

Male genitalia. Aedeagal median lobe shaft slightly broader at midlength, ventral surface slightly convex (Fig. 24F); apex broadly curved dorsally beyond ostium, the apex expanded both dorsally and ventrally, the ventral expansion angulate at the very broadly curved apical face, the dorsal surface acuminate; ostial canal situated on left face of apex, very near and parallel to the dorsal margin; right paramere expanded apically as a ventrally setose lamellate structure; internal sac narrowly tubular, with broadly distributed ventral ostial microtrichial patch, and smaller dorsal ostial microtrichial patch; flagellar plate short, length 0.30× distance from parameral articulations to apical face.

Female reproductive tract. Bursa copulatrix broad with rounded apex, length 2.5× length, cuticle appearing thick based on depth of staining with Chlorazol Black (Fig. 14C); basal gonocoxite 1 quite setose (Fig. 8E), 4–5 setae near apex at medial angle, 4–5 setae along lateral margin of coxite, and unilaterally one seta isolated between; medial surface of gonocoxite 1 lined with about 13 smaller setae; apical gonocoxite 2 robust, broad basolaterally with broadly rounded apex, bearing two broad, parallel-sided lateral ensiform setae and one tapered dorsal ensiform seta; apical sensory furrow bearing two apical nematiform setae and two furrow pegs.

Holotype male (MNHN) labeled: French Polynesia: Tahiti Nui / Pito Hito el. 2000 m 2-VI- / 2006 lot 02 pyrethrin fog / 17°36.790'S, 149°27.842'W / E.M. Claridge // HOLOTYPE / Mecyclothorax / pitohitiensis / J.K. Liebherr 2013 (black-bordered red label).

Allotype female (MNHN): same data as holotype.

Paratypes: Tahiti Nui; Pito Hiti, 2070 m el., 17°36.819'S, 149°27.842'W, 2-vi-2006 lot 01, Claridge, pyrethrin fog (CUIC, 2; EMEC, 3; NMNH, 1); same data as holotype (CUIC, 2; EMEC, 4); 2080 m el., 17°36.806'S, 149°27.842'W, 2-vi-2006 lot 03, Claridge, pyrethrin fog (EMEC, 1).

##### Etymology.

This species name is derived from the type locality, Piti Hiti, resulting in an adjectival epithet – pitohitiensis – that is derived in parallel with that of the closely related species, *Mecyclothorax tahitiensis*.

##### Distribution and habitat.

All specimens representing this species have been collected from 2000–2080 m elevation on Pito Hiti via pyrethrin fogging of moss-covered vegetation.

#### 37. Mecyclothorax teatara

Perrault, 1986: 448

http://species-id.net/wiki/Mecyclothorax_teatara

##### Identification.

This species is easily diagnosed by the broadly ellipsoid elytra and small, little transverse pronotum, MPW/PL = 1.25 (n = 2) (Fig. 23D). It shares pronotal proportions with the sympatric *Mecyclothorax ramagei* (Fig. 22E), but differs in the larger body size, standardized body length 5.3–5.6 mm, and by the presence of two supraorbital setae each side; setal formula 2221. *Mecyclothorax teatara* is characterized by elytral intervals covered by dense transverse lines, with only occasional cross-connections resulting in transverse sculpticells versus the transverse mesh characterizing *Mecyclothorax ramagei*. The male aedeagal median lobe is nearly symmetrically expanded apically, with a rounded ventral and a subangulate dorsal expansion, and the apex face is rounded (Fig. 24G). The ostial canal parallels the dorsal margin, and the right paramere is narrowly extended to a whiplike apex.

##### Distribution and habitat.

This species is known from 800–1100 m elevations on Mont Teatara, Tahiti Iti. One specimen was collected via pyrethrin fogging of mossy horizontal logs and mossy tree trunks.

#### 38. Mecyclothorax fairmairei

Perrault, 1986: 452

http://species-id.net/wiki/Mecyclothorax_fairmairei

##### Identification.

Among species of the *Mecyclothorax dannieae* group, this species uniquely lacks dorsal elytral setae. The apical elytral setae are polymorphic in the single known specimen, with both apical and subapical setae on the right elytron, only the apical seta on the left; therefore the setal formula is 220(1–2). The discal elytral striae 1–5 are distinctly punctate in the basal half (Fig. 25A), the punctures elongate and expanding the strial breadth. The convexity of the eighth elytral interval equals that of the seventh, with a subcarinate ridge parallel to and near the seventh stria. The head bears evident microsculpture in depressed areas and wrinkles, with the isodiametric and transverse sculpticells on the frons arranged in a transverse mesh, the arrangement more isodiametric on the neck. The pronotal disc and discal elytral intervals have well-developed transverse microsculpture, a mixture of transverse lines and transverse mesh, the mesh comprising sculpticells with breadth 2–3× length. The male aedeagal median lobe is slender basally, and expanded both ventrally and dorsally at its apex (Fig. 27A), with the dorsal expansion more tightly rounded than the ventral. Standardized body length 4.3 mm.

##### Distribution and habitat.

The male holotype and only known specimen was collected between 900 and 1100 m elevation on Mont Marau.

#### 39. Mecyclothorax tutei

Liebherr, 2012b: 69

http://species-id.net/wiki/Mecyclothorax_tutei

##### Identification.

This and the similarly sized *Mecyclothorax teatara* share the characteristics of a narrow, subquadrate pronotum and broadly ovate elytra (Figs 23D, 25B), but in *Mecyclothorax tutei* the pronotal hind angles are more angulate, the pronotal lateral margins less explanate, and the elytra base broader. The pronotal median base is also more punctate, with about 13 punctures each side of midline plus rugose wrinkles laterally, versus 6–8 small isolated punctures each and a smooth surface laterally in *Mecyclothorax teatara*. The head has a distinct isodiametric mesh in transverse rows covering the vertex. The pronotal disc is covered with a distinct transverse mesh, sculpticell breadth 2–4× length, and the discal elytral intervals bear a distinct transverse mesh, sculpticell breadth 2–3× length intermixed with transverse lines. Setal formula 2221; standardized body length 5.3 mm.

##### Distribution and habitat.

This species is known from 1080 m elevation on Mont Mauru, where the single known female specimen was collected by pyrethrin fogging tangles of dead fern fronds.

#### 40. Mecyclothorax aorai

Perrault, 1978b: 139; 1986: 449

http://species-id.net/wiki/Mecyclothorax_aorai

##### Identification.

Among *Mecyclothorax dannieae* group species characterized by expanded lateral pronotal margins anterad the hind angles, and well-impressed, nearly smooth elytral striae (Figs 23A–C, 25A, C, D, and 26C, D), *Mecyclothorax aorai* stands out based on the subquadrate elytra with little convex discal elytral intervals (Fig. 25C). The apical elytral setae are polymorphic in this species, with either the apical seta alone present, or both apical and subapical setae present (this configuration varies unilaterally within individuals); setal formula 222(1–2). If a male is available, the diagnosis is certain, as the male aedeagal median lobe is exceedingly slender, with the apex extremely long and narrow and only slightly expanded at the tip (Figs 25C, 27B). The head bears well-developed microsculpture consisting of isodiametric sculpticells in transverse rows. The pronotal disc is covered with an elongate transverse mesh, sculpticell breadth 4× length, and the discal elytral intervals are lined with elongate transverse microsculpture consisting of transverse lines intermixed with transverse sculpticells, breadth 3–4× length. Standardized body length 5.2–5.9 mm.

##### Distribution and habitat.

This species is known to occur from 1200–1900 m elevation on Mont Aorai. All five known specimens were collected in association with moss-covered vegetation, either by finding specimens by hand in moss, or via application of pyrethrin insecticide fog to the vegetation.

#### 41. Mecyclothorax cooki

Perrault, 1986: 449

http://species-id.net/wiki/Mecyclothorax_cooki

##### Identification.

This species is extremely similar to the preceding, *Mecyclothorax aorai*, and in addition to the characters of the key, can be diagnosed by the less transverse pronotum; MPW/PL = 1.20–1.27 (n = 2) for this species versus 1.30–1.37 (n = 2) for *Mecyclothorax aorai*. The pronotal basal margin is straighter immediately mesad the hind angles in this species (Fig. 25D), versus curved forward in *Mecyclothorax aorai* where the basal seta is placed noticeably anterad a line defined by the basal margin of the pronotum near the midline. *Head* with well-developed microsculpture consisting of upraised, transversely stretched sculpticells. The pronotal disc is covered with a swirling transverse mesh, the sculpticell breadth 3–4× length on lateral convex areas. Discal elytral intervals with rough-appearing transverse mesh microsculpture that includes a small proportion of isodiametric sculpticells, the sculpticell margins distinct, transverse sculpticell breadth 2–4× length. Setal formula 2221; standardized body length 5.7 mm.

##### Distribution and habitat.

This species is known to live on Mont Aorai from 1265 m to 1900 m elevation. One of the four known specimens was collected by beating live and dead fern fronds over a beating sheet, indicating the use of an arboreal refuge during daytime.

#### 42. Mecyclothorax marau

Perrault, 1978b: 133; 1986: 450

http://species-id.net/wiki/Mecyclothorax_marau

##### Identification.

Of the five *Mecyclothorax dannieae* group species that exhibit setal formula 2222 (Figs 25C, 26), this species is characterized by a little transverse pronotum – MPW/PL = 1.18–1.26 (n = 5) – with basal margins anteriorly rounded just behind the basal pronotal setae (Fig. 26A). The subquadrate elytra are broad relative to the small pronotum; MEW/MPW = 1.55–1.62. The male aedeagus is perhaps the most distinctive among Tahitian *Mecyclothorax* (Fig. 27C), with a broadly expanded right side to the median lobe laterad the ostium, and a sharply downturned apex. The ostial canal runs along the dorsal surface of the median lobe indicating that the right face of the lobe has been distortedly expanded to produce this configuration. The internal sac bears a stout spicule. The head is glossy in part, with transverse mesh microsculpture in the frontal grooves and associated wrinkles, and an isodiametric mesh on the neck. The pronotal disc is covered with an evident transverse mesh, sculpticell breadth 2–4× length. The discal elytral intervals are lined with transverse lines, only occasionally joined into a mesh with elongate transverse sculpticells. Standardized body length 5.5–5.9 mm.

##### Distribution and habitat.

This species is known from Mont Marau at 1000–1400 m elevations. Associated ecological data are restricted to arboreal situations; in banks of living *Dicranopteris* ferns, in dense banks of dead ferns, and on moss-covered trunks of *Weinmannia* trees with associated epiphytic *Astelia* plants.

#### 43. Mecyclothorax dannieae

Perrault, 1978b: 141; 1986: 450

http://species-id.net/wiki/Mecyclothorax_dannieae

##### Identification.

Of the *Mecyclothorax dannieae* group species with setal formula 2222 (Figs 25C, 26), this species is characterized by the most well-defined pronotal hind angles, with the pronotal lateral margins slightly sinuate anterad the angles (Fig. 26B). The elytra are moderately narrowed basally, the subangulate humeri proximate. The male aedeagal median lobe is sinuously curved apically with a truncate apex (Fig. 27D), with the ostial canal starting at the right-directed apex of the ostium, and terminating near the dorsal margin of the median lobe apex. The male right paramere is expanded apically into a setose lamellate structure, as also observed in males of *Mecyclothorax tahitiensis*, *Mecyclothorax pitohitiensis*, and *Mecyclothorax villiersi* (Figs 24E, F, 27E). Of these four species, *Mecyclothorax dannieae* exhibits the most plesiomorphic median lobe, with only slight sinuation of the lobe beyond the ostium, and minimal dorsoventral expansion at the tip. This species is characterized by well-developed microsculpture, including: 1, upraised, transversely stretched sculpticells covering the frons and vertex; 2, a swirling transverse mesh on the pronotal disc, sculpticell breadth 2–4× length; and 3, regular transverse mesh on the discal elytral intervals 1–4, sculpticell breadth 2–3× length, the mesh more elongate but just as regular on the lateral elytral intervals. Standardized body length 5.4–6.2 mm.

##### Distribution and habitat.

Perrault (1986) reported this species from 1000–2000 m on Mont Aorai. Several of his specimens from between 1100 and 1400 m elevation were collected in vinegar pitfall traps.

#### 44. Mecyclothorax villiersi

Perrault, 1986: 451

http://species-id.net/wiki/Mecyclothorax_villiersi

##### Identification.

This member of the *Mecyclothorax dannieae* species group quartet with setal formula 2222 can be diagnosed by the transvere pronotum with trisinuate basal margin, the margin convexly curved behind the pronotal laterobasal depression toward the obtuse-rounded hind angle defined by the articulatory socket of the basal pronotal seta (Fig. 26C). The rufoflavous femora and tibiae contrast with the rufobrunneous to rufopiceous dorsal body color. The male aedeagal median lobe is sinuously curved apically, with the apex expanded both dorsally and moreso ventrally (Figs 3E, 27E). The ostial canal runs along the dorsal margin of the apex. The right paramere is expanded apically as a setose lamellate structure. The internal sac is tubular, with a variously developed dorsal ostial microtrichial patch, broad ventral ostial microtrichial patch and a small flagellar plate, the plate length 0.22× the distance from the parameral articulations to the apical face (Fig. 27E). The head bears shallow to evident microsculpture consisting of isodiametric and transverse sculpticells in transverse rows. The pronotal disc is covered with distinct transverse mesh microsculpture, the sculpticells isodiametric to 3× broad as long, and the discal elytral intervals are lined with upraised transverse sculpticells, the surface rough on the inner four intervals. Standardized body length 5.0–6.0 mm.

##### Distribution and habitat.

This species is known from Mont Marau at elevations between 1000 and 1400 m. All individuals with ecological data have been associated with arboreal microhabitats on a variety of plant substrates: *Melicope*, *Myrsine*, and *Weinmannia*, as well as ferns. Specimens have been obtained by beating such vegetation, and through the use of pyrethrin insecticide fog on moss-covered vegetation and dense fern banks.

#### 45. Mecyclothorax negrei

Perrault, 1986: 451

http://species-id.net/wiki/Mecyclothorax_negrei

##### Identification.

Among the *Mecyclothorax dannieae* group species with setal formula 2222, this species can be diagnosed by a pronotum with distinct, obtuse hind angles and straight lateral margins anterad the angles (Fig. 26D). The basal pronotal setae are situated directly inside the obtuse angles on an expanded portion of the upraised margin. The pronotal basal margin is indistinctly trisinuate, with slight concavities posterad the deepest portions of each laterobasal depression. The elytra are broad basally, with rounded humeri. The male aedeagal median lobe is even curved with a dorsoventrally expanded apex with a straight apical face (Fig. 27F). The right paramere has an expanded apex with subparallel margins; a configuration observed in males of *Mecyclothorax brittoni* (Fig. 24C). The head is uniformly covered with distinct isodiametric and slightly transverse sculpticells in transverse rows. The pronotal disc is covered with a transverse mesh, sculpticell breadth 1–3× breadth, and the discal elytral intervals are lined with an elongate transverse mesh, sculpticell breadth 3–4× length. Over portions of the elytra, the cross-connections of the mesh are reduced producing transverse-line microsculpture. Standardized body length 5.6–6.4 mm.

##### Distribution and habitat.

The four known specimens of *Mecyclothorax negrei* have been collected on Mont Aorai from 1000 to 1900 m elevation.

### 5. *Mecyclothorax striatopunctatus* species group

**Figure 28.**
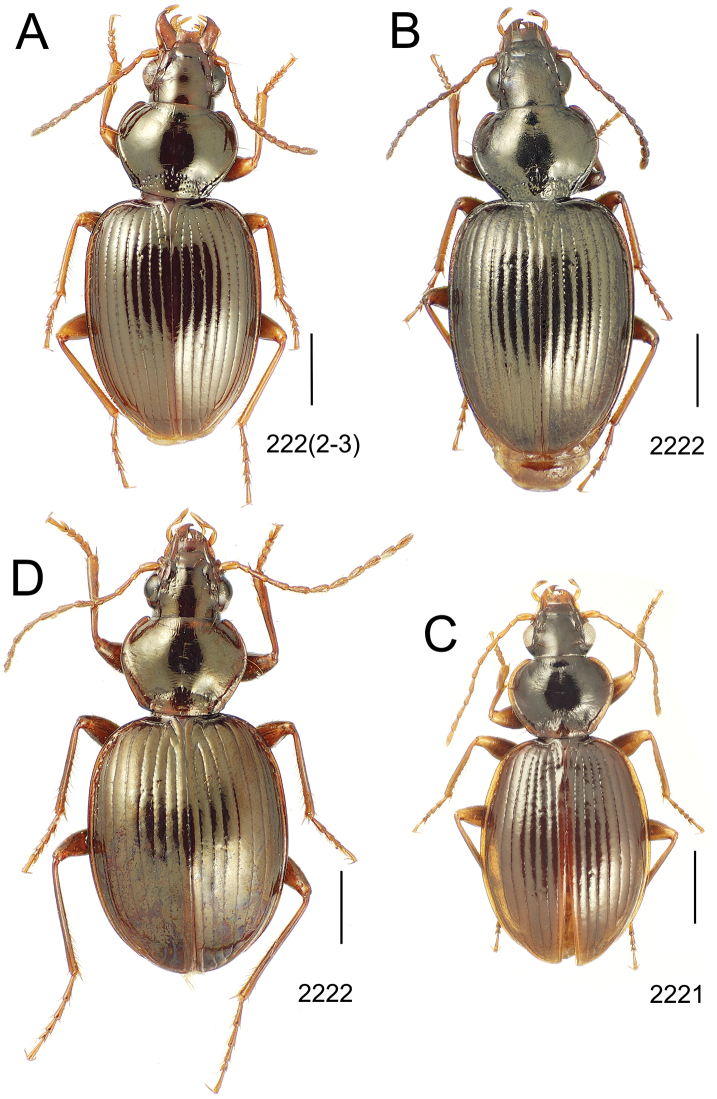
*Mecyclothorax* spp., dorsal view; scale bars 1.0 mm; setal formula (see Fig. 10) at lower right of each figure **A** *Mecyclothorax striatopunctatus* holotype male **B** *Mecyclothorax wallisi* **C** *Mecyclothorax curtisi* holotype male **D** *Mecyclothorax bougainvillei* holotype male.

**Figure 29.**
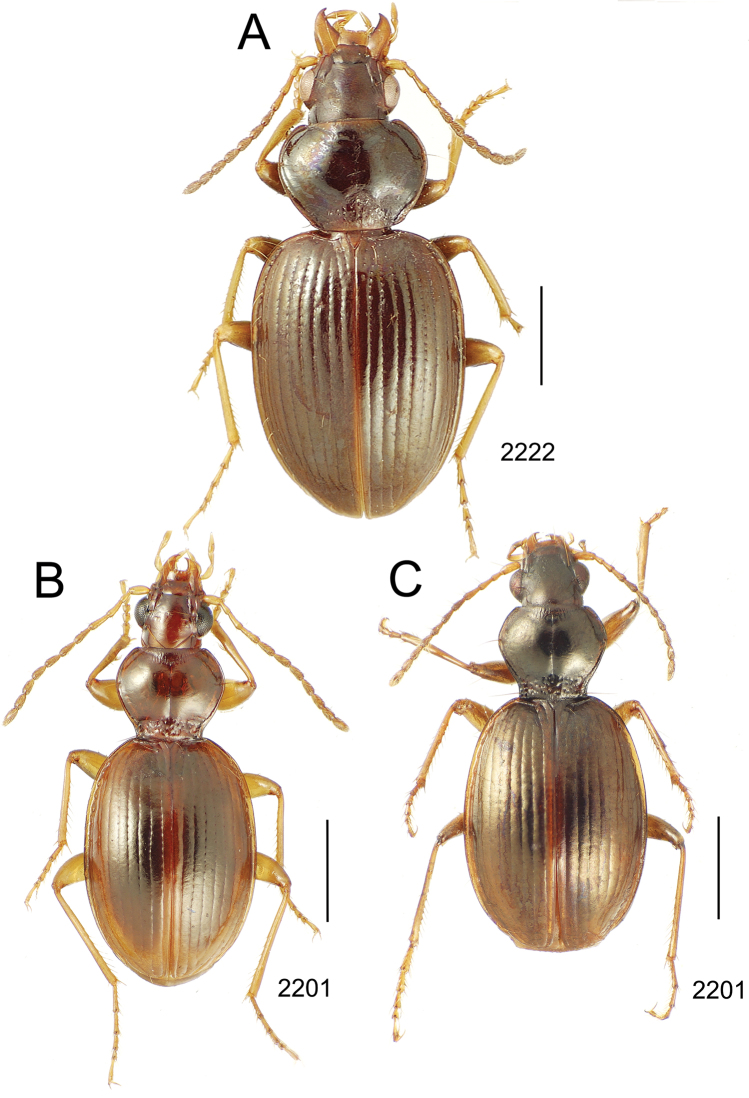
*Mecyclothorax* spp., dorsal view; scale bars 1.0 mm; setal formula (see Fig. 10) at lower right of each figure **A** *Mecyclothorax pomarei* holotype male **B** *Mecyclothorax marginatus* paratype male (MNHN) **C** *Mecyclothorax hoeahiti* paratype female (CUIC).

**Diagnosis.** These five species are characterized by pronotal configuration, with the hind angles rounded and best defined by the basal pronotal setae whose articulatory socket produces a jag in the lateral pronotal margin. The beetles classified in this group exhibit two supraorbital setae, both lateral and basal pronotal setae, two dorsal elytral setae, and from one to three apical elytral setae; setal formulas in the group range include 222(2-3), 2222, and 2221. The head bears convex eyes with the ocular lobe distinctly projected from the gena (Figs 28, 29A). The pronotum is transverse with rounded to bluntly obtuse hind angles. The elytral striae are well developed and punctate, and the elytral intervals broadly convex. Cuticular microsculpture is variable, from reduced to obsolete, to well developed; when present, the elytra bear a transverse mesh to transverse lines. The rounded pronotal hind angles, punctate elytral striae, and transverse elytral microsculpture are observed in the Australian *Mecyclothorax punctipennis* (MacLeay), proposed as the adelphotaxon of the Tahitian radiation (Liebherr 2012a). Thus the *Mecyclothorax striatopunctatus* group species are hypothesized to approximate the groundplan of the Tahitian *Mecyclothorax* radiation, with deviation from these states representing synapomorphies that have evolved during diversification.

#### Identification key to Tahitian species of the *Mecyclothorax striatopunctatus* species group

**Table:** 

1	Discal intervals on elytral base with shallow but distinct transverse microsculpture	2
–	Discal intervals on elytral base glossy, microsculpture not visible (Marau)	46. *Mecyclothorax striatopunctatus* Perrault
2	Pronotal median base smoother mediobasally (Figs 28C, D, 29A), margin with disc lined with elongate punctures, but basal portion only indistinctly punctured, the punctures small and isolated, or broad and shallow	3
–	Pronotal median base densely punctured (Fig. 28B), elongate punctures along margin with disc, and 10 or more distinct, rounded punctures more posteriorly each side (Aorai)	47. *Mecyclothorax wallisi* Perrault
3	Elytra with two setae at apex, the apical seta near apex of stria 2, and the subapical more basad in stria 7; pronotal lateral marginal depression narrow in apical half, concolorous with disc (Figs 28D, 29A)	4
–	Elytra with a single apical seta near apex of stria 2, subapical seta absent; pronotal lateral marginal depression broad to front angle, rufoflavous, contrasted with rufopiceous pronotal disc (Fig. 28C) (Teatara)	48. *Mecyclothorax curtisi* sp. n.
4	Elytra broadly ovate, MEW/MPW = 1.59 (Fig. 28D); sutural stria and stria 2 fused basally laterad the entire length of parascutellar striole, interval 2 absent from elytral base (Marau)	49. *Mecyclothorax bougainvillei* Perrault
–	Elytra more narrowly ovate, MEW/MPW = 1.46 (Fig. 29A); sutural stria and stria 2 free basally until position of parascutellar seta, their short fused stalk extended to elytral basal groove (Aorai)	50. *Mecyclothorax pomarei* Perrault

#### 46. Mecyclothorax striatopunctatus

Perrault, 1986: 442

http://species-id.net/wiki/Mecyclothorax_striatopunctatus

##### Identification.

Among members of this group, this species is diagnosable by the very glossy upper body surface, with the vertex, pronotal disc, and discal elytral intervals extremely smooth such that tiny micropunctures – pore canals – are visible across the smooth surface. The pronotal hind angles are obtuse and only slightly protruded, the projection consisting of an expansion of the marginal bead surrounding the articulatory socket of the basal pronotal seta (Fig. 28A). The pronotal median base is distinctly punctate with about 20 rounded punctures each side that are isolated by glossy cuticle. All elytral striae are deep with rounded punctures that expand their breadth in the basal half. The eighth elytral interval is subcarinate immediately laterad the seventh stria and convex laterally. There are two to three setae near the elytral apex; a short one in interval 7 along the elytral margin, and a second longer one laterad the apically fused terminus of striae 3 + 4. In the male paratype examined (MNHN), there is a third short seta immediately basal to the second; setal formula 222(2-3). The male aedeagal median lobe is narrowed apically, with a small rounded expansion of the tip (Fig. 3A). The right paramere is parallel sided, and slightly expanded to its tightly rounded apex. Standardized body length 5.9 mm.

##### Distribution and habitat.

This species is known from the type series of five specimens collected at 1000 m elevation on Mont Marau.

#### 47. Mecyclothorax wallisi

Perrault, 1986: 442

http://species-id.net/wiki/Mecyclothorax_wallisi

##### Identification.

Beetles of this species appear much like those *Mecyclothorax striatopunctatus*, with basally broad elytra bearing distinctly punctate striae and convex intervals, and glossy dorsal body surface (Figs 28A, B). However this species exhibits rudimentary transverse microsculpture on the head and pronotum, and distinct transverse mesh microsculpture, sculpticell breadth 2–3× length, on the discal elytral intervals. The pronotal median base is distinctly punctate, with >25 deep, round punctures each side, and the pronotal hind angles are more rounded than in *Mecyclothorax striatopunctatus*. In *Mecyclothorax wallisi*, the basal pronotal seta is positioned anterad the rounded hind angles, and its articulatory socket causes a jag in the basal portion of the pronotal lateral margin. The eighth elytral interval is broadly convex laterad the seventh stria, with its surface bulging laterally. The male aedeagal median lobe is similar to that of male *Mecyclothorax striatopunctatus* (Fig. 3A), with an evenly curved gracile shaft that is elongate apicad the ostium, and a dorsoventrally expanded apex, the ventral expansion larger and more rounded (Perrault 1986: fig. 22) than observed in the males of *Mecyclothorax striatopunctatus* (Fig. 3A). Setal formula 2222; standardized body length 5.9–6.2 mm.

##### Distribution and habitat.

Perrault (1986) collected the type series between 1000 and 1800 m elevation on Mont Aorai. One subsequent specimen was collected by beating dead and live fern fronds between 1265 and 1320 m elevation on Aorai.

#### 48. Mecyclothorax curtisi sp. n.

http://zoobank.org/F583A292-4519-48A7-95F7-AD2581B8A02E

http://species-id.net/wiki/Mecyclothorax_curtisi

##### Diagnosis.

Unique among the *Mecyclothorax striatopunctatus* group in the orbicular pronotum with broad pale margins, and the obovate elytra with pale, broadly explanate lateral marginal depressions (Fig. 28C). Setal formula 2221; standardized body length 5.4 mm. *Head* with deep frontal grooves, narrow behind and depressed mesad a low carina inside anterior supraorbital seta, sinuous toward midline at midlength, and broad, shallow near frontoclypeal suture; eyes moderately convex but small in diameter, ocular ratio 1.50, ocular lobe distinctly expanded from gena, ocular lobe ratio 0.80; antennae moderately elongate, filiform, eighth anennomere length 2.0× maximal breadth. *Pronotum* transverse, MPW/PL = 1.31, orbicular, with broadly rounded hind angles and convex basal margin; basal pronotal setae expanding lateral margin, causing minute jag in margin, the seta and jag set 0.12× pronotal length anterad the pronotal basal margin at the midline; median base with four shallow, isolated punctures each side and 7–8 longitudinal wrinkles at juncture with disc; anterior transverse impression broad, shallow, complete medially, crossed by 10–11 fine wrinkles in median quarter of each side; front angles broadly rounded, little protruded anteriorly, the lateral marginal depression gradually widened posterad to base, laterobasal depression defined by a U-shaped depression that is continuous with the lateral depression surrounding a low, irregular tubercle. *Elytra* narrow basally, MEW/HuW = 2.67, the basal groove distinctly recurved anteriorly to the subangulate humerus; parascutellar seta on papillate dome that is bordered behind by a depression connecting parascutellar striole and interval 1; disc depressed across first four intervals each side directly posterad the inflated parascutellar striole; flat lateral marginal depression gradually widened with margin more upraised posterad humerus, achieving maximal breadth near posterior seta of anterior lateral elytral setal series; discal elytral striae 1–6 deep, set with distinct elongate punctures that expand the strial breadth in a crenulate pattern; stria 7 shallow, finely inscribed, punctures isolated, especially apically; eighth elytral interval upraised above striae 7 from anterad subapical sinuation to apex, tightly convex, looking pinched laterally dorsad subapical sinuation; lateral elytral setae 7 + 1 + 5 (left) and 7 + 6 (right) of unique holotype. *Metathoracic wings* stenopterous straps 4× long as wide, margins parallel in apical half, apex narrowly rounded; alae with distinct Sc+R and M veins present, and indistinct wrinkles in cubital region of wing rudiment. *Microsculpture* of head a distinct transverse mesh visible across frontal grooves, frons, and vertex; pronotal disc covered with well-developed transverse mesh, the sculpticell pattern influenced by wrinkles radiating from the disc center, sculpticell breadth 3–4× length; discal elytral intervals with regular transverse mesh, sculpticell breadth 2–3× length, with some isodiametric sculpticells intermixed. *Coloration* of head a uniform rufobrunneous; antennomere 1 flavous, each antennomere 2-11 rufobrunneous basally, rufoflavous apically; pronotal disc rufobrunneous to match head color, lateral marginal depression rufoflavous, palest at front angle; elytral disc flavobrunneous, paler than head and pronotal disc; elytral lateral marginal depression concolorous with disc at humerus, paler apically all the way to subapical sinuation; femora brunneous with flavous apex, tibiae flavous with brunneous cast. Male abdomen with extra transverse, arcuate fold near midlength of visible abdominal ventrite 6, resulting in a partially formed ventrite 7; two smaller setae at apical margin of pseudosegment 6, in line with accessory setae of ventrites 3–5; apical margin of pseudosegment 7 with the usual two apical setae at lateral quarter of breadth each side.

Male genitalia. Aedeagal median lobe only moderately curved, ventral surface of shaft straight apicad parameral articulations (Fig. 27G); median lobe apex short, rounded, slightly more expanded on dorsal margin; ostial canal starting on ride side of lobe apex, terminated closer to dorsal margin; flagellar plate elongate, length ~0.50× distance from parameral articulations to apical face; right paramere slightly broadened, parallel sided from base to tightly rounded apex.

Holotype male (MNHN) labeled: French Polynesia: Tahiti Iti / Mts. Teatara summit / 1195 m el. 17-IX-2006 lot 10 / 17°47.907'S, 149°14.183'W / pyr. fog *Astelia* on mossy / log C.P. Ewing // HOLOTYPE / Mecyclothorax / curtisi / J.K. Liebherr 2013 (black-bordered red label).

##### Etymology.

The patronymic species epithet curtisi honors the collector, Dr. Curtis P. Ewing, who collected extensively at the summit of Mont Teatara, one of several inaccessible French Polynesian peaks he has worked hard to explore.

##### Distribution and habitat.

The holotype and only known specimen was collected at 1195 m elevation at the summit of Mont Teatara, Tahiti Iti. The specimen came from a pyrethrin fog sample of a horizontal mossy log with an epiphytic *Astelia* plant growing on it.

#### 49. Mecyclothorax bougainvillei

Perrault, 1986: 443

http://species-id.net/wiki/Mecyclothorax_bougainvillei

##### Identification.

This species shares with *Mecyclothorax curtisi* the rounded pronotal hind angles and basal pronotal setal placement 0.12× the pronotal length before the median basal margin, but differs in: 1, the nearly concolorous pronotal and elytra discs and margin; 2, the very convexly domed elytra with the scutellum much depressed from the middle of the disc; 3, the presence of both apical and subapical elytral setae, therefore a setal formula of 2222; and 4, larger size, standardized body length 6.2 mm. Like *Mecyclothorax curtisi*, the pronotal median base has only small, shallow punctures, about 8–10 each side, and elongate wrinkles along the juncture with the disc. The head is glossy with an indistinct transverse mesh visible in wrinkles and on the neck. The pronotal disc is also glossy, with elongate transverse mesh microsculpture visible in the lateral marginal depressions and laterobasal depressions, as well as the median base between punctures. The discal elytral intervals are covered with a shallow, regular transverse mesh, sculpticell breadth 2–3× length. The male aedeagal median lobe is slightly more curved than in *Mecyclothorax curtisi*, but shares: 1, a short apex (Fig. 3B); 2, an ostial canal that traverses the right face and terminates near the dorsal margin; and 3, a right paramere that is somewhat broad and parallel sided from base to tightly rounded apex. The apex of the median lobe in *Mecyclothorax bougainvillei* males uniquely bears an apical tooth that extends from the basal quarter of the apical face.

##### Distribution and habitat.

All that is known about this species’ distribution is the elevation of the type locality for the unique holotype: 1400 m elevation on Mont Marau.

#### 50. Mecyclothorax pomarei

Perrault, 1986: 443

http://species-id.net/wiki/Mecyclothorax_pomarei

##### Identification.

This is the smallest-bodied species in the *Mecyclothorax striatopunctatus* group, standardized body length 5.3 mm, and it is further diagnosable by the little widened pronotal lateral depressions near the obtusely angulate hind angles (Fig. 29A). As in the preceding species, the basal pronotal setae are positioned anterad a transverse line defined by the median basal pronotal margin, but in this species the distance anterad is less, only 0.10× the pronotal length. The pronotal median base is mostly smooth, bearing 20–25 small, isolated punctures each side but no longitudinal wrinkles at the median base-pronotal disc juncture. The eighth elytral interval is elevated, broadly subcarinate above stria 7 dorsad the subapical sinuation, and convexly bulging laterally. The head bears a shallow, indistinct transverse mesh on frons and vertex, the surface only moderately glossy. The pronotal disc and median base are glossy, without indication of any sculpticells. The discal elytral striae are lined with elongate transverse microsculpture, consisting mostly of shallow transverse lines with occasional crossconnection causing areas of transverse mesh. Setal formula 2222.

##### Distribution and habitat.

The unique female holotype of this species was collected between 1000 and 1800 m elevation on Mont Aorai.

### 6. *Mecyclothorax marginatus* species group

**Diagnosis.** Beetles in this group exhibit the unique combination of basally constricted, quadrisetose pronotum with elongate sinuation of the lateral margins, narrowly ovate elytra with proximate humeri, and no dorsal setae (setal formula 2201). Standardized body lengths 3.7–4.3 mm.

#### Identification key to Tahitian species of the *Mecyclothorax marginatus* species group

**Table:** 

1	Discal elytral striae lined with rounded punctures that laterally expand striae; elytral sutural interval and lateral two intervals contrastedly paler than brunneous intervals 2–6 (Fig. 29B); male aedeagal median lobe apex distinctly downturned, the ventral surface decidedly concave (Fig. 30A) (Aorai)	51. *Mecyclothorax marginatus* Perrault
–	Discal elytral striae indistinctly punctured, the punctures elongate, not laterally expanding striae; elytra distinctly paler laterally, but only the lateral marginal depression contrastedly paler than disc (Fig. 29C); male aedeagal median lobe apex only slightly downturned, the ventral surface little concave (Fig. 30B) (Pito Hiti)	52. *Mecyclothorax hoeahiti* sp. n.

#### 51. Mecyclothorax marginatus

Perrault, 1978b: 160; 1986: 453

http://species-id.net/wiki/Mecyclothorax_marginatus

##### Identification.

**Figure 30.**
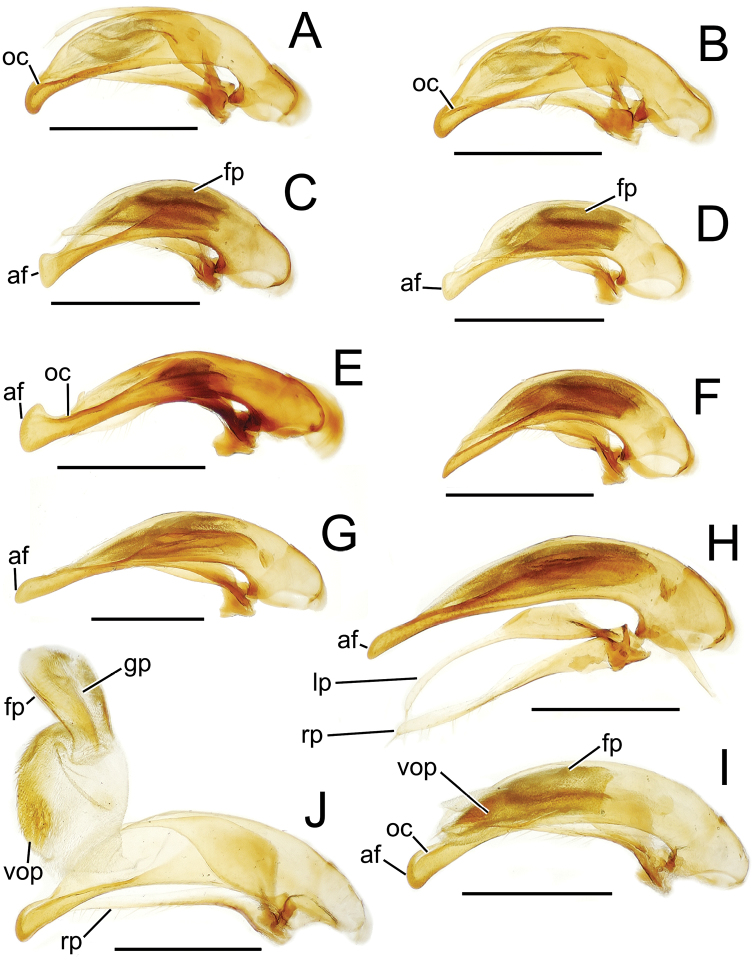
Male aedeagal median lobe and associated parameres, *Mecyclothorax* spp., right lateral view; scale bars 0.5 mm **A** *Mecyclothorax marginatus* paratype (MHNB) **B** *Mecyclothorax hoeahiti* paratype (CUIC) **C** *Mecyclothorax ninamu* paratype (CUIC) **D** *Mecyclothorax viridis* **E** *Mecyclothorax castaneus* **F** *Mecyclothorax kayballae* paratype (CUIC) **G** *Mecyclothorax balli* paratype (MNHN) **H** *Mecyclothorax ata* holotype **I** *Mecyclothorax ehu* paratype (CUIC) **J** *Mecyclothorax ehu* paratype (CUIC), internal sac everted Abbreviations: **af** apical face **fp** flagellar plate **gp** gonopore **lp** left paramere **oc** ostial canal **rp** right paramere **vop** ventral ostial microtrichial patch.

The pale rufoflavous head and pronotum, and pale-margined elytra with contrasting dark brunneous intervals 1–6 serve to diagnose this species at first glance (Fig. 29B). The presence of two supraorbital setae, lateral plus basal pronotal setae, and the absence of dorsal elytral setae – setal formula 2201 – serve to separate this species from all other Tahitian species with narrowly constricted pronota, with the single exception of *Mecyclothorax kayballae* sp. n. (Fig. 32A), a darkly metallic member of the *Mecyclothorax viridis* group. The frons and vertex of the head are covered with a regular, well-developed transverse mesh. The pronotal disc is similarly microsculptured, though the sculpticells are more elongate; 2–4× broad as long. The discal elytral intervals bear a regular transverse mesh, sculpticell breadth 2–4× length, with some isodiametric sculpticells in transverse rows mixed in. The male aedeagal median lobe is bowed dorsally, with the ventral margin straight apicad the parameral articulations, and the tip downturned to a rounded apex (Fig. 30A). The ostium extends almost to the tip, with the ostial canal short and parallel to the dorsal margin. Standardized body length 4.1–4.2 mm.

**Figure 31.**
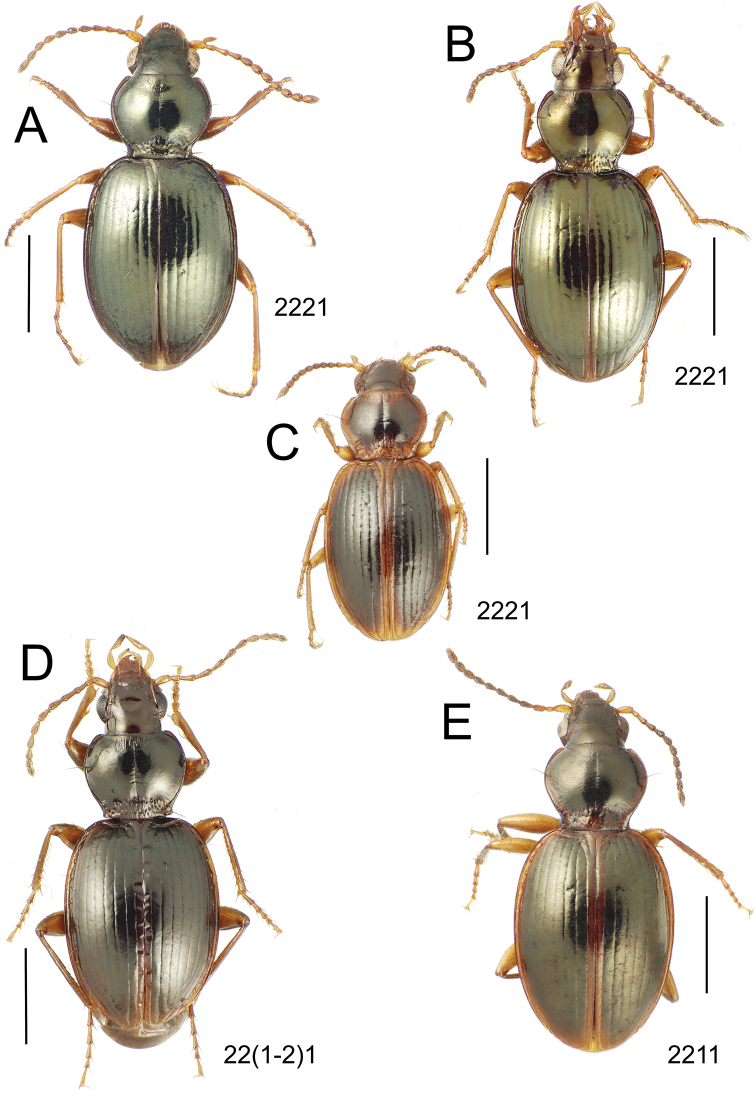
*Mecyclothorax* spp., dorsal view; scale bars 1.0 mm; setal formula (see Fig. 10) at lower right of each figure **A** *Mecyclothorax ninamu* paratype male (CUIC) **B** *Mecyclothorax viridisi* paratype male (MNHN) **C** *Mecyclothorax kokone* holotype female **D** *Mecyclothorax castaneus* **E** *Mecyclothorax paahonu* holotype female.

**Figure 32.**
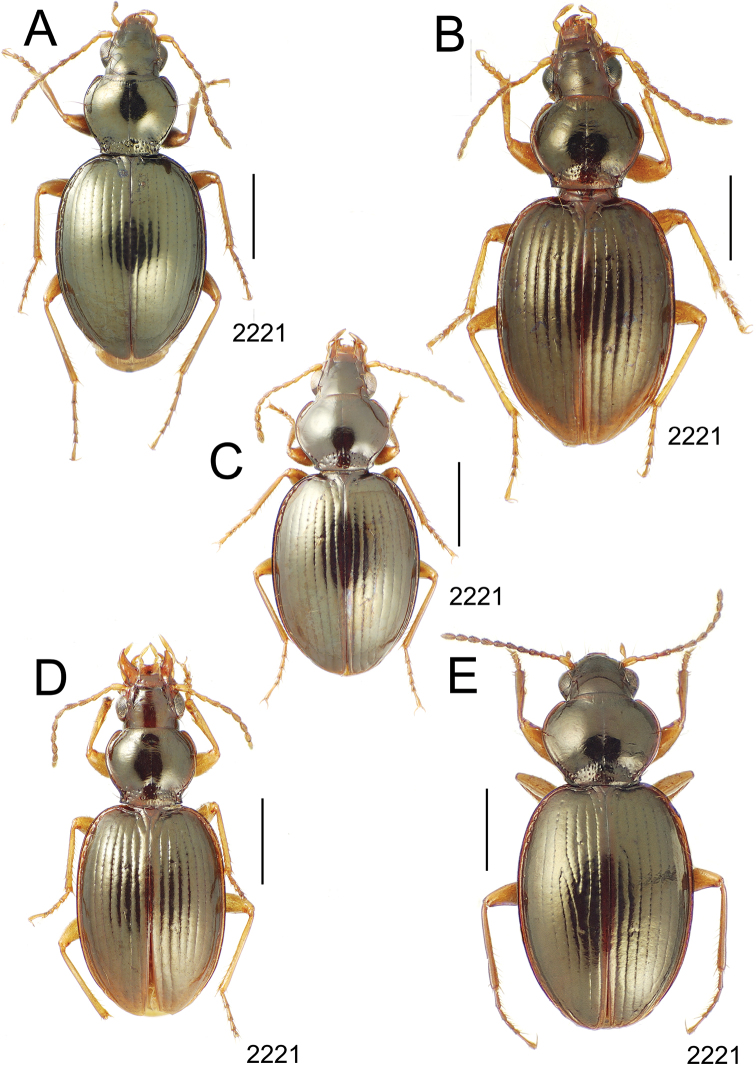
*Mecyclothorax* spp., dorsal view; scale bars 1.0 mm; setal formula (see Fig. 10) at lower right of each figure **A** *Mecyclothorax kayballae* paratype male (CUIC)**B** *Mecyclothorax balli* paratype male (MNHN) **C** *Mecyclothorax putaputa* paratype female (CUIC) **D** *Mecyclothorax ata* holotype male **E** *Mecyclothorax ehu* paratype male (CUIC)

##### Distribution and habitat.

The known distribution of this species spans 1750–1900 m elevation on Mont Aorai.

#### 52. Mecyclothorax hoeahiti sp. n.

http://zoobank.org/5A84EB38-0939-4F20-A364-111CE4EAE277

http://species-id.net/wiki/Mecyclothorax_hoeahiti

##### Diagnosis.

This species can be diagnosed from *Mecyclothorax marginatus* by the narrower, more elongate pronotum – MPW/PL = 1.13–1.22 (n = 5) (Figs 29B, C) – and the more uniform coloration; rufobrunneous head and pronotum, and dark rufobrunneous elytra. On the elytra, only the moderately explanate elytral lateral marginal depressions are flavous. The male aedeagal median lobe is also diagnostic, with the apex broadly rounded as in males of *Mecyclothorax marginatus*, but not downturned (Figs 30A, B). Setal formula 2201; standardized body length 3.7–4.3 mm. *Head* with broadly convex frons, the frontal grooves posteriorly only a shallow, sinuous depression mesad fine carina inside eye, anteriorly a broad shallow triangular depression at frontoclypeal suture; eyes convex, ocular ratio 1.47–1.61, on ocular lobes that are distinctly protruded behind, ocular lobe ratio 0.84–0.87 (n = 5); antennae filiform, antennal segment 8 length 2.25× maximal breadth. *Pronotum* cordate, MPW/BPW = 1.50–1.60; lateral margins distinctly sinuate anterad distinct right to slightly obtuse hind angles, the lateral margins subparallel to convergent for short distance anterad angles; median base with 9–10 distinct, isolated punctures each side; median longitudinal impression moderately deep, crossed by 7–8 indistinct longitudinal wrinkles in median half of each side; lateral marginal depression narrowly upturned, the upturned area translucent, twice as broad and upraised basally laterad laterobasal depression; laterobasal depression a quadrate flat expanse inside hind angle. *Elytra* narrowly obovate, convex, sides sloped to nearly perpendicular with narrowly upturned lateral marginal depression; humeri proximate, angulate, a distinct hitch at the juncture of the basal groove and lateral marginal depression, MEW/HuW = 2.23–2.54; discal elytral striae shallow, with shallow punctures that expand the strial breadth but not depth, discal intervals only slightly convex; eighth interval broadly convex laterad stria 7 apicad subapical sinuation, not raised much above stria 7; lateral elytral setae 7 + 6. *Microsculpture* of head a distinct transverse mesh composed of isodiametric and transverse sculpticells; pronotal disc with evident transverse mesh, sculpticell breadth 2–3× length; discal elytral intervals covered with a distinct transverse mesh, sculpticells 2–3× length, mixed with areas of transverse lines. *Coloration* of head and pronotal disc rufobrunneous, pronotal margins narrowly rufoflavous; elytra rufobrunneous, the sutural interval rufous basally and rufoflavous apically, and the apex gradually more flavous apicad the subapical sinuation.

Male genitalia. Aedeagal median lobe bowed dorsally with straight ventral margin apicad parameral articulations (Fig. 30B), as in males of *Mecyclothorax marginatus* (Fig. 30A), but apex not downturned.

Female reproductive tract. Bursa copulatrix narrowly extended from broader vagina, the bursal extension twice as long as broad (Fig. 14D); basal gonocoxite 1 broad, squat, with apical fringe of four setae, three more laterally and a shorter one more medially (Fig. 8F), and 5–6 short setae in apical portion of median margin; apical gonocoxite 2 broadly extended laterally, with arcuate lateral margin and narrow, finely rounded apex; apical gonocoxite with two narrow lateral ensiform setae and one narrow dorsal ensiform seta; apical sensory furrow with two nematiform setae and two furrow pegs.

Holotype male (MNHN) labeled: French Polynesia: Tahiti Nui / Pito Hito el. 2000 m 2-VI- / 2006 lot 02 pyrethrin fog / 17°36.790'S, 149°27.842'W / E.M. Claridge // HOLOTYPE / Mecyclothorax / hoeahiti / J.K. Liebherr 2013 (black-bordered red label).

Allotype female (MNHN) labeled as holotype.

Paratypes: labeled as holotype (EMEC, 1); Tahiti Nui, Pito Hiti, 2070 m el., 17°36.813'S, 149°27.842'W, 2-vi-2006 lot 01, Claridge, pyrethrin fog (EMEC, 1), 2080 m el., 17°36.806'S, 149°27.842'W, 2-vi-2006 lot 03, Claridge, pyrethrin fog (CUIC, 2; EMEC, 1).

##### Etymology.

The species epithet compounds the Tahitian word for same, hō’ēā with the word for edge, hiti, signifying the similar coloration of pronotal and elytral margins in beetles of this species; *Mecyclothorax hoeahiti*.

##### Distribution and habitat.

This species is known from pyrethrin fogging of moss-covered vegetation from 2000–2080 m elevation on Pito Hiti.

### 7. *Mecyclothorax viridis* species group

**Diagnosis.** Beetles classified in this group exhibit a quadrisetose pronotum with narrow base and distinctly sinuate lateral margins (Figs 31, 32). The elytra are ovate with narrowly rounded to slightly laterally extended humeri. There is always a single apical elytral seta, the subapical seta uniformly absent. Two Moorean species assigned to this group have also been described (Liebherr 2012a). Standardized body lengths 3.3–5.1 mm.

#### Identification key to Tahitian species of the *Mecyclothorax viridis* species group

**Table:** 

1	Elytral microsculpture absent, surface glossy, coloration dark rufobrunneous to rufopiceous with aeneous reflection; elytral striae shallow and smooth, intervals slightly convex; pronotal lateral margin very narrow, edge beaded near lateral setae	2
–	Elytral microsculpture evident, more or less transverse, coloration rufobrunneous without distinct metallic reflection; elytral intervals moderately convex, striae deep, punctate basally; pronotal margin narrow, edge upturned	3
2	Body dark, glossy piceous (Fig. 31A), head and pronotum as dark as elytra; elytra more broadly ovate, MEW/HuW = 1.97–2.09; elytral striae 1 and 7 deeply and narrowly impressed at elytral apex, striae 2–4 broadly and more shallowly depressed, margins not well defined (Pito Hiti)	53. *Mecyclothorax ninamu* sp. n.
–	Body paler, rufopiceous (Fig. 31B), head and pronotum more brunneous than glossy metallic piceous elytra; elytra more narrowly ovate, MEW/HuW = 2.28; elytral striae 1, 2 and 7 deeply and narrowly impressed at elytral apex, striae 3–4 nearly as deep and well defined (Aorai)	54. *Mecyclothorax viridis* Perrault
3	Elytral striae 2–6 continuously and uniformly impressed from disc to elytral basal groove (Figs 31C–E)	4
–	Elytral striae 2–6 much deeper on disc than near elytral basal groove where striae may be very shallow and traceable or absent (Fig. 32)	6
4	Larger beetles, standardized body length 3.8–5.7 mm; pronotal and elytral margins narrowly paler, rufobrunneous to rufoflavous versus – depending on degree of melanization – correspondingly rufopiceous to rufobrunneous discs; one dorsal elytral seta in third interval	5
–	Small beetles, standardized body length 3.3–3.4 mm; pronotal and elytral margins and suture broadly flavous, contrasted with rufobrunneous pronotal and elytral discs (Fig. 31C); two dorsal elytral setae in third interval (Pito Hiti)	55. *Mecyclothorax kokone* sp. n.
5	Pronotal median base unformly depressed relative to disc, margined anteriorly at disc by dense longitudinal strigae (Fig. 31D); parascutellar striole shallower than adjacent portion of sutural stria 1, shallow to discontinuous between shallow punctures; elytral microsculpture dense transverse lines without tendency to form a mesh (Marau)	56. *Mecyclothorax castaneus* Perrault
–	Pronotal median base sloping anteriorly to meet disc, anterior margin nearly coplanar with disc and as punctured as remainder of median base (Fig. 31E); parascutellar striole deep and smooth and nearly as deep as adjacent portion of sutural stria 1; elytral microsculpture a regular transverse mesh, a majority of elongate sculpticells mixing with a few nearly isodiametric sculpticells (Pito Hiti)	57. *Mecyclothorax paahonu* sp. n.
6	Third elytral interval with two dorsal setae, the setal articulatory sockets in evident, depressed punctures (Figs 32B–E)	7
–	Third elytral interval glabrous, dorsal elytral setae absent (Fig. 32A) (Marau)	58. *Mecyclothorax kayballae* sp. n.
7	Elytral microsculpture consisting of dense transverse lines with little tendency to form a mesh	8
–	Elytral microsculpture consisting of transverse mesh in regular transverse rows, crossconnections between transverse sculpticell margins obvious	9
8	Pronotal lateral margin distinctly sinuate before hind angle, laterobasal depression deep; pronotal median base covered with irregularly distributed, deep and distinct punctures; discal elytral striae convex, striae 1–7 distinctly punctate in basal half of length (Fig. 32B) (Marau)	59. *Mecyclothorax balli* Perrault
–	Pronotal lateral margin slightly sinuate before hind angle, laterobasal depression shallow; pronotal median base bearing 2–3 transverse rows of small, shallow, sparsely distributed punctures; discal elytral striae broadly, slightly convex, striae 1–5 minutely punctate in basal half of length (Fig. 32C) (Mauru)	60. *Mecyclothorax putaputa* Liebherr
9	Pronotal hind angles protruded, right, pronotal lateral margin distinctly, angularly convergent immediately anterad hind angle; elytral striae distinctly punctate on disc, discal intervals convex (Fig. 32D) (Aorai)	61. *Mecyclothorax ata* Perrault
–	Pronotal hind angles obtuse, denticulate, pronotal lateral margin subparallel for very short distance outside basal seta articulatory socket; elytral striae moderately shallow, punctate, but intervals only slightly convex (Fig. 32E) (Pito Hiti)	62. *Mecyclothorax ehu* sp. n.

#### 53. Mecyclothorax ninamu sp. n.

http://zoobank.org/AEDA605B-0011-4C55-97B4-42EFDC5E2920

http://species-id.net/wiki/Mecyclothorax_ninamu

##### Diagnosis.

Among the five species in the *Mecyclothorax viridis* group that exhibit smaller body size, standardized body lengths 3.3–4.2 mm (Fig. 31), this species is characterized by a very dark, glossy dorsal surface with metallic blue reflection. The head, pronotal disc, and discal elytral intervals lack distinct microsculpture, with areas of obsolete, elongate transverse mesh visible over portions of each somite. Only the first and seventh elytral striae are deeply impressed apically, with the second stria broader and much less defined. Setal formula 2221; standardized body length 3.5–4.0 mm. *Head* with narrowly incised, subparallel frontal grooves, the mesal surface with transversely radiating wrinkles inside anterior supraorbital seta, grooves broader, quadrately depressed at frontoclypeal suture; eyes moderately convex but with dorsal margin convexly convergent, ocular ratio 1.49–1.55, ocular lobe distinctly protruded from gena, ocular lobe ratio 0.82–0.89; antennae short, submoniliform, antennomere 8 length 1.75× maximal breadth. *Pronotum* cordate, lateral margins distinctly convergent anterad right to slightly obtuse, protruded hind angles, MPW/BPW = 1.49–1.56 (n = 5), and moderately transverse, MPW/PL = 1.19–1.24; median base only slightly depressed relative to disc, 16–19 deep, rounded punctures each side, the punctures more elongate along juncture with disc; anterior transverse impression broad, shallow, medially crossed by indistinct longitudinal wrinkles in some individuals, defined anteriorly by upraised, nearly flat anterior callosity; front angles not protruded, rounded behind; lateral marginal depression narrow throughout length, edge beaded, broadened inside front angle; laterobasal depression a narrow oblique continuation of lateral marginal depression, surface punctate, the depression defining a triangular raised area with the basal pronotal seta. *Elytra* broadly ovate, convex, sides distinctly sloped to vertical at lateral marginal depression; humerus broadly angulate laterad the evenly curved basal groove; striae 1–7 shallow but evident, continuous, obsolete basally and not reaching basal groove, indistinctly punctate in basal half, smooth and deeper apically; lateral marginal depression broadest at humerus, narrow with beaded margin posteriorly; interval 8 more convex than intervals 2 or 7 at elytral apex, surface broadly convex both dorsally and laterally; lateral elytral setae 7 + 6; the lone apical elytral seta in outer half of terminally fused striae 3 + 7 near apical terminus of interval 8. *Coloration* of head rufopiceous with silvery reflection, mandibles rufoflavous; antennomeres 1–3 rufoflavous, 4–11 with rufous cast; pronotal disc rufopiceous with indistinct blue-green reflection, only the median base paler, rufobrunneous near margin; elytral disc rufopiceous with more distinct blue-green reflection, lateral marginal depression narrowly rufobrunneous at humerus, rufoflavous apically; femora and tibiae contrastedly pale, rufoflavous, the tibiae with brunneous cast.

Male genitalia. Aedeagal median lobe curved dorsally, apex broadened dorsoventrally with broad, flat apical face (Fig. 30C), the apical configuration an exaggeration of that observed in males of *Mecyclothorax viridis* (Fig. 30D); flagellar plate relatively elongate for the short median lobe, length 0.52× distance from parameral articulations to apical face.

**Figure 33.**
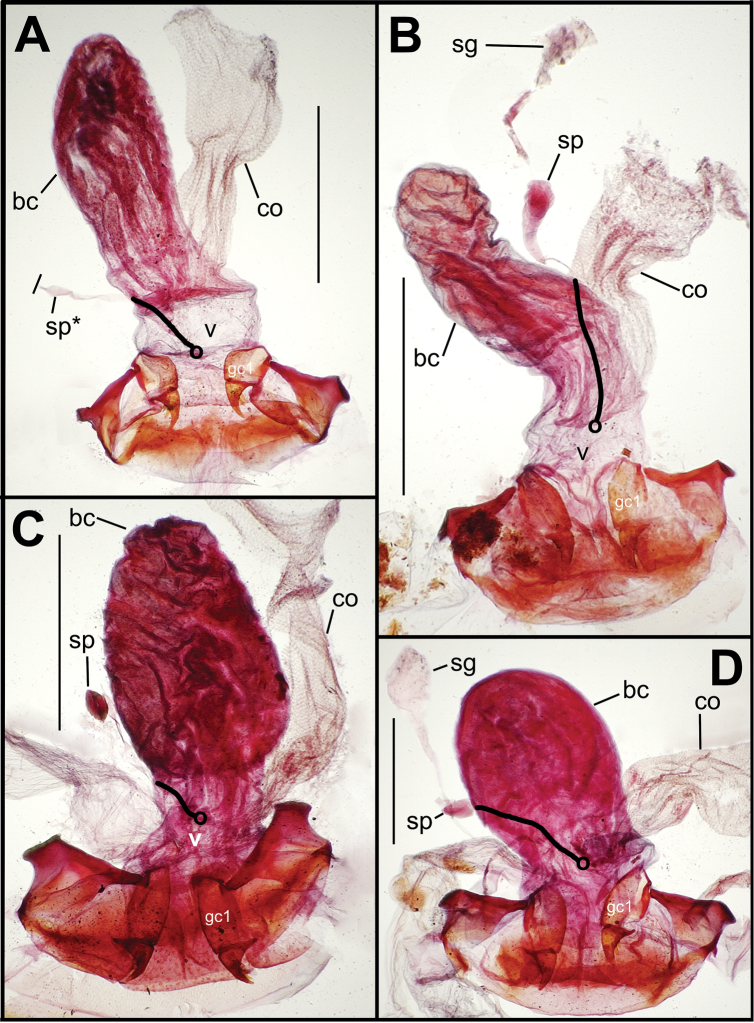
Female reproductive tract dissections, *Mecyclothorax* spp., ventral view; scale bars 0.5 mm **A** *Mecyclothorax ninamu* paratype (CUIC); spermatheca and spermathecal gland broken off dissection **B** *Mecyclothorax kokone* paratype (CUIC) **C** *Mecyclothorax ehu* paratype (CUIC) **D** *Mecyclothorax taatitore* paratype (CUIC) Abbreviations: **bc** bursa copulatrix **co** common oviduct **gc1** basal gonocoxite 1 **sg** spermathecal gland **sp** spermatheca sp* spermatheca, reservoir detached **v** vagina. Position of spermathecal duct and juncture of duct with dorsal wall of bursa indicated by black line and terminal circle, respectively.

Female reproductive tract. Bursa copulatrix elongate, symmetrical and only slightly narrower than vagina in unstretched configuration (Fig. 33A), bursal + vaginal length 3.7× bursal breadth when flattened under cover slip; basal gonocoxite 1 short, broad, with apical fringe of four setae and 6–8 smaller setae along medial margin (Fig. 9A); apical gonocoxite 2 stout, moderately expanded basally with arcuate lateral margin, and bearing two subequal lateral ensiform setae, a dorsal ensiform seta; apical sensory furrow with two apical nematiform setae and two furrow pegs.

Holotype male (MNHN) labeled: French Polynesia: Tahiti Nui / Pito Hito el. 2000 m 2-VI- / 2006 lot 02 pyrethrin fog / 17°36.790'S, 149°27.842'W / E.M. Claridge // HOLOTYPE / Mecyclothorax / ninamu / J.K. Liebherr 2013 (black-bordered red label).

Allotype female (MNHN) labeled: French Polynesia: Tahiti Nui / Pito Hito el. 2070 m 2-VI- / 2006 lot 01 pyrethrin fog / 17°36.813'S, 149°27.842'W / E.M. Claridge // HOLOTYPE / Mecyclothorax / ninamu / J.K. Liebherr 2013 (black-bordered red label).

Paratypes: 3 paratypes labeled as the holotype (CUIC, 2; EMEC, 1).

##### Etymology.

The Tahitian word nīnamu means blue as in the blue color of a lagoon, and signifies the metallic dorsal coloration of these beetles.

##### Distribution and habitat.

This species inhabits the summit area of Pito Hiti from 2000–2070 m elevation, with specimens collected from moss-covered vegetation through the application of pyrethrin fog.

#### 54. Mecyclothorax viridis

Perrault, 1978b: 158; 1986: 454

http://species-id.net/wiki/Mecyclothorax_viridis

##### Identification.

Within the *Mecyclothorax viridis* group this species shares with *Mecyclothorax ninamu* the absence of discernible microsculpture on the dorsal body surface and cordate pronotum (Figs 31A, B), but the dorsal body coloration is not so dark, with the head a dark rufobrunneous, contrasted with the metallic piceous elytral disc. The overall body is narrower, with the pronotum less transverse, MPW/PL = 1.11–1.15 (n = 2), and the elytra more narrowly ovate. The elytral striae are more well developed in this species, with the eighth interval subcarinately elevated above striae 7 apicad the subapical sinuation, and striae 2–4 nearly as deep as the sutural stria 1 on the elytral apex. The male aedeagal median lobe is similar to that observed in males of *Mecyclothorax nimanu* (Figs 30C, D), but the apical expansion is more narrowly rounded, and the apical face more convex. Setal formula 2221; standardized body length 3.4–3.9 mm.

##### Distribution and habitat.

This species is known only from near the summit of Mont Aorai, with all specimens collected between 1750 and 1900 m elevation.

#### 55. Mecyclothorax kokone sp. n.

http://zoobank.org/A24B9174-71E1-4EF9-B284-5548963F95A1

http://species-id.net/wiki/Mecyclothorax_kokone

##### Diagnosis.

This smallest-bodied Tahitian *Mecyclothorax* – standardized body length 3.35 mm – is also diagnosable by the broadly flavous pronotal and elytral margins contrasted with rufopiceous discs, and small, little convex eyes (Fig. 31C); ocular ratio 1.41 (n = 2), ocular lobe ratio 0.75–0.76. The antennae are very short, the segments moniliform, with the antennomere 8 length 1.5× its maximal breadth. Setal formula 2221. *Head* with broadly impressed frontal grooves, sinuously curved toward midline anteriorly, frons broadly convex between groove and antennal base; eye diameter anteriorly defined by antennal articulation crossing 12 ommatidia. *Pronotum* moderately transverse with broad base, MPW/PL = 1.22–1.24, MPW/BPW = 1.45; lateral margin straight anterad obtuse hind angle that is defined by triangular, raised expansion of lateral bead that houses basal pronotal seta articulatory socket, the hind angle therefore little more than a jag in the pronotal margin at the basal seta; median base slightly depressed relative to disc, rugosely wrinkled laterally, ~5 elongate punctures each side along margin with disc; anterior transverse impression shallow, broad, crossed by 3–4 very fine, indistinct wrinkles each side of midline; front angles rounded, not protruded, lateral marginal depression very narrow even at front angles, the edge beaded; laterobasal depression a broad expansion of lateral margin depression, crossed by transverse raised areas that also occur near base of lateral depression. *Elytra* subquadrate, slightly convex medially, flattened behind scutellum; striae 1–7 distinctly impressed, with elongate punctures in basal half, striae 1 + 2, 3, 4, and 5 continuous to basal groove; interval 8 broadly convex laterad stria 7 at elytral apex, only slightly raised above level of interval 7; lateral elytral setae 7 + 6. *Metathoracic wings* assessable due to breakage of left elytron from paratype; wing rudiment triangular with anterior subcostal margin 1.5× length of truncate apical margin, the wing apex extended only to posterior margin of metanotum; alar surface ridged, with unmelanized indications of Sc + R and M veins, and a transverse budge more apically that seems not homologous with a vein as it does not reach the alar base. *Microsculpture* of frons a transverse mesh, sculpticell breadth 2–4× length, with more transverse sculpticells in frontal grooves, sculpticells isodiametric on neck; pronotal disc with shallow transverse mesh, breadth 2–4× length intermixed with transverse lines; elytral disc with well-developed transverse mesh, breadth 2–4× length. *Coloration* of head rufous, more flavous behind eyes and near dark rufous frontoclypeal suture; antennomeres 1–3 flavous, 4–11 rufoflavous; pronotal disc each side of midline rufobrunneous with silvery reflection, lateral margins broadly flavous, anterior callosity and median base rufoflavous; elytral disc rufopiceous with purplish metallic reflection; base and humerus, lateral depression and ninth interval, apex of eighth interval and elytral apex apicad apical seta all flavous; sutural interval broadly rufoflavous basally, paler apically; femora flavous, tibiae flavous with brunneous cast.

Female reproductive tract. Bursa copulatrix relatively elongate, vagina of similar diameter and therefore not discernible (Fig. 33B), bursal length >3.0× breadth compressed under cover slip; basal gonocoxite 1 narrow, with apical fringe of three setae placed laterally, and 6–8 setae lining the medial margin (Fig. 9B); apical gonocoxite 2 narrowly triangular, not extended laterally at base, rightly rounded apically, with two lateral ensiform setae, the basal seta of smaller diameter, and one dorsal ensiform seta; apical sensory furrow with two apical nematiform setae and two furrow pegs.

Holotype female (MNHN) labeled: French Polynesia: Tahiti Nui / Pito Hito el. 2000 m 2-VI- / 2006 lot 02 pyrethrin fog / 17°36.790'S, 149°27.842'W / E.M. Claridge // HOLOTYPE / Mecyclothorax / kokone / J.K. Liebherr 2013 (black-bordered red label).

Paratype: female specimen, reproductive tract dissected and left elytron removed (CUIC, 1).

##### Etymology.

The species epithet kokone is the Tahitian word for diminutive, and it signifies the very small body size of these beetles. Kokone is one of only a few Tahitian words (Wahlroos 2002) to share the k sound with Tahitian’s close linguistic relative, Hawaiian; a fitting connection given the derivation of Tahitian and Hawaiian *Mecyclothorax* biotas from the same Australian stock (Britton 1948; Liebherr 2012a).

##### Distribution and habitat.

This is another species known only from 2070 m elevation near the summit of Pito Hiti. As for the others, beetles of this species have been shown to inhabit arboreal moss on emergent vegetation through sampling using pyrethrin fog.

#### 56. Mecyclothorax castaneus

Perrault, 1986: 455

http://species-id.net/wiki/Mecyclothorax_castaneus

##### Identification.

Among the smaller-bodied members of the *Mecyclothorax viridis* group with elytral striae 1–5 continuously impressed to the elytral basal groove (Fig. 31), this species can be diagnosed by the rufopiceous dorsal body coloration (Fig. 31D) and the well-developed microsculpture: 1, head covered with regular transverse mesh across frons, neck with isodiametric sculpticells; 2, pronotal disc with densely packed transverse microsculpture, consisting of elongate transverse sculpticells and unconnected transverse lines; and 3, elytral discal intervals bearing gratelike transverse microsculpture consisting predominantly of transverse lines, with some areas including crossconnections resulting in an elongate transverse mesh. Summarizing the type series of two specimens (Perrault 1986) plus six more specimens collected in 2006, only the 1986 female paratype unilaterally possesses a posterior dorsal elytral seta, the other elytron bearing only the anterior dorsal seta. Thus the most usual setal formula is 2211, a formula shared with *Mecyclothorax paahonu*, below. The male aedeagal median lobe is gracile and elongate, with an apicodorsal hooklike expansion (Fig. 30E). Standardized body length 3.8–4.1 mm.

##### Distribution and habitat.

In 1977, Perrault (1986) collected two specimens between 900 and 1100 m elevation on Mont Marau. In 2006 that elevational zone had become invaded with *Albizia chinensis* (Fabaceae) and was too dry for successful collecting. That year specimens were collected at 1275–1335 m elevation, through beating soft ferns and mossy *Weinmannia* branches, and via the use of pyrethrin fog on a tangled bank of dead ferns and a horizontal, moss-covered *Weinmannia* log.

#### 57. Mecyclothorax paahonu sp. n.

http://zoobank.org/4610B356-AFD2-41B5-8EAE-4C31D6B4454D

http://species-id.net/wiki/Mecyclothorax_paahonu

##### Diagnosis.

Among the *Mecyclothorax viridis* group species, this is the only one to share the 2211 setal formula with *Mecyclothorax castaneus*, but differing from that species in coloration, with the elytral margins – lateral marginal depression and ninth interval – rufoflavously contrasted to each rufopiceous elytral disc. The rufopiceous disc of each elytron is isolated by the rufoflavous sutural interval (Fig. 31E). The elytral base is paler than the pronotal disc, rufobrunneous versus rufopiceous, and the lateral margins of the pronotum are subparallel for a greater distance anterad the right hind angles (Figs 31D versus E). Standardized body length 4.2 mm. *Head* with frontal grooves narrow posteriorly, linear, curved mesally inside broad convexity near frontoclypeal suture; eyes moderately convex, ocular ratio 1.48, ocular lobe hind margin quite obtuse to genal margin, ocular lobe ratio 0.75; antennae short, segments moniliform, antennomere 8 length 1.56× maximal breadth. *Pronotum* little transverse, MPW/PL = 1.17, base moderately broad, MPW/BPW = 1.47; median base slightly depressed relative to disc, rugosely wrinkled laterally, ~7 elongate punctures along margin with disc; anterior transverse impression broad, shallow, crossed by 8–10 finely inscribed, indistinct longitudinal wrinkles that extend across anterior callosity to anterior margin; front angles tightly rounded, very slightly protruded; lateral marginal depression very narrow, edge beaded, depression slightly wider inside front angles; laterobasal depression a broadened sinuate extension of lateral depression, crossed by transverse raised bars, basally defining a triangular raised area bearing basal seta articulatory socket. *Elytra* narrowly ovate, lateral margins rounded posterad from subangulate humeri; elytral striae 1–6 shallow, with fine irregular elongate punctures that little expand striae, stria 7 shallower, irregularly impressed along length but not punctate; striae 1+ 2, and 3–6 continuously impressed to elytral basal groove; interval 8 more broadly convex laterally near elytral apex than interval 7, but not upraised above stria 7 more than the inner interval; lateral elytral setae 7 + 6. *Microsculpture* of head a well-developed transverse mesh composed of transverse and isodiametric sculpticells in transverse rows, the sculpticells more isodiametric and upraised on neck; pronotal disc with shallow transverse mesh, sculpticells breadth 2–4× length; elytral discal intervals with regular transverse mesh, sculpticell breadth 2–4× length, with crossconnections absent from some limited areas resulting in transverse lines. *Coloration* of head uniformly rufobrunneous; antennomere 1 flavous, 2–3 rufoflavous, 4–11 rufobrunneous; pronotum rufopiceous with purplish reflection, lateral margins narrowly and median base broadly rufobrunneous; rufopiceous discs of each elytron with purplish reflection.

Holotype female (MNHN) labeled: French Polynesia: Tahiti Nui / Pito Hito el. 2070 m 2-VI- / 2006 lot 01 pyrethrin fog / 17°36.813'S, 149°27.842'W / E.M. Claridge // HOLOTYPE / Mecyclothorax / paahonu / J.K. Liebherr 2013 (black-bordered red label).

##### Etymology.

The species epithet paahonu is taken from the Tahitian word for maroon, a rough approximation of the dorsal body coloration of the holotype.

##### Distribution and habitat.

Known from a pyrethrin fog sample of moss-covered vegetation taken 40 m elevation below the summit of Pito Hiti.

#### 58. Mecyclothorax kayballae sp. n.

http://zoobank.org/958D4FB7-4D06-47B7-A4DA-A7D1C1D3B3CE

http://species-id.net/wiki/Mecyclothorax_kayballae

##### Diagnosis.

Of the *Mecyclothorax viridis* group species with evident transverse elytral microsculpture and basally obsolete elytral striae 1–5 (Fig. 32), this species uniquely lacks dorsal elytral setae; setal formula 2201. The dorsal coloration is dark, rufopiceous with a blue reflection (Fig. 32A), much darker than all other species in the group save *Mecyclothorax ninamu* (Fig. 31A), but beetles of that species are much smaller – 3.5–4.0 mm – versus those of *Mecyclothorax kayballae*; standardized body length 4.3–4.7 mm. *Head* with frontal grooves very shallow posteriorly, separated from anterior supraorbital seta by fine, low carina, frons mesally with radiating wrinkles, groove broadly, triangularly depressed anteriorly near frontoclypeal suture; eyes moderately convex, ocular ratio 1.49–1.50 (n = 3), ocular lobe protruded, posterior margin of lobe meeting gena at shallow groove with carina behind, ocular lobe ratio 0.80–0.83; antennae submoniliform, antennomere 8 length 1.80× maximal breadth. *Pronotum* cordate, base narrow, MPW/BPW = 1.61–1.63, moderately transverse, MPW/PL = 1.20–1.24; hind angles right to slightly obtuse, lateral margins convergent immediately anterad angle then distinctly divergent; median base convex medially, moderately depressed relative to convex disc, 12–14 distinct punctures each side plus 5–7 elongate wrinkles along margin with disc; anterior transverse impression finely incised laterally with fine wrinkles behind, obsolete near midline; front angles rounded, slightly protruded, lateral marginal depression narrow with beaded edge laterally, depression broader with less upraised margin at front angle; laterobasal depression a narrow, punctate expansion of lateral depression, defining laterally a triangular raised area bearing the basal seta articulatory socket. *Elytra* ovate, convex, scutellum depressed relative to disc, sides sloping to near vertical at lateral elytral depression; humeri proximate, but elytral margins extended laterally in a broad even curve posteriorly, MEW/HuW = 2.41–2.51; striae 1–6 shallow with distinct rounded punctures on disc, striae much shallower and impunctate near basal groove, appearing obsolete in dorsal view but traceable if basal surface of elytra is positioned horizontally in field of view; stria 7 very shallow with periodic shallow dimples along length; humeri angulate, elytral basal groove more distinctly curved laterally near juncture with lateral marginal depression; lateral marginal depression narrow throughout length, only slightly broader near midlength; elytral striae 2–7 reduced over much of elytral apex, the apex of stria 7 impressed apicad subapical sinuation, with interval 8 more convex at that point resulting in a short, subcarinate ridge adjacent to stria 7; elytral lateral setae 7 + 6. *Microsculpture* of frons transverse, obsolete, the surface glossy between areas with visible sculpticells, neck with more visible transverse microsculpture, the rows including isodiametric and transverse sculpticells; pronotal disc with regular, shallow transverse mesh, sculpticell breadth 2–3× length; discal elytral intervals covered with a mixture of transverse sculpticells, sculpticell breadth 3× length, and transverse lines. *Coloration* of frons and vertex rufopiceous, clypeus and labrum rufobrunneous; antennomeres 1–4 rufoflavous, 5–11 with brunneous cast; pronotal disc rufopiceous, lateral marginal depressions narrowly rufobrunneous, median base dark rufous; elytral disc rufopiceous in basal half, rufous in apical half, sutural interval paler, rufobrunnous only near apex; lateral marginal depression narrowly rufobrunneous; femora and tibiae contrasted with body coloration, rufoflavous.

Male genitalia. Aedeagal median lobe distinctly dorsally curved, apex very short, the tip subacuminate (Fig. 30F).

Female reproductive tract. Bursa copulatrix and vagina separated by a distinct constriction (Fig. 6A), length of bursa plus vagina slightly more than twice bursal breadth compressed under cover slip; basal gonocoxite 1 elongate, with apical fringe of four setae, three larger setae laterally and one smaller, isolated seta medially, 5–6 setae along mesal margin and several more on ventral face (Fig. 9C); apical gonocoxite 2 broadly extended laterally at base, apex subacuminate, lateral margin broadly arcuate, with two lateral ensiform setae, the apical seta broadened along shaft, one dorsal ensiform seta; apical sensory furrow with two apical nematiform setae and two furrow pegs.

Holotype male (MNHN) labeled: French Polynesia: Tahiti Nui / Mt. Marau road el. 1125 m / 4-IX-2006 lot 01 / 17°36.437'S, 149°33.110'W / beat dead tree fern fronds / wet rock face J.K. Liebherr // 1 // HOLOTYPE / Mecyclothorax / kayballae / J.K. Liebherr 2013 (black-bordered red label).

Paratypes: same data as holotype (CUIC, 2). The paratypes have been dissected for male genitalic and female reproductive tract description.

##### Etymology.

This species is named in honor of Dr. Kathleen Ball for her support for and active engagement in the carabidological community. It should be noted that *Mecyclothorax kayballae* and *Mecyclothorax balli*, named by Georges Perrault in honor of Professor George Ball, co-inhabit Mont Marau, establishing the possibility of collecting both species during a one day’s trip to the summit.

##### Distribution and habitat.

This species is known from the type series collected at 1125 m elevation on Mont Marau. The beetles were beaten from dead fern fronds that were associated with living plants growing on a wet rock face.

#### 59. Mecyclothorax balli

Perrault, 1978b: 147; 1986: 454

http://species-id.net/wiki/Mecyclothorax_balli

##### Identification.

Among the *Mecyclothorax viridis* group species with basally reduced elytral striae and two dorsal elytral setae (Figs 32B–E), diagnosable by the very transverse elytral microsculpture composed of transverse lines with little development of a mesh, and larger standardized body length, 4.8–5.1 mm. From the smaller bodied *Mecyclothorax putaputa*, with which this species shares the very transverse elytral microsculpture, this species can be differentiated by the deep, distinctly punctate discal elytral striae and associated convex intervals (Figs 32B), and the distinctly sinuate lateral pronotal margins and projected pronotal hind angles. The vertex of the head is glossy with the shallow to obsolete transverse microsculpture partially to totally obscured by irregular transverse wrinkles. The pronotal disc is covered with an obsolete (male paratype) to shallow (female paratype) elongate transverse mesh mixed with transverse lines, the defined sculpticells 2–4× broad as long. The male aedeagal median lobe has an elongate, parallel-sided apex with oblique apical face (Fig. 30G); the elongate apex a shortened version of the exaggeratedly elongate apex observed in males of *Mecyclothorax ata* (Fig. 30H). Setal formula 2221.

##### Distribution and habitat.

All that is known about the biology and distribution of this species is restricted to the 1976 collection of the eight-member type series from 1300–1400 m elevation on Mont Marau (Perrault 1978b).

#### 60. Mecyclothorax putaputa

Liebherr, 2012b: 79

http://species-id.net/wiki/Mecyclothorax_putaputa

##### Identification.

This species shares with *Mecyclothorax balli* the setal formula 2221, effaced elytral striae near the elytral basal groove, and transverse-line elytral microsculpture, but these beetles are smaller, standardized body length 4.3–4.5 mm versus 4.8–5.1 mm for *Mecyclothorax balli*. The pronotal hind angles are marked by a large transverse fault-like hitch in the pronotal margin, with the margin before and after the hitch of nearly the same curvature (Fig. 32C). The elytral striae are moderately shallow with elongate punctures that impress the stria in a dashed pattern. The head bears distinct transverse mesh microsculpture mixed with transverse lines, the defined sculpticells only slightly broader than long, and the pronotal disc is covered with a shallow but evident transverse mesh, sculpticell breadth 2–3× length.

##### Distribution and habitat.

This species is so far known to be associated with low-stature *Metrosideros* and *Weinmannia* forest from 1065–1100 m elevation on Mont Mauru. The two specimens were in subarboreal microhabitats from which they were beaten onto a sheet or collected through the use of pyrethrin fog.

#### 61. Mecyclothorax ata

Perrault, 1978b: 148; 1986: 454

http://species-id.net/wiki/Mecyclothorax_ata

##### Identification.

Among the *Mecyclothorax viridis* group, this species comprises narrow-bodied beetles with subquadrate elytra and a cordate pronotum, the pronotal hind angles right and the basal margins preceding them angularly convergent (Fig. 32D). The elytral striae are deep though finely inscribed, with rounded punctures that expand the strial breadth. As opposed to the preceding two species (Figs 32B, C), this species is characterized by a very well-developed transverse mesh on the discal elytral intervals, the sculpticells raised and varying from isodiametric to transverse with breadth 2× length. The frons is glossy in part with larger transverse wrinkles, an intermittent transverse mesh near the frontoclypeal suture and a more regular transverse mesh on the neck. The pronotal disc is covered by a shallow transverse mesh, sculpticell breadth 3–4× length, with the mesh obscured by reflected light. The male aedeagal median lobe is very characteristic, with a narrowly elongate apex and oblique chisel-like tip defined by the oblique apical face (Fig. 30H). Setal formula 2221; standardized body length 4.4–4.9 mm.

##### Distribution and habitat.

This species is known from the higher reaches of Mont Aorai, with the male holotype and female allotype collected near Fare Ata, 1900 m elevation.

#### 62. Mecyclothorax ehu sp. n.

http://zoobank.org/84D4A2A5-455B-4FEC-BF16-D65BD5D2AE3F

http://species-id.net/wiki/Mecyclothorax_ehu

##### Diagnosis.

This species shares with *Mecyclothorax putaputa* (Figs 32C, E) the little projected, hitch-like pronotal hind angles with pronotal lateral margins subparallel only outside the basal seta articulatory socket, but individuals of this species are larger, standardized body length 4.7–5.1 mm. The elytral striae are shallow with distinct rounded punctures on the disc that expand the breadth of the striae. The discal elytral intervals are only slight convex. Setal formula 2221. *Head* with laterally linear frontal grooves, posteriorly lined mesally with fine transverse wrinkles and bordered laterally by fine carina, moderately expanded anteriorly, with two grooves divergent at frontoclypeal suture; eyes convex on protruded ocular lobes, ocular ratio 1.53–1.62 (n = 5), ocular lobe ratio 0.86–0.90; antennae somewhat elongate, submoniliform, antennomere 8 length 1.8× maximal breadth. *Pronotum* variably broad, moderately transverse to transverse, MPW/PL = 1.14–1.28, base similarly, variably constricted, MPW/BPW = 1.47–1.64; median base evenly depressed relative to convex disc, ~12 distinct punctures each side; anterior transverse impression broad, shallow, continuous across breadth, crossed laterally by ~9 fine longitudinal wrinkles that extend anterad across anterior callosity; front angles not protruded, rounded behind; lateral marginal depression narrow, margin upraised but not beadlike, depression slightly wider inside front angles; laterobasal depression an elongate expansion of lateral marginal depression, crossed near base by transverse bar that connects median base to the upraised lateral margin. *Elytra* ovate, lateral margin evenly curved posteriorly outside subangulate humerus, the humeri moderately proximate, MPW/HuW = 2.22–2.32; elytral disc moderately convex, sutural interval elevated relative to striae 2–6; striae 1–6 shallow to obsolete near elytral basal groove and humerus; apicad base, stria 6 shallower than striae 1–5, but still continuous, stria 7 interrupted through much of length, consisting of a series of isolated punctures except for deep canaliculated portion apicad fused terminus of striae 5 + 6; eighth interval increasingly more convex as it narrows near apex, appearing pinched, the mesal margin upraised and subcarinate along stria 7 near apex; lateral elytral setae 7 + 6. *Microsculpture* of frons an elongate transverse mesh, sculpticell breadth 2–4× breadth, the sculpticells visible even in transverse wrinkles, neck with sculpticells 2× broad as long; pronotal disc with indistinct transverse mesh visible outside areas of reflected light, sculpticell breadth 2–3× length; discal elytral intervals lined with regular transverse mesh, the sculpticells distinctly bordered, sculpticell breadth 2–4× length. *Coloration* of head rufous; antennomere 1 flavous, 2–3 rufoflavous, 4–11 rufobrunneous; pronotal disc rufobrunneous, margins narrowly paler, rufous; elytral disc rufous with a silvery reflection, sutural interval narrowly paler near scutellum, rufoflavous apically; elytral lateral marginal depression concolorous at humerus, contrastedly rufoflavous anterad subapical sinuation; femora rufoflavous, tibiae rufoflavous with brunneous cast.

Male genitalia. Aedeagal median lobe shaft broad, apex moderately extended beyond ostium and broad dorsoventrally (Fig. 30I), the apical face moderately oblique; internal sac with broad, diffuse ventral ostial microtrichial patch (Fig. 30J), flagellar plate elongate, length 0.5× distance from parameral articulations to apical face; dorsal surface of flagellar plate with a broad median depression that includes gonopore, lateral reaches of dorsal membrane associated with plate lightly sclerotized.

Female reproductive tract. Bursa copulatrix ovately expanded apicad a slightly narrower vagina; bursal plus vaginal length twice as long as maximal breadth when compressed under cover slip (Fig. 33C); basal gonocoxite 1 narrow, elongate, with apical fringe of 4 setae, the middle pair doubled, 5–7 smaller setae along mesal margin, 1–3 of these near apex (Fig. 9D); apical gonocoxite 2 very broadly expanded laterobasally, the lateral margin distinctly arcuate laterad the tightly rounded apex, two lateral ensiform setae and one dorsal ensiform seta, the latter extended laterally past lateral margin; apical sensory furrow with two apical nematiform setae and two sensory pegs.

Holotype male (MNHN) labeled: French Polynesia: Tahiti Nui / Pito Hito el. 2070 m 2-VI- / 2006 lot 01 pyrethrin fog / 17°36.813'S, 149°27.842'W / E.M. Claridge // HOLOTYPE / Mecyclothorax / ehu / J.K. Liebherr 2013 (black-bordered red label).

Allotype female (MNHN) labeled as holotype.

Paratypes: labeled as holotype (CUIC, 1; EMEC, 1); Pito Hiti, 2000 m el., 17°36.790'S, 149°27.842'W, 2-vi-2006 lot 02, Claridge, pyrethrin fog.

##### Etymology.

The species epithet ehu is taken from the Tahitian word 'ehu, meaning the color red. The name signifies the reddish coloration of beetles of this species.

##### Distribution and habitat.

This species is known from 2000-2070 m elevation on Pito Hiti, and based on collections made using pyrethrin fog, spends at least part of its life within arboreal mossmats.

### 8. *Mecyclothorax gourvesi* species group

**Figure 34.**
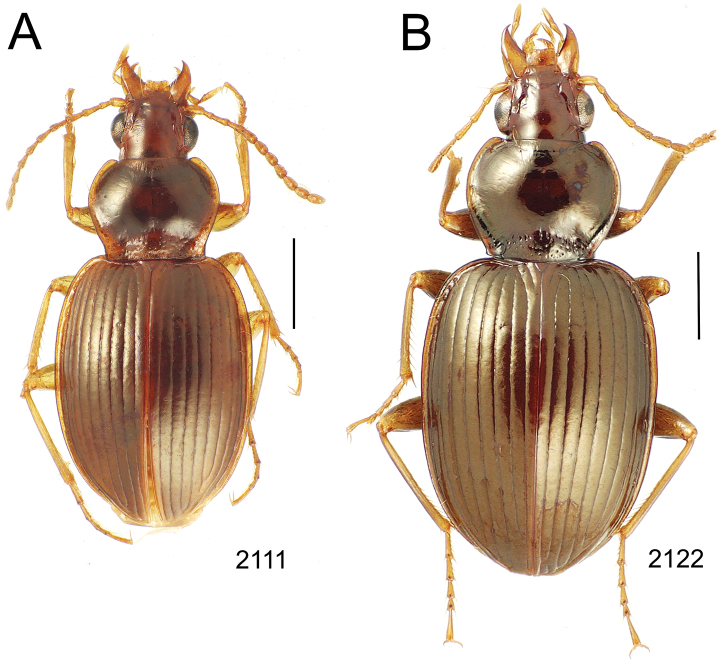
*Mecyclothorax* spp., dorsal view; scale bars 1.0 mm; setal formula (see Fig. 10) at lower right of each figure **A** *Mecyclothorax zimmermani* holotype male (BPBM) **B** *Mecyclothorax acutangulus* holotype male.

**Figure 35.**
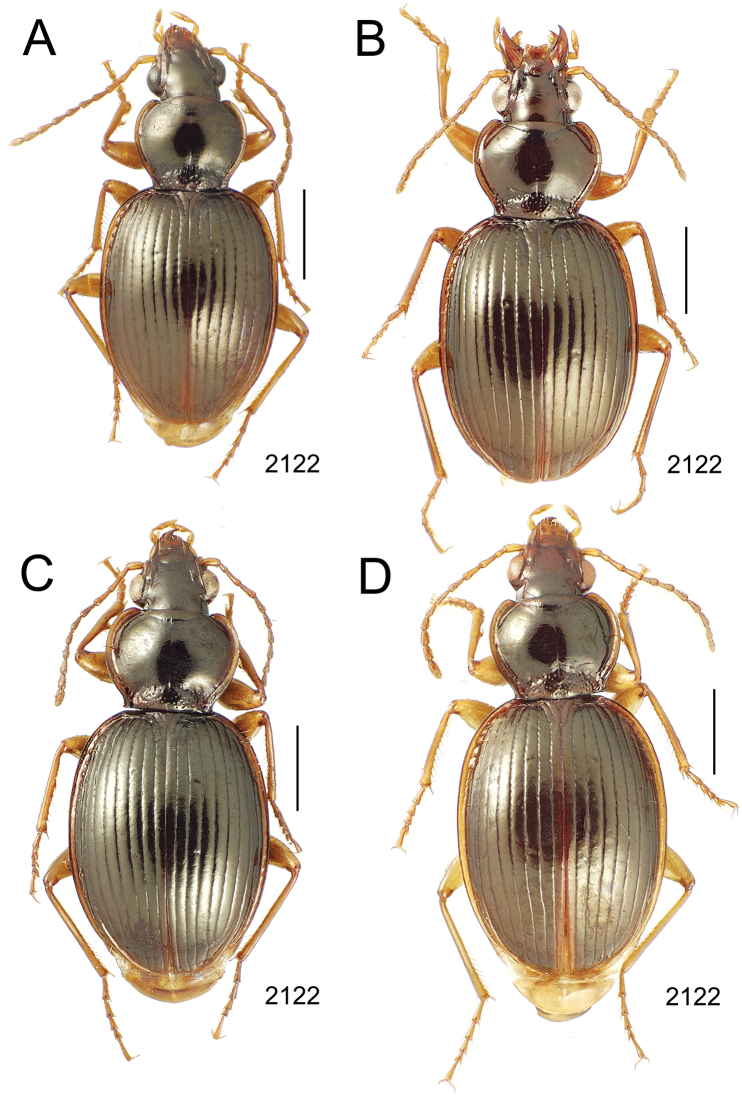
*Mecyclothorax* spp., dorsal view; scale bars 1.0 mm; setal formula (see Fig. 10) at lower right of each figure **A** *Mecyclothorax papuhiti* holotype male **B** *Mecyclothorax gourvesioides* paratype male (MNHN) **C** *Mecyclothorax gourvesi*, Marau, 1125 m el. **D** *Mecyclothorax gourvesi*, Marau, 1335 m el.

**Figure 36.**
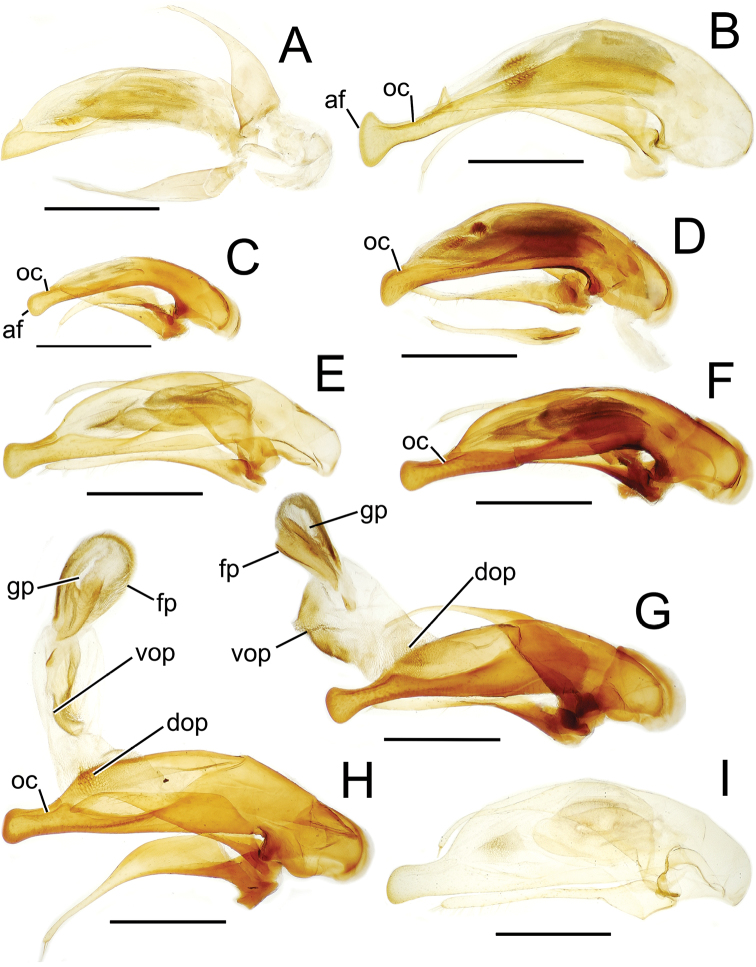
Male aedeagal median lobe and associated parameres, *Mecyclothorax* spp., right lateral view; scale bars 0.5 mm **A** *Mecyclothorax zimmermani* holotype **B** *Mecyclothorax acutangulus* holotype **C** *Mecyclothorax papuhiti* holotype **D** *Mecyclothorax gourvesioides* paratype (MNHN) **E** *Mecyclothorax gourvesi*, Marau **F** *Mecyclothorax gourvesi*, Marau **G** *Mecyclothorax gourvesi*, Marau, internal sac everted **H** *Mecyclothorax gourvesi*, Aorai, internal sac everted **I** *Mecyclothorax gourvesi*, Aorai, teneral Abbreviations: **af** apical face **dop** dorsal ostial microtrichial patch **fp** flagellar plate **gp** gonopore **oc** ostial canal **vop** ventral ostial microtrichial patch

**Diagnosis.** Species of this group are characterized by the broad, translucent pronotal lateral margin that is sinuate anterad the denticulate, right to slightly obtuse hind angle (Figs 34, 35). The pronotum is bisetose; the hind angles glabrous. The elytral lateral marginal depression is also explanate outside the anterior series of lateral elytral setae, the explanate translucent border contrasted to the brunneous to rufopiceous elytral disc. The male aedeagus takes two basic conformations; with an apically blunt median lobe (Figs 36A, C), or with a spoonlike, dorsoventrally and symmetrically expanded apex (Figs 36B, D–I). *Mecyclothorax perraulti* Liebherr (2012a) of Moorea is also a member of this group. Standardized body lengths 4.7–6.2 mm.

#### Identification key to Tahitian species of the *Mecyclothorax gourvesi* species group

**Table:** 

1	Elytral third interval with two dorsal setae, setal formula 2122 or 2121	2
–	Elytral third interval with one dorsal seta before midlength (Fig. 34A), apical elytral seta present near apex of stria 2, subapical elytral seta absent, setal formula 2111 (Aorai)	63. *Mecyclothorax zimmermani* Perrault
2	Pronotal lateral margins distinctly subparallel or convergent anterad projected hind angles which are right to acute; body narrower, elytral basal margins sloping posteriorly immediately laterad humeral angle	3
–	Pronotal lateral margins subparallel for very short distance anterad little projected hind angles which are slightly obtuse; body broad, elytral basal margins extended laterally outside humeral angle before anterior series of lateral elytral setae (Fig. 34B) (Teatara)	64. *Mecyclothorax acutangulus* Perrault
3	Elytral lateral marginal depressions very broad, explanate and translucent from humeral angle posterad past anterior series of lateral elytral setae (Figs 35B–D), the depressed surface broadly concave with margin evenly elevated, margin not beaded	4
–	Elytral lateral marginal depression narrower just laterad humeral angle than posterad outside anterior series of lateral elytral setae (Fig. 35A), surface not translucent near humeral angle, the margin narrowly elevated (Marau)	65. *Mecyclothorax papuhiti* sp. n.
4	Eighth elytral interval broadly convex from middle of posterior series of lateral elytral setae to elytral apex, convexly raised laterally outside stria 7, never carinate; elytral lateral marginal depression of even breadth near bases of elytral striae 5–7 laterad humerus; elytra broadly ovate (Fig. 35B) (Pito Hiti)	66. *Mecyclothorax gourvesioide* s Perrault
–	Eighth elytral interval raised above stria 7 from lateral elytral setae to elytral apex, the interval either flat laterally and sharply carinate, or more broadly convex and dorsally subcarinate; elytra narrowly ovate (Figs 35C, D) (Aorai, Marau)	67. *Mecyclothorax gourvesi* Perrault

#### 63. Mecyclothorax zimmermani

Perrault, 1978b: 145; 1984: 31

http://species-id.net/wiki/Mecyclothorax_zimmermani

##### Identification.

Beside the presence of only the anterior elytral seta, and therefore setal formula 2111, this species is set off from the other members of the *Mecyclothorax gourvesi* group by the basally broad pronotum; MPW/BPW = 1.32 (Fig. 34A). The head, frons, and vertex, are covered with a well-developed isodiametric mesh, the sculpticells in more transverse rows amongst the wrinkles of the frons, regularly isodiametric on the neck. The pronotal disc is covered with a regular transverse mesh, sculpticell breadth 2× length, and the discal elytral intervals are lined with a well-developed transverse mesh with sculpticells 3–4× broad as long. The male aedeagal median lobe has a very broad, very short apex with an acuminate tip at the juncture of the apical face and ventral margin (Fig. 36A). Standardized body length 5.2 mm.

##### Distribution and habitat.

The unique holotype specimen was collected by Elwood Zimmerman between 1675 and 1900 m elevation on Mont Aorai. The specimen was beaten from moss on either a tree or a shrub.

#### 64. Mecyclothorax acutangulus

Perrault, 1988: 241

http://species-id.net/wiki/Mecyclothorax_acutangulus

##### Identification.

This species shares the expanded pronotal and elytral lateral margins of other members of the *Mecyclothorax gourvesi* group (Fig. 34B), but deviates by the larger body size, standardized body length 6.2 mm. The elytra are broadly extended laterad from the humeri, moreso even than in *Mecyclothorax gourvesioides* (Fig. 35B), resulting in broadly quadrate elytra. The head surface is glossy, with only small micropunctures – i.e., pore canals – disturbing the smooth surface. An obsolete transverse mesh, sculpticell breadth 3–4× length, is visible on the pronotal disc, best seen in areas partially in shadow from illumination due to the curvature of the surface; micropunctures also visible across the pronotal surface. The discal elytral intervals are covered with elongate transverse microsculpture consisting of a mixture of transverse sculpticells, breadth 2–4× length, and transverse lines. The male aedeagal median lobe is broadly expanded both dorsally and ventrally forming a spoon-shaped apex with a broadly convex apical face (Fig. 36B), a configuration that exaggerates the apical expansion of the median lobe in males of *Mecyclothorax gourvesi* (Figs 36E–I). The setal formula 2122 is shared with both *Mecyclothorax gourvesioides* and *Mecyclothorax gourvesi*.

##### Distribution and habitat.

This Tahiti Iti representative of the species group is known from specimens collected 900–1100 m elevation on Mont Teatara. A teneral female specimen was obtained by beating *Freycinetia* plants with rotten leaves, upon some of which lay *Cyathea* tree fern fronds.

#### 65. Mecyclothorax papuhiti sp. n.

http://zoobank.org/FEC59D44-2A8E-4EDA-B58E-3375E2DB9F69

http://species-id.net/wiki/Mecyclothorax_papuhiti

##### Diagnosis.

Among the five species currently known to comprise this group, this one has the least transverse pronotum (Fig. 35A), MPW/PL = 1.18, in company with evenly explanate lateral margins that are narrower than those characterizing *Mecyclothorax gourvesioides* and *Mecyclothorax gourvesi* (Figs 35B–D). Also, though the elytral lateral margin is broad outside the anterior series of lateral elytral setae, it is narrow and opaque immediately laterad the humeral angle. The male holotype of this species is also smaller than individuals of the other species in the group; standardized body length 4.8 mm versus 5.0–6.2 mm for specimens of the other four species. Setal formula 2122. *Head* with deep, canaliculate frontal grooves, posteriorly bordered laterally mesad eye by robust, convex carina, anteriorly bordered laterally by broadly sinuous convexity; eyes convex on protruded ocular lobe, ocular ratio 1.52, ocular lobe meeting gena at distinct groove, ocular lobe ratio 0.82; antennae elongate, segments filiform, antennomere 8 length 2.0× maximal breadth. *Pronotum* with distinctly sinuate lateral margins before nearly right hind angles, the basal margin slightly convex behind laterobasal depression; median base moderately depressed relative to disc, with ~24 small but distinct punctures on each side, the punctures separated by smooth cuticle; anterior transverse impression obsolete medially, sharply incised in lateral 2/3 of each side, without longitudinal wrinkles; front angles slightly protruded anteriorly, broadly rounded; lateral margins broadly and evenly upturned, slightly broader inside front angles; laterobasal depression a deep, linear extension of the deepest portion of lateral marginal depression, with about three indistinct, irregularities mesad lateral sinuation. *Elytra* broadly ovate, lateral margins curved posteriorly outside obtuse-angulate humeri; disc convex relative to depressed scutellum and lateral margins; striae 1–5 well defined to basal groove, distinctly punctate on disc, the round punctures expanding strial breadth, stria 6 shallower with punctures more elongate, and stria 7 shallower still, interrupted in places with punctures forming elongate dashes; interval 8 convex, bulging laterally along posterior series of lateral elytral setae to its apex, its mesal margin forming a long subcarinate ridge adjacent to stria 7; lateral elytral setae 7 + 6. *Microsculpture* absent on frons and vertex, the cuticle glossy; pronotal disc with obsolete transverse mesh microsculpture, sculpticell breadth 2–3× length, the sculpticells visible only near edges of fields of reflected light; discal elytral intervals lined with evident transverse lines intermixed with areas of transverse mesh, sculpticell breadth 3× length. *Coloration* of frons and vertex dark rufous, clypeus rufous, labrum rufoflavous; antennomere 1 flavous, 2–3 rufoflavous, 4–11 rufobrunneous; pronotal disc slightly darker than head, base rufous and lateral margins rufoflavous; elytral disc concolorous with head, sutural interval rufous basally, rufoflavous apically; elytral lateral marginal depression and apices of elytral intervals 7–9 near posterior lateral elytral setae rufoflavous, inner intervals also rufoflavous beyond level of subapical sinuation.

Male genitalia. Aedeagal median lobe shaft narrow and of even diameter from parameral articulations to apex of ostium, barely narrower apically with an evenly proportioned dorsoventrally spatulate apex (Fig. 36C); ostial canal short and close to dorsal margin of lobe; internal sac not everted, but spiculate fields not evident inside lobe shaft.

Holotype male (MNHN) labeled: French Polynesia: Tahiti Nui / Mt. Marau road el. 1125 m / 4-IX-2006 lot 01 / 17°36.437'S, 149°33.110'W / beat dead tree fern fronds/ / wet rock face J.K. Liebherr // HOLOTYPE / Mecyclothorax / papuhiti / J.K. Liebherr 2013 (black-bordered red label).

##### Etymology.

The species epithet pahuhiti compounds the Tahitian words pāpū, meaning even, with hiti, meaning border or edge, the species name signifying the evenly explanate pronotal lateral margins.

##### Distribution and habitat.

The single known specimen was collected in a beating sample along with one specimen of *Mecyclothorax gourvesi* and three specimens of *Mecyclothorax kayballae*. These microsympatric collections from dead tree fern fronds along a wet rock face demonstrate that: 1, *Mecyclothorax pahuhiti* shares ecological space with the more abundant and geographically widespread species group member, *Mecyclothorax gourvesi*; and 2, sampling a wet rock face and associated vegetation resulted in the discovery of two new species. Neither newly described species has been found at any other time in any other ecological situation. Additionally, specimens of three other species – *Mecyclothorax ballioides*, *Mecyclothorax globosus*, and *Mecyclothorax ovalipennis* – were also present in this sample, demonstrating that a diverse array of *Mecyclothorax* species can occupy this microhabitat at a single site.

#### 66. Mecyclothorax gourvesioides

Perrault, 1988: 244

http://species-id.net/wiki/Mecyclothorax_gourvesioides

##### Identification.

Most similar to *Mecyclothorax papuhiti* and *Mecyclothorax gourvesi* in the sinuate pronotum with broadly explanate lateral margins (Fig. 35) and setal formula 2122, but distinguished by the very distinctly punctate elytral striae and laterally pinched, very convex eighth elytral interval, the convex roll extended from inside the middle of the posterior series of the lateral elytral setae to the elytral apex. The elytral marginal depression is also very broad near the humerus in these beetles versus narrower in individuals of the other two species At standardized body length 5.0–5.2 mm, this species exhibits larger body size than *Mecyclothorax papuhiti*. The surfaces of the frons and vertex are superficially glossy, with an obsolete transverse mesh visible outside fields of reflected light. Isodiametric and transverse sculpticells arranged in transverse rows are more visible near the pronotum. The pronotal disc is covered with a shallow but regular transverse mesh, sculpticell breadth 2–3× length, and the discal elytral intervals bear transverse lines that are joined over limited portions of their surface into an elongate transverse mesh. The male aedeagal median lobe is robust, with a broad shaft and briefly extended apex that is ventrally expanded (Fig. 36D). Of this configuration, the short apex is shared with *Mecyclothorax papuhiti* (Fig. 36C), and the broad shaft is shared with *Mecyclothorax gourvesi* (Figs 36E–I).

##### Distribution and habitat.

This species is known from lower elevations north of Mapura to Mapura summit, on the ridge that leads upward to Pihaaiateta and Pito Hiti. Specimens were collected during 1978 between 900 and 1200 m elevations by beating ferns (Perrault 1988). The area within which this species has been collected includes the upper reaches of the Valle de Opearahi that has been developed into the Supermahina housing estate. Supermahina was one of the first areas in Tahiti known to be infested with the little fire ant, *Wasmannia auropunctata*, introduced at some time before 2004 (I.P.P.C. 2005). Thus a portion of the known range of this species has witnessed an alien ant invasion suggesting that a post-invasion assessment of populations of this species is warranted.

#### 67. Mecyclothorax gourvesi

Perrault, 1978b: 146; 1988: 244

http://species-id.net/wiki/Mecyclothorax_gourvesi

##### Identification.

From the most similarly appearing *Mecyclothorax gourvesioides*, this species can be diagnosed by the convexity of the eighth elytral interval taking the form of a carina adjacent to stria 7, not an elongate, more broadly convex roll. The elytra are also narrower in this species, with the basal margins less extended laterally outside the humeral angles. At standardized body length 5.2–5.7 mm, individuals of this species are also larger than those of the former species. The head is glossy, with no indication of microsculpture on the frons or vertex, and only a narrow band of transverse-mesh microsculpture near the margin with the pronotum. The pronotal disc is covered with an obsolete transverse mesh, the sculpticells only visible outside areas of reflected light; sculpticell breadth 2× length. The discal elytral intervals are lined with a well-developed transverse mesh, sculpticell breadth 2–3× length. The male aedeagal median lobe has a robust, broad shaft, and is narrowly extended beyond the ostium to a spatulate, dorsoventrally expanded apex (Figs 36E–I). The aedeagal internal sac bears a broad, diffuse ventral ostial microtrichial patch, a broadly spiculate dorsal ostial microtrichial patch, and a moderately elongate flagellar plate, length 0.43–0.47× distance from parameral articulations to apical face (Figs 36G, H). This and *Mecyclothorax acutangulus* (Fig. 36B) are the two species in the group characterized by a spatulate median lobe apex, though the apex is not nearly so expanded here. Setal formula 2122.

##### Distribution and habitat.

This species is known from both Mont Marau and Mont Aorai; from 1125–1400 m elevation on the former, and 1210–1400 m on the latter. Various male specimens were dissected to verify conspecificity of populations on the two ridge systems, with observed variation precluding division into two species. There is variability in the dorsoventral expansion of the median lobe apex, with two specimens from Marau exhibiting a narrow apical extension and narrowly spatulate apex (Fig. 36F) to broadly spatulate apex (Fig. 36G). A third Marau specimen exhibits a broader apical extension with moderately expanded apex (Fig. 36E), a conformation shared with a specimen from Aorai (Fig. 36H). Finally, a fifth specimen from Mont Aorai has a very broad apical extension with moderately expanded apex (Fig. 36I), though the teneral nature of the specimen may have influenced its conformation when temporarily slide-mounted for photography.

Specimens have been collected from the rotten cambial layer under the bark of a dead *Reynoldsia* tree. They have been collected by beating *Dicranopteris* ferns, other soft ferns, or dead *Cyathea* tree fern fronds. Several were obtained through the use of pyrethrin fog applied to moss-covered *Metrosideros*, horizontal moss-covered logs, and banks of dead ferns. And two specimens were collected in a Malaise trap, indicating a penchant for climbing.

### 9. *Mecyclothorax tuea* species group

**Diagnosis.** This group comprises a single anatomically isolated species from Pito Hiti, uniquely diagnosable by the very broad elytral base with nearly right-angled humeri, and the narrowly margined pronotum that is broadly rectangular behind.

#### 68. Mecyclothorax tuea sp. n.

http://zoobank.org/D48A5702-4BD9-4954-AD1D-9001D278E71F

http://species-id.net/wiki/Mecyclothorax_tuea

##### Diagnosis.

**Figure 37.**
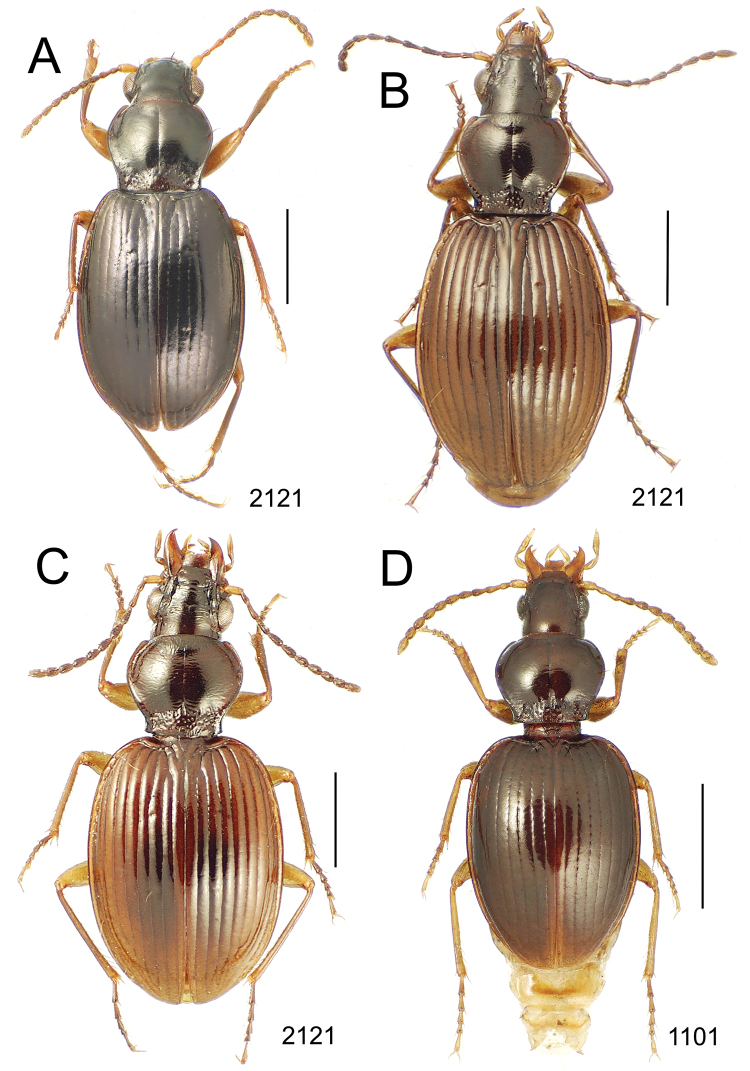
*Mecyclothorax* spp., dorsal view; scale bars 1.0 mm; setal formula (see Fig. 10) at lower right of each figure **A** *Mecyclothorax tuea* holotype female **B** *Mecyclothorax taatitore* paratype male (CUIC) **C** *Mecyclothorax georgettae* paratype female (MNHN) **D** *Mecyclothorax konemata* holotype female

This species is characterized by a narrow pronotum, MPW/PL = 1.14, pronotal lateral margins parallel for 0.10× pronotal length anterad the slightly obtuse hind angles, and the broadly based elytra, the humeri extended laterally, and the lateral elytral margins distinctly curved posteriorly outside the humeral angles (Fig. 37A). The body surface is distinctly microsculptured, with: 1, the head covered with an upraised transverse mesh on the frons, the sculpticells more isodiametric near pronotum; 2, the pronotal disc with well-developed transverse mesh, sculpticell breadth 2–3× length, plus the pronotal median base with evident microsculpture among the punctures, the sculpticells a mixture of isodiametric and transverse; and 3, the discal elytral intervals with an upraised transverse mesh, sculpticell breadth 2× length, the microsculpture associated with a purplish metallic reflection. Setal formula 2121; standardized body length 4.5 mm. *Head* with broad, shallow frontal grooves, defined laterally by fine carina inside anterior supraorbital seta, irregularly shaped and shallow anteriorly; eyes moderately convex, ocular ratio 1.48, ocular lobes moderately protruded, joined to gena at narrow groove, ocular lobe ratio 0.80; antennae moderately short, submoniliform, antennomere 8 length 1.60× maximal breadth. *Pronotum* narrow, median base moderately depressed relative to moderately convex disc, the surface of the median base irregular due to ~9 isolated punctures each side, ~7 short strigae lining margin with disc, and well-developed microsculpture; anterior portion of medial depression and medial portions of anterior transverse impression very broadly and shallowly depressed, the anterior portion of disc appearing flat; lateral reaches of anterior transverse impression not distinct inside protruded, tightly rounded front angles; lateral marginal depression narrow but not deep, margin beaded; a broad shallow oblique depression extended from lateral pronotal seta posteromedially to median base; laterobasal depression irregularly defined by variously raised portions of the lateral and basal margins. *Elytra* quadrate, the humeri abacoid, MEW/HuW = 1.85; medial surface of disc flat between fourth elytral intervals, though scutellum to sutural interval raised above discal surface; elytral striae 1–6 shallow, distinctly punctate though the punctures are elongate and do not greatly expand strial breadth, stria 7 shallow, irregularly impressed along length, interrupted dorsad posterior series of lateral elytral seta, then deeper and continuous mesad eighth elytral interval that is carinately raised above the stria apicad the elytral subapical sinuation, surface of eighth interval obliquely flattened laterally; lateral elytral setae 7 + 6. *Coloration* of head and pronotal disc rufopiceous, clypeus and pronotal base and apex rufous, depression inside pronotal front angles rufoflavous; antennomere 1 flavous, 2–3 rufoflavous, 4–11 rufobrunneous; elytral disc rufopiceous with purplish metallic reflection, scutellum and parascutellar striole slightly paler, rufobrunneous, lateral marginal depression narrowly rufoflavous; femora and elytral epipleuron contrastedly rufoflavous, tibiae rufoflavous with base darker, more rufous.

Holotype female (MNHN) labeled: French Polynesia: Tahiti Nui / Pito Hiti el. 2070 m 2-VI- / 2006 lot 01 pyrethrin fog / 17°36.813'S, 149°27.842'W / E.M. Claridge // HOLOTYPE / Mecyclothorax / tuea / J.K. Liebherr 2013 (black-bordered red label).

##### Etymology.

The Tahitian word tuea – square, level, fit together – well characterizes the tight fit of the prothorax to the elytra observed in this species.

##### Distribution and habitat.

The single specimen was collected in a pyrethrin fog sample of moss-covered vegetation at 2070 m elevation on Pito Hiti.

### 10. *Mecyclothorax globosus* species group

**Diagnosis.** Beetles classified in this group are characterized by a narrow pronotum with narrow lateral marginal depressions, the lateral pronotal margins distinctly sinuate before the glabrous hind angles. The 33 included Tahitian species vary extensively in setation, eye development, elytral striation, and coloration. In aggregate, they represent the smallest bodied Tahitian *Mecyclothorax*, with standardized body lengths 3.5–5.0 mm. Two Moorean species are members of this group (Liebherr 2012a).

**Figure 38.**
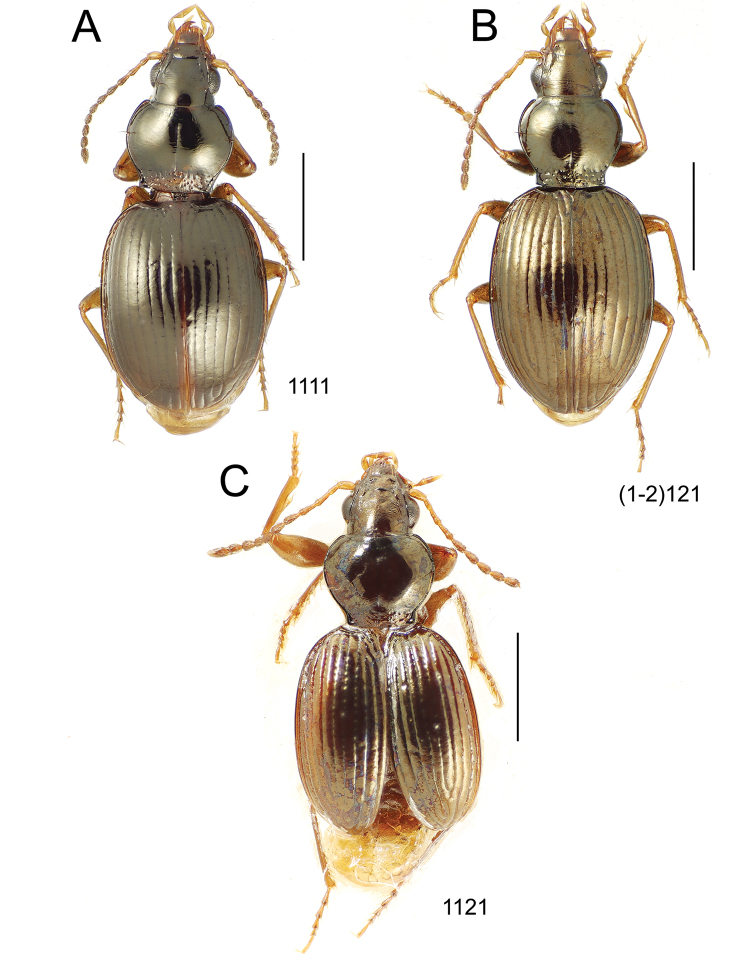
*Mecyclothorax* spp., dorsal view; scale bars 1.0 mm; setal formula (see Fig. 10) at lower right of each figure **A** *Mecyclothorax cupripennis* **B** *Mecyclothorax profondestriatus* holotype male **C** *Mecyclothorax vaifaufa* holotype male.

**Figure 39.**
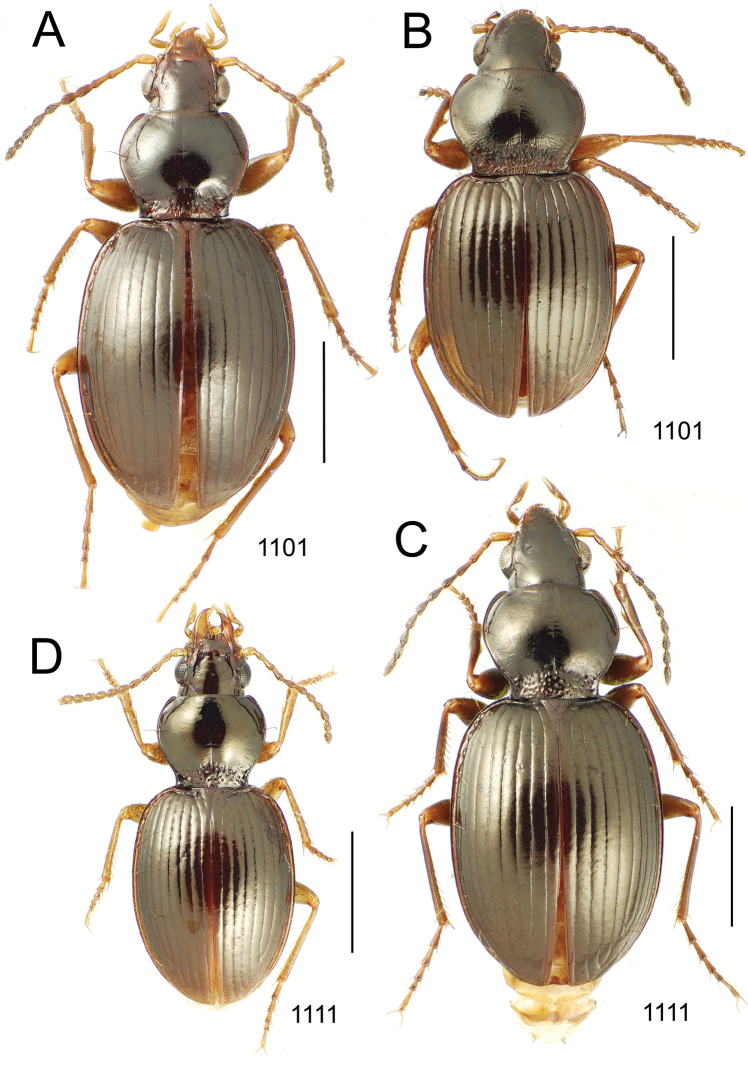
*Mecyclothorax* spp., dorsal view; scale bars 1.0 mm; setal formula (see Fig. 10) at lower right of each figure **A** *Mecyclothorax arboricola* holotype male **B** *Mecyclothorax sabulicola* lectotype male (BPBM) **C** *Mecyclothorax taiarapu* **D** *Mecyclothorax ataraensis* holotype female.

**Figure 40.**
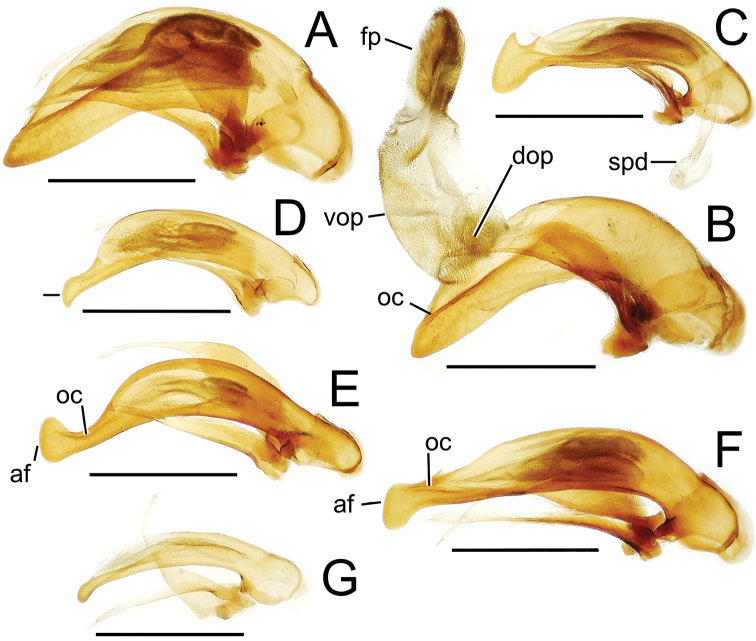
Male aedeagal median lobe and associated parameres, *Mecyclothorax* spp., right lateral view; scale bars 0.5 mm **A** *Mecyclothorax taatitore* paratype (CUIC) **B** *Mecyclothorax taatitore* paratype (CUIC), internal sac everted **C** *Mecyclothorax cupripennis* **D** *Mecyclothorax profondestriatus* holotype **E** *Mecyclothorax vaifaufa* holotype **F** *Mecyclothorax arboricola* paratype (CUIC) **G** *Mecyclothorax sabulicola* lectotype (BPBM) Abbreviations: **af** apical face **dop** dorsal ostial microtrichial patch **fp** flagellar plate **oc** ostial canal **spd** sperm duct **vop** ventral ostial microtrichial patch.

**Figure 41.**
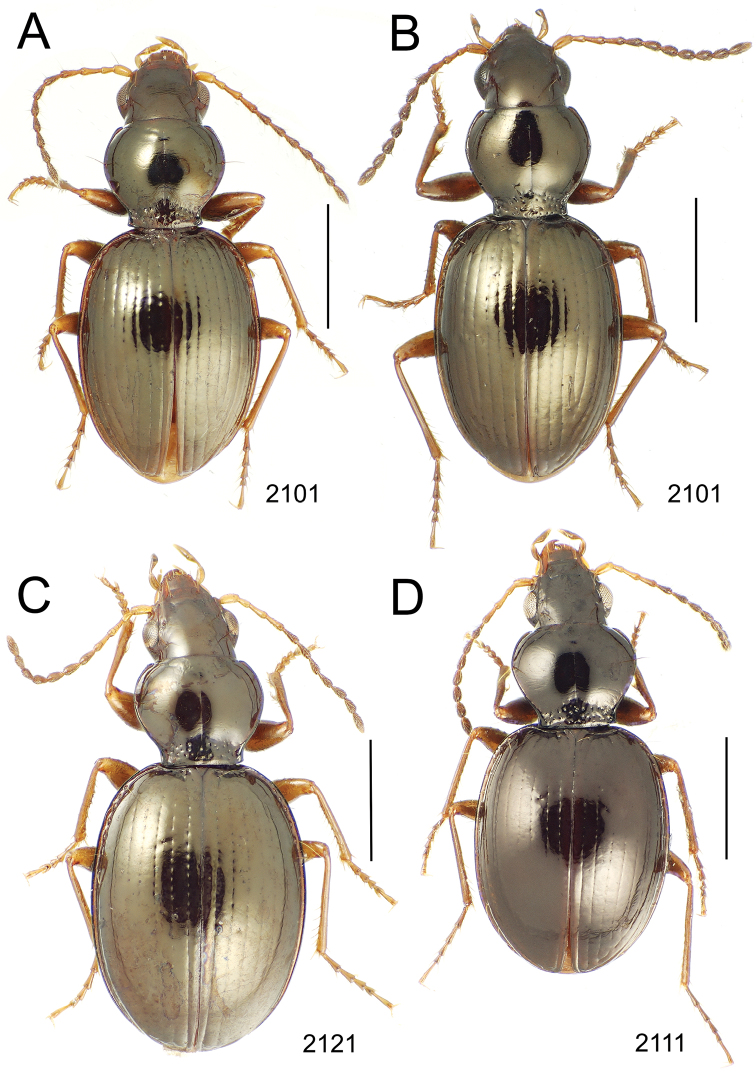
*Mecyclothorax* spp., dorsal view; scale bars 1.0 mm; setal formula (see Fig. 10) at lower right of each figure **A** *Mecyclothorax rahimata* holotype male **B** *Mecyclothorax popotioaoa* holotype male, Mont Tohiea, Moorea **C** *Mecyclothorax anaana* paratype male (CUIC) **D** *Mecyclothorax hemisphaericus*

**Figure 42.**
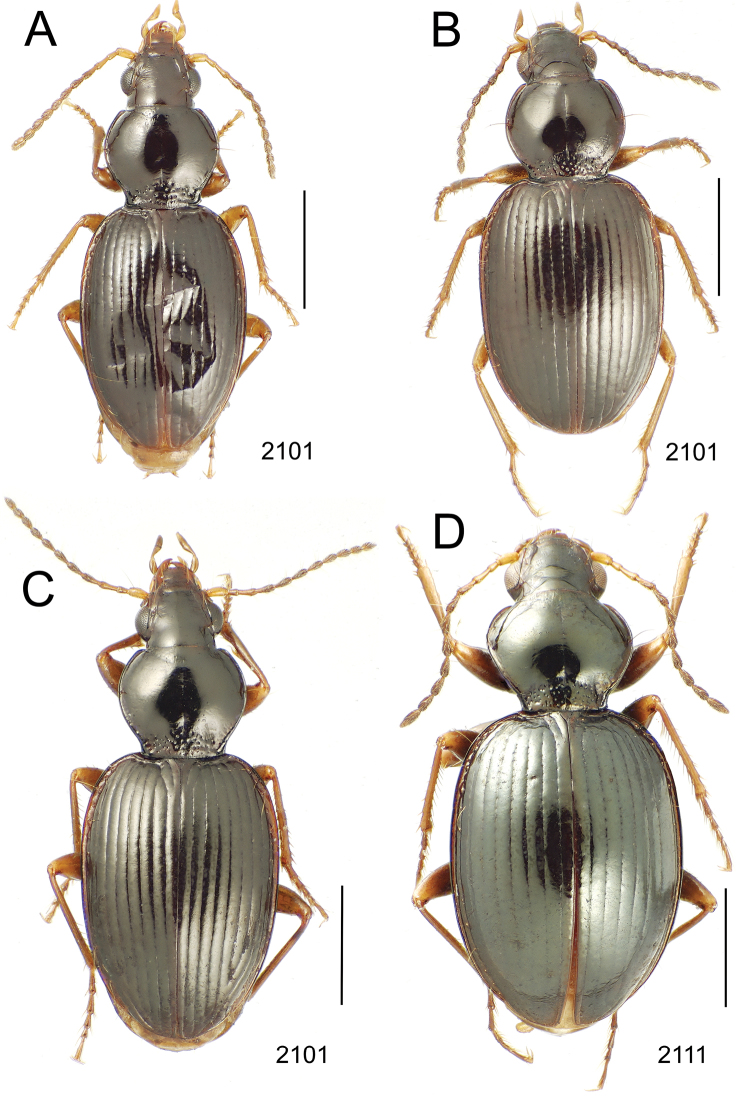
*Mecyclothorax* spp., dorsal view; scale bars 1.0 mm; setal formula (see Fig. 10) at lower right of each figure **A** *Mecyclothorax oaoa* holotype female **B** *Mecyclothorax maninapopoti* holotype female **C** *Mecyclothorax hunapopoti* holotype female **D** *Mecyclothorax fefemata* holotype male.

**Figure 43.**
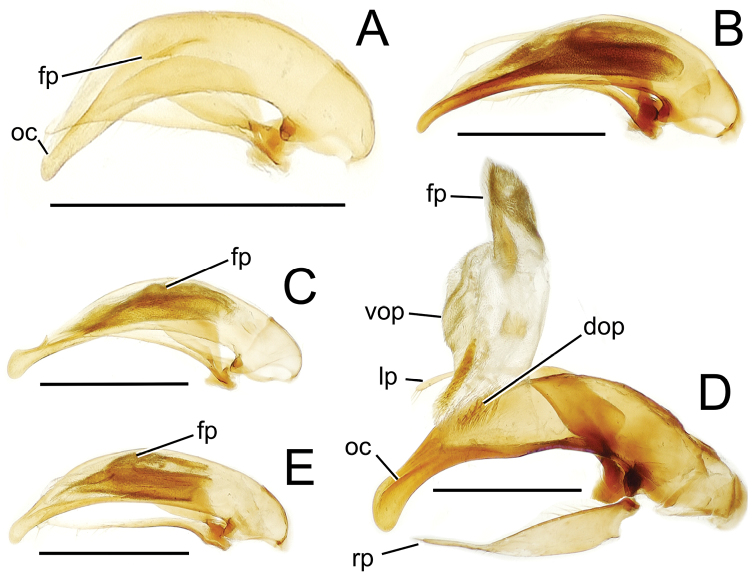
Male aedeagal median lobe and associated parameres, *Mecyclothorax* spp., right lateral view; scale bars 0.5 mm **B** *Mecyclothorax rahimata* holotype **B** *Mecyclothorax anaana* paratype (CUIC) **C** *Mecyclothorax hemisphaericus* **D** *Mecyclothorax fefemata* paratype (CUIC), internal sac everted **E** *Mecyclothorax globosoides* Abbreviations: **dop** dorsal ostial microtrichial patch **fp** flagellar plate **lp** left paramere **oc** ostial canal **rp** right paramere **vop** ventral ostial microtrichial patch.

**Figure 44.**
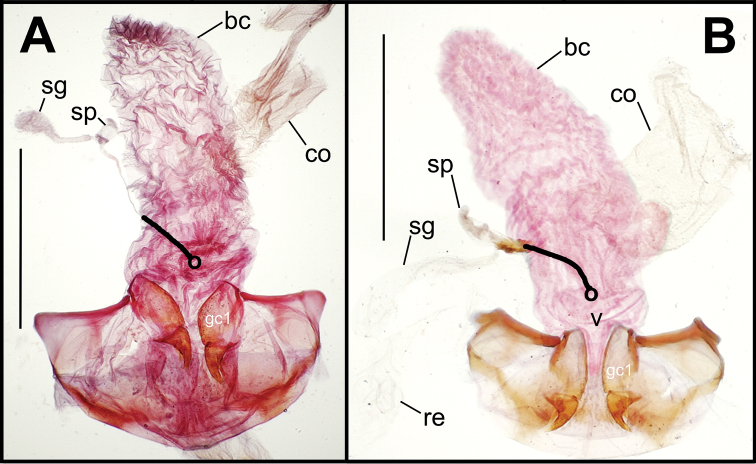
Female reproductive tract dissections, *Mecyclothorax* spp., ventral view; scale bars 0.5 mm **A** *Mecyclothorax fefemata* paratype (CUIC) **B** *Mecyclothorax maninamata* holotype Abbreviations: **bc** bursa copulatrix **co** common oviduct **gc1** basal gonocoxite 1 **re** rectal pads **sg** spermathecal gland **sp** spermatheca **v** vagina. Position of spermathecal duct and juncture of duct with dorsal wall of bursa indicated by black line and terminal circle, respectively.

**Figure 45.**
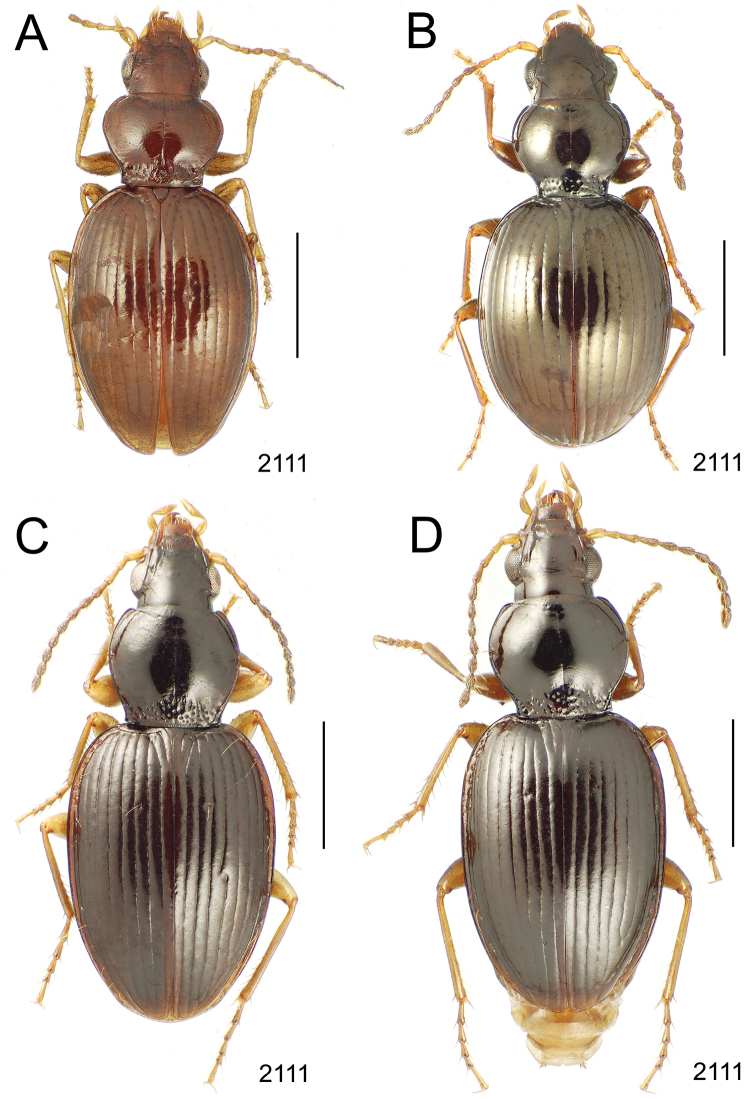
*Mecyclothorax* spp., dorsal view; scale bars 1.0 mm; setal formula (see Fig. 10) at lower right of each figure **A** *Mecyclothorax maninamata* holotype female **B** *Mecyclothorax globosoides* **C** *Mecyclothorax niho* holotype female **D** *Mecyclothorax toretore* holotype female.

**Figure 46.**
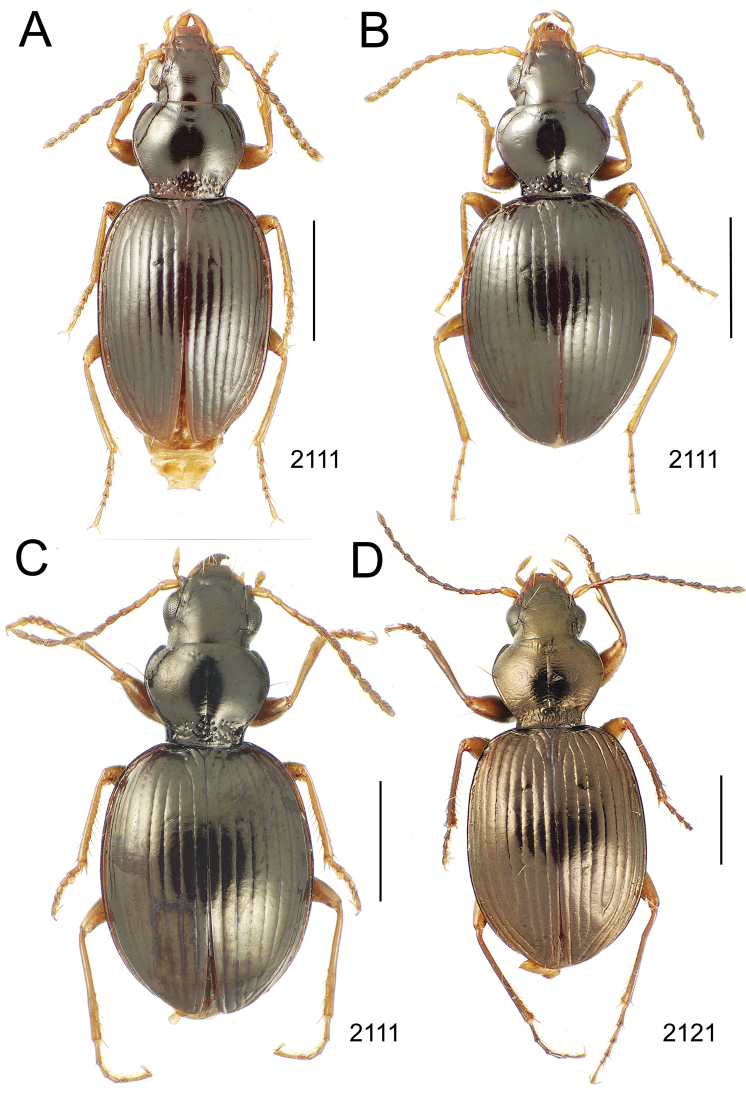
*Mecyclothorax* spp., dorsal view; scale bars 1.0 mm; setal formula (see Fig. 10) at lower right of each figure **A** *Mecyclothorax fuscus* paratype female (MNHN) **B** *Mecyclothorax globosus*, Marau (setal formula 1111 very rare) **C** *Mecyclothorax paraglobosus* **D** *Mecyclothorax cupreus* paratype female (MNHN).

**Figure 47.**
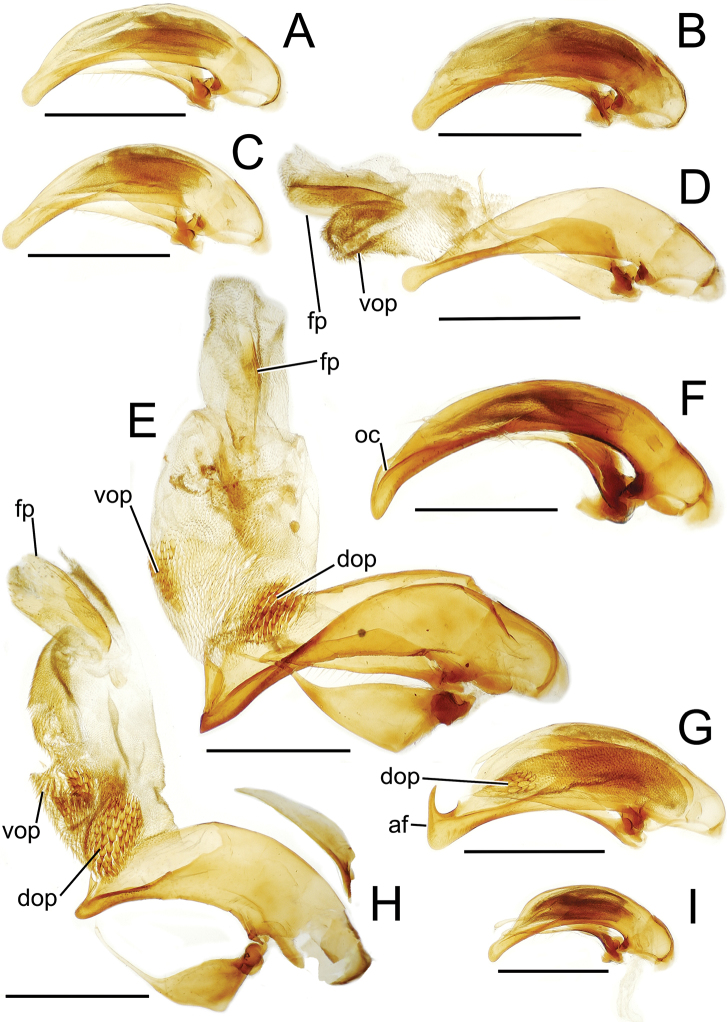
Male aedeagal median lobe and associated parameres, *Mecyclothorax* spp., right lateral view; scale bars 0.5 mm **A** *Mecyclothorax globosus*, Marau **B** *Mecyclothorax globosus*, Marau **C** *Mecyclothorax globosus*, Aorai **D** *Mecyclothorax paraglobosus*, internal sac everted **E** *Mecyclothorax cupreus*, internal sac everted **F** *Mecyclothorax pirihao* paratype (CUIC) **G** *Mecyclothorax spinosus* paratype (MHNB) **H** *Mecyclothorax cupreoides* paratype (MHNB), interal sac everted **I** *Mecyclothorax laevilateralis* Abbreviations: **af** apical face **dop** dorsal ostial microtrichial patch **fp** flagellar plate **oc** ostial canal **vop** ventral ostial microtrichial patch.

**Figure 48.**
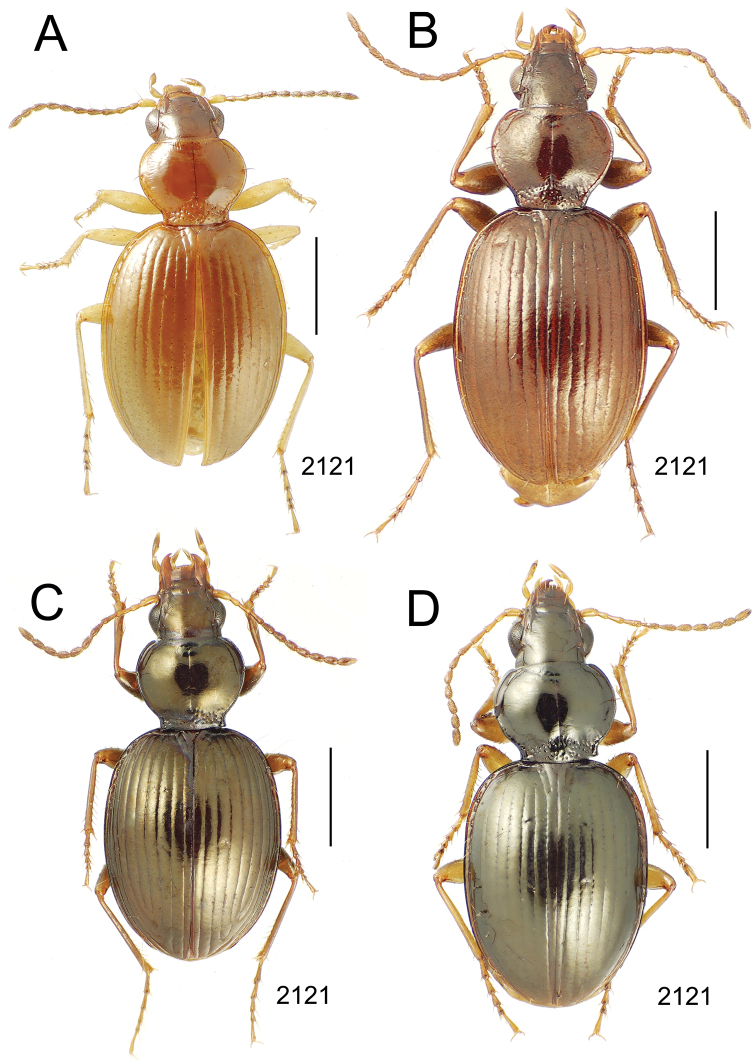
*Mecyclothorax* spp., dorsal view; scale bars 1.0 mm; setal formula (see Fig. 10) at lower right of each figure **A** *Mecyclothorax externestriatus* teneral female **B** *Mecyclothorax pirihao* paratype male (CUIC) **C** *Mecyclothorax spinosus* paratype female (MNHN) **D** *Mecyclothorax poro* holotype female.

**Figure 49.**
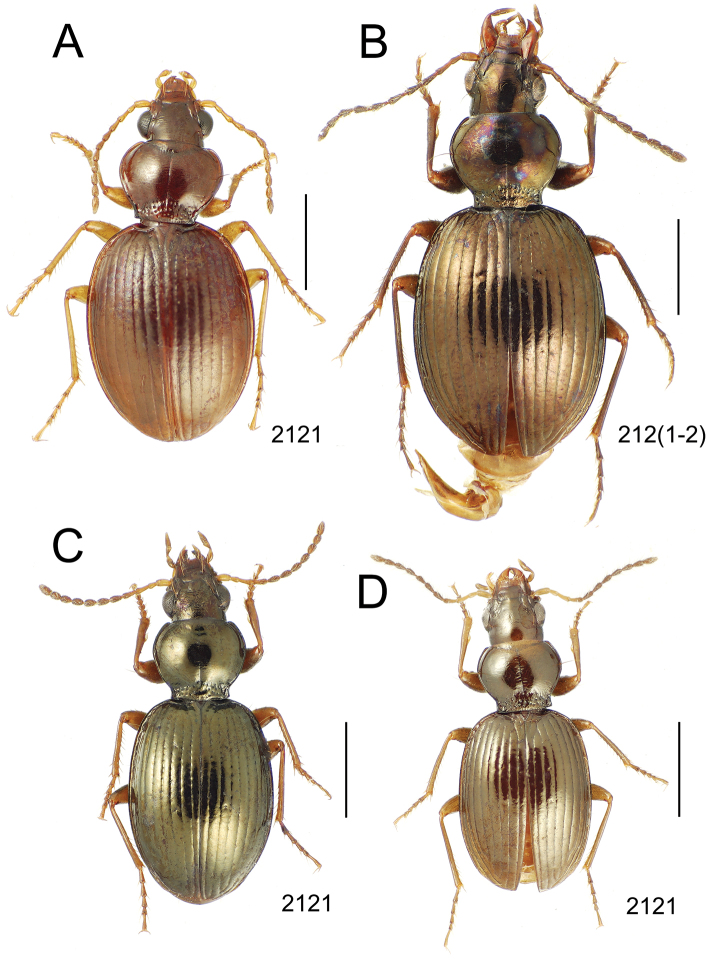
*Mecyclothorax* spp., dorsal view; scale bars 1.0 mm; setal formula (see Fig. 10) at lower right of each figure **A** *Mecyclothorax angulosus* teneral female **B** *Mecyclothorax cupreoides* paratype male (MHNB) **C** *Mecyclothorax laevilateralis* **D** *Mecyclothorax globulosus* allotype female

#### Identification Key to Tahitian Species of the *Mecyclothorax globosus* species group

**Table:** 

1	Pronotal lateral marginal depression evident, of equal breadth throughout length and not narrower at lateral seta, edge upturned; sutural stria 1 and stria 2 basally fused for 0.1× elytral length (Figs 37B, C)	2
–	Pronotal lateral marginal depression obsolete, narrowest at position of lateral seta, wider at front angle, margin beaded or very narrowly upturned; sutural stria and stria 2 free at elytral base, though they may approach, or stria 2 may be obsolete	3
2	Dorsal microsculpture evident, pronotal disc with indistinct transverse mesh and transverse lines visible outside areas of reflected light, elytral intervals covered with distinct transverse microsculpture, the sculpticells ranging from twice as broad as long, to very elongate and so defining transverse lines (Marau)	69. *Mecyclothorax taatitore* sp. n.
–	Dorsal microsculpture obsolete, pronotal disc glossy with only indistinct transverse-line microsculpture outside areas of reflected light, elytral intervals glossy, at most indistinct sculpticells occurring in depressed areas within striae (Aorai)	70. *Mecyclothorax georgettae* Perrault
3	One supraorbital seta each side	4
–	Two supraorbital setae each side	12
4	Elytral microsculpture indistinct or absent, integument glossy	5
–	Elytral microsculpture distinct, transverse mesh to transverse lines	7
5	One or two dorsal elytral setae present in the basal half of third interval	6
–	Third elytral interval glabrous on disc, dorsal elytral setae absent (Aorai)	71. *Mecyclothorax konemata* sp. n.
6	One dorsal elytral seta present in the basal half of third interval (Teatara)	72. *Mecyclothorax cupripennis* Perrault
–	Two dorsal elytral setae present in third interval, on near basal ⅓, the second beyond midlength (Teatara)	73. *Mecyclothorax profondestriatus* Perrault
7	Elytral third interval with one dorsal seta before midlength or glabrous, no dorsal setae present (in rare instances of the common *Mecyclothorax globosus*, if two setae are present on one elytron, then only one seta on the other elytron)	8
–	Elytral third intervals with two dorsal elytral seta bilaterally present	74. M. vaifaufa Perrault
8	Dorsal elytral seta absent, third elytral interval glabrous	9
–	One dorsal elytral seta present in third interval before midlength	10
9	Pronotal median base minutely punctate across width, surface glossy among the punctures, laterobasal depressions deep, margined laterally and basally by marginal bead, no median tubercle present (Fig. 39A); head glossy, transverse wrinkles on frons with some transverse sculpticells between them, neck with shallow isodiametric sculpticells	75. *Mecyclothorax arboricola* sp. n.
–	Pronotal median base irregularly, longitudinally strigose along juncture with disc, surface with granulate isodiametric microsculpture among the large punctures and wrinkles, laterobasal depressions shallow with median tubercle (Fig. 39B); head with well-developed microsculpture, a mixture of isodiametric and transverse sculpticells in transverse rows	76. *Mecyclothorax sabulicola* Britton
10	Elytral microsculpture consisting of elongate, more or less regularly aligned transverse mesh, elytral surface glossy but not iridescent; legs flavous, antennae rufoflavous to brunneous apically	11
–	Elytral microsculpture consisting of fine transverse lines with little tendency to form a mesh, elytral surface subiridescent; legs rufoflavous, antennae darker apically, antennomeres 4–11 dark brunneous to piceous (Teatara)	77. *Mecyclothorax taiarapu* Perrault
11	Elytral striae shallow, deepest portions with elongate punctures that do not expand discal strial breadth, the punctures producing an undulated surface along the depth of the stria, elytral intervals broadly, slightly convex (Fig. 46B); larger bodied beetles, standardized body length 3.5–4.1 mm (Marau, Aorai)	91. *Mecyclothorax globosus* Britton
–	Elytral striae well developed, with rounded punctures that expand discal striae in basal half of elytral length, elytral intervals moderately convex (Fig. 39D); small-bodied beetles, standardized body length 3.3 mm (Teatara)	78. *Mecyclothorax ataraensis* Perrault
12	Discal elytral striae, especially striae 3–5, very shallow, interrupted or scarcely indicated between some of the strial punctures	13
–	Discal elytral discal striae continuous, shallow to deeply incised	15
13	Two or one dorsal elytral setae present, setal formula 2121 or 2111	14
–	Third elytral interval glabous, dorsal elytral seta absent, setal formula 2101 (Fig. 41A) (Marau)	79. *Mecyclothorax rahimata* sp. n.
14	Third elytral interval with two dorsal elytral setae, one near basal ¼ and the second just posterad elytral midlength (Fig. 41C) (Mauru)	80. *Mecyclothorax anaana* Liebherr
–	Third elytral interval with one dorsal elytral seta at ~ 0.30× elytral length (Fig. 41D) (Marau)	81. *Mecyclothorax hemisphaericus* Perrault
15	Third elytral interval glabrous on disc, dorsal elytral setae absent	16
–	One or two dorsal elytral setae present in third interval	18
16	Elytra ovoid, body robust, MEW/MHW = 2.25–2.26 (Fig. 42B, C); discal elytral striae smooth or minutely punctate basally, if punctate the punctures do not expand breadth of striae; frons glossy, with indistinct transverse sculpticells visible over part of surface	17
–	Elytra narrowly ovoid, body gracile, MEW/MHW = 2.03 (Fig. 42A); discal elytral striae distinctly punctate basally, the elongate punctures expanding breadth of striae; frons with evident transverse mesh microsculpture (Teatara)	82. *Mecyclothorax oaoa* sp. n.
17	Discal elytral striae irregular, wavering, elongate punctulae in depths of striae, striae 2–5 shallow to absent basally, not deeply joined to basal groove, sutural stria 1 and stria 2 free basally (Fig. 42B); pronotal basal margins very briefly sinuate anterad hind angles; parascutellar striole shallow, irregularly impressed along length (Marau)	83. *Mecyclothorax maninapopoti* sp. n.
–	Discal elytral striae smooth, deep, striae 1–5 deep and continuous basally, joined to basal groove, sutural stria 1 and stria 2 fused basally (Fig. 42C); pronotal basal margins with elongate sinuation anterad hind angles; parascutellar striole deep and smooth throughout length (Marau)	84. *Mecyclothorax hunapopoti* sp. n.
18	Elytral microsculpture indistinct, discal intervals entirely glossy or indistinct sculpticells visible only in small patches	19
–	Elytra with distinct microsculpture, either a transverse mesh or transverse lines that cross entire breadth of elytral intervals	21
19	Eyes little convex, posterior margin of ocular lobe meeting gena at > 135° angle, ocular ratio 1.31–1.41 (Figs 45A, B); body size smaller, standardized body length 3.5–4.0 mm	20
–	Eyes convex, posterior margin of ocular lobe distinctly projected, meeting gena at < 135° angle, ocular ratio 1.47–1.50 (Fig. 42D); body size larger, standardized body length 4.7–4.9 mm (Marau)	85. *Mecyclothorax fefemata* sp. n.
20	Eyes very flat, frons relatively broad, ocular ratio = 1.31; pronotum densely, longitudinally strigose anterad transverse impression, median base distinctly depressed relative to disc, the surface shagreened by isodiametric sculpticells among deep, distinct punctures (Marau)	86. *Mecyclothorax maninamata* sp. n.
–	Eyes slightly projected, ocular ratio = 1.41; pronotal anteromedial surface glossy, anterior transverse impression obsolete, median base sparsely lined with small punctures, the surface among punctures glossy (Teatara)	87. *Mecyclothorax globosoides* Perrault
21	One dorsal elytral seta present in the basal half of third elytral interval (or in rare instances of the abundant *Mecyclothorax globosus* Britton of Marau and Aorai, unilaterally two dorsal setae)	22
–	Two dorsal elytral setae bilaterally present in third interval	26
22	Elytra with distinct microsculpture consisting of dense transverse lines without formation of a mesh	23
–	Elytra with evident transverse microsculpture arranged in a mesh that is more or less transverse	25
23	Pronotal lateral margins anterad hind angles convergent to the level of the anterolateral portion of the median base, then divergent, hind angles projected (Figs 45D, 46A); elytral striae 2–6 shallower near basal groove than on disc	24
–	Pronotal lateral margins convergent for only a very short distance anterad hind angles, divergent for much of distance laterad median base, hind angles denticulate (Fig. 45C); elytral striae 2–6 fully as deep near basal groove as on disc (Teatara)	88. *Mecyclothorax niho* sp. n.
24	Elytra subquadrate, broad basally, MEW/HuW = 1.87; elytral striae 5 and 6 obsolete basally, the striae not joined to elytral basal groove at humeral angle (Fig. 45D); antennae elongate, antennomere 8 filiform, length >2.0× maximum breadth (Mauru)	89. *Mecyclothorax toretore* Liebherr
–	Elytra subovate, humeri rounded, MEW/HuW = 2.0; elytral striae 5 and 6 continuous basally though shallow, joined to elytral basal groove at humeral angle (Fig. 46A); antennae shorter, antennomere 8 submoniliform, length 1.6× maximum breadth (Aorai)	90. *Mecyclothorax fuscus* Perrault
25	Male aedeagal median lobe short and broad (Figs 47A–C) (Aorai, Marau)	91. *Mecyclothorax globosus* Britton
–	Male aedeagal median lobe long and narrow (Fig. 47D) (Pito Hiti)	92. *Mecyclothorax paraglobosus* Perrault
26	Vertex of head and pronotal disc glossy, lacking distinct, raised, mesh-like microsculpture (Figs 48, 49)	27
–	Vertex of head and pronotal disc covered with raised isodiametric to transverse sculpticells, the surface with cupreous reflection (Fig. 46D) (Aorai)	93. *Mecyclothorax cupreus* Perrault
27	Elytral disc with well-developed transverse microsculpture	28
–	Elytral disc glossy, microsculpture indistinct	32
28	Discal elytral striae deep, smooth to finely punctate, the punctures not expanding strial breadth; elytra dark with aeneous metallic reflection or pallid, brunneous	29
–	Discal elytral striae distinctly punctate basally, punctures expanding strial breadth; elytra rufopiceous (Marau)	94. *Mecyclothorax externestriatus* Perrault
29	Pronotal hind angles distinct, right to acute, pronotal lateral margin distinctly sinuate anterad hind angle (Figs 48C, D, 49), pronotal base moderately narrow, MPW/BPW = 1.46–1.63	30
–	Pronotal hind angles obtuse-rounded, little projected, pronotal lateral margin moderately sinuate anterad hind angle (Fig. 48B), pronotal base very narrow, MPW/BPW = 1.83–1.90 (Mauru)	95. *Mecyclothorax pirihao* Liebherr
30	Pronotum more transverse, MPW/PL = 1.25–1.26 (Figs 48D, 49); pronotal median base with deep, distinct punctures, punctures along margin with disc more elongate, deeper than punctures closer to basal margin	31
–	Pronotum less transverse, MPW/PL = 1.16–1.20 (Figs 48C); pronotal median base indistinctly and sparsely punctate, all punctures circular, punctures along margin with disc smaller than those closer to basal margin (Teatara)	96. *Mecyclothorax spinosus* Perrault
31	Elytral striae 3–5 obsolete on elytral base, elytral humeral region smooth (Fig. 48D); elytra subquadrate, base broader, elytral margins extended laterally outside humeral angles (Mauru)	97. *Mecyclothorax poro* Liebherr
–	Elytral striae 3–5 continuous and traceable to juncture with elytral basal groove (Fig. 49A); elytra ovoid, narrow basally, lateral margins rounded off posteriorly laterad humeral angles (Aorai)	98. *Mecyclothorax angulosus* Perrault
32	Elytra narrowly ovoid, not so broad relative to pronotum, MEW/MPW = 1.52–1.62 (Figs 49C, D); standardized body length 3.4–4.1 mm	33
–	Elytra broad, orbicular, pronotum narrow, MEW/MPW = 1.65–1.76 (Fig. 49B); standardized body length 4.0–4.9 mm (Teatara)	99. *Mecyclothorax cupreoides* Perrault
33	Pronotal base broader, MPW/BPW = 1.50–1.53; lateral elytral striae 6–7 as deep or deeper than discal striae 1–5	34
–	Pronotal base narrower, MPW/BPW = 1.56–1.58; discal elytral striae moderately deep, lateral stria 6–7 much shallower, 7^th^ indistinct (Teatara)	100. *Mecyclothorax laevilateralis* Perrault
34	Dorsal body surface glossy, rufobrunneous, but without bronzed metallic reflection; elytra symmetrically ellipsoid, lateral margins nearly parallel at midlength (Fig. 49D); elytral striae 3–5 shallower near elytral basal groove than on disc, obsolete to broad and shallow basally (Aorai)	101. *Mecyclothorax globulosus* Perrault
–	Dorsal body surface glossy, rufobrunneous, but with bluish metallic reflection on pronotal disc; elytra slightly obovate, lateral margins more narrowly rounded near humeri, margins convex at midlength (Fig. 38B); elytral striae 3–5 complete, finely incised basally, deeply joined to elytral basal groove (Teatara)	72. *Mecyclothorax profondestriatus* Perrault

#### 69. Mecyclothorax taatitore sp. n.

http://zoobank.org/14A83A4D-19F3-48D9-88A8-46F2D05D36BA

http://species-id.net/wiki/Mecyclothorax_taatitore

##### Diagnosis.

This species and *Mecyclothorax georgettae* are isolated within the *Mecyclothorax globosus* group by their moderately broad pronotal lateral margins, but the bisetose, cordate pronotum and small body size are consistent with group membership. Both species exhibit a unique synapomorphy within the Tahitian *Mecyclothorax* fauna; the elongate basal fusion of sutural stria 1 and stria 2 (Figs 37B, C). Beside the well-developed elytral microsculpture used in the key, this species may be diagnosed from *Mecyclothorax georgettae* by the obovate elytra, the elytral base narrow and humeri proximate, MEW/HuW = 2.23–2.57 (n = 5). Setal formula 2121; standardized body length 4.1–5.0 mm. *Head* with triangularly depressed frontal grooves, the apex a fine low carina just mesad the anterior supraorbital seta, the grooves expanded medially across frons to nearly meet at frontoclypeal suture, the frons transversely wrinkled behind, obliquely wrinkled anteriorly; eyes moderately convex, dorsal margins convergent at supraorbital setae, ocular ratio 1.53–1.57, ocular lobe gently protruded from gena, ocular lobe ratio 0.78–0.82; antennae moderately elongate, submoniliform, antennomere 8 length 1.80× maximal breadth. *Pronotum* little transverse, MPW/PL = 1.14–1.25 (n = 5), narrow basally, lateral margins convergent anterad right to slightly obtuse hind angles, MPW/BPW = 1.44–1.54; median base distinctly depressed relative to convex disc, with ~24 punctures each side, the punctures anastomosing laterally, and punctures along margin with disc elongate; anterior transverse impression very shallow medially, obscured by 10–12 fine longitudinal wrinkles that extend across slightly convex anterior callosity; front angles protruded, tightly rounded, lateral marginal depression narrowest just before lateral pronotal seta, the edge upturned, broader inside front angles and posteriorly; laterobasal depression a broadened extension of lateral depression, the surface rugose due to transverse wrinkles that span the laterobasal depression and basal portion of lateral depression. *Elytra* broadly convex medially, the sides laterad interval 6 more sloped to near vertical juncture with lateral marginal depression; elytral basal groove distinctly curved to the angulate humerus, striae 1 + 2, 3–5 deep and continuous to basal groove mesad humerus; discal striae 1–7 deep, indistinctly punctate before midlength; sutural interval 1 and scutellum elevated relative to intervals 3 and 4 basally; all striae complete to apex though broader and shallower than on disc, striae 1 and 7 the narrowest of them apically, interval 8 the same convexity as inner intervals except apically where intervals narrows to its terminus; lateral elytral setae 7 + 6. *Microsculpture* of frons and vertex a well-developed transverse mesh visible amongst the transverse wrinkles, sculpticell breadth 2–4× length, more elongate in wrinkles, neck with isodiametric sculpticells near pronotum; pronotal disc with indistinct elongate transverse mesh, microsculpture also covering median base among punctures, the sculpticells there a mix of isodiametric and transverse. *Coloration* of head rufobrunneous with piceous cast in frontal grooves; antennomere 1 flavous, 2–3 rufoflavous, and 4–11 brunneous; pronotal disc rufobrunneous to rufopiceous, lateral pronotal margins narrowly rufoflavous; elytra darkest on discal intervals 2–4, progressively paler laterally and apically, the coloration varying from a piceous cloud on disc and flavous lateral marginal depression (as in Fig. 37C), to more uniformly brunneous with the disc only slightly darker (Fig. 37B), sutural interval paler than piceous cloud when latter present; femora and elytral epipleuron flavous, tibiae rufoflavous with rufous cast.

Male genitalia. In keeping with the isolated external anatomy of this species within Tahitian *Mecyclothorax* spp., the aedeagal median lobe is very broad basally and terminated apically by a short, distinctly pointed apex with variably curved ventral margin (Figs 3F, 40A); ostial canal not apparent, extended only a short distance beyond ostium along dorsal margin of lobe (Figs 3F, 40B); internal sac broad, with diffuse ventral ostial microtrichial patch and denser more spiculate dorsal ostial microtrichial patch; flagellar plate moderately elongate, length 0.42–0.46× distance from parameral articulations to tip (Figs 3F, 40B).

Female reproductive tract. Bursa copulatrix broad, slightly expanded apically, length 1.6× maximal breadth compressed under microslide cover slip (Fig. 33D); basal gonocoxite 1 with apical fringe of 3–4 setae laterally, these setae basad lateral ensiform setae of apical gonocoxite 2, and one seta isolated medially near basal condyle of gonocoxite 2 (Fig. 9E); medial surface of gonocoxite 1 lined with setae, a somewhat larger seta at the medioapical angle, and 8–11 smaller setae arrayed basally; apical gonocoxite 2 stout, moderately expanded basolaterally, apex rounded, with two short lateral ensiform setae, one dorsal ensiform seta, and an apical sensory furrow with two apical nematiform setae and two furrow pegs.

Holotype male (MNHN) labeled: French Polynesia: Tahiti Nui / Mt. Marau road el. 1165 m / 4-IX-2006 lot 03 / 17°36.440'S, 149°32.877'W / pyrethrin fog rheocrene w/ / moss/liverwort J.K. Liebherr // HOLOTYPE / Mecyclothorax / taatitore / J.K. Liebherr 2013 (black-bordered red label).

Allotype female (MNHN) labeled as the holotype.

Paratypes: labeled as the holotype (CUIC, 6; EMEC, 1; NMNH, 1).

##### Etymology.

The species epithet compounds the Tahitian words tā’ati, meaning unite or join together, and tore, which means, among other things, streaked, or military stripes. The epithet signifies the fused bases of elytral striae 1 + 2.

##### Distribution and habitat.

These beetles were discovered by pyrethrin fogging a wet rock face above a small stream. The rock face lay on the sidewall of a small gulch that was shaded by trees, and was about 6 m in height and about 10 m across, the fractured face lying at a 75° angle to the horizontal. The surface was covered with mosses and liverworts, and the beetles crawled out of the cracks due to the action of the pyrethrin. One adult and four larvae of *Colpodes eremita* (Fairmaire) were also found escaping cracks in the rock after the pyrethrin application.

#### 70. Mecyclothorax georgettae

Perrault, 1978b: 149; 1989: 60

http://species-id.net/wiki/Mecyclothorax_georgettae

##### Identification.

Sharing the basally fused elytral striae 1 + 2 and narrow pronotum with *Mecyclothorax taatitore*, this species can be told by the glossy discal elytral intervals, their surface without any microsculpture, the only microsculpture on the elytra confined to the deepest portions of the narrowly incised striae, where indistinct isodiametric sculpticells occur near the small, elongate strial punctures. The frons, clypeus, and vertex are rufopiceous, with the pronotal disc slightly paler, a dark rufobrunneous. The elytra are contrastedly paler, with a ground color of dark rufoflavous, and a piceous cloud on each elytron that spans elytral intervals 2–4 and centers on the posterior dorsal elytral seta. The elytra are more ovate than in *Mecyclothorax taatitore*, with broadly rounded lateral margins outside the angulate humeri (Fig. 37C). The frons is covered with mixed isodiametric and transverse-mesh microsculpture that occurs on the flat surfaces between the transverse wrinkles, and the pronotal disc is covered with an indistinct transverse mesh, sculpticell breadth 2–4× length, the microsculpture visible only outside areas of reflected light. The male aedeagal median lobe is similar to that of *Mecyclothorax taatitore* in its great breadth and short apex, but in *Mecyclothorax georgettae* males, the median lobe apex is truncate with an acuminate dorsal tooth (Perrault 1989; fig. 5). Setal formula 2121; standardized body length 5.0 mm.

##### Distribution and habitat.

This species’ known distribution is Mont Aorai from 1000–1750 m elevation. Two of the three known specimens were collected in a vinegar pitfall trap, demonstrating that this species shares geophilic habits with its sister, *Mecyclothorax taatitore*.

#### 71. Mecyclothorax konemata sp. n.

http://zoobank.org/2FC2C9B4-AECD-4141-BF24-0A523B9965DD

http://species-id.net/wiki/Mecyclothorax_konemata

##### Diagnosis.

This is the smallest-bodied species in the *Mecyclothorax globosus* group characterized by presence of a single, posterior supraorbital seta in company with a glabrous third elytral interval (Figs 37D, 39A, B); seta formula 1101; standardized body length 3.4 mm. The eyes are very small and little convex (Fig. 37D); ocular ratio 1.32, ocular lobe ratio 0.72. A diameter line with one end set at the antennal articulation crosses 8 ommatidia on the compound eye. *Head* with straight, narrowly depressed, and slightly convergent frontal grooves, the grooves’ posterior terminus just anteromesad the posterior supraorbital seta; antennae short, moniliform, antennomere 8 length 1.43× maximal breadth. *Pronotum* little transverse, lateral margin straight to slightly convergent anterad right to slightly obtuse hind angle, MPW/PL = 1.18, MPW/BPW = 1.47; anterior margin of median base depressed relative to convex disc, but base inflated nearly to basal margin medially and so appearing convex; juncture between median base and disc lined with 5–6 longitudinal punctures, ~6 indistinct punctures each side toward basal margin; anterior transverse impression well defined, a carinate anterior margin present across breadth, impression medially crossed by about 6 longitudinal wrinkles each side, the wrinkles continuing across anterior callosity; front angles only slightly protruded, the lateral marginal depression very narrow throughout length, the edge beaded; laterobasal depression with irregular surface, broad shallow depressions near pronotal basal margin and transverse wrinkles anteriorly near lateral depression. *Elytra* ovate, broad basally and attenuate apically, the basal groove gently curved mesad the obtuse-rounded humerus, MEW/HuW = 2.05; elytral disc convex, scutellum and base depressed, and sides sloped to vertical juncture with lateral marginal depression; striae 1–6 shallow, distinctly punctate on disc with punctures expanding striae, the associated intervals slightly convex medially, flatter in lateral intervals; stria 7 very shallow, interrupted, with irregular elongate depressions along length, slightly deeper and broader near elytral apex; interval 8 convex from posterior end of lateral elytral setae to apex, but not upraised above stria 7; lateral elytral setae 6 + 5, the posterior seta of the anterior series, and the anterior seta of the posterior series isolated from the other setae in the respective series. *Microsculpture* of frons an evident transverse mesh, sculpticell breadth 2–3× length, sculpticells isodiametric on neck; pronotal disc with obsolete transverse mesh, mesh difficult to discern in reflected light, sculpticell breadth 2–4× medially; elytral discal intervals covered with shallow transverse mesh, sculpticell breadth 2–3× length. *Coloration* of head rufous; antennomere 1 flavous, 2–3 with smoky cast, 4–11 rufobrunneous; pronotal disc dark rufous, margins concolorous; elytra rufobrunneous, the sutural interval rufous basally and rufoflavous apically, the lateral marginal depression paler, rufoflavous only near pale, rufoflavous elytral apex; femora flavous, tibiae flavous with rufoflavous cast.

Holotype female (MNHN) labeled: Aorai - Tahiti / piege 1100/1400 m / 20/21.V.1978 (pink paper label // Museum Paris / 1994 / Coll. G. Perrault (pink paper) // ε (hand written on white label) // HOLOTYPE / Mecyclothorax / konemata / J.K. Liebherr 2013 (black-bordered red label).

##### Etymology.

The species epithet compounds the Tahitian word kone, meaning small, with mata, meaning eye; konemata signifying the small eyes characterizing this species.

##### Distribution and habitat.

This specimen was collected by Georges Perrault on Mont Aorai, 1000–1400 m elevation in 1978, and placed a the end of his collection in a small section of undescribed material. The epsilon – i.e., “ε” – identifier label suggests that he understood this specimen to represent something distinctive, but he did not have the opportunity to describe it.

#### 72. Mecyclothorax cupripennis

Perrault, 1989: 69

http://species-id.net/wiki/Mecyclothorax_cupripennis

##### Identification.

Among the eight *Mecyclothorax globosus* group species from Tahiti characterized by a single supraorbital seta (Figs 37D, 38, 39), this species stands out based on: 1, the narrow head with small eyes, ocular ratio 1.42 (Fig. 38A); 2, the presence of a single (anterior) dorsal elytral seta: and 3, greatly reduced elytral microsculpture consisting of a very shallow transverse mesh, sculpticell breadth 2–3× length, that is visible only outside areas of reflected light due to the glossy nature of the elytral cuticle. The breadth of the head can be quantified by comparison with the maximum elytral width; MEW/MHW = 2.32. The frons and vertex are glossy, the former crossed by fine radiating wrinkles emanating from the hind portion of the frontal grooves, and the pronotal disc is glossy with small patches of indistinct transverse mesh outside the areas of reflected light. The male aedeagal median lobe is exceedingly diagnostic, with the lobe shaft narrow, and the apex beyond the ostium expanded ventrally as a rounded expansion, and dorsally as a hooklike projection (Fig. 40C), the hooked configuration looking very much like a can-opener. Setal formula 1111; standardized body length 3.6–3.9 mm.

##### Distribution and habitat.

This species is known from Mont Teatara, Tahiti Iti, from 900–1100 m elevation. One specimen was obtained through the application of pyrethrin fog to a horizontal mossy log

#### 73. Mecyclothorax profondestriatus

Perrault, 1989: 66

http://species-id.net/wiki/Mecyclothorax_profondestriatus

##### Identification.

This species is distinguished by the narrowly ovoid elytra with very deep elytral striae, the lateral striae 7 and 8 actually more deeply incised than the discal striae. All striae are deeply impressed to the elytral basal groove, and striae 1 + 2 are basally fused from the basal groove to a level halfway along the parascutellar striole (Fig. 38B). The third elytral interval bears two dorsal setae. The anterior supraorbital seta is polymorphically present or absent, with two of the four specimens unilaterally exhibiting the anterior supraorbital seta; setal formula (1-2)121. The frons bears an evident isodiametric mesh, the sculpticell margins shallow but traceable outside areas of reflected light. The pronotal disc is covered with a very shallow elongate transverse mesh, those sculpticells traceable outside areas of reflected light having breadth 4× length. The convex discal elytral intervals are glossy. The male aedeagal median lobe is broad for much of its length then precipitously narrowed dorsoventrally apicad the ostium (Fig. 40D). The apex is narrowly downturned at the tip, resulting in an oblique apical face. Standardized body length 3.4–3.7 mm.

##### Distribution and habitat.

This species is known from the type series of three specimens collected on Mont Teatara, 900–1100 m elevation, plus a fourth specimen collected in a 2006 pyrethrin fog sample of a moss-covered *Weinmannia* log, 1080–1100 m elevation.

#### 74. Mecyclothorax vaifaufa

Perrault, 1989: 64

http://species-id.net/wiki/Mecyclothorax_vaifaufa

##### Identification.

This and *Mecyclothorax profondestriatus* (Figs 38B, C) are the only two species of the *Mecyclothorax globosus* group with individuals possessing only the posterior supraorbital seta in combination with two dorsal elytral setae, resulting in a setal formula of 1121. *Mecyclothorax vaifaufa* can be distinguished by the dense, well-developed transverse microsculpture lining the elytral intervals, the microsculpture predominantly consisting of transverse lines surrounding areas of transverse mesh, sculpticell breadth 2–4× length in areas with mesh. All elytral striae are deeply impressed, striae 1–5 with elongate punctures that do not expand striae, and all intervals 1–9 are equally convex. The frons is glossy, the surface covered with elongate transverse sculpticells and larger transverse wrinkles. The pronotal disc is also glossy, with only the vaguest suggestion of transverse microsculpture, the micropunctures distributed across the cuticle more visible than any sculpticells. The male aedeagal median lobe is distinctly curved dorsally, broader at midlength than at basal bulb, and the apex is broadly expanded into an asymmetrical spatulate tip, with tightly rounded dorsal projection and broad apical face (Fig. 40E). The ostial canal is extended parallel to the dorsal margin from the ostium to the base of the dorsal expansion. Standardized body length 3.8 mm.

##### Distribution and habitat.

The unique type specimen was collected at 1000 m elevation at a location labeled Parc Vaifaufa, Tahiti Iti (Perrault 1989). The specimen was found in moss. At 1000 m elevation, Parc Vaifaufa lies on the northwest approach to Mont Teatara, 500 m elevation above the Belvedere de Taravao that overlooks Lac du Vaiufaufa (IGN 1994). Thus we should expect this species to occupy similarly elevated habitats on other flanks of Teatara.

#### 75. Mecyclothorax arboricola sp. n.

http://zoobank.org/819FFDBC-93C7-4B56-B020-3765B729915D

http://species-id.net/wiki/Mecyclothorax_arboricola

##### Diagnosis.

These beetles are the largest in the *Mecyclothorax globosus* group to combine the presence of only the posterior supraorbital seta with the absence of dorsal elytral setae (Figs 37D, 39A, B); setal formula 1101, standardized body length 3.7–4.1 mm. The pronotum is more constricted basally than in the similarly setose *Mecyclothorax sabulicola* (Figs 39A versus B); MPW/BPW = 1.45–1.52 (n = 5). The dorsal microsculpture is also less developed: 1, frons glossy with a shallow isodiametric mesh on the neck; 2, pronotal disc with an indistinct transverse mesh visible outside areas of reflected light; and 3, discal elytral intervals covered with evident transverse lines accompanied by areas of transverse mesh, sculpticell breadth 3–4× breadth. *Head* with frontal grooves narrow behind, radiating wrinkles emanating from groove onto frons, groove broad and shallow near frontoclypeal suture; eyes small, little convex, ocular ratio 1.38–1.43, ocular lobe meeting gena at broad shallow groove, lobe very obtusely protruded from gena, ocular lobe ratio 0.79–0.86; antennae moderately elongate, submoniliform, antennomere 8 length 1.75× maximal breadth. *Pronotum* moderately transverse, MPW/PL = 1.18–1.22, cordate, lateral margins convergent before acute, projected hind angles; median base depressed relative to convex disc, smoother medially with 9–10 punctures laterally each side; anterior transverse impression broad, shallow, smooth, with at most three indistinct longitudinal wrinkles each side near middle, anterior callosity smooth, slightly convex; front angles rounded, slightly protruded; lateral marginal depression narrow, edge beaded, slightly wider inside front angle; laterobasal depression deep, surface rugose to irregularly punctate, margined laterally and basally by raised margin. *Elytra* ovate, MEW/HuW = 2.08–2.20, humeri angulate laterad evenly curved basal groove; disc convexly domed above depressed scutellum, sides sloped to near vertical juncture with lateral marginal depressions, sutural intervals of each elytron raised as a broad callous joined at the elevated suture; elytral striae 1–7 smooth, continuous, minute punctures at depth of stria on disc the only indication of punctation, striae 1–6 continuous and deep to basal groove, striae 1+2 fused for short distance from laterad parascutellar seta to basal groove; interval 8 convex laterally, upraised slightly laterad stria 7 apicad fused terminus of striae 3 + 4; lateral elytral setae 7 + (5–6). *Coloration* of head rufobrunneous, clypeus and labrum rufous; antennomere 1 flavous, 2–3 rufoflavous; 4–11 rufobrunneous; pronotal disc dark rufobrunneous, the anterior callosity and lateral margins narrowly, and median base more broadly, rufous; elytral disc rufobrunneous with silvery metallic reflection, the apex concolorous with disc, upraised sutural interval rufous, lateral marginal depressions narrowly rufoflavous, palest apically; femora and tibiae rufoflavous.

Male genitalia. Aedeagal median lobe gracile, shaft moderately narrow and slightly curved (Fig. 40F); apex asymmetrically expanded with larger ventral expansion and slight dorsal expansion, the apical face oblique; ostial canal short, terminated near dorsal margin.

Female reproductive tract. Bursa copulatrix basally as broad as vagina, apically narrowed and parallel sided when compressed under microslide cover slip (Fig. 7A); bursal surface membranous, capable of extensive expansion based on wrinkles across surface of prepared dissection; basal gonocoxite 1 narrow, elongate, with 3 small apical fringe setae and a fourth smaller seta near medioapical angle, ~6 small setae along mesal margin (Fig. 9F); apical gonocoxite 2 narrow with elongate laterobasal expansion, apex acuminate; two narrow lateral ensiform setae and one dorsal ensiform setae, plus an apical sensory furrow with two apical nematiform setae and two furrow pegs.

Holotype male (MNHN) labeled: French Polynesia: Tahiti Nui / Mt. Marau road el. 1275 m / 10-IX-2006 lot 02 / 17°36.433'S, 149°32.339'W / pyr. fog horiz. *Weinmannia* / trunks + veg. J.K. Liebherr // HOLOTYPE / Mecyclothorax / arboricola / J.K. Liebherr 2013 (black-bordered red label).

Allotype female (MNHN) labeled as holotype.

Paratypes: labeled as the holotype (CUIC, 2); Mt. Marau, 1280 m el., 6-xi-1999, Polhemus, pyr. fog *Weinmannia* forest (CUIC, 1; NMNH, 2); Mt. Marau, 1185 m el., 17°36.440'S, 149°32.877'W, 4-ix-2006 lot 02, Liebherr, pyr. fog tree fern fronds/*Weinmannia* (CUIC, 1).

##### Etymology.

The species epithet arboricola, tree loving, is of parallel derivation to that of the anatomically similar species *Mecyclothorax sabulicola*; i.e., the sand-loving *Mecyclothorax*.

##### Distribution and habitat.

This species is known to occupy *Weinmannia* forest habitats from 1185–1280 m elevation on Mont Marau.

#### 76. Mecyclothorax sabulicola

Britton, 1948: 113

http://species-id.net/wiki/Mecyclothorax_sabulicola

Thriscothorax minutus Britton, 1938: 106 (junior homonym; Britton 1948).

##### Identification.

Of the three Tahitian species in the *Mecyclothorax globosus* group with only the posterior supraorbital seta and no dorsal elytral setae – setal formula 1101 (Figs 37D, 39A, B) – individuals of this species exhibit the most well-developed microsculpture. The head is covered with a distinct isodiametric mesh, the sculpticells elongate when associated with transverse wrinkles on the frons. The pronotal disc has a well-developed transverse mesh over the entire surface, the sculpticells visible in areas of reflected light, and the discal elytral intervals are lined with transverse microsculpture, a mixture of transverse mesh and transverse lines. The pronotal base is broad (Fig. 39B) and the pronotal laterobasal depressions shallow with a median tubercle. The elytra are broad basally, the lateral margins extended laterally beyond obtusely subangulate humeri. The eyes are moderately convex, ocular ratio 1.42, with a horizontal diameter crossing a maximum of 14 ommatidia. The male aedeagal median lobe is gracile, the shaft of the same diameter from basal bulb to ostium (Fig. 40G). The lobe apex is expanded downward, resulting in an asymmetrical apical expansion and an oblique apical face. In this regard the aedeagus looks like a less exaggerated version of the aedeagus of *Mecyclothorax arboricola* (Fig. 40F). Standardized body length 3.5–3.6 mm.

##### Distribution and habitat.

This species is the geographically most widespread Tahitian *Mecyclothorax*, recorded from Monts Marau, Aorai, and the lower reaches of the Piti Hiti massif. Elwood Zimmerman collected the type series along the sandy shore of a small pond at 1200 m elevation on Aorai. I have examined three specimens in the Perrault collection from Aorai, 1000–1900 m elevation, and five specimens from Marau, 900–1400 m elevation. Perrault’s Pito Hiti massif localities for this species lie along the ridge from Supermahina upwards to Pihaaiateta; two specimens are labeled 1000 m elevation, and two labeled 900–1200 m.

#### 77. Mecyclothorax taiarapu

Perrault, 1989: 64

http://species-id.net/wiki/Mecyclothorax_taiarapu

##### Identification.

Among the *Mecyclothorax globosus* group, this species can be diagnosed by: 1, presence of only the posterior supraorbital seta and anterior dorsal elytral seta, setal formula 1111 (Fig. 39C); 2, well-developed transverse elytral microsculpture, a mixture of transverse sculpticells, breadth 2–4× length, and transverse lines; and 3, standardized body length 3.4–4.3 mm. The pronotal median base bears deep round punctures, about 18 each side, with the punctures anastomosing in the rugose laterobasal pronotal depressions. The discal elytral striae are deep and broad, and the discal intervals broadly convex. The head bears evident, though shallow transverse-mesh microsculpture, the more elongate sculpticells near the transverse wrinkles emanating from the frontal grooves. The neck is covered with shallow isodiametric microsculpture. The pronotal disc bears elongate transverse microsculpture, the mesh irregular leading to a predominance of transverse lines. The sculpticell margins are shallow and they cannot be traced in areas of reflected light. The male aedeagal median lobe is gracile, with an elongate ventral expansion at the apex (Perrault 1989, fig. 9); essentially a doubling of the longitudinal dimension of the ventral aedeagal expansion observed in *Mecyclothorax arboricola* males (Fig. 40F).

##### Distribution and habitat.

Individuals of this species have been found from 900–1200 m elevation on Mont Teatara, Tahiti Iti. Beetles have been found inhabiting arboreal mosses, and among the appressed leaves of the clumped *Astelia* lilies.

#### 78. Mecyclothorax ataraensis

Perrault, 1989: 63

http://species-id.net/wiki/Mecyclothorax_ataraensis

##### Identification.

Along with the previous species, *Mecyclothorax ataraensis* is diagnosable within the *Mecyclothorax globosus* group by the presence of only the posterior supraorbital seta and anterior dorsal elytral seta – setal formula 1111 – in combination with transverse microsculpture on the discal elytral intervals, but this species is distinguished by standardized body length 3.3 mm; the smallest body size recorded for any Tahitian *Mecyclothorax* species. The eyes are small, ocular ratio 1.43, yet they cover nearly all of the small ocular lobes in dorsal view, ocular lobe ratio 0.92. The pronotum is constricted basally, MPW/BPW = 1.52, and the lateral margins are subparallel anterad the right hind angles (Fig. 39D). The discal elytral striae are shallow yet distinctly punctate, with deep rounded punctures that expand the strial breadth spaced about 4× their diameter along the striae. The elytral intervals are broadly convex; each nearly flat medially. The head is glossy, with transverse wrinkles on the frons but no evident microsculpture. The pronotal disc bears an elongate transverse mesh, traceable outside areas of reflected light. The discal elytral intervals are lined with a distinct transverse mesh, the sculpticell breadth 2–3× length. The species is currently known from two female specimens.

##### Distribution and habitat.

The holotype and paratype were collected between 900–1100 m elevation on Mont Teatara, Tahiti Iti.

#### 79. Mecyclothorax rahimata sp. n.

http://zoobank.org/55998D07-D7BF-4BD2-BD5A-CF2E6A7353D5

http://species-id.net/wiki/Mecyclothorax_rahimata

##### Diagnosis.

Among the Tahitian *Mecyclothorax globosus* group species with two supraorbital setae and no dorsal elytral setae – setal formula 2101 (Figs 41A, 42A–C) – this species is diagnosable by the very large head and narrowly ovate elytra with very shallow, minutely punctate discal striae (Fig. 41A). The head is very broad relative to the pronotum and elytra; MEW/MHW = 1.90. Only *Mecyclothorax popotioaoa* Liebherr of Moorea (Fig. 41B) shares the same body shape incorporating a large head and narrow hindbody, and given the similarity in setation and microsculpture, the two species are deemed sister species. Standardized body length 3.75 mm. *Head* broad with small, little convex eyes, ocular ratio 1.42; the ocular lobe broadly convex, little projected from head, eye not covering posterior portion, ocular lobe ratio 0.75; frontal grooves well defined, convergent anteriorly, narrow posteriorly, broad and deep near frontoclypeal suture; antennae moderately elongate, submoniliform, antennomere 8 length 1.75× maximal breadth. *Pronotum* not transverse, MPW/PL = 1.08, base narrowly constricted, MPW/BPW = 1.63; lateral margins distinctly convergent anterad right hind angles, then subangularly divergent anteriorly; median base sloped upward to meet convex disc, moderately depressed, 10–11 isolated punctures each side; basal margin broadly convex between hind angles, the angles canted forward due to convexity of margin; anterior transverse impression narrow and shallow, smooth; anterior callosity slightly convex, smooth but with 10–12 very minute longitudinal wrinkles crossing each side; front angles tightly rounded, slightly protruded; lateral marginal depression very narrow, the edge carinate, depression slightly broader inside front angles; laterobasal depression only slightly depressed, surface as punctate as lateral reaches of median base, lateral and basal margins beaded. *Elytra* narrowly ovate, disc convex medially with sides sloped to near vertical juncture with lateral marginal depression; discal striae 1–6 shallow, the striae appearing very shallow to discontinuous between rounded punctures, stria 7 shallower, smooth; sutural interval coplanar with disc basally, narrowed and slightly upraised apically; discal intervals 2–4 slightly convex; humeri angulate, an upraised ridge that obliquely traverses the elytral base joined to humeral angle; lateral marginal depression narrow, edge upturned basally and beaded anterad subapical sinuation; eighth interval subcarinate laterad stria 7 from subapical sinuation to apex; lateral elytral setae 7 + 6. *Microsculpture* of vertex an evident transverse mesh, the sculpticells difficult to discern in areas of reflected light; pronotal disc with obsolete transverse mesh visible just outside areas of reflected light, pronotal base with shallow isodiametric mesh between the punctures; elytral discal intervals glossy, microsculpture absent. *Coloration* of head rufous with a brunneous cast, clypeus and labrum rufoflavous; antennomere 1 flavous, 2–3 rufoflavous, 4–11 rufobrunneous; pronotal disc rufopiceous, base and anterior callosity narrowly rufous; elytral disc rufobrunneous with silvery reflection, slightly darker basally, broadly paler apically; elytral lateral marginal depression narrowly rufoflavous apically; femora and tibiae rufoflavous.

Male genitalia. Aedeagal median lobe very small, about half the length relative to adult body length when compared to aedeagi of species with similarly sized males (Fig. 43A versus Figs 43B, C, E); median shaft nearly as broad as basal bulb, evenly curved; apex not extended beyond ostial opening, the ostial canal therefore incredibly short; apex slightly asymmetrical with tightly rounded tip oriented ventrally; flagellar plate small, length 0.3× distance from parameral articulations to apex.

Holotype male (MNHN) labeled: French Polynesia: Tahiti Nui / Mt. Marau summit el. 1445 m / 4-IX-2006 lot 04 / 17°36.530'S, 149°31.978'W / beating mixed vegetation on / summit ridge J.K. Liebherr // HOLOTYPE / Mecyclothorax / rahimata / J.K. Liebherr 2013 (black-bordered red label).

##### Etymology.

The species epithet compounds the Tahitian words rahi, or big, and mata, or face, the name signifying the disproportionately large head in beetles of this species.

##### Distribution and habitat.

The single specimen was found by beating vegetation on the summit ridge of Mont Marau, 1445 m elevation. The vegetation included low stature, 3–4 m tall *Weinmannia* and *Myrsine*, plus *Dicranopteris* ferns and *Cyathea* tree ferns. The situation was quite mesic, and the type specimen was the only carabid beetle found.

#### 80. Mecyclothorax anaana

Liebherr, 2012b: 85

http://species-id.net/wiki/Mecyclothorax_anaana

##### Identification.

This is one of only two *Mecyclothorax globosus* group species (Figs 40C, D) within which the discal elytral striae are so shallow that they are intermittently interrupted between adjacent strial punctures. From the similar appearing *Mecyclothorax hemisphaericus*, this species can be distinguished by the presence of two dorsal elytral setae; setal formula 2121. The pronotum is quite constricted basally, with the lateral margins distinctly convergent anterad the projected, right hind angles; MPW/BPW = 1.55–1.65 (n = 5). The eyes are small and little convex, ocular ratio 1.41–1.46, and the ocular lobe projects little from the gena, ocular lobe ratio 0.77–0.81. The frons is covered with an indistinct transverse mesh, the sculpticells 2–3× broad as long, and the pronotal disc is glossy with an indistinct transverse mesh visible outside areas of reflected light. The discal elytral intervals are glossy with no discernible microsculpture. The male aedeagal median lobe is broad basally and very narrow apically, the shaft as broad as the basal bulb and narrowing precipitously at the ostium (Fig. 43B), and the apex is narrowly downturned to a tightly rounded tip. Standardized body length 3.7–4.2 mm.

##### Distribution and habitat.

This species is known to inhabit Mont Mauru from 960–1110 m elevation. It is associated with arboreal microhabitats based on its discovery through beating vegetation, including *Melicope*, *Myrsine*, *Weinmannia*, and soft ferns. Individuals have also been found by applying pyrethrin fog to a bank of dead and live ferns.

#### 81. Mecyclothorax hemisphaericus

Perrault, 1989: 68

http://species-id.net/wiki/Mecyclothorax_hemisphaericus

##### Identification.

The second of the *Mecyclothorax globosus* group species with very shallow discal elytral striae that are intermittently interrupted along their length (Figs 40C, D), this species is unique in having only the anterior dorsal elytral seta; setal formula 2111. The head, pronotum and elytra are dark brunneous, with all dorsal surfaces glossy. The pronotal median base is little depressed relative to the disc, and has 8–9 deep, round punctures isolated on each side by areas of glossy cuticle. The male aedeagal median lobe narrows evenly from the basal bulb to a narrow apex (Fig. 43C), much as in males of *Mecyclothorax anaana* (Fig. 43B), but the apex is broader dorsoventrally, with a slight spatulate expansion of the tip. Standardized body length 3.6–4.3 mm.

##### Distribution and habitat.

This species inhabits Mont Marau from 900–1400 m elevation. It has been found by in dead *Dicranopteris* ferns and moss-covered *Weinmannia* trees via pyrethrin fogging.

#### 82. Mecyclothorax oaoa sp. n.

http://zoobank.org/5C043319-A520-40A6-B9E0-A7A2A903BCD6

http://species-id.net/wiki/Mecyclothorax_oaoa

##### Diagnosis.

Of the four Tahitian *Mecyclothorax globosus* group species with two supraorbital setae each side and no dorsal elytral setae, setal formula 2101 (Figs 41A, Figs 42A–C), this species can be diagnosed by the narrow, subquadrate elytra and the impressed and distinctly punctate discal elytral striae (Fig. 42A). The pronotum is only slightly transverse – MPW/PL = 1.14 – and basally constricted; MPW/BPW = 1.52. The eyes are moderately small, leaving the posterior fifth of the protruded ocular lobe uncovered; ocular ratio = 1.43, ocular lobe ratio 0.82. Standardized body length 3.7 mm. *Head* with convex frons, frontal grooves linear, convergent anteriorly, with transverse wrinkles radiating onto frons from grooves; anterior and posterior supraorbital setae proximate, separated by 3× the diameter of the depression housing the anterior seta; antennae short, nearly moniliform, antennomere 8 length 1.5× maximal breadth. *Pronotum* cordate, lateral margins concavely convergent for short distance anterad projected, obtuse hind angles, the distinct angle filled with a triangularly raised bead; median base sloped upward to meet convex disc, moderately depressed, with ~14 deep punctures each side, the punctures elongate along discal margin; anterior transverse impression broad, shallow, more defined laterally along anterior margin posterad slightly convex anterior callosity, fine longitudinal wrinkles lining impression laterally; front angles slightly protruded, very tightly rounded; lateral marginal depression narrow, edge beaded, depression broader inside front angles but beaded margin as high there as laterally; laterobasal depression a shallow extension of the discal base, smooth unilaterally, irregularly rugose on other side of type specimen. *Elytra* moderately convex, the sides gently sloped to the lateral marginal depression; discal surface between intervals 1–4 appearing flat each side (single specimen has crumpled elytra), sutural interval raised as a median callous; discal striae 1–5 moderately convex, punctate, the elongate punctures slightly enlarging strial breadth, striae 6–7 smoother, surface undulated along length; striae 1–7 variously shallower basally than on disc or obsolete for short distance posterad elytral basal groove; humeri distinctly angulate, basal groove evenly curved, MEW/HuW = 1.97; eighth interval subcarinate laterad stria 7, upraised above stria from subapical sinuation to apex; lateral elytral setae 7 + 6. *Microsculpture* of frons an evident transverse mesh, sculpticell breadth 2.0× length, neck with evident isodiametric mesh; pronotal disc with shallow but evident transverse mesh, sculpticell breadth 2–4× length; discal elytral intervals lined with well-developed transverse microsculpture including areas of transverse mesh, sculpticell breadth 2–4× length, and transverse lines. *Coloration* of head dark rufous, clypeus and labrum rufoflavous; antennomeres 1–3 rufoflavous, 4–11 rufobrunneous; pronotal disc dark rufous to match frons, lateral margin depression narrowly, median base broadly, rufous; elytral disc rufobrunneous with silvery metallic reflection; sutural interval rufous basally, rufoflavous apically, lateral marginal depression narrowly brunneous; femora rufoflavous, tibiae rufoflavous with brunneous cast.

Holotype female (MNHN) labeled: French Polynesia: Tahiti Iti / Mts. Teatara NW ridge / 1146 m el. 19-IX-2006 lot 09 / 17°47.855'S, 149°14.266'W / pyr. fog moss log C.P. Ewing / HOLOTYPE / Mecyclothorax / oaoa / J.K. Liebherr 2013 (black-bordered red label).

##### Etymology.

The species epithet is the Tahitian word oaoa, meaning narrow, the name signifying the narrowly ovate elytra characterizing this species.

##### Distribution and habitat.

The type specimen was found on Mont Teatara, Tahiti Iti in *Weinmannia* forest at 1150 m elevation. The specimen was collected by applying pyrethrin fog to a mossy log upon which grew a mature *Astelia* plant with numerous dead leaves. Two specimens of *Mecyclothorax globosoides* were also collected in this sample.

#### 83. Mecyclothorax maninapopoti sp. n.

http://zoobank.org/C5FD3B45-AAAA-4327-90B4-EA4DB1DB7912

http://species-id.net/wiki/Mecyclothorax_maninapopoti

##### Diagnosis.

This is the broadest-bodied species (Fig. 42B) of the *Mecyclothorax globosus* group to exhibit two supraorbital seta and no dorsal elytral setae, setal formula 2101. The pronotum is robust, with the lateral margins only slightly sinuate anterad the obtuse hind angles. The elytra are broadly ovate, MEW/HuW = 2.13. The discal elytral striae are narrow with elongate punctures along their length, the punctures only slightly expanding strial breadth. The discal elytral intervals are covered with transverse-line microsculpture, the lines closely packed and only occasionally connected in association with elongate sculpticells. Standardized body length 4.0 mm. *Head* with shallow, broad frontal grooves, their surface undulated along their length; eyes moderately convex, ocular ratio 1.44, ocular lobe gently protruded from gena, ocular lobe ratio 0.81; antennae short, apical segments nearly moniliform, antennomere 8 length 1.55× maximal breadth. *Pronotum* constricted basally, MPW/BPW = 1.54, but lateral sinuation anterad hind angles very brief; median base slightly depressed relative to disc, with ~20 distinct, fine punctures each side on the smooth surface, the punctures elongate along discal margin; anterior transverse impression shallow and broad at the midline, finely incised, deep for outer ¾ of each side; anterior callosity a narrow, convex roll; front angles slightly protruded, narrowly rounded; lateral marginal depression very narrow, margin beaded, only slightly broader inside front angle; laterobasal depression a smooth, U-shaped expansion of lateral depression, the surface only slightly irregular. *Elytra* broadly ovate, the sides subparallel at midlength, disc convex, the sides distinctly sloped to near vertical juncture with lateral marginal depression; elytral striae 1–6 well incised, associated intervals moderately convex, stria 7 shallower and broader though still with undulations along length; striae 1–5 shallow to obsolete basally (varying unilaterally), not deeply joined to elytral basal groove (surface oriented perpendicular to viewpoint), striae 6–7 obsolete basally; humeri subangulate, the juncture of basal broove and lateral margin tightly rounded; lateral marginal depression narrow throughout length, margin upturned; eighth interval upraised above stria 7 from subapical sinuation to apex, its surface convex, slightly bulging laterally; lateral elytral setae 7 + 5. *Microsculpture* of frons an obsolete transverse mesh, the sculpticells more visible near transverse wrinkles; pronotal disc with evident transverse mesh, sculpticell breadth 2–4× length. *Coloration* of head rufous with a piceous cast, clypeus and frons rufous; antennomere 1 flavous, 2–3 rufoflavous, 4–11 rufobrunneous; pronotal disc rufous with a piceous cast, anterior callosity, lateral margins narrowly, and median base broadly, rufous; elytral discal intervals dark rufous (paler than pronotal disc) with a silvery metallic reflection, sutural interval concolorous but scutellum and parascutellar striole rufous; lateral marginal depression narrowly rufobrunneous; femora rufoflavous, tibiae rufoflavous with brunneous cast.

Holotype female (MNHN) labeled: French Polynesia: Tahiti Nui / Mt. Marau road el. 1315 m / 10-IX-2006 lot 05 / 17°36.433'S, 149°32.333'W / pyr. fog mossy *Weinmannia* / w/ *Astelia* D.A. Polhemus // HOLOTYPE / Mecyclothorax / maninapopoti / J.K. Liebherr 2013 (black-bordered red label).

##### Etymology.

The species epithet compounds the Tahitian words mānina, or smooth, with popoti – i.e., roach or cockroach – signifying the smooth elytral striae of this beetle.

##### Distribution and habitat.

This is a species of Mont Marau, known from 1315 m elevation. The lone specimen was included in a pyrethrin fog sample from moss-covered *Weinmannia* vegetation that also produced one specimen each of *Mecyclothorax poria* and *Mecyclothorax marau*.

#### 84. Mecyclothorax hunapopoti sp. n.

http://zoobank.org/9A098D33-61C4-4C1D-8B43-2CF68C254862

http://species-id.net/wiki/Mecyclothorax_hunapopoti

##### Diagnosis.

Quite similar in appearance to *Mecyclothorax maninapopoti* in the setal formula 2101, standardized body length 4.2 mm, moderately transverse pronotum with briefly sinuate lateral margins, and ovate elytra with moderately convex elytral intervals, but this species can be diagnosed by: 1, elytral striae 1–5 deep and continuously joined to elytral basal groove; 2, discal elytral striae smooth, deep, only slightly undulated at depth, the undulations not regularly placed along the striae; and 3, elytra broader basally, more narrowly ovate behind, MEW/HuW = 2.0. *Head* with frontal grooves shallow, broad, the surface undulated by transverse wrinkles; eyes large, moderately convex, covering much of protruded ocular lobe, ocular ratio 1.43, ocular lobe ratio 0.85; antennae moderately elongate, submoniliform, antennomere 8 length 1.75× maximal breadth. *Pronotum* little transverse, moderately constricted basally, MPW/PL = 1.17, MPW/BPW = 1.51; median base not depressed relative to disc, the basal and discal surface coplanar medially; median base with ~16 punctures each side, the punctures anastomosing laterally, elongate along margin with disc; anterior transverse impression broad, very shallow medially, finely incised in lateral quarters; front angles only slightly protruded, rounded; lateral marginal depression narrow, edge beaded, depression broader inside front angles; laterobasal depression a smooth U-shaped expansion of lateral depression. *Elytra* moderately convex, the disc broadly raised with upraised, callous-like sutural intervals, the sides distinctly sloped to a near vertical juncture with the lateral marginal depression; elytral intervals distinctly convex; humeri subangulate laterad evenly curved basal groove; lateral marginal depression moderately broad outside anterior series of lateral elytral setae, evenly narrowed to carinate margin at subapical sinuation; interval 8 broadly convex, slightly upraised laterad stria 7 dorsad subapical sinuation, but all intervals convex apically so difference in convexity is minimal; lateral elytral setae 7 + 6. *Microsculpture* of frons an obsolete transverse mesh, transverse sculpticells deeper on neck impression and near pronotal margin; pronotal disc with evident transverse mesh, sculpticell breadth 2–4× length; discal elytral intervals covered with well-developed transverse lines with little tendency to form an elongate mesh. *Coloration* of frons rufopiceous, clypeus and labrum rufous; antennomere 1 flavous, 2–3 rufoflavous; 4–11 rufobrunneous; pronotal disc rufopiceous, lateral margin narrowly and median base broadly rufous; elytral disc rufobrunneous with purplish metallic reflection, sutural interval rufous basally and apically; lateral elytral depression narrowly rufoflavous; femora flavous with a brunneous cast, tibiae slightly more brunneous.

Holotype female (MNHN) labeled: French Polynesia: Tahiti Nui / Mt. Marau road el. 1185 m / 4-IX-2006 lot 02 / 17°36.440'S, 149°32.877'W / pyrethrin fog tree ferns // *Weinmannia* J.K. Liebherr // HOLOTYPE / Mecyclothorax / hunapopoti / J.K. Liebherr 2013 (black-bordered red label).

##### Etymology.

The species epithet compounds the Tahitian words huna, meaning secret or hidden, with popoti, cockroach, signifying the similarity of this species to *Mecyclothorax maninapopoti*.

##### Distribution and habitat.

This species is known only from a pyrethrin fog sample taken at 1185 m on Mont Marau. The vegetation fogged was unremarkable, consisting of a *Weinmannia* tree mixed with *Cyathea* tree fern foliage, both plants abundant on Tahiti. The sample also included one specimen each of *Mecyclothorax arboricola* and *Mecyclothorax poria*, and three specimens of the common *Mecyclothorax globosus*.

#### 85. Mecyclothorax fefemata sp. n.

http://zoobank.org/FD443C2C-1FEA-4DFA-A93F-4CA9637C7D1D

http://species-id.net/wiki/Mecyclothorax_fefemata

##### Diagnosis.

Of the eight Tahitian species in the *Mecyclothorax globosus* group with two supraorbital setae and one dorsal elytral seta – setal formula 2111 (Figs 41D, 42D, 45, 46A–C) – this species can be diagnosed by the combination of: 1, eyes convex, ocular ratio 1.47–1.50 (n = 5), the ocular lobe protruded from gena at less than 135°, ocular lobe ratio 0.80–0.85; 2, microsculpture reduced, head glossy, pronotal disc and discal elytral intervals with indistinct transverse sculpticells visible only outside areas of reflected light; and 3, larger body size, standardized body length 4.7–4.9 mm. This species (Fig. 42D) shares a constricted pronotal base and broadly ovate, convex elytra with *Mecyclothorax globosoides*, *Mecyclothorax globosus*, and *Mecyclothorax paraglobosus* (Figs 45B, 46B, C), however given a male specimen, the aedeagus with downturned apex and elongate ostial canal (Fig. 43D) is easily told from the arcuate aedeagi without an apical expansion characteristic of the other three species (Figs 47A–D). *Head* with frontal grooves well defined, narrow at depth, sinuously convergent to frontoclypeal suture; antennae elongate, filiform, antennomere 8 length 2.0× maximum breadth. *Pronotum* moderately transverse, basally constricted, MPW/PL = 1.15–1.25, MPW/BPW = 1.66-1.71; pronotal hind angles right, to slightly obtuse or slight acute, lateral margins subparallel to slightly convergent anterad angles; median base moderately depressed relative to convex disc, ~16 distinct punctures each side isolated by glossy cuticle; anterior transverse impression obsolete medially, crossed by indistinct longitudinal wrinkles in median 4/6 of breadth, anteriorly carinate in lateral 1/6 each side; front angles little protruded, broadly rounded; lateral marginal depression narrow, margin beaded, slightly broader inside front angles; laterobasal depression a deep, narrow extension of the lateral depression, very small punctures present on mesal surface. *Elytra* ovate, lateral margins evenly curved posterad angulate humeri; disc convex, sides distinctly sloped to near vertical juncture with lateral marginal depression; discal elytral striae shallow but complete, minutely and regularly punctate along length; striae 2–4 obsolete basally, not reaching elytral basal groove, sutural stria complete basally, striae 5–6 broad and shallow basally, traceable to basal groove; discal elytral intervals broadly, slightly convex; sutural stria deep to apex, striae 2–3 shallow and broad apically, striae 4–6 obsolete, and stria 7 evident inside slightly upraised interval 8, that interval only slightly more convex than the inner intervals dorsad subapical sinuation; lateral elytral setae 7 + 6. *Coloration* of frons dark rufobrunneous, neck and clypeus rufous, labrum rufoflavous; pronotal disc rufopiceous, lateral margins narrowly rufous, base more broadly so; elytral disc dark rufous, sutural interval basally rufous, apically rufobrunneous; femora and tibiae rufoflavous, the femora with piceous medial cloud.

Male genitalia. Aedeagal median lobe robust, shaft broad with expanded ventral margin at ostium (Fig. 43D); lobe apex elongate, downturned at tip as a rounded ventral expansion; ostial canal long, traversing right face of lobe from apex of ostium to ventral expansion; internal sac with dorsal and ventral ostial microtrichial patches, the flagellar plate moderately elongate, length 0.48× distance from parameral articulations to apical face.

Female reproductive tract. Bursa copulatrix columnar, length nearly 3× maximal breadth when pressed under microslide cover slip (Fig. 44A), bursal membrane thin; basal gonocoxite 1 short and broad, with apical fringe of five setae, 5–7 smaller setae arrayed along mesal half of coxite (Fig. 9G); apical gonocoxite 2 broadly subtriangular, moderately expanded basolaterally; unilaterally with 2–3 lateral ensiform setae, a dorsal ensiform seta, and an apical sensory furrow with two apical nematiform setae and two furrow pegs.

Holotype male (MNHN) labeled: SOCIETY IS: Tahiti / Tahiti Nui Mont Marau / 1280 m el. 6-XI-1999 / D.A. Polhemus pyr. fog / sta. 1 *Weinmannia* for. // HOLOTYPE / Mecyclothorax / fefemata / J.K. Liebherr 2013 (black-bordered red label).

Allotype female (MNHN) labeled as holotype.

Paratypes labeled as holotype (CUIC, 2; NMNH, 1).

##### Etymology.

The species epithet compounds the Tahitian words fefe, or curved, and mata, meaning eyes or face. The name refers to the convex eyes that differentiate this species from the one below, *Mecyclothorax maninamata*.

##### Distribution and habitat.

This species is known from 1280 m elevation on Mont Marau. The type series comprised a portion of pyrethrin fog samples from moss-covered *Weinmannia* trees. Six other species were collected during this sampling; *Mecyclothorax poria*, *Mecyclothorax ovalipennis*, *Mecyclothorax villiersi*, *Mecyclothorax arboricola*, *Mecyclothorax hemisphaericus*, and *Mecyclothorax globosus*.

#### 86. Mecyclothorax maninamata sp. n.

http://zoobank.org/6439FF2D-9514-4FA9-8596-1113C64492C3

http://species-id.net/wiki/ Mecyclothorax_maninamata

##### Diagnosis.

Among species of the *Mecyclothorax globosus* group with two supraorbital setae each side and one dorsal elytral seta – setal formula 2111 – this species can be diagnosed by the pallid body coloration (Fig. 45A) and very small eyes, ocular ratio 1.35. A horizontal diameter of the compound eye crosses 15 ommatidia. Standardized body length 3.7 mm. *Head* with frontal grooves very fine and shallow posteriorly, the convex frons immediately mesad the supraorbital carina crossed by transverse wrinkles, deeper and broader anteriorly at frontoclypeal suture, the grooves extended laterally onto labrum; ocular lobe little protruded, eyes little convex; antennae moderately elongate, submoniliform, antennomere 8 length 1.75× maximal breadth. *Pronotum* transverse, lateral margins maximally curved at lateral pronotal setae; MPW/PL = 1.29; base moderately constricted, MPW/BPW = 1.48, lateral margins subparallel for 0.14× length anterad slightly obtuse, well-defined hind angles; median base depressed relative to disc, ~7 punctures each side bordered by larger depressions mesad the laterobasal depression; anterior transverse impression broad, shallow medially, crossed by a dense array of distinct longitudinal strigae that extend nearly to pronotal anterior margin, more narrowly incised laterally near rounded, non-protruded front angles; lateral marginal depression narrow, margin beaded, depression hardly wider inside front angles; laterobasal depression consisting of two pits separated by an oblique transverse bar that crosses from base to lateral margin anterad the hind angle. *Elytra* narrowly ovate, the lateral margins evenly curved posterad the proximate, angulate humeri, MEW/HuW = 2.29; elytral disc broadly convex, the surface flat between the fourth intervals at midlength, sides downturned to near vertical juncture with lateral marginal depression; discal elytral striae moderately incised, punctate, the rounded punctures expanding strial width; striae 1–5 shallow basally, but traceable to basal groove; discal elytral intervals broadly, moderately convex; striae 1–7 well-developed apically, the associated intervals convex, eighth interval subcarinately upraised above stria 7 dorsad subapical sinuation, the interval convexly bulging from there to apex; lateral elytral setae 7 + 6. *Microsculpture* of frons obsolete, surface glossy between the transverse wrinkles, shallow transverse mesh visible on neck; pronotal disc glossy over parts, the surface appearing lapped due to irregular transverse wrinkles, an indistinct transverse mesh visible over complementary parts, sculpticell breadth 2–4× length; pronotal median base with evident isodiametric and transverse sculpticells between the punctures; discal elytral intervals with an indistinct transverse mesh, sculpticell breadth 2–3× length visible outside areas of reflected light. *Coloration* of head pale rufous; antennomeres 1–4 flavous, 5–11 rufobrunneous; pronotal disc pale rufous, all margins concolorous; elytra pale rufous, lateral marginal depressions narrowly rufoflavous; femora and tibiae flavous.

Female reproductive tract. Bursa copulatrix elongate, narrowly rounded apically, length 2.6× width when compressed under microslide cover slip (Fig. 44B); spermathecal duct broad, almost as broad as the spermathecal reservoir; basal gonocoxite 1 broad apically, narrow basally, with apical fringe of 3 setae set laterally, and 6–7 small setae arrayed along the mesal margin (Fig. 9H); apical gonocoxite 2 broad basally, narrowly arcuate apically, with broadly arcuate lateral margin; apical coxite with two elongate lateral ensiform setae, a dorsal ensiform seta, and an apical sensory furrow with two apical nematiform setae and two furrow pegs.

Holotype female (MNHN) labeled: Mt. Marau, Tahiti / 1200–1400 m / XII-1977 / J. Gourvès (on reverse) (pink paper) // Museum Paris / 1994 / Coll. G. Perrault (pink paper) // Ƶ (white paper) // HOLOTYPE / Mecyclothorax maninamata / J.K. Liebherr 2013 (black-bordered red label).

Etymology. The species epithet compounds the Tahitian words manina, in this usage meaning flat, and mata, herein meaning eye. The name therefore refers to the flat eyes diagnostic of this species.

##### Distribution and habitat.

The holotype specimen was placed at the end of the Georges Perrault collection, with the tertiary label “Ƶ” indicating his recognition of it as distinct, though undescribed. It was collected between 1200 and 1400 m elevation on Mont Marau, though the situation within which Professeur Gourvès collected it was not recorded.

#### 87. Mecyclothorax globosoides

Perrault, 1989: 62

http://species-id.net/wiki/Mecyclothorax_globosoides

##### Identification.

Along with abundant *Mecyclothorax globosus* of Marau and Aorai (Fig. 46B), and *Mecyclothorax paraglobosus* from Pito Hiti (Fig. 46C), this species (Fig. 45B) can be diagnosed by the basally constricted pronotum, broadly ellipsoid elytra, shallow yet punctate elytral striae, and setal formula 2111. But this species is easily distinguishable from the other two by the very reduced microsculpture. The frons is glossy, with micropunctures visible but indistinct transverse sculpticells discernible only near the margins of fields of reflected light. The pronotal disc is also glossy with indistinct, very elongate transverse sculpticells similarly traceable near the edges of reflected light, and the discal elytral intervals are completely smooth, their surfaces glossy, with the cuticle at most with irregular wrinkles and undulations. The male aedeagal median lobe is evenly narrowed apically to a slightly asymmetrical apex, the tip slightly downturned (Fig. 43E). Should a comparison be necessary, the lobe apex is more similar to that seen in males of *Mecyclothorax paraglobosus* (Fig. 47D) than to those of *Mecyclothorax globosus* (Figs 47A–C), though more narrowly rounded than either. Standardized body length 3.5–4.0 mm.

##### Distribution and habitat.

This is the most commonly encountered species on Mont Teatara, Tahiti Iti. Perrault’s (1989) type series included 214 individuals obtained by beating vegetation. More recent records document this species’ very diverse occurrences, with individuals found associated with living and dead growths of *Astelia*, living and rotten *Freycinetia*, moss-covered *Weinmannia* trunks and logs, and *Dicranopteris* ferns, both living and dead. and in dense banks. Individuals have been found from 1050–1195 m elevation.

#### 88. Mecyclothorax niho sp. n.

http://zoobank.org/870F14BB-D78C-4704-9FA9-1879A8BACEC5

http://species-id.net/wiki/Mecyclothorax_niho

##### Diagnosis.

This comprises one of the three species within the *Mecyclothorax globosus* group that are characterized by two supraorbital setae each side and one dorsal elytral seta – setal formula 2111 – plus subquadrate elytra, the lateral margins subparallel at midlength (Figs 45C, D, 46A). However this species can be diagnosed from the other two, *Mecyclothorax toretore* and *Mecyclothorax fuscus*, by the narrow pronotum, MPW/PL = 1.12, with very briefly sinuate lateral margins anterad the obtuse, little-projected, denticulate hind angles, and by the elytral striae that are deep and continuous to their juncture with the elytral basal groove. Standardized body length 4.1 mm. *Head* with broad and shallow frontal grooves, the mesal surface undulated and transversely wrinkled at midlength, broadly depressed anteriorly near frontoclypeal suture; eyes moderately convex, covering much of ocular lobe, ocular ratio 1.45, ocular lobe ratio 0.93; antennae moderately elongate, submoniliform, antennomere 8 length 1.75× maximal breadth. *Pronotum* narrow, subquadrate; median base slightly depressed relative to disc, 16–17 punctures each side, the punctures rounded on base, more elongate along margin with disc; anterior transverse impression broad, shallow, smooth medially, finely incised in outer third of breadth each side; frontal angles protruded, tightly rounded; lateral marginal depression narrow, margin beaded, broader and with lower bead inside front angles; laterobasal depression an expansion of the lateral depression with ~5 small punctures over the surface. *Elytra* broadest in basal half, lateral margins broadly rounded outside angulate humeri; disc moderately convex, sides moderately sloped to lateral marginal depression; striae 1–7 deep, continuous, with small micropunctures at depth on disc, the punctures not expanding strial breadth; discal elytral intervals moderately convex; all striae well impressed apically, depth of stria 2 subequal to depth of sutural stria 1; eighth interval subcarinate laterad stria 7 dorsad subapical sinuation to apex, the interval convexly bulging laterally; lateral elytral setae 7 + 6. *Microsculpture* of frons an obsolete transverse mesh, the elongate sculpticells more visible in association with transverse wrinkles, neck glossy; pronotal disc with shallow transverse mesh, sculpticell breadth 2–4× length; discal elytral intervals covered by transverse lines with little tendency to form an elongate mesh. *Coloration* of frons dark rufous, clypeus and labrum rufoflavous; antennomere 1 flavous, 2–3 plus base of 4 rufoflavous, remainder of antennae rufobrunneous; pronotal disc dark rufous to match head, depression inside front angle and median base paler, rufoflavous and rufous respectively; elytra rufous with silvery metallic reflection, the sutural interval slightly paler near scutellum, rufoflavous apically to match pale elytral apex; femora flavous, tibiae flavous with rufobrunneous cast.

Holotype female (MNHN) labeled: French Polynesia: Tahiti Iti / Mts. Teatara NW ridge 1080– / 1100 m el. 17-IX-2006 lot 07 / 17°47.778'S, 149°14.339'W / pyrethrin fog mossy logs of / *Weinmannia* J.K. Liebherr // HOLOTYPE / Mecyclothorax / niho / J.K. Liebherr 2013 (black-bordered red label).

##### Etymology.

The species epithet niho is the Tahitian word for tooth, the name referring to the denticulate pronotal hind angles.

##### Distribution and habitat.

This species has been recorded from near 1100 m elevation, Mont Teatara, Tahiti Iti. The pyrethrin fog sample from a moss-covered *Weinmannia* log also included specimens of *Mecyclothorax bryobioides*, *Mecyclothorax globosoides*, and *Mecyclothorax laevilateralis*.

#### 89. Mecyclothorax toretore

Liebherr, 2012b: 83

http://species-id.net/wiki/Mecyclothorax_toretore

##### Identification.

Among the three *Mecyclothorax globosus* group species with two supraorbital setae each side and only the anterior dorsal elytral setae, setal formula 2111, plus dense transverse-line microsculpture on the discal elytral intervals (Figs 45C, D, 46A), this species can be diagnosed by the pronotal lateral margins that are subparallel outside the laterobasal depression, the hind angles therefore projected (separating this species from *Mecyclothorax niho*), and more narrowly ovate elytra with relatively broad base, MEW/HuW = 1.89, versus the more broadly ovate elytra characterizing *Mecyclothorax fuscus*, MEW/HuW = 1.97. Elytral striae 1–4 are shallow basally and striae 5–6 are obsolete near the basal groove (Fig. 45D) as opposed to the basally deep striae 1–6 of *Mecyclothorax niho* (Fig. 45C). Elytral striae 1–6 are minutely punctate in their basal third, versus smooth and slightly undulated in *Mecyclothorax fuscus*. The vertex is covered with indistinct transverse-mesh microsculpture, the surface glossy in reflected light, and the pronotal disc bears a transverse mesh, sculpticell breadth 2–4× length, intermixed with transverse lines. No males are known for the species triplet *Mecyclothorax niho*, *Mecyclothorax toretore*, and *Mecyclothorax fuscus*. Standardized body length 4.2 mm.

##### Distribution and habitat.

This species is known from 1080–1100 m elevation on Mont Mauru. The specimen was collected from dense tangles of dead fern fronds (Liebherr 2012b).

#### 90. Mecyclothorax fuscus

Perrault, 1989: 62

http://species-id.net/wiki/Mecyclothorax_fuscus

##### Identification.

Very much like the preceding two species, *Mecyclothorax niho* and *Mecyclothorax toretore* sharing setal formula 2111 (Figs 45C, D, 46A), but deviating from the former by the more elongate sinuation of the pronotal lateral margin, the margin convergent outside the laterobasal depression (Figs 45C, 46A), and from the latter by the broadly ovate elytra; MEW/HuW = 1.97 versus 1.89 in *Mecyclothorax toretore* (Figs 45D, 46A). The pronotum is also more transverse than observed in specimens of the other two species, with MPW/PL = 1.20 verus 1.12 in *Mecyclothorax niho* and 1.14 in *Mecyclothorax toretore*. This species can also be diagnosed by the smoother discal elytral striae that are irregular along their length but are not distinctly punctate in their deepest portions. Finally, known specimens of this species are smaller than those of the other two species; standardized body length here 3.6 mm, versus 4.1–4.2 mm for the other two species. The frons and vertex bear a shallow transverse mesh that is visible through the reflected shine of the microscope light, and the pronotal disc is covered with an evident transverse mesh, sculpticell breadth 2–4× length.

##### Distribution and habitat.

The two type and only known specimens of this species were collected near the summit of Mont Aorai at 1900m elevation.

#### 91. Mecyclothorax globosus

Britton, 1948: 113; Perrault, 1978b: 152; 1989: 60

http://species-id.net/wiki/Mecyclothorax_globosus

Thriscothorax constrictus Britton, 1938: 104 (junior homonym; Britton 1948).

##### Identification.

Among the *Mecyclothorax globosus* group species with two supraorbital setae each side and only the anterior dorsal elytral setae, setal formula 2111, this species can be diagnosed from all but *Mecyclothorax paraglobosus* by: 1, small body size, standardized body length 3.5–4.1 mm; 2, broadly ovate and convexly domed elytra (Fig. 46B); 3, basally constricted pronotum, MPW/BPW = 1.53–1.64 (n = 5); and 4, discal elytral intervals covered with an evident transverse mesh, sculpticells isodiametric to transverse with breadth 2× length. This species can be reliably diagnosed from *Mecyclothorax paraglobosus* only by the conformation of the male aedeagal median lobe. In males of this species, the lobe apex is short and broadly rounded with little to no expansion dorsoventrally from the apex of the ostial opening to the tip (Figs 47A–C). The median lobe apex varies in dorsoventral breadth (Fig. 47B versus 47C), but it is always short relative to its breadth. The male aedeagal median lobe of *Mecyclothorax paraglobosus* males, in contrast, is more elongate apicad the ostial opening, with the tip slightly expanded and asymmetrically downturned (Fig. 47D). Perrault (1989) diagnosed these two species using pronotal dimensions, characterizing *Mecyclothorax paraglobosus* by a more transverse pronotum. At the extremes this is so, but the ratios of MPW/PL and MPW/BPW overlap substantially precluding diagnosis. In this species MPW/BPW = 1.53–1.64 (n = 5), and MPW/PL = 1.21–1.28. The frons and vertex bear shallow transverse-mesh microsculpture, the sculpticells on the neck more developed and therefore visible in reflected light. The pronotal disc is covered with an evident transverse mesh, sculpticell breadth 2–4× length. Standardized body length 3.5–4.1 mm.

Variation. Limited setal variation occurs among individuals of this abundant, geographically widespread species. A large series of 77 individuals – Mt. Marau, 1335 m el., 15-ix-2006 lot 01 (CUIC) – includes 2 individuals with just the posterior supraorbital seta, 1 with two supraorbital setae on the right side versus one on the left, and 74 with the usual 2111setal formula. Perrault (1978b) noted that some individuals unilaterally possessed two dorsal elytral setae, however in the much larger amount of comparative material available in 1989, he stated that the species is essentially invariant (Perrault 1989), indicating that such setal sports are exceedingly rare.

##### Distribution and habitat.

This species is broadly distributed on Monts Marau and Aorai, with recorded localities from 1100–1380 m elevation on the former, and 1067–1900 m elevation on the latter. Based on pyrethrin fog samples, these beetles occupy moss-covered *Weinmannia* growth, ferns (both low ferns such as *Dicranopteris*, and also *Cyathea* tree ferns), and the axils of *Freycinetia* plants, perferrably dead and rotting, the more likely to provide food sources such as dipteran larvae. During recent surveys, of the 318 specimens of *Mecyclothorax* collected on Mont Marau, *Mecyclothorax globosus* individuals accounted for 151.

#### 92. Mecyclothorax paraglobosus

Perrault, 1989: 62

http://species-id.net/wiki/Mecyclothorax_paraglobosus

##### Identification.

This species (Fig. 46C) can be diagnosed from all others except *Mecyclothorax globosus* by the four criteria listed under that species. From *Mecyclothorax globosus* (Figs 47A–C) it can be diagnosed by the male aedeagus (Fig. 47D) with the narrower, more elongate and apically expanded apex. The pronotum is more transverse than in *Mecyclothorax globosus*, but not diagnostically so: MPW/PL = 1.23–1.34 (n = 5). The pronotum is similarly constricted basally in both species; here MPW/BPW = 1.51–1.63. The frons and vertex are covered with an evident transverse mesh, the sculpticells more isodiametric on the neck. The pronotal disc bears a shallow transverse mesh, sculpticell breadth 2–4× length. Setal formula 2111; standardized body length 3.8–4.3 mm.

The aedeagal configuration reported here (Fig. 47D) is based on a 2006 male specimen collected at 2000 m elevation on Pito Hiti (E.M. Claridge pers. comm.). Though its aedeagal median lobe is similar to that reported by Perrault (1989: fig. 12) in: 1, the breadth of the median lobe shaft subequal to that of the basal bulb; and 2, a moderately elongate apex with slightly downturned tip, the 2006 specimen differs by exhibiting a dorsoventrally narrower apex with a more spatulate tip. There are no *Mecyclothorax* samples between 1000 and 2000 m elevation along this ridge system – e.g., the summit Pihaaiateta – and so any possible variation associated with elevation or geographic distance remains unknown. Given our ignorance, the Pito Hiti samples are assigned to *Mecyclothorax paraglobosus*, recognizing that future collections from intermediate elevations will shed light on patterns of variation.

##### Distribution and habitat.

This species is known from the Pito Hiti massif from 1000–1200 m elevation above the Supermahina housing estate. More recently it has been collected from 2000–2090 m elevation near the summit of Pito Hiti in pyrethrin fog samples of moss-covered vegetation.

#### 93. Mecyclothorax cupreus

Perrault, 1978b: 155; 1989: 68

http://species-id.net/wiki/Mecyclothorax_cupreus

##### Identification.

Instantly recognizable among *Mecyclothorax globosus* group species, indeed all Tahitian *Mecyclothorax*, by the metallic copper reflection of the dorsal body surface (Fig. 46D) in company with well-developed microsculpture: 1, head with upraised, nearly granulate transverse mesh; 2, pronotal disc with a distinct transverse mesh, sculpticells isodiametric to 3× broad as long; and 3, discal elytral intervals lined with a regular transverse mesh, the lines of sculpticels arcing over the intervals with sculpticells slightly transverse to 3× broad as long. The discal elytral striae are punctate in their basal half, the punctures elongate and restricted to the deeper portions of the striae. The male aedeagal median lobe is robust, with a broad shaft, and apex little extended past ostium, pointed, with an oblique apical face. The internal sac is well armored, with densely spiculate dorsal and ventral ostial microtrichial patches (Fig. 47E). The flagellar plate is very long, length 0.8× distance from parameral articulations to apical face. Setal formula 2121; standardized body length 4.1–5.0 mm.

##### Distribution and habitat.

This species is distributed from 1200–1900 m elevation on Mont Aorai. Most of the specimens have been collected in pitfall traps indicating activity in the litter or near the soil surface.

#### 94. Mecyclothorax externestriatus

Perrault, 1989: 66

http://species-id.net/wiki/Mecyclothorax_externestriatus

##### Identification.

Among *Mecyclothorax globosus* group species with narrow pronotal lateral margins, two supraorbital setae each side and two dorsal elytral setae, setal formula 2121, this species can be diagnosed by the following combination: 1, head glossy, frons and vertex without visible microsculpture, neck with indistinct transverse mesh near pronotal margin; 2 pronotal disc glossy, shallowly defined transverse sculpticells visible over part of surface, mostly in association with irregular transverse wrinkles; 3, discal elytral intervals lined with an elongate transverse mesh, sculpticell breadth 2–4× length; and 4, elytral striae 1–7 deep, distinctly punctate in basal half though shallow to obsolete near elytral basal groove. The pronotum is moderately transverse (Fig. 48A), MPW/PL = 1.16–1.23 (n = 2), and constricted basally, MPW/BPW = 1.51–1.63. The pronotal lateral margins are slightly convergent anterad the sharply obtuse hind angles. The pronotal median base is sparsely punctate, ~17 punctures each side isolated by glossy cuticle. This species is currently known from three female specimens. Standardized body length 3.9–4.3 mm.

##### Distribution and habitat.

This species is recorded from 900–1380 m elevation on Mont Marau. It has been collected using pyrethrin fog in association with ferns growing along the slopes of a small gulch.

#### 95. Mecyclothorax pirihao

Liebherr, 2012b: 88

http://species-id.net/wiki/Mecyclothorax_pirihao

##### Identification.

This *Mecyclothorax globosus* group species with setal formula 2121 can be diagnosed by the pronotal shape, with the very constricted base, obtuse-rounded hind angles, and lateral margins moderately divergent immediately before, and then more angularly divergent further anterad the hind angles (Fig. 48B). The discal elytral striae are smooth to minutely punctate at their depths in the basal half of the elytra. Striae 2–6 are absent from the elytral base, not continuous to the elytral basal groove. The elytra are narrow basally, the humeri angulate and proximate; MEW/HuW = 2.42–2.56 (n = 5). The frons and vertex are covered with an indistinct transverse mesh, sculpticell breadth 2–3× length, and the pronotal disc bears a more elongate mesh, sculpticell breadth 3–4× length, the areas of mesh mixed with glossy portions. The discal elytral intervals are lined with an elongate transverse mesh, sculpticell breadth 3–5× length, intermixed with areas of transverse lines. The male aedeagal median lobe is slender, evenly curved from basal bulb to ostial apex (Fig. 47F), with the apex variably displaced apically along the dorsal margin and narrowly downturned ventrally to a tightly rounded tip (Figs 3G, 47F). The ostial canal is moderately elongate and parallels the dorsal margin. The male aedeagus is very similar to that observed in males of *Mecyclothorax bryobius* (Fig. 19E), that species a member of the *Mecyclothorax altiusculus* species group. Nevertheless the key pronotal characters of narrow sinuate lateral margins place *Mecyclothorax pirihao* here. Phylogenetic analysis incorporating information from all characters will assess this incongruence. Standardized body length 4.2–5.0 mm.

##### Distribution and habitat.

This species is recorded from 880–1110 m elevation on Mont Mauru. Most of the specimens have been collected in association with ferns, either by beating or through application of pyrethrin fog. Only 2 of the 22 known specimens were collected by beating flowering plants; a mixed beating sample of *Melicope*, *Myrsine*, and *Weinmannia*.

#### 96. Mecyclothorax spinosus

Perrault, 1989: 65

http://species-id.net/wiki/Mecyclothorax_spinosus

##### Identification.

Among *Mecyclothorax globosus* group species with setal formula 2121, this species can be diagnosed by the little transverse pronotum with sharply acute hind angles (Fig. 48C); MPW/PL = 1.16–1.20 (n = 3). The upper surface has a bronzed metallic reflection on the dark rufopiceous surface, and the legs are rufobrunneous. The elytra are broadly ovate, with the margins extended laterally from the sharply angulate humeri. The elytral striae are deep, with minute elongate punctures at their depth, striae 2–7 deep and continuous to the elytral basal groove. The head is glossy, though indistinct transverse sculpticells occur in association with dense transverse wrinkles covering the frons and vertex. The pronotal disc is covered with a shallow, indistinct, and very elongate transverse mesh most visible outside the areas of reflected light. The discal elytral intervals bear a distinct, elongate transverse microsculpture intermixing elongate sculpticells, breadth 2–4× length with transverse lines. The male aedeagal median lobe is very characteristic, with an immense dorsoapical spine (Fig. 47G) extended from a straight apical face. The aedeagal shaft is broad, robust, as observed in males of *Mecyclothorax cupreus* and *Mecyclothorax cupreoides* (Figs 47E, H), and the internal sacs also share a grossly spiculate ventral ostial microtrichial patch (Figs 47E, G, H). Standardized body length 3.8–4.5 mm.

##### Distribution and habitat.

This species is recorded from 900–1200 m elevation on the lower reaches of the western ridge leading to Pito Hiti; above Supermahina housing etates and near the summit of Mapura (Perrault 1989). Individuals have been collected in ground-level litter.

#### 97. Mecyclothorax poro

Liebherr, 2012b: 91

http://species-id.net/wiki/Mecyclothorax_poro

##### Identification.

Beetles of this species are most similar to those of *Mecyclothorax spinosus* and *Mecyclothorax angulosus* (Figs 48C, D, 49A), but differentially combine the following, therefore being diagnosable from both: 1, pronotum moderately transverse, MPW/BPW = 1.25 (n = 1), and moderately constricted basally, MPW/BPW = 1.56; 2, pronotal median base sparsely punctate, 10–12 small, isolated punctures each side surrounded by glossy cuticle, plus 7–8 elongate punctures each side along juncture with pronotal disc; 3, elytra broadly subquadrate, margins extended laterally outside subangulate humeri; 4, elytral striae 2–6 basally obsolete, not continuous to elytral basal groove. The frons and vertex of the head are glossy with an indistinct transverse mesh; sculpticell breadth 2–3× length. The pronotal disc bears an indistinct transverse mesh visible outside areas of reflected light, sculpticell breadth 2–4× length, and the discal elytral intervals are covered with transverse lines irregularly joined into an irregular mesh. Setal formula 2121; standardized body length 4.4 mm.

##### Distribution and habitat.

The single adult specimen was collected in moss on a low rockface along the Faatautia River as the river exits from a large, uncollapsed lava tube at 705 m elevation on Mont Mauru (see fig. 2A, Liebherr 2012b). This riparian situation is the lowest elevational habitat within which any Tahitian *Mecyclothorax* specimen has been discovered.

#### 98. Mecyclothorax angulosus

Perrault, 1989: 65

http://species-id.net/wiki/Mecyclothorax_angulosus

##### Identification.

Sharing the basally constricted, moderately transverse pronotum with the prevous two species, though the pronotum is more constricted basally in this species; MPW/BPW = 1.58–1.70 (n = 2). The eyes are much more convex in this species (Fig. 49A), ocular ratio 1.58, and the ocular lobe is nearly rightly projected from the gena; ocular lobe ratio 0.93. Also, the elytra are more narrowly rounded in beetles of this species (Fig. 49A) versus the broadly ovate, subquadrate elytra of the previous two species (Figs 48C, D). The elytral striae are also continuous with the elytral basal groove here and in *Mecyclothorax spinosus*, versus the basally obsolete striae observed in beetles of *Mecyclothorax poro*. The dorsal microsculpture is better developed here, with the head covered with a shallow but traceable transverse mesh, and the pronotal disc bearing an elongate transverse mesh, the sculpticells causing an iridescent reflection. The discal elytral intervals are covered with dense transverse lines irregularly joined into a transverse mesh. The male aedeagal median lobe has a slender shaft and a dorsoventrally expanded apex, the expansions of similar development but the dorsal one more angulate (Perrault 1989, fig. 7). The apical face is broadly convex, and the ostial canal is short and it approaches the dorsal margin at its terminus. Setal formula 2121; standardized body length 4.0 mm.

##### Distribution and habitat.

Perrault (1989) reported the male holotype of this species from 1900 m elevation on Mont Aorai. Subsequently a second semi-teneral female was collected in 2006 from 1320 m elevation on Aorai. It was discovered by beating dead and live fern fronds.

#### 99. Mecyclothorax cupreoides

Perrault, 1978b: 156; 1989: 69

http://species-id.net/wiki/Mecyclothorax_cupreoides

##### Identification.

Along with *Mecyclothorax cupreus* (Fig. 46D), this species can be diagnosed by the brilliant coppery metallic reflection of the dorsal cuticle (Fig. 49B). Unlike *Mecyclothorax cupreus*, the frons and vertex of the head are glossy in individuals of this species, at most with patches of indistinct transverse sculpticells visible among the transverse wrinkles associated with the frontal grooves. The pronotal disc and discal elytral intervals are also glossy, with indistinct transverse sculpticells sporadically visible across their undulated surfaces. The elytra are obovate, the lateral margins narrowly rounded posterad the obtusely angulate humeri. The male aedeagal median lobes in the two species are quite similar (Figs 47E, H), supporting a close phylogenetic relationship. The median lobe shaft is broad, and the apex only slightly extended past the ostial apex, the ostial canal very short. The internal sacs in males of both species possess a spiculate dorsal ostial microtrichial patch, a less spiculate ventral ostial microtrichial patch, and an elongate flagellar plate. In males of this species, the plate length is 0.77× the distance from parameral articulations to apex. Setal formula 2121, though a male paratype (MHNB) unilaterally exhibits a subapical elytral seta on the left elytron; standardized body length 4.0–4.9 mm.

##### Distribution and habitat.

This species is known from over 100 specimens – mostly collected in pitfall traps – at localities ranging 800–1400 m elevation on Mont Marau.

#### 100. Mecyclothorax laevilateralis

Perrault, 1989: 67

http://species-id.net/wiki/Mecyclothorax_laevilateralis

##### Identification.

Beetles of this species bear superficial similarity to *Mecyclothorax spinosus* and *Mecyclothorax profondestriatus* (Figs 38B, 48C, 49C), the three species sharing a little transverse, basally constricted pronotum and bronzed dorsal body coloration. However beetles of both other species exhibit elytra with striae 6–7 as deeply incised as the inner five striae, whereas this species is characterized by the lateral striae 6–7 being shallower than the inner striae, with the impunctate stria 7 so shallow that it is nearly interrupted along its length. The elytra of these beetles are more narrowly obovate with more punctate striae (Fig. 49C) than the basally broad, ovate elytra characterizing *Mecyclothorax spinosus* (Fig. 48C). Conversely, the pronotum is more constricted basally – MPW/BPW = 1.56–1.58 (n = 4) – than observed in individuals of *Mecyclothorax profondestriatus*; MPW/BPW = 1.51–1.53 (n = 2). The frons is glossy with no apparent sculpticells (the shagreened frontal condition of the photographed male [Fig. 49C] is not observed in other specimens), and the pronotal disc is glossy with transverse lines visible near the margins of reflected light. The discal elytral intervals are glossy with only limited areas of transverse mesh visible across the highly reflective surface. The male aedeagal median lobe is small relative to the male body size in comparison to *Mecyclothorax spinosus* (Figs 47G versus 47I), but of similar relative size to that of *Mecyclothorax profondestriatus* (Figs 40D, 47I). The median lobe is evenly arcuate with a simple apex that has a slightly downturned tip; a similar though less exaggerated conformation than observed in *Mecyclothorax profondestriatus* males. Setal formula 2121; standardized body length 3.6–4.1 mm.

##### Distribution and habitat.

This species has been recorded from 900–1100 m elevation on Mont Teatara, Tahiti Iti. All specimens with ecological data have been associated with moss-covered trunks or horizontal logs, with collections made by beating small trunks or through application of pyrethrin fog to downed logs.

#### 101. Mecyclothorax globulosus

Perrault, 1978b: 159; 1989: 67

http://species-id.net/wiki/Mecyclothorax_globulosus

##### Identification.

As one of the dorsally glossy members of the *Mecyclothorax globosus* group with setal formula 2121 (Figs 48C, D, 49B–D), this species can be recognized by the basally broad, subparallel elytra (Fig. 49D). The eyes are small and the ocular lobes are little protruded – ocular ratio 1.42, ocular lobe ratio 0.78 – with a horizontal diameter of the eye maximally crossing 16 ommatidia. The discal elytral striae 1–6 are broad and slightly wavering in their deepest portions, but not punctate. Stria 7 is deeper and more narrowly incised than stria 6. Striae 3–5 are shallower near the elytral basal groove than on disc, and stria 3 is incomplete there, i.e., obsolete basally. The frons is covered with an evident transverse mesh, the sculpticells clearly visible between the transverse wrinkles associated with the frontal grooves. The pronotal and elytral discs are glossy, with indistinct, elongate transverse mesh microsculpture visible outside the fields of reflected light. The male aedeagal median lobe shaft is arcuately curved with a narrowly extended apex bearing a ventrally pointed tip (Perrault 1989, fig. 14). Standardized body length 3.4–3.6 mm.

##### Distribution and habitat.

This species is known from three specimens collected 1000–1400 m elevation on Mont Aorai, one collected in a vinegar pitfall trap (Perrault 1989).

## Discussion

The Tahitian *Mecyclothorax* species are intensely philopatric, with 96 of the 101 known species restricted to a single massif. The neighboring Marau and Aorai ridge systems currently house the greatest known diversity of species (Table 1), with three species – *Mecyclothorax gourvesi*, *Mecyclothorax sabulicola*, and *Mecyclothorax globosus* – shared between the two massifs (Table 2). Moving clockwise on the Tahiti Nui volcano (Fig. 1), the only other widespread species are shared between Aorai and the ridge system including Pihaaiateta and Pito Hiti; i.e. *Mecyclothorax fosbergioides*, *Mecyclothorax jarrigei*, and again, *Mecyclothorax sabulicola*. The mountains of Presqu`Île de Taiarapu – i.e., the Tahiti Iti volcano – and the Mont Mauru massif of Tahiti Nui plus the island of Moorea are populated strictly by precinctive species (Table 1).

**Table 1.** The number of specimens collected, known species diversity, and percent endemism for *Mecyclothorax* species occupying the massifs of Tahiti Nui, Tahiti Iti volcano, and the island of Moorea (Liebherr 2012a).<br/>

**Island/massif**	**No. specimens**	**No. species groups**	**No. spp. (endemic spp.)**	**% endemism**
Tahiti Nui/Marau	947	7	31(28)	90%
Tahiti Nui/Aorai	461	9	30(25)	83%
Tahiti Nui/Pito Hiti	157	9	21(18)	86%
Tahiti Nui/Mauru	40	4	7(7)	100%
Tahiti Iti	387	5	17(17)	100%
Moorea/Tohiea	90	4	7(7)	100%

**Table 2.** Chorographic depiction of closely related representative species from areas of endemism within Tahiti and Moorea; modified from Perrault (1992, table 10.2). Boxed species names are considered to represent clades, although relationships within clades are not proposed. Vertically aligned species without any close relatives in other areas of endemism are considered sister taxa. Characters used to group species include setation, elytral conformation, microsculpture, coloration, and configuration of the male aedeagal median lobe and parameres (see text).<br/>

**Group**	**Moorea**	**Marau**	**Aorai**	**Pito Hiti**	**Mauru**	**Teatara**
*muriauxi*		*subquadratus*		*claridgeiae*		
				*mahina*		
		*muriauxi*				
			*obtusus*			
		*poria*	*gerardi*	*jeanyvesi*		
		*brevipennis*		*mapura*		
		*quadraticollis*				
*fosbergi*			*fosbergioides------*	*fosbergioides*		
				*fosbergi*		
*altiusculus*	*menemene*		*jarrigei------------*	*jarrigei*		
		*hamatus*	*altiusculoides*	*aano*		
			*tuberculatus*			
	*pahere*	*pseudaltiusculus*	*altiusculus*			*paraltiusculus*
		*ovalipennis*	*parovalipennis*			
		*ballioides*	*bryobius*		*tihotii*	*bryobioides*
						*ferruginosus*
						*sinuatus*
*dannieae*				*papau*		
				*mahina*		
			*brittoni*	*everardi*		
						*ramagei*
						*teatara*
		*villiersi*	*tahitiensis*	*pitohitiensis*		
			*dannieae*			
		*fairmairei*	*negrei*			
			*aorai*		*tutei*	
			*cooki*			
		*marau*				
**Table 2.** Continued.						
*striatopunctatus*		*striatopunctatus*	*wallisi*			
			*pomarei*			
		*bougainvillei*				*curtisi*
*marginatus*			*marginatus*	*hoeahiti*		
*viridis*	*mapo*	*castaneus*	*viridis*	*ninamu*		
				*kokone*		
		*kayballae*		*paahonu*		
	*fatata*	*balli*	*ata*	*ehu*	*putaputa*	
*gourvesi*			*zimmermani*			
	*perraulti*	*gourvesi-------------*	*gourvesi*	*gourvesioides*		*acutangulus*
		*papuhiti*				
*tuea*				*tuea*		
*globosus*		*taatitore*	*georgettae*			
	*mahatahi*		*konemata*			*cupripennis*
						*profondestriatus*
						*vaifaufa*
		*arboricola*				
		*sabulicola----------*	*sabulicola-------*	*sabulicola*		
						*taiarapu*
						*ataraensis*
	*popotioaoa*	*rahimata*				
		*hemisphaericus*			*anaana*	
		*maninapopoti*				*oaoa*
		*hunapopoti*				
		*fefemata*				
		*globosus------------*	*globosus*	*paraglobosus*		*globosoides*
		*maninamata*	*fuscus*		*toretore*	*niho*
		*cupreoides*	*cupreus*	*spinosus*		
		*externestriatus*	*angulosus*		*poro*	
					*pirihao*	
			*globulosus*			*laevilateralis*

The two massifs with the greatest elevational disparity – Aorai and Pito Hiti – also exhibit the most disparate fauna, with members of 9 of the 10 species groups found on each mountain. Though the species groups are taxonomic constucts based on key characters, and therefore unlikely to represent monophyletic groups in all cases, the various groups represent a diversity of body forms. Thus a broader representation of species groups is likely to signify broader representation of heretofore unidentified clades across the Tahitian *Mecyclothorax* radiation. The converse may also be invoked, whereby the small island of Moorea supports seven known species representing only four species groups. Also, the geologically youngest area, Tahiti Iti (Table 3), houses 17 known species, but these represent only 5 of the 10 groups. For the present we can discount further discussion of diversity on Mauru given the very preliminary state of biological sampling on that mountain (Liebherr 2012b).

**Table 3.** Comparisons of island or lake size, species diversity, percent endemism, and areal extent of species for several diverse and geographically restricted animal radiations. The criterion “km^2^/species” represents the average range size per species within a restricted area (island or lake) assuming all species are completely allopatric (i.e., parapatric). For Maui *Drosophila*, this value is computed two ways: 1, using all species found on the island, both endemic to the island and widespread on other islands; and 2, counting only species endemic to that island (in parentheses).<br/>

**Area of endemism**	**Taxon**	**Area (km^2^)**	**No. species**	**% endemism**	**km^2^/species**
Tahiti	*Mecyclothorax*	1,045	101	100	10.3
Tahiti Nui	*Mecyclothorax*	788	84	100	9.4
Tahiti Iti	*Mecyclothorax*	257	17	100	15.1
Moorea	*Mecyclothorax*	134	7	100	19.1
Hawaiian Islands	*Mecyclothorax*	14,899†	239^1^	100	62.3
Maui	*Mecyclothorax*	1,887	142^1^	100	13.3
Haleakala	*Mecyclothorax*	1,440	115^1^	100	12.5
West Maui	*Mecyclothorax*	447	27^2^	100	16.6
Hawaiian Islands	*Drosophila*	16,329†	415^3^	100	39.3
Maui	*Drosophila*	1,887	123^3^	53	15.3(29.0)
Haleakala	*Drosophila*	1,440	103^3^	36	14.0(38.9)
West Maui	*Drosophila*	447	58^3^	21	7.7(37.2)
Hawaii Island	*Laupala*	10,430	6^4^	100	1738.3
Hawaii Island	Cave *Oliarus*	10,430	7^5^	100	1490.0
Lake Tanganyika	Cichlidae	32,900	250^6^	95-99	131.6
Lake Victoria	Haplochromini	68,800	~700^6^	95-99	98.3
Lake Malawi	Haplochromini	29,600	~700^6^	95-99	42.3

† Summed areas of islands occupied by taxon.

References:

^1^ Liebherr (2005b, 2006, 2008, 2009), Liebherr and Krushelnycky (2011), Liebherr (unpubl. data)

^2^ Liebherr (2011)

^3^ Hardy (1965, 1966, 1977, 1978), Hardy and Kaneshiro (1968, 1969, 1971, 1972, 1975, 1979), Hardy et al. (2001), Kaneshiro and Kambysellis (1999), Lapoint et al. (2009), Magnacca and O’Grady (2008a, 2008b, 2009), Magnacca and Price (2012), O’Grady et al. (2001, 2003a, 2003b), Perreira and Kaneshiro (1990); K.N. Magnacca (pers. comm.)

^4^ Mendelson and Shaw (2005)

^5^ Wessel et al. (2013)

^6^ Turner et al. (2001)

One would predict that populations of widespread species occupying several massifs should be found in proximate elevational zones of habitat on those neighboring massifs. Based on recorded localities, this is so. The very large-bodied *Mecyclothorax fosbergioides* (Fig. 15B) is known from two specimens collected at 1900 m elevation on Aorai, and at 2070 m elevation on Pito Hiti. These two localities are separated by less than 4 km distance, that distance encompassing a col between the summit of Aorai and Mont Orohena, the high peak adjacent to Pito Hiti. Similarly, specimens of *Mecyclothorax jarrigei* are known from 2000 m elevation on both Aorai and Pito Hiti. The summits of Aorai and Marau are also separated by 4 km distance along the Diadème, a very thin, cristate ridge also known as Mont Te Taro O Maiao. This ridge runs from the summits down to slightly more than 1300 m elevation. The three species shared between Aorai and Marau have all been found in this elevational zone: 1, *Mecyclothorax gourvesi* from 1210–1400 m elevation on Aorai and 1125–1400 m on Marau; 2, *Mecyclothorax sabulicola* from 1000–1900 m elevation on Aorai and 900–1400 m on Marau; and 3, *Mecyclothorax globosus* from 1067–1900 m elevation on Aorai and 1100 – 1380 m on Marau. *Mecyclothorax sabulicola* is known to occupy habitats on the Pito Hiti massif ranging from 900 – 1200 m elevation. However sampling along the elevational transect from Mapura to Pito Hiti is extensively incomplete, and so it is predicted that *Mecyclothorax sabulicola* will be found in higher elevational habitats on this massif.

All other species occupying these three mountains are precinctive to only one, demonstrating that the narrow ridges and cols have not maintained sufficient dispersal avenues to preclude geographic isolation and subsequent speciation. Such isolation has occurred even though the beetles may currently be found in the same elevational zones as the widespread species. For example, the members of the species triplet *Mecyclothorax hamatus*+*Mecyclothorax altiusculoides*+*Mecyclothorax aano* (Figs 16B–D, 18B–E, Table 2) are distributed nearly to the summits of Marau and Aorai, and Pito Hiti respectively. Their respective elevational ranges are 1000–1400 m, 1000–1900 m, and 2000 m; no different than the elevational ranges of the widespread *Mecyclothorax sabulicola* and *Mecyclothorax globosus*. The sister species *Mecyclothorax marginatus* and *Mecyclothorax hoeahiti* are known from the summits of Aorai and Pito Hiti respectively. These two species are anatomically isolated members of the Tahitian *Mecyclothorax* radiation attesting to their adelphotaxon status. In this instance the 4 km col joining their native mountains has not supported sufficient populations and attendant gene flow to have precluded speciation.

A slightly more complicated pattern affirms the existence of ecological factors beyond mere landscape physiognomy as critical to speciation. The four species exhibiting lamellate male parameres – *Mecyclothorax villiersi*, *Mecyclothorax tahitiensis*, *Mecyclothorax pitohitiensis*, and *Mecyclothorax dannieae* (Figs 23A–C, 24E, F, 26B, C, 27D, E) – may be considered a clade based on the uniquely derived and complex, broadened apex of the right paramere. Two of the four species occupy Aorai, with singletons on Marau and Pito Hiti (Table 2). All of the species are found to the summits of their respective mountains: 1, *Mecyclothorax villiersi* from 1000–1400 m; 2, *Mecyclothorax tahitiensis* 1750–2042 m; 3, *Mecyclothorax dannieae* 1000–2000 m; and 4, *Mecyclothorax pitohitiensis* 2000–2080 m. In this instance, representative species have evolved on each mountain, yet there exists extensive sympatry on Aorai between *Mecyclothorax tahitiensis* and *Mecyclothorax dannieae*. Based on derived characters of setation and the male aedeagus, these four species are phylogenetically related as (*Mecyclothorax dannieae* (*Mecyclothorax villiersi* (*Mecyclothorax tahitiensis* + *Mecyclothorax pitohitiensis*))). The latter three species can be grouped by the derived position of the median lobe ostial canal, positioned along the dorsal margin of the lobe (Figs 24E, F, 27E) versus the plesiomorphic position in *Mecyclothorax dannieae* males on the right face of the lobe (Fig. 27D). Both *Mecyclothorax dannieae* and *Mecyclothorax villiersi* exhibit the plesiomorphic presence of the subapical elytral seta, setal formula 2222, whereas the seta is synapomorphously lost in *Mecyclothorax tahitiensis* and *Mecyclothorax pitohitiensis*. Though the curved median lobe apex is shared between *Mecyclothorax villiersi* and *Mecyclothorax pitohitiensis* (Figs 24F, 27E), *Mecyclothorax tahitiensis* and *Mecyclothorax pitohitiensis* synapomorphously share: 1, expanded pronotal margins at the hind angles, with the basal margin extended posteriorly; and 2, less convex eyes (Figs 23A–C versus 26B, C). Given this provisional hypothesis, it follows that *Mecyclothorax dannieae* diverged first, with isolation at lower altitudes along the Aorai ridge as a possible hypothesis for the establishment of allopatry. Subsequent speciation of the three species characterized by the dorsal position of the ostial canal first isolated *Mecyclothorax villiersi* on Marau, and finally *Mecyclothorax tahitiensis* and *Mecyclothorax pitohitiensis* on the neighboring summits of Aorai and Pito Hiti. This putative pattern, then, includes both localized speciation associated with elevationally mediated isolation along a single ridge, and also more dramatic isolation and subsequent speciation of populations on isolated massifs.

Numerous sets of representative species were proposed by Perrault (1992, table 10.2), these related species living on different massifs. Perrault recognized *Mecyclothorax balli* and *Mecyclothorax ata* as very similar and putatively closely related species, the former from Marau and the latter from Aorai. Recent survey activities have added three more species to this complex, with representatives now known from Moorea (*Mecyclothorax fatata*), Pito Hiti (*Mecyclothorax ehu*), and Mauru (*Mecyclothorax putaputa*) (Table 2).

Analogous additions of recently discovered species to complexes hypothesized by Perrault include the addition of Moorea’s *Mecyclothorax pahere* (Liebherr 2012a) to a group including *Mecyclothorax pseudaltiusculus*, *Mecyclothorax altiusculus*, and *Mecyclothorax paraltiusculus*, and of *Mecyclothorax tihotii* (Liebherr 2012b) to *Mecyclothorax ballioides*, *Mecyclothorax bryobius*, and *Mecyclothorax bryobioides* (Table 2), from Marau, Aorai, and Teatara of Tahiti Iti respectively. The great overall similarity within these sets of representative species, coupled with setational variation and extensive male genitalic evolution allows facile recognition of these new species. Future survey activities should focus on “missing entries” in these sets of representative species. For example, undiscovered species in the *Mecyclothorax pahere*–*Mecyclothorax paralitusculus* group from are predicted from Pito Hiti and Mauru. Based on the occurrence of *Mecyclothorax altiusculus* from 1255–1900 m elevation on Aorai, it should be present at Pito Hiti, and would be predicted near the summit of Mauru.

Sympatric most-similar species are also recorded in this radiation, most often from Mont Teatara, Tahiti Iti (Table 2). Among these are the sister species *Mecyclothorax ferruginosus* and *Mecyclothorax sinuatus*, taxonomically isolated taxa in the *Mecyclothorax altiusculus* group. *Mecyclothorax ramagei* and *Mecyclothorax teatara* of the *Mecyclothorax dannieae* group are diagnosable by setation (Figs 22E, 23D) and body size, but their pronotal configuration, elytral shape, and male genitalia (Figs 24D, G) are exceedingly similar. Given that no other species share such characters, they are also best interpreted as sympatric sister species. Two analogous complexes of most-closely related species in the *Mecyclothorax globosus* group also occur in Tahiti Iti; *Mecyclothorax profondestriatus* and *Mecyclothorax vaifaufa*, and *Mecyclothorax taiarapu* and *Mecyclothorax ataraensis*. In Tahiti Nui, only *Mecyclothorax papau* and *Mecyclothorax manina* of Pito Hiti represent taxonomically isolated, sympatric sister species. Future discoveries may broaden the geographic distributions of these complexes, with species on the closest Tahiti Nui massif, Mont Mauru predicted as closest relatives of the Tahiti Iti complexes.

The *Mecyclothorax* fauna of Moorea is small, in keeping with its small island area (Table 3). Moreover, no two Moorean species are most closely related. Instead, they are all independently related to various species of Tahiti. In five of the seven instances, a Moorean species falls into a complex of Tahitian species that also includes a representative species on Marau (Table 2). One of these examples enlists the newly described, big-headed species, *Mecyclothorax rahimata* of Marau, as the adelphotaxon to *Mecyclothorax popotioaoa* from Mont Tohiea (Figs 41A, B). No other big-headed species is known from any other Tahitian massif. In the other two instances, the complex including the Moorean species has a representative inhabiting Aorai. The combination of multiple independent relationships to a geographically adjacent or next removed massif in Tahiti is consistent with the hypothesis that the Moorean *Mecyclothorax* fauna has been derived by overwater dispersal from Tahiti (Liebherr 2012a). Moreover, no member of the most plesiotypic Tahitian species group – the *Mecyclothorax striatopunctatus* group (Liebherr 2012a) – is currently known from Moorea. Should such a member be found on Moorea, the placement of that species in the phylogenetic scheme of Tahitian *Mecyclothorax* would have to be carefully evaluated to determine whether it might represent the closest extant ancestor to the Tahitian *Mecyclothorax* colonist. Based on current knowledge, *Mecyclothorax striatopunctatus* of Marau fills that role (Liebherr 2012a).

*Species diversity and area* – The remarkable diversity of Tahitian *Mecyclothorax* raises the question of just how diverse that radiation is relative to other speciose animal radiations. The numbers of species in various radiations can be compared however the areal extent of the geographic range of those taxa must be taken into account, as larger aggregate ranges once vicariated should support more speciation events, the dispersal capacities of the taxa being equal. The index chosen to assess this concentration of diversity is the area inhabited by the entire taxon of interest divided by the number of species (Table 3). The average area per species criterion assumes that all species are parapatric; clearly not the case. But it is a straightforward index that allows comparison of highly diverse groups in larger areas to somewhat less rich radiations in very restricted areas. For the Society Islands, *Mecyclothorax* species are most highly concentrated in Tahiti Nui, and somewhat less so in Tahiti Iti (Table 3). There may be numerous factors that skew such a criterion, not the least the level of sampling in the different areas. In this instance, more species have been recorded from Piti Hiti with less than half the specimens collected there than in Tahiti Iti (Table 1), supporting a hypothesis of greater diversity on the Pito Hiti massif. Nevertheless, Mont Rooniu, isolated to the east of Mont Teatara in Tahiti Iti, has never been explored entomologically, so we cannot assume Tahiti Iti represents only a single area of endemism.

The Hawaiian Islands support the second great radiation within the genus *Mecyclothorax* (Britton 1948; Table 3). The Hawaiian and Tahitian *Mecyclothorax* faunas are hypothesized to have been founded on Maui and Tahiti respectively – i.e., 1.8–1.4 Ma (Table 4) – by a colonizing propagule drawn from the geographically widespread, flight capable Australian *Mecyclothorax punctipennis* (MacLeay) (Britton 1948; Moore 1984; Liebherr 2012a). Area per *Mecyclothorax* species is about 30% greater on Maui than Tahiti, with Tahiti Nui the most diverse spot per unit area of any portion of the *Mecyclothorax* distributional range. In both radiations, species are extensively philopatric, with no species found on more than one island. The major difference between the islands of Tahiti and Haleakala lies in the much more extensive erosional valley formation that has occurred in Tahiti (Hildenbrand et al. 2008). More than 350 km^3^ of volcanic material has been removed from this 1045 km^2^ island; an average removal of material to a depth of 330 m across the entire island surface. This erosion has been concentrated across the north and south quadrants of the main shield volcano where it experienced mass wasting events, with subsequent erosion over the past 500 ka producing the deep valleys that isolate Marau, Aorai, Pito Hiti and Mauru; i.e., the Fautaua, Tuauru, and Papenoo Valleys (Fig. 1). Though Haleakala has experienced repeated bouts of volcanism, resulting in older Kula Lavas being subdivided and fragmented by younger overflowing Hana lavas (Sherrod et al. 2003), extensive erosion and mass wasting has not dramatically dissected the volcano’s surface (Moore et al. 1989) to the degree observed in Tahiti. Thus the greater concentration of *Mecyclothorax* diversity in Tahiti may be ascribed at least in part to its more advanced state of geological disintegration.

**Table 4.** Comparisons of diversity, ages of origin, and resultant calculated net diversification interval (N.D.I.) (Coyne and Orr 2004), for several diverse and geographically restricted animal radiations. Net diversification interval estimates average species duration, and is calculated as: t / (ln N – ln N_0_), where t = age of origin of group, N = extant number of species, and N_0_ = initial number of species in the group; i.e., 1 for monophyletic taxa.<br/>

**Area of endemism**	**Taxon**	**No. species^1^**	**Origin (Ma)**	**N.D.I.**
Society Islands	*Mecyclothorax*	108	1.4^2^	0.30
Hawaiian Islands	*Mecyclothorax*	239	1.8^3^	0.33
Hawaiian Islands	*Drosophila*	415	5.3^3^, 26–30^4^	0.88, 4.3–5.0
Hawaii Island	*Laupala*	6	0.78^3^	0.44
Hawaii Island	Cave *Oliarus*	7	0.13 ^3, 5^, 0.78^3^	0.07, 0.40
Lake Tanganyika	Cichlidae	250	9.0–12.0^6^	1.6–2.2
Lake Victoria	Haplochromini	~700	0.19–0.27, 5.0^7^	0.03–0.04, 0.76
Lake Malawi	Haplochromini	~700	2.4–4.6^7^	0.37–0.70

References:

^1^ See Table 3 for species diversity references

^2^ Hildenbrand et al. (2004), Guillou et al. (2005), Uto et al. (2007)

^3^ Sherrod et al. (2007)

^4^ DeSalle (1992); Russo et al. (1995)

^5^ Wessel et al. (2013)

^6^ Cohen et al. (1993), Takahashi and Koblmüller (2011)

^7^ Genner et al. (2007), Koblmüller et al. (2008)

Among Pacific insects, the Hawaiian *Drosophila* radiation represents the model for understanding processes that brought about evolutionary end products of an adaptive radiation; (Carson and Kaneshiro 1976; Kambysellis et al. 1995; Kaneshiro 1988). There are currently 415 described Hawaiian *Drosophila*, with the radiation’s founding hypothesized to have occurred 26–30 Ma (DeSalle 1992; Kaneshiro et al. 1995) after emergence of Kure, now a member of the Northwest Hawaiian Islands (Table 4). Maui houses 123 *Drosophila* species, somewhat fewer than the 142 species of *Mecyclothorax* that live there. Moreover, individual *Drosophila* species are much more geographically widespread, with only 53% of the species precinctive to Maui, and 36% and 21% precinctive to the two volcanoes comprising Maui; Haleakala and the West Maui Mountains. The Maui *Drosophila* fauna has been built up by extensive dispersal from various older islands. Thus the Maui *Drosophila* fauna is extensively polyphyletic. Given that the most plesiotypic Hawaiian *Mecyclothorax* species, *Mecyclothorax montivagus* (Blackburn), occurs on Haleakala, it may be presumed that the *Mecyclothorax* radiation was founded there (Liebherr 2012a). Thus the Haleakala *Mecyclothorax* radiation is paraphyletic, with dispersal from Maui to other islands such as Oahu and Hawaii Island a principal feature of the radiation. Such an inverted pattern of in situ diversification with outbound dispersal suggests that the *Mecyclothorax* speciation rate on Maui may be greater than that of *Drosophila*. Conversely, the flies are much better colonists, with 157 species, 104 precinctive (K.N. Magnacca pers. comm.), known from Hawaii Island, versus only 30 precinctive *Mecyclothorax* species (Liebherr 2008) known from there. Better dispersal ability accelerates *Drosophila* speciation on newly available islands of the Hawaiian archipelago.

Worldwide, the adaptive radiations of cichlid fishes in the East African Rift lakes serve as textbook examples of adaptive radiation (Turner et al. 2001; Futuyma 1986, 2009). Diversification of these fishes has been attributed to trophic specialization involving extensive modifications of the pharyngeal jaws. Occupation of shoreline habitats coupled with oscillating Pleistocene water levels are proposed as factors that accelerated speciation, with low water levels fragmenting populations, thereby allowing evolutionary divergence. At the return to higher water levels, secondary sympatry led to reinforcement of any incipient reproductive isolating mechanisms. Examining the species/area criterion for Cichlidae of Lake Tanganyika, and the cichlid tribe Haplochromini for Lake Victoria and Lake Malawi (Table 3) shows that diversification of these fishes has taken place across a much more expansive geographic setting than required for the island insect radiations of *Mecyclothorax* and *Drosophila*.

*Species diversity and time* – Comparing the rates at which species diversity has been generated across these radiations shows that Pacific island insects have among the fastest speciation rates on Earth (Table 4; Coyne and Orr 2004, table 12.1). Based on the numbers of extant species, and the origins of their respective radiations via single colonization events on islands of known age (Liebherr 2012a), both the Society Island and Hawaiian *Mecyclothorax* radiations have been built up from ancestral species persisting an average of about 0.3 Myr (Table 4). These estimates are in line with 0.20–0.30 Myr species’ durations hypothesized using paleoclimatic data for Haleakala and West Maui sister-species pairs of *Mecyclothorax*, *Drosophila*, and land snails in the families Achatinellidae and Amastridae (Liebherr 2011). Somewhat longer species durations have been reported for *Laupala* crickets (Table 4; Mendelson and Shaw 2005; Sherrod et al. 2007), and for cave-inhabiting *Oliarus* planthoppers (Wessel et al. 2013), two monophyletic groups occupying Hawaii Island. However, speciation in *Oliarus* planthoppers may be nearly an order of magnitude faster because the lava tube caves they inhabit formed only as Hawaii Island entered its post-shield building volcanic phase 130,000 years ago (Sherrod et al. 2007). *Oliarus* speciation may be even more rapid, as many of the caves inhabited by the planthoppers are only 5,000–25,000 years old (Wessel et al. 2013). The 0.88 Myr average species duration estimate for *Drosophila* (Table 4) is based on setting the age of the radiation at the origin of Kauai (Sherrod et al. 2007). Taking the hypothesized 26–30 Ma age of origin of *Drosophila* in the Hawaiian Chain (DeSalle 1992; Russo et al. 1995), a much larger value is obtained. Because this latter calculation does not take into account all *Drosophila* speciation events that occurred on the northwest Hawaiian Islands, its use should be discounted in favor of the smaller value. These species durations are on the order of, or shorter than, those observed in the Lake Tanganyika and Lake Malawi cichlid fish radiations though not as rapid as hypothesized for the haplochromine cichlids of Lake Victoria (Table 4). The age of origin of the Lake Victoria radiation has been constrained by an assumption of Pleistocene drying of the lake (Genner et al. 2007), though the extent of that drying event remains controversial (Fryer 2001). As the Lake Victoria cichlid species flock diverged from its sister taxon – the East African river haplochromine cichlids – about 5 Ma (Genner et al. 2007), an alternate hypothesis putting the age of the Lake Victoria radiation at 5.0 Ma results in an average species duration of 0.76 Myr, a value similar to that obtained for the cichlid radiations in the other two lakes. Summarizing these findings, it is clear that Pacific island insect taxa offer the most robust examples of rapid speciation on Earth, phenomonologically uniting rapid speciation with extreme levels of endemism while minimizing ambiguity concerning ages of the occupied areas and the probability of taxic dispersal from adjoining habitats.

## Conclusions

And so we return to the question posed at the beginning of this article; why so many species of *Mecyclothorax* across so little space over such a short period of time? Flightlessness is an obvious factor facilitating diversification of these beetles. Species generally differ on neighboring massifs, showing that any intervening ridge-like habitats are not sufficient avenues of dispersal to support gene flow required to maintain species integrity. The erosional history of Tahiti (Hildenbrand et al. 2008) has largely isolated populations of these sedentary beetles on linear ridge systems. Isolation by distance may play a part in the divergence of species on one ridge (Hoelzer et al. 2008), though a robust phylogenetic analysis is needed to test such intra-ridge species relationships. Moreover, we do not have comprehensive knowledge of *Mecyclothorax* distributions on the flanks of the ridges, and given their sheerness we will have to estimate that through better sampling of accessible habitats. Also, the rapid development of deep valleys over the past 500,000 ka has resulted in nearly simultaneous isolation of populations on the various ridge systems. This rapid disintegration of Tahiti has operated as an irreversible “species pump” analogous to that hypothesized for African cichlid fishes (Takahashi and Koblmüller 2011), enabling nearly simultaneous speciation of a cosmopolitan ancestor into multiple daughter species. With the progressive disintegration of Tahiti, then, species durations may be decreasing relative to the period when the volcano had just completed its shield-building phase.

Sexual selection has been invoked as a factor facilitating speciation in Hawaiian *Drosophila* (Kaneshiro, 1988). The role of sexual selection in *Mecyclothorax* beetles of the Society and Hawaiian Islands remains unstudied. Both speciose island radiations have been derived from the same geographically widespread, winged Australian colonizing taxon (Liebherr 2012a). The entire Australian *Mecyclothorax* fauna comprises fewer than 20 species (Moore et al. 1987; Baehr 2003, 2009), the vast majority of which are characterized by vestigialized flight wings. If sexual selection operates to facilitate *Mecyclothorax* diversification, why has accelerated speciation not occurred in Australia when it amply characterizes the Tahitian and Hawaiian radiations? It seems much more reasonable to view the greatly accelerated speciation rates observed in the island radiations as a product of the places, not of the players. Such a conclusion does not deny that great genitalic evolution has occurred among the island species, as male aedeagal configuration is a potent diagnostic feature. Yet, many of the closely related species are allopatric (Table 2), suggesting the sexual signals accompanying speciation for those taxa evolved in isolation. Comparing speciation rates among allopatric, inter-ridge sister taxa versus sympatric within-ridge sister taxa would illuminate the role of secondary sympatry in their speciation (Coyne and Orr 1997), opening the way to an evaluation of the role of sexual behavior in *Mecyclothorax* speciation.

The generalized predatory life cycle of *Mecyclothorax* carabid beetles ensures that their populations can persist within habitats that support at least some suitable prey items, though the menu of prey available over time may change. Small-sized adult beetles can develop via a limited number of feeding events during the larval stadia, ensuring this persistent occupation of small habitat patches by the various species. Thus population extinction in areas of suitable habitat may be minimized. This temporal persistence was recognized by Southwood (1977) as one of the essential factors producing sedentary taxa. Conversely, given that sedentary taxa are less able to accommodate climatic change by dispersing to newly suitable habitats, the presence of flightless taxa suggests that the habitats themselves have persisted through evolutionary time. A second factor, spatial variance in habitat favorableness, is facilitated in Tahiti by the limitation of habitats suitable for *Mecyclothorax* habitation. Within limited elevational bands along a ridge, habitat parameters may be relatively uniform. However such favorableness is lost moving off the ridge to a valley, or with elevational change along the ridge in association with different precipitation regimes, soil types, and forest structure. As long as populations persist over small, isolated ranges, a species may evolve and diverge from neighboring sets of populations. Habitat isolation represents Southwood’s third factor facilitating philopatry. Tahiti’s isolation in the Pacific Ocean represents the largest scale at which this factor has operated. This isolation has permitted relatively few successful colonizing taxa resulting in a disharmonic fauna (Zimmerman 1948). Considering the native French Polynesian carabid fauna derived via autochthonous speciation, the 108 *Mecyclothorax* beetle species share the archipelago with only two *Colpodes* species and *Metacolpodes monticola* (Fairmaire) (tribe Platynini: Perrault 1977; Liebherr 2005a; Nishida 2008), and a single species of *Bembidion* (tribe Bembidiini: Liebherr and Maddison 2013). Disharmony also extends to possible competitors, as Tahiti supports only one potentially native ant species; the rarely encountered *Oligomyrmex tahitiensis* Wheeler (Wilson and Taylor 1967; Perrault 1976, 1987). Thus competition between *Mecyclothorax* beetles and socially predaceous ants, as occurs in Australia, did not occur prehistorically in Tahiti. This competitive naivety of island species plays out in Hawaii where *Mecyclothorax* populations are impacted adversely by alien invasive ants (Cole et al. 1992; Liebherr and Krushelnycky 2007). Thus the flight-capable *Mecyclothorax* colonist (Liebherr 2012a) left Australia to establish in a Tahiti that lacked many of the dominant terrestrial predators regulating Australian *Mecyclothorax* populations. Thereupon the flightless condition quickly became fixed in the burgeoning radiation. The progressive dissection of Tahiti since that colonization event has intensified this isolation, resulting in ever more species speciating in ever diminishing kingdoms.

## References

[B1] Baehr M (2003) Psydrine ground beetles (Coleoptera: Carabidae: Psydrinae), excluding Amblytelini, of eastern Queensland rainforests. Memoirs of the Queensland Museum 49: 65–109.

[B2] Baehr M (2009) A new species of the genus *Mecyclothorax* Sharp from New South Wales (Insecta: Carabidae: Psydrinae). Records of the Australian Museum 61: 89-92. doi: 10.3853/j.0067-1975.61.2009.1519

[B3] Ball GE Shpeley D (1983) The species of eucheiloid Pericalina: classification and evolutionary considerations (Coleoptera: Carabidae: Lebiini). Canadian Entomologist 115: 743-806. doi: 10.4039/Ent115743-7

[B4] Bils W (1976) Das Abdomenende weiblicher, terrestrisch lebender Adephaga (Coleoptera) und seine Bedeutung für die Phylogenie. Zoomorphologie 84: 113-193. doi: 10.1007/BF00999711

[B5] Britton EB (1938) Carabidae of the Society Islands and Rapa (Coleoptera).Occasional Papers of Bernice P. Bishop Museum 14: 103-110.

[B6] Britton EB (1948) A revision of the Hawaiian species of *Mecyclothorax* (Coleoptera: Carabidae). Occasional Papers of Bernice P. Bishop Museum 19: 107-166.

[B7] Carson HL Kaneshiro KY (1976) *Drosophila* of Hawaii: systematics and ecological genetics. Annual Review of Ecology and Systematics 7: 311-345. doi: 10.1146/annurev.es.07.110176.001523

[B8] Cohen AS Soreghan MJ Scholz CA (1993) Estimating the age of formation of lakes: an example from Lake Tanganyika, East African Rift system. Geology 21: 511-514. doi: 10.1130/0091-7613(1993)021<0511:ETAOFO>2.3.CO;2

[B9] Cole FR Medeiros AC Loope LL Zuhlke WM (1992) Effects of the Argentine ant on arthropod fauna of Hawaiian high-elevation shrubland. Ecology 73: 1313-1322. doi: 10.2307/1940678

[B10] Coyne JA Orr HA (1997) “Patterns of speciation in Drosophila” revisited. Evolution 51: 295–303. doi: 10.2307/2410984 28568795

[B11] Coyne JA Orr HA (2004) Speciation. Sunderland, MA, Sinauer Associates, Inc. Publishers.

[B12] DeSalle R (1992) The origin and possible time of divergence of the Hawaiian Drosophilidae: evidence from DNA sequences. Molecular Biology and Evolution 9: 905-916. 1528112 10.1093/oxfordjournals.molbev.a040767

[B13] Deuve T (1993) L’abdomen et les genitalia des femelles des Coléoptères Adephaga. Mémoires du Muséum National d’Histoire Naturelle Serie A Zoologie 155: 1-184.

[B14] Fryer G (2001) On the age of origin of the species flock of haplochromine cichlid fishes of Lake Victoria. Proceedings of the Royal Society of London (B) 268: 1147-1152. doi: 10.1098/rspb.2001.1601 PMC1088720 11375102

[B15] Futuyma DJ (1986) Evolutionary Biology. Sunderland, MA, Sinauer Associates Inc. Publishers

[B16] Futuyma DJ (2009) Evolution. Sunderland, MA, Sinauer Associates, Inc. Publishers.

[B17] Genner MJ Seehausen O Lunt DH Joyce DA Shaw PW Carvalho GR Turner GF (2007) Age of cichlids: new dates for ancient lake fish radiations. Molecular Biology and Evolution 24: 1269-1282. doi: 10.1093/molbev/msm050 17369195

[B18] Guillou H Maury RC Blais S Cotten J Legendre C Guille G Caroff M (2005) Age progression along the Society hotspot chain (French Polynesia) based on new unspiked K-Ar ages. Bulletin de la Société Géologique de France 176: 135-150. doi: 10.2113/176.2.135

[B19] Hardy DE (1965) Diptera: Cyclorrhapha II. Series Schizophora, Section Acalypterae I, Family Drosophilidae. Insects of Hawaii 12: vii + 814 pp.

[B20] Hardy DE (1966) Descriptions and notes on Hawaiian Drosophilidae (Diptera). Studies in Genetics No. 3 (University of Texas Publication 6615): 195– 244.

[B21] Hardy DE (1977) Review of the Hawaiian *Drosophila (Antopocerus)* Hardy. Proceedings of the Entomological Society of Washington 79: 82-95.

[B22] Hardy DE (1978) A new symmorphic sibling species of *Drosophila* (Diptera) from the island of Maui, Hawaii. American Midland Naturalist 99: 350-351. doi: 10.2307/2424811

[B23] Hardy DE Kaneshiro KY (1968) New picture-winged *Drosophila* from Hawaii. Studies in Genetics No. 4 (University of Texas Publication 6818): 171– 262.

[B24] Hardy DE Kaneshiro KY (1969) Descriptions of New Hawaiian *Drosophila*. Studies in Genetics 5 (University of Texas Publication 6918): 39– 54.

[B25] Hardy DE Kaneshiro KY (1971) New picture-winged *Drosophila* from Hawaii, part II. (Drosophilidae, Diptera). Studies in Genetics 6 (University of Texas Publication 7103): 151– 170.

[B26] Hardy DE Kaneshiro KY (1972) New picture-winged *Drosophila* from Hawaii, part III (Drosophilidae, Diptera). Studies in Genetics 7 (University of Texas Publication 7213): 155– 161.

[B27] Hardy DE Kaneshiro KY (1975) Studies in Hawaiian *Drosophila*, miscellaneous new species, no. I. Proceedings of the Hawaiian Entomological Society 22: 57-64.

[B28] Hardy DE Kaneshiro KY (1979) A review of the modified tarsus species group of Hawaiian *Drosophila* (Drosophilidae: Diptera) I. the “split-tarsus” subgroup. Proceedings of the Hawaiian Entomological Society 23: 71-90.

[B29] Hardy DE Kaneshiro KY Val FC O’Grady PM (2001) Review of the *haleakalae* species group of Hawaiian *Drosophila* (Diptera: Drosophilidae). Bishop Museum Bulletins in Entomology 9: viii + 88 pp.

[B30] Hildenbrand A Gillot P-Y Le Roy I (2004) Volcano-tectonic and geochemical evolution of an oceanic intra-plate volcano: Tahiti-Nui (French Polynesia). Earth and Plaetary Science Letters 217: 349-365. doi: 10.1016/S0012-821X(03)00599-5

[B31] Hildenbrand A Gillot P-Y Marlin C (2008) Geomorphological study of long-term erosion on a tropical volcanic ocean island: Tahiti-Nui (French Polynesia). Geomorphology 93: 460– 481. doi: 10.1016/j.geomorph.2007.03.012

[B32] Hoelzer GA Drewes R Meier J Doursat R (2008) Isolation-by-distance and outbreeding depression are sufficient to drive parapatric speciation in the absence of environmental influences. PLOS Computational Biology 4: e1000126, 11 pp. 10.1371/journal.pcbi.1000126 PMC2440541 18654617

[B33] IGN [Institut Géographique National] (1994) Tahiti, Archipel de la Société, Carte Touristique au 1:100,000. Paris.

[B34] IPPC (International Plant Protection Convention) (2005) *Wasmannia auropunctata*, little fireant, established in French Polynesia and control measures. http://www.ippc.int/file_uploaded/1121803401792_Wasmannia_en.pdf. [accessed 27 March, 2013]

[B35] Iuga VG Rosça A (1966) Morphologie du sommet abdominal des Caraboïdes, comparé à celui des Coléoptères avec ovipositeur. Travaux du Muséum d’Histoire Naturelle ‘Grigore Antipa’ 6: 171-226.

[B36] Jeannel R (1941) Coléoptères Carabiques, première partie. Faune de France 39: 1-571.

[B37] Kambysellis MP Ho K-F Craddock EM Piano F Parisi M Cohen J (1995) Pattern of ecological shifts in the diversification of Hawaiian *Drosophila* inferred from a molecular phylogeny. Current Biology 5: 1129-1139. doi: 10.1016/S0960-9822(95)00229-6 8548285

[B38] Kaneshiro KY (1988) Speciation in the Hawaiian *Drosophila*, sexual selection appears to play an important role. Bioscience 38: 258-263. doi: 10.2307/1310849

[B39] Kaneshiro KY Gillespie RG Carson HL (1995) Chromosomes and male genitalia of Hawaiian *Drosophila*: tools for interpreting phylogeny and geography. In: Wagner WL Funk VA (Eds). Hawaiian Biogeography: Evolution on a Hot Spot Archipelago. Smithsonian Institution Press, Washington: 57-71.

[B40] Kaneshiro KY Kambysellis MP (1999) Description of a new allopatric sibling species of Hawaiian picture-winged *Drosophila*. Pacific Science 53: 208-213.

[B41] Koblmüller S Schliewen UK Duftner N Sefc KM Katongo C Sturmbauer C (2008) Age and spread of the haplochromine cichlid fishes in Africa. Molecular Phylogenetics and Evolution 49: 153-169. doi: 10.1016/j.ympev.2008.05.045 18582582

[B42] Kukalová-Peck J Lawrence JF (1993) Evolution of the hind wing in Coleoptera. Canadian Entomologist 125: 181-258. doi: 10.4039/Ent125181-2

[B43] LaPoint RT Magnacca KN O’Grady PM (2009) Review of the spoon tarsus subgroup of Hawaiian *Drosophila* (Drosophilidae: Diptera), with a description of one new species. Zootaxa 2003: 53-68.

[B44] Liebherr JK (2005a) Platynini (Coleoptera: Carabidae) of Vanuatu: Miocene diversification on the Melanesian Arc. Invertebrate Systematics 19: 263-295. doi: 10.1071/IS04032

[B45] Liebherr JK (2005b) New species of *Mecyclothorax* (Coleoptera: Carabidae, Psydrini) from Polipoli, Maui define an area of endemism on Haleakala Volcano, Hawaii. Journal of the New York Entomological Society 113: 97-128. doi: 10.1664/0028-7199(2005)113[0097:NSOMCC]2.0.CO;2

[B46] Liebherr JK (2006) Taxonomic revision of the *Mecyclothorax* beetles (Coleoptera: Carabidae, Psydrini) of Molokai, Hawaii and recognition of areas of endemism on Kamakou volcano. Journal of the New York Entomological Society 114: 179–281. doi: 10.1664/0028-7199(2007)114[179:TROTMB]2.0.CO;2

[B47] Liebherr JK (2008) Taxonomic revision of *Mecyclothorax* Sharp (Coleoptera, Carabidae) of Hawaii Island: abundant genitalic variation in a nascent island radiation. Deutsche Entomologische Zeitshrift 55: 19-78. doi: 10.1002/mmnd.200800004

[B48] Liebherr JK (2009) Taxonomic revision of the *Mecyclothorax* beetles (Coleoptera: Carabidae) of Oahu: epithets as epitaphs for an endangered fauna? Systematic Entomology 34: 649–687. doi: 10.1111/j.1365-3113.2009.00477.x

[B49] Liebherr JK (2011) The *Mecyclothorax* beetles (Coleoptera: Carabidae: Moriomorphini) of West Maui, Hawaii: taxonomy, biogeography, and conservation. Deutsche Entomologische Zeitschrift 58: 15-76. doi: 10.1002/mmnd.201100005

[B50] Liebherr JK (2012a) The first precinctive Carabidae from Moorea, Society Islands: new *Mecyclothorax* spp. (Coleoptera) from the summit of Mont Tohiea. ZooKeys 224: 37-80. doi: 10.3897/zookeys.224.3675 PMC3487645 23129989

[B51] Liebherr JK (2012b) New *Mecyclothorax* spp. (Coleoptera: Carabidae: Moriomorphini) define Mont Mauru, eastern Tahiti Nui, as a distinct area of endemism. ZooKeys 227: 63-99. doi: 10.3897/zookeys.227.3797 PMC3487650 23166465

[B52] Liebherr JK Krushelnycky PD (2007) Unfortunate encounters? Novel interactions of native *Mecyclothorax*, alien *Trechus obtusus* (Coleoptera: Carabidae), and Argentine ant (*Linepithema humile*, Hymenoptera: Formicidae) across a Hawaiian landscape. Journal of Insect Conservation 11: 61-73. doi: 10.1007/s10841-006-9019-8

[B53] Liebherr JK Krushelnycky PD (2011) *Mecyclothorax palikea* sp. n. from the Waianae Range, Oahu, and the biogeographical history of Hawaii’s *M. flavomarginatus* species group (Coleoptera: Carabidae: Moriomorphini). Insect Systematics and Evolution 42: 365– 384. doi: 10.1163/187631211X608718

[B54] Liebherr JK Maddison DR (2013) Colonisation of the Pacific by *Bembidion* beetles (Coleoptera: Carabidae), with description of *Bembidion tahitiense*, sp. nov., from Tahiti, French Polynesia. Invertebrate Systematics 27: 11 pp. doi: 10.1071/IS13003

[B55] Liebherr JK Will KW (1998) Inferring phylogenetic relationships within Carabidae (Insecta, Coleoptera) from characters of the female reproductive tract. In: Ball GE Casale A Vigna Taglianti V (Eds). Phylogeny and classification of Caraboidea (Coleoptera: Adephaga). Proceedings of a symposium (28 August 1996, Florence, Italy). 20 International Congress of Entomology, Atti Museo Regionale di Scienze Naturali (Museo Regionale di Scienze Naturali–Torino, Torino): 107-170.

[B56] Lindroth CH (1974) On the elytral microsculpture of carabid beetles (Col. Carabidae). Entomologica Scandinavica 5: 251-264. doi: 10.1163/187631274X00290

[B57] Loope LL Krushelnycky PD (2007) Current and potential ant impacts in the Pacific Region. Proceedings of the Hawaiian Entomological Society 39: 69-73.

[B58] Maddison DR (1993) Systematics of the Holarctic beetle subgenus *Bracteon* and related *Bembidion* (Coleoptera: Carabidae). Bulletin of the Museum of Comparative Zoology 153: 143–299.

[B59] Magnacca KN O’Grady PM (2008a) Revision of the ‘nudidrosophila’ and ‘ateledrosophila’ species groups of Hawaiian *Drosophila* (Diptera: Drosophilidae), with descriptions of twenty-two new species. Systematic Entomology 33: 395-428. doi: 10.1111/j.1365-3113.2007.00400.x

[B60] Magnacca KN O’Grady PM (2008b) New combinations in Hawaiian *Drosophila* and *Scaptomyza* (Diptera: Drosophilidae). Zootaxa 1926: 53-60.

[B61] Magnacca KN O’Grady PM (2009) Revision of the modified mouthparts species group of Hawaiian *Drosophila* (Diptera: Drosophilidae) the *ceratostoma*, *freycinetiae*, *semifuscata*, and *setiger* subgroups, and unplaced species. University of California Publications in Entomology 139: viii + 1–94.

[B62] Magnacca KN Price DK (2012) New species of Hawaiian picture wing *Drosophila* (Diptera: Drosophilidae), with a key to species. Zootaxa 3188: 1-30.

[B63] Mendelson TC Shaw KL (2005) Rapid speciation in an arthropod. Nature 433: 375-376. doi: 10.1038/433375a 15674280

[B64] Moore BP (1984) Taxonomic notes on some Australian *Mecyclothorax* Sharp (Coleoptera: Carabidae: Psydrinae) and descriptions of new species. Journal of the Australian Entomological Society 23: 161-166. doi: 10.1111/j.1440-6055.1984.tb01935.x

[B65] Moore BP Weir TA Pyke JE (1987) Coleoptera: Adephaga: Rhysodidae and Carabidae. In: Walton DW (Eds) Zoological Catalogue of Australia 4: 17– 320. Australian Government Printing Service, Canberra.

[B66] Moore JG Clague DA Holcomb RT Lipman PW Normark WR Torresan ME (1989) Prodigious submarine landslides on the Hawaiian Ridge. Journal of Geophysical Research B94: 17,465– 417,484.

[B67] Nishida GM (2008) Checklists: French Polynesia (27), French Polynesia Beetle Checklist, Version 2008 November 19. Electronic Word® document available at: essig.db.berkeley.edu. Essig Museum of Entomology, Berkeley Natural History Museums, University of California, Berkeley. [accessed 2 May 2013]

[B68] O’Grady P Bonacum J DeSalle R Val Fd (2003a) The placement of *Engiscaptomyza*, *Grimshawomyia*, and *Titanochaeta*, three clades of endemic Hawaiian Drosophilidae (Diptera). Zootaxa 159: 1-16.

[B69] O’Grady PM Kam MWY Val FC Perreira WD (2003b) Revision of the *Drosophila mimica* subgroup, with descriptions of ten new species. Annals of the Entomological Society of America 96: 12-38. doi: 10.1603/0013-8746(2003)096[0012:ROTDMS]2.0.CO;2

[B70] O’Grady PM Val FC Hardy DE Kaneshiro KY (2001) The rustica species group of Hawaiian *Drosophila* (Diptera: Drosophilidae). Pan-Pacific Entomologist 77: 254-260.

[B71] Perrault GG (1977) La faune des Carabidae de Tahiti. Nouvelle Revue d’Entomologie 7: 283-289.

[B72] Perrault GG (1978a) La faune des Carabidae de Tahiti II. genre *Mecyclothorax* (Sharp). Nouvelle Revue d’Entomologie 8: 27-36.

[B73] Perrault GG (1978b) La faune des Carabidae de Tahiti II. genre *Mecyclothorax* (Sharp). Nouvelle Revue d’Entomologie 8: 133-162.

[B74] Perrault GG (1984) La faune des Carabidae de Tahiti VI. révision du genre *Mecyclothorax* (Sharp) (Psydrini). 1. le groupe de *M. muriauxi* Perrault (Coleoptera). Nouvelle Revue d’Entomologie (NS) 1: 19-31.

[B75] Perrault GG (1986) La faune des Carabidae de Tahiti VII. révision du genre *Mecyclothorax* (Sharp) (Psydrini). 2. les groupes de *M. striatopunctatus* n. sp., *M. dannieae* Perrault, *M. marginatus* Perrault et *M. viridis* Perrault (Coleoptera). Nouvelle Revue d’Entomologie (NS) 3: 439-455.

[B76] Perrault GG (1987) Microendemisme et speciation du genre *Mecyclothorax* (Coleoptera – Carabidae Psydrini) à Tahiti. Bulletin de la Société Zoologique de France 112: 419-427

[B77] Perrault GG (1988) La faune des Carabidae de Tahiti. VIII. révision du genre *Mecyclothorax* Sharp (Psydrini) 3. les groups de *M. altiusculus* Britton et de *M. gourvesi* Perrault (Coleoptera). Nouvelle Revue d’Entomologie (NS) 5: 229-245.

[B78] Perrault GG (1989) La faune des Carabidae de Tahiti: IX. révision du genre *Mecyclothorax* (Sharp) (Psydrini) 4. le groupe de *M. globosus* Britton (Coleoptera). Nouvelle Revue d’Entomologie (NS) 6: 57-70.

[B79] Perrault GG (1992) Endemism and biogeography among Tahitian *Mecyclothorax* species (Coleoptera: Carabidae: Psydrini). In: Noonan GR Ball GE Stork NE (Eds). The Biogeography of Ground Beetles of Mountains and Islands. Intercept, Ltd., Andover, Hampshire, UK: 201-215.

[B80] Perrault GH (1976) Description des ouvrières et des soldats de *Oligomyrmex tahitiensis* Wheeler. mise au point concernant les sexués. Nouvelle Revue d’Entomologie 6: 303-307.

[B81] Perrault GH (1987) Les fourmis de Tahiti. Bulletin de la Société Zoologique de France 112: 429–446.

[B82] Perreira WD Kaneshiro KY (1990) Three new species of picture-winged *Drosophila* from the Hawaiian Islands. Proceedings of the Hawaiian Entomological Society 30: 79-84.

[B83] RGPF [Réseau Géodésique de Polynésie Française] (2001) Ile de Tahiti, Communes de Taiarapu Est et Ouest, Edition Provisionaire, Fonds Topographique, scale 1/25,000. Papeete.

[B84] Russo CAM Takezaki N Nei M (1995) Molecular phylogeny and divergence times of drosophilid species. Molecular Biology and Evolution 12: 391-404. 7739381 10.1093/oxfordjournals.molbev.a040214

[B85] Sharp D (1903) Coleoptera II. Caraboidea. Fauna Hawaiiensis 3: 175– 292 + 2 pls.

[B86] Sharp D Muir FAG (1912) The comparative anatomy of the male genital tube in Coleoptera. Transactions of the Entomological Society of London 1912: 477– 642 + 437 pls.

[B87] Sherrod DR Nishimitsu Y Tagami T (2003) New K-Ar ages of the geological evidence against rejuvenated-stage volcanism at Haleakalā, East Maui, a post-shield-stage volcano of the Hawaiian island chain. Geological Society of America Bulletin 115: 683-694. doi: 10.1130/0016-7606(2003)115<0683:NKAATG>2.0.CO;2

[B88] Sherrod DR Sinton JM Watkins SE Brunt KM (2007) Geological Map of the State of Hawai`i. U.S. Geological Survey Open-File Report 2007-1089, Version 1.0 (22 May 2007) http://pubs.usgs.gov/of/2007/1089/ [accessed 5 July 2013]

[B89] Southwood TRE (1977) Habitat, the templet for ecological strategies? Journal of Animal Ecology 46: 337–365. doi: 10.2307/3817

[B90] Takahashi T Koblmüller S (2011) The adaptive radiation of cichlid fish in Lake Tanganyika: a morphological perspective. International Journal of Evolutionary Biology 2011: Article ID 620754, 14 pp. 10.4061/2011/620754 PMC3119568 21716857

[B91] Turner GF Seehausen O Knight ME Allender CJ Robinson RL (2001) How many species of cichlid fishes are there in African lakes? Molecular Ecology 10: 793–806. doi: 10.1046/j.1365-294x.2001.01200.x 11298988

[B92] Uto K Yamamoto Y Sudo M Uchiumi S Ishizuka O Kogiso T Tsunakawa H (2007) New K–Ar ages of the Society Islands, French Polynesia, and implications for the Society hotspot feature. Earth Planets Space 59: 879-885.

[B93] Wahlroos S (2002) English–Tahitian Tahitian–English Dictionary. Honolulu, Hawai`i, The Mā`ohi Heritage Press, xxvi + 684 pp.

[B94] Wessel A Hoch H Asche M von Rintelen T Heck V Stone FD Howarth FG (2013) Founder effects initiated rapid species radiation in Hawaii cave planthoppers. Proceedings of the Natinal Academy of Sciences (U.S.A. ) 110: 9391-9396. doi: 10.1073/pnas.1301657110 23696661 PMC3677470

[B95] Wilson EO Taylor RW (1967) The ants of Polynesia (Hymenoptera: Formicidae). Pacific Insects Monograph 14: 1-109.

[B96] Zimmerman EC (1948) Introduction. Insects of Hawaii 1: xx + 206.

